# Supplementary material for: Increased reproducibility of brain organoids through controlled fluid dynamics
Source: EMBO Rep. 2025 Nov 19;26(24):6209–39. doi: 10.1038/s44319-025-00619-x (PMC12715241; doi:10.1038/s44319-025-00619-x)
Supplement: Supplementary file 3 — Data Set EV2 [file 44319_2025_619_MOESM3_ESM.docx]

**Dataset EV2.** Comparison of the morphological parameters’ variability.

| **Morphological parameters** | **p-values (Brown-Forsythe test)** | **Results** |
| --- | --- | --- |
| Area | 1.31ꞏ10^-3^ | ** |
| Perimeter | 1.82ꞏ10^-4^ | *** |
| Average radius | 7.13ꞏ10^-7^ | **** |
| Roundness | 2.28ꞏ10^-7^ | **** |
| Min Feret | 1.05ꞏ10^-5^ | **** |
| Max Feret | 5.33ꞏ10^-7^ | **** |
| Std curvature | 1.16ꞏ10^-9^ | **** |
| Std curvature x R0 | 3.04ꞏ10^-6^ | **** |
| Mean curvature | < 2.2ꞏ10^-16^ | **** |
| Dirichlet Normal Energy | 1.97ꞏ10^-8^ | **** |

**Dataset EV2A. Comparison of the morphological parameters’ variability between PA and RC embryoid bodies at day 6.** The variability was compared using Brown-Forsythe test. R0 = average radius, Sd = standard deviation, ns = non-significative (p > 0.05), * p < 0.05, ** p < 0.01, *** p < 0.001, **** p < 0.0001. n = 102 PA EBs, n = 88 RC EBs.

| **Morphological parameters** | **p-values (Brown-Forsythe test)** | **Results** |
| --- | --- | --- |
| Area | 5.31ꞏ10^-3^ | ** |
| Perimeter | 1.04ꞏ10^-5^ | **** |
| Average radius | 3.61ꞏ10^-6^ | **** |
| Roundness | 0.27 | ns |
| Min Feret | 9.00ꞏ10^-4^ | *** |
| Max Feret | 5.29ꞏ10^-6^ | **** |
| Std curvature | 0.76 | ns |
| Std curvature x R0 | 1.80ꞏ10^-4^ | *** |
| Mean curvature | 1.55ꞏ10^-3^ | ** |
| Dirichlet Normal Energy | 2.37ꞏ10^-7^ | **** |

**Dataset EV2B. Comparison of the morphological parameters’ variability between OS and RC organoids at day 90.** The variability was compared using Brown-Forsythe test. R0 = average radius, Sd = standard deviation, ns = non-significative (p > 0.05), * p < 0.05, ** p < 0.01, *** p < 0.001, **** p < 0.0001. n = 67 OS organoids, n = 41 RC organoids.
