# Supplementary material for: Increased reproducibility of brain organoids through controlled fluid dynamics
Source: EMBO Rep. 2025 Nov 19;26(24):6209–39. doi: 10.1038/s44319-025-00619-x (PMC12715241; doi:10.1038/s44319-025-00619-x)
Supplement: Supplementary file 7 — Movie EV1 [file 44319_2025_619_MOESM7_ESM.zip › Movie EV1.rtf]

Movie EV1. Particle trajectories and surface velocity magnitude.
