# Supplementary figures and images for: Increased reproducibility of brain organoids through controlled fluid dynamics (part 1 of 2)
Source: EMBO Rep. 2025 Nov 19;26(24):6209–39. doi: 10.1038/s44319-025-00619-x (PMC12715241; doi:10.1038/s44319-025-00619-x)

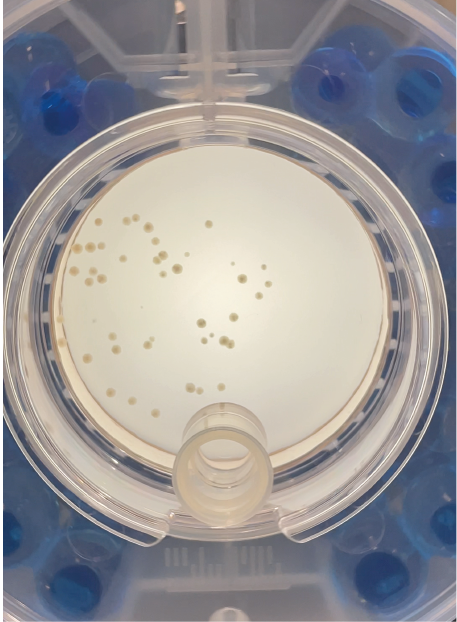

Supplement: Supplementary file 9 — Source data Fig. 1 [file 44319_2025_619_MOESM9_ESM.zip › Figure 1/D/Frame_Movie.png]

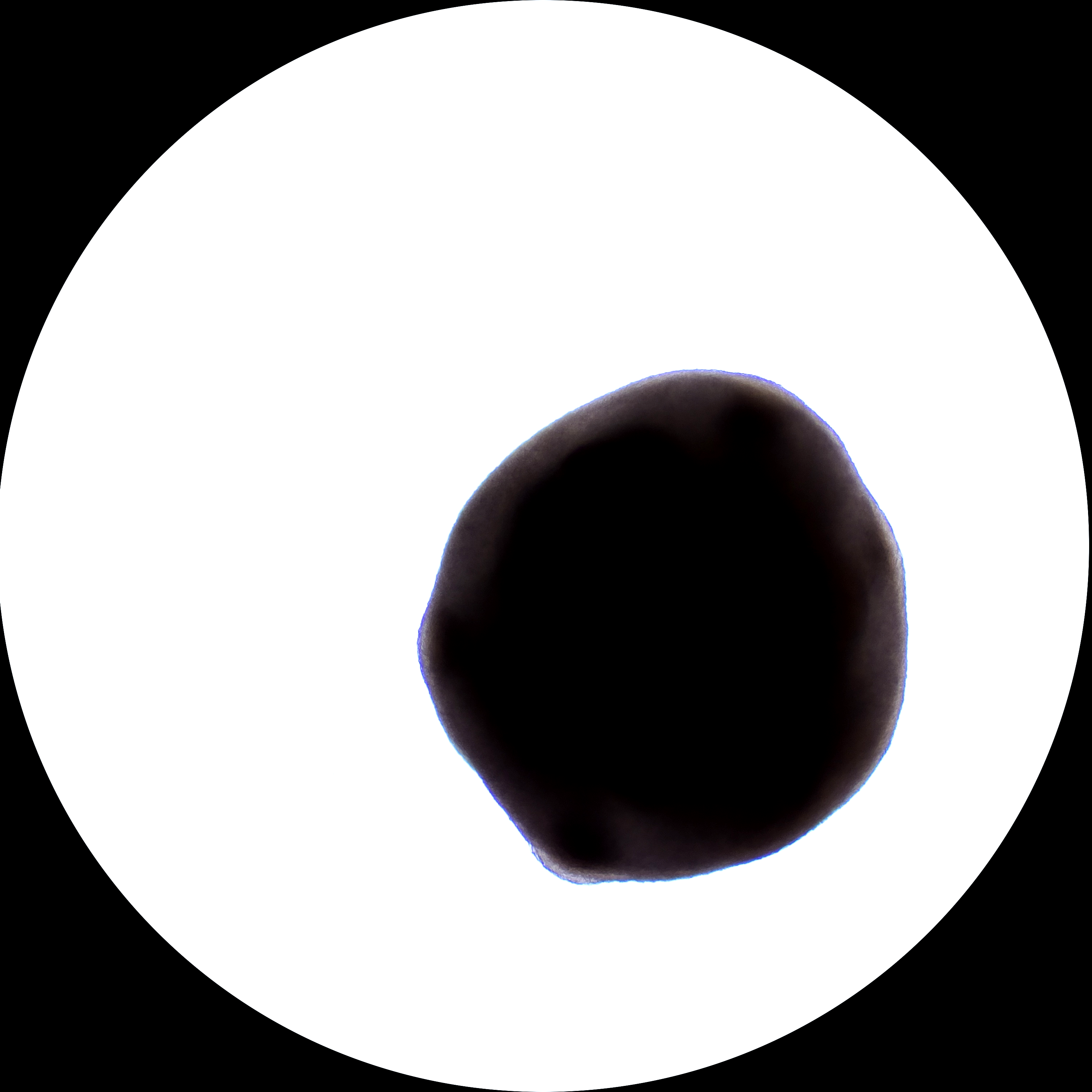

Supplement: Supplementary file 10 — Source data Fig. 2 [file 44319_2025_619_MOESM10_ESM.zip › Figure 2/A/Day 25_0000.tif]

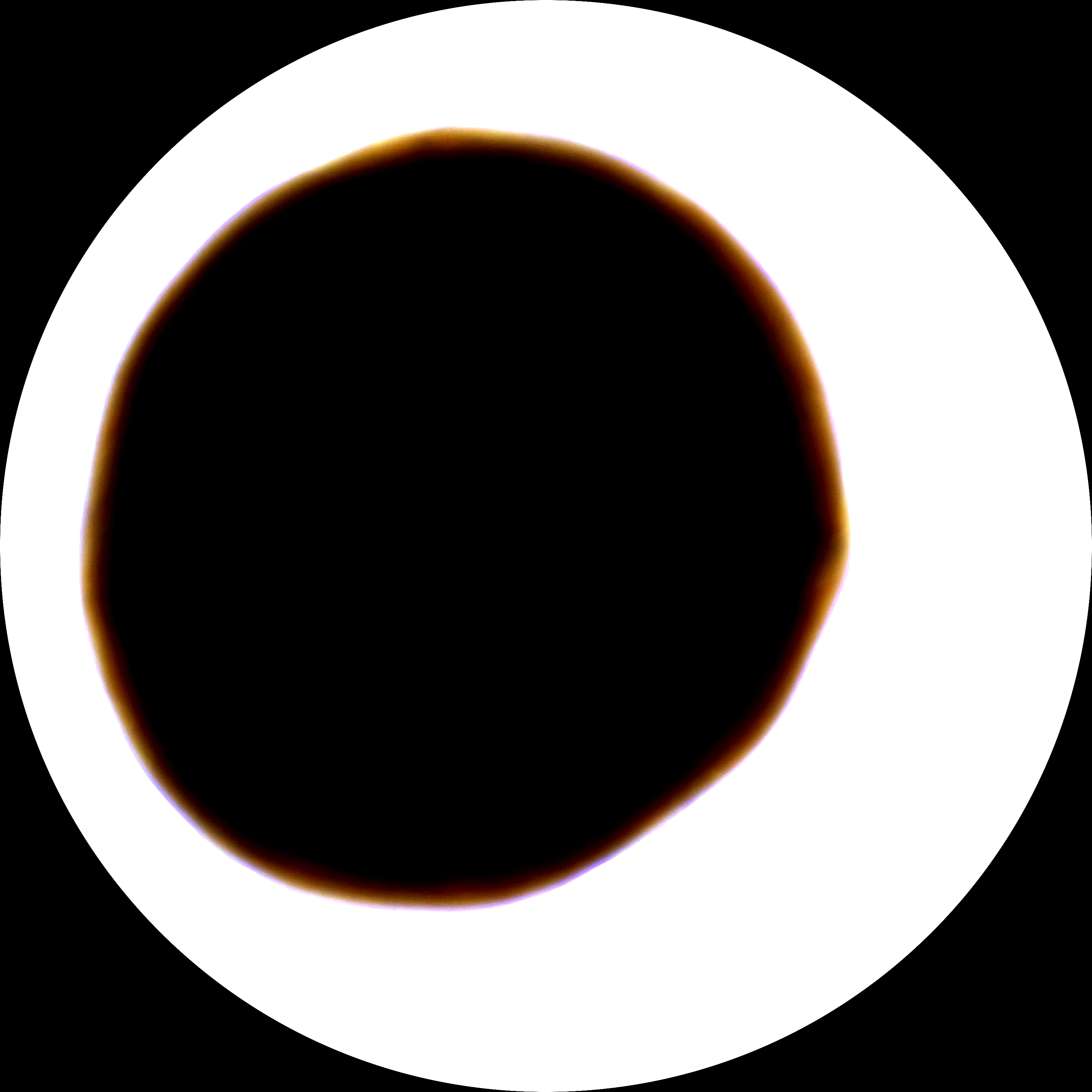

Supplement: Supplementary file 10 — Source data Fig. 2 [file 44319_2025_619_MOESM10_ESM.zip › Figure 2/A/Day90_0007.tif]

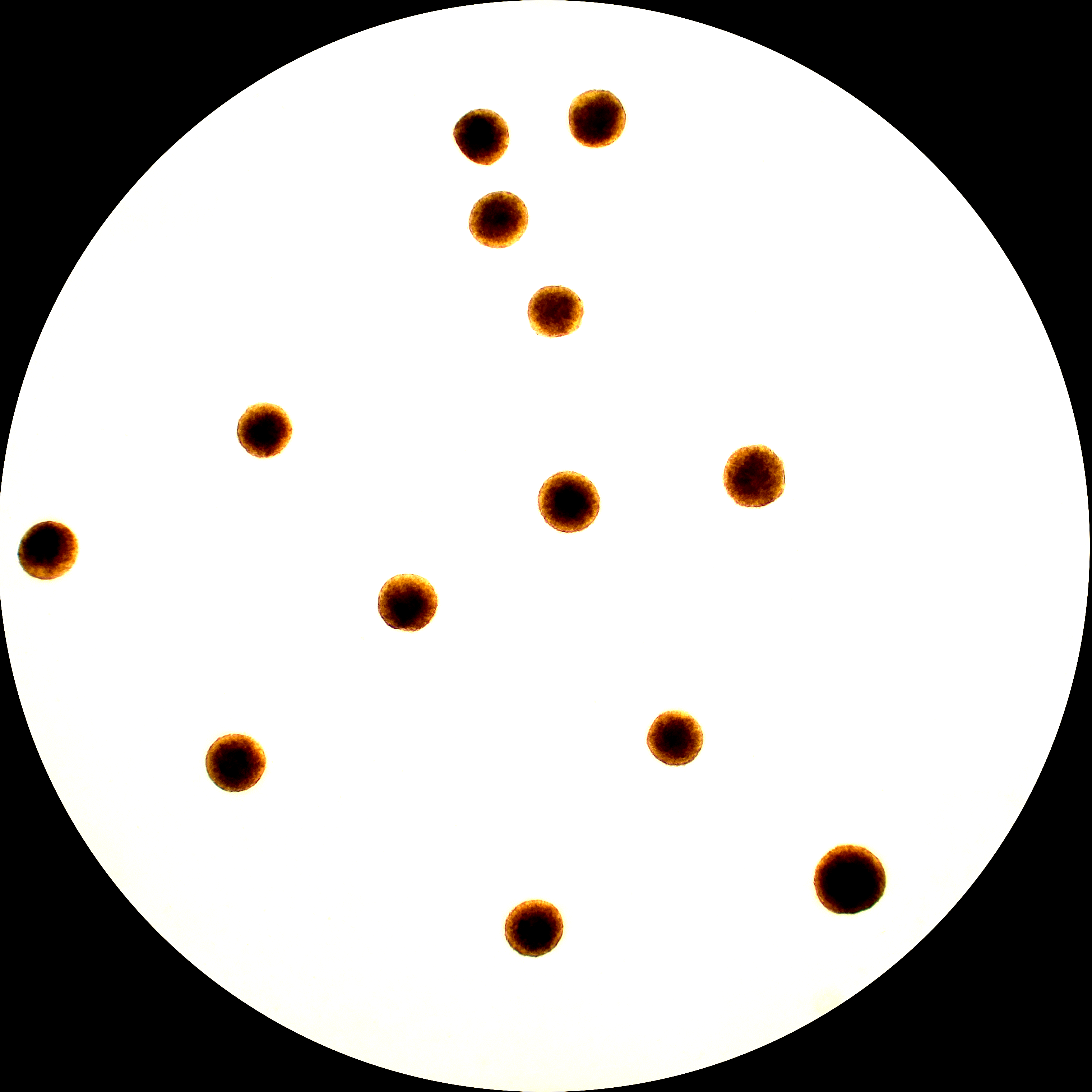

Supplement: Supplementary file 10 — Source data Fig. 2 [file 44319_2025_619_MOESM10_ESM.zip › Figure 2/A/Day 6_0001.tif]

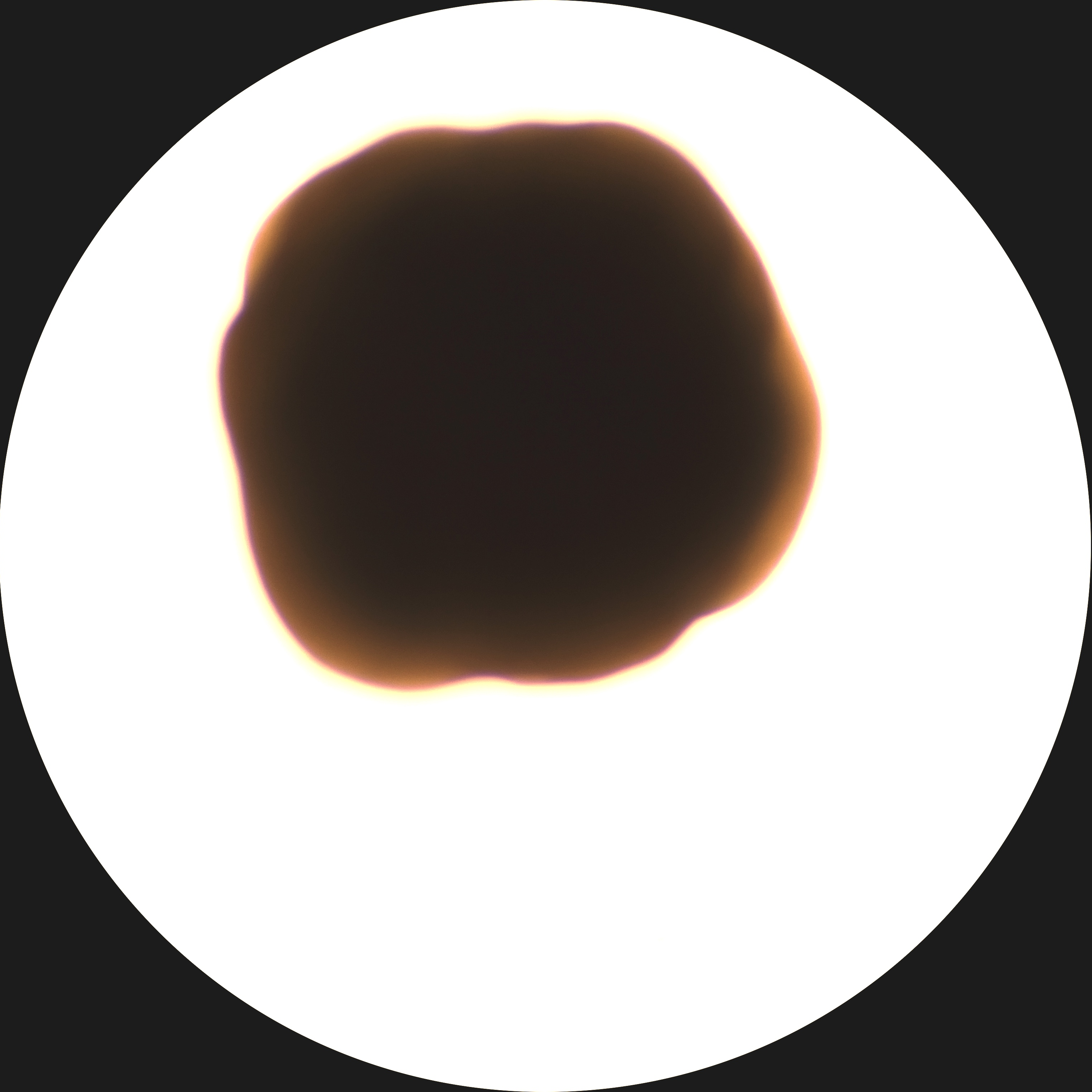

Supplement: Supplementary file 10 — Source data Fig. 2 [file 44319_2025_619_MOESM10_ESM.zip › Figure 2/A/Day 45_0003.tif]

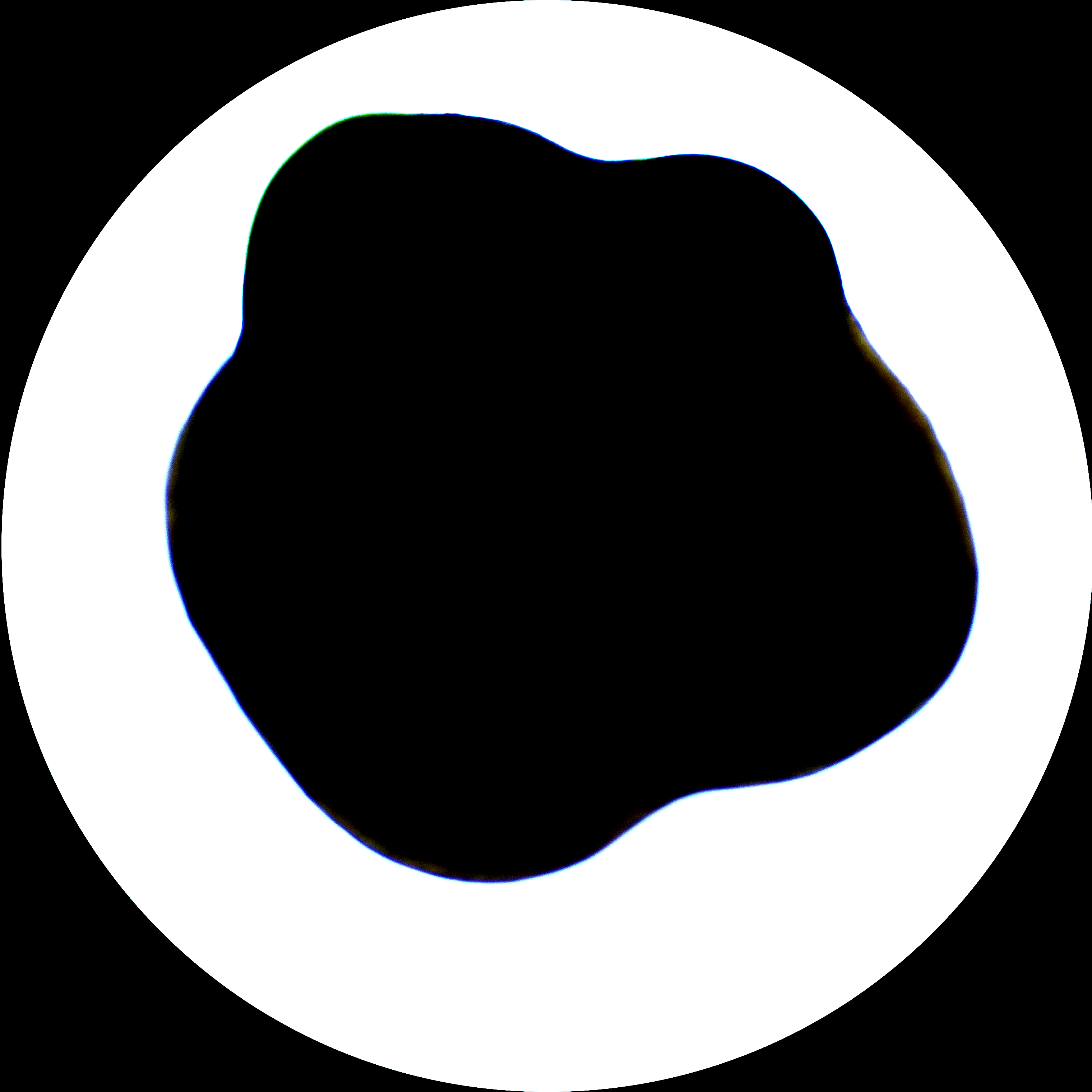

Supplement: Supplementary file 11 — Source data Fig. 3 [file 44319_2025_619_MOESM11_ESM.zip › Figure 3/E/OS/MN_B_11C1_C7_D90_0016 original.tif]

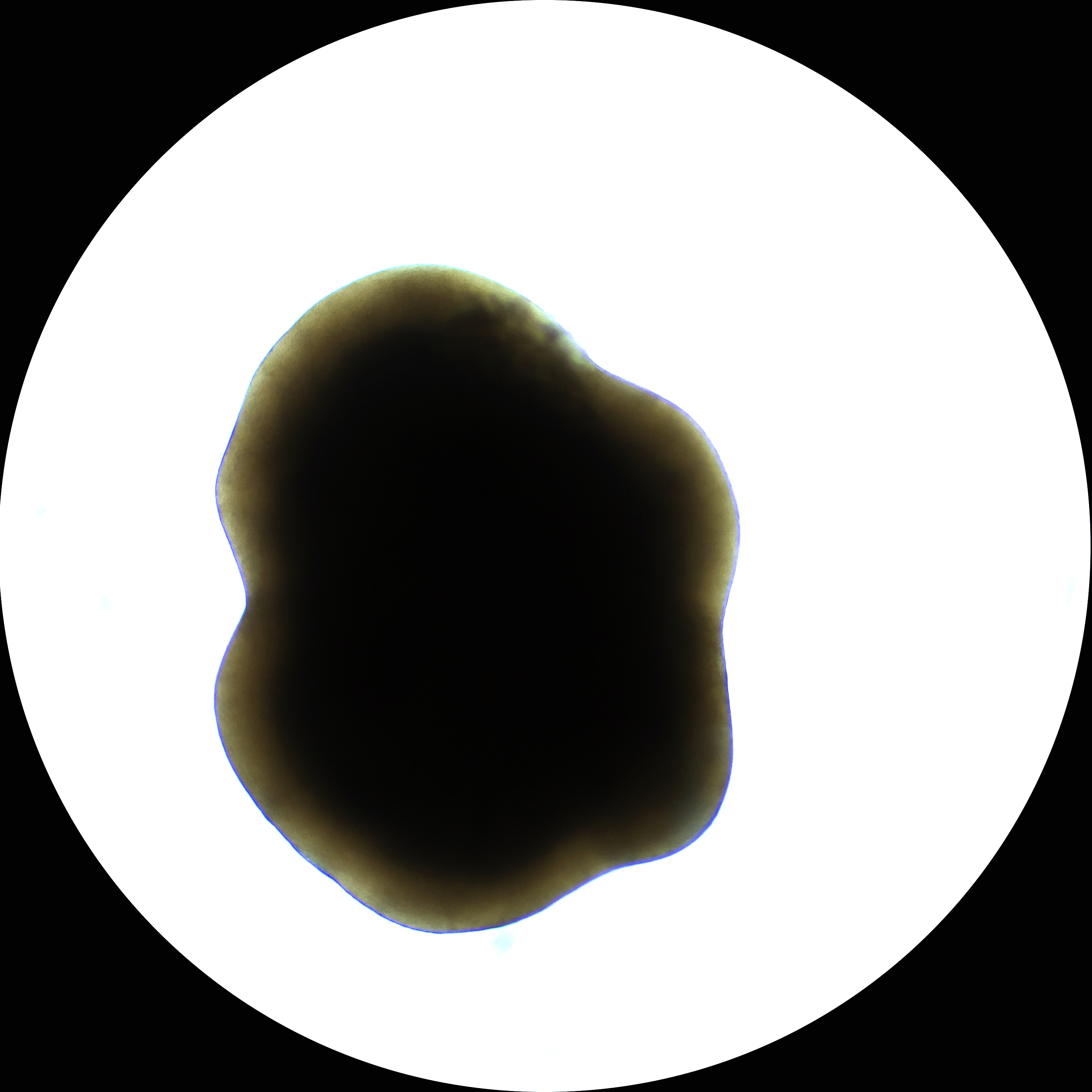

Supplement: Supplementary file 11 — Source data Fig. 3 [file 44319_2025_619_MOESM11_ESM.zip › Figure 3/E/OS/Day90_0000.tif]

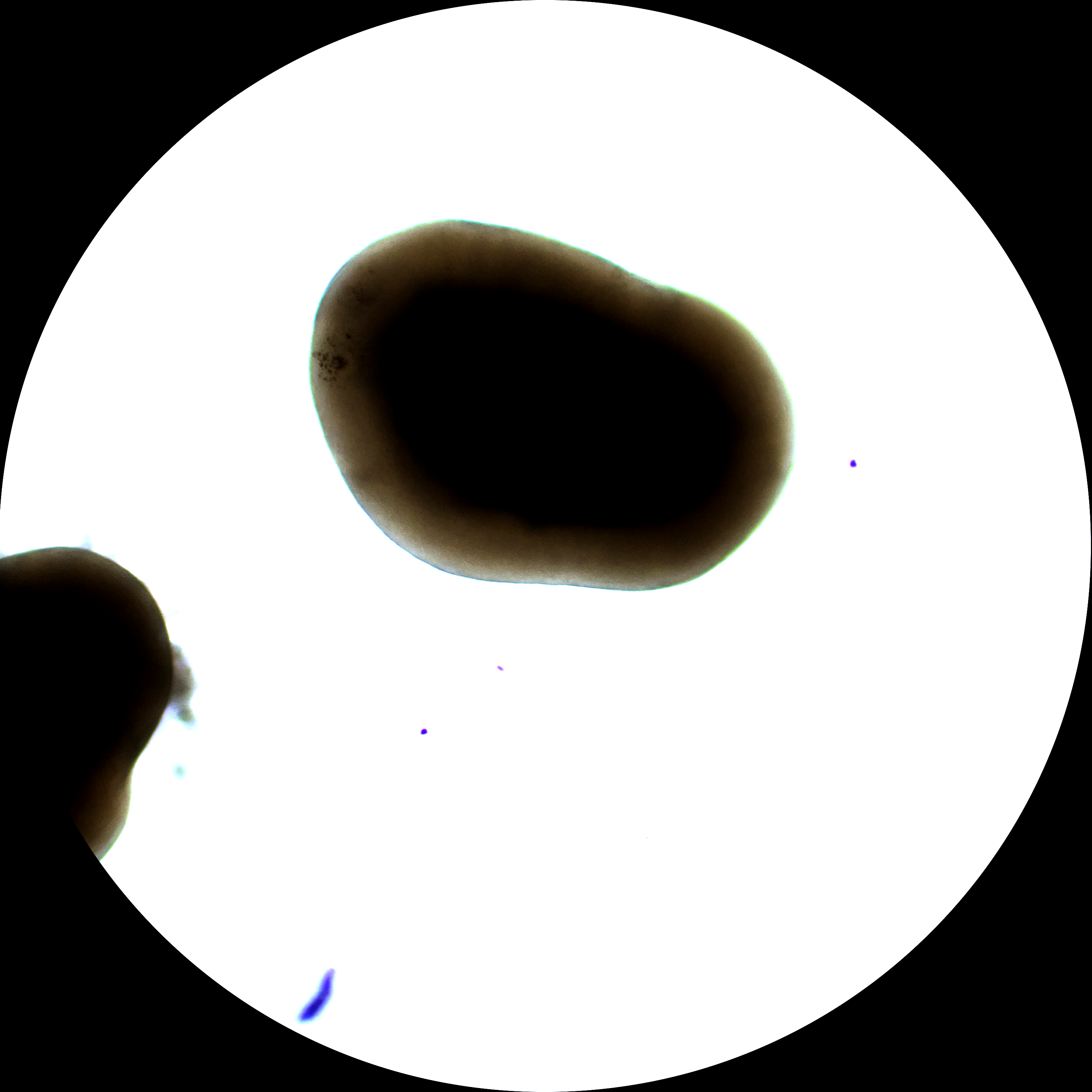

Supplement: Supplementary file 11 — Source data Fig. 3 [file 44319_2025_619_MOESM11_ESM.zip › Figure 3/E/OS/MN_B_12C1_C4_D90_0001 original.tif]

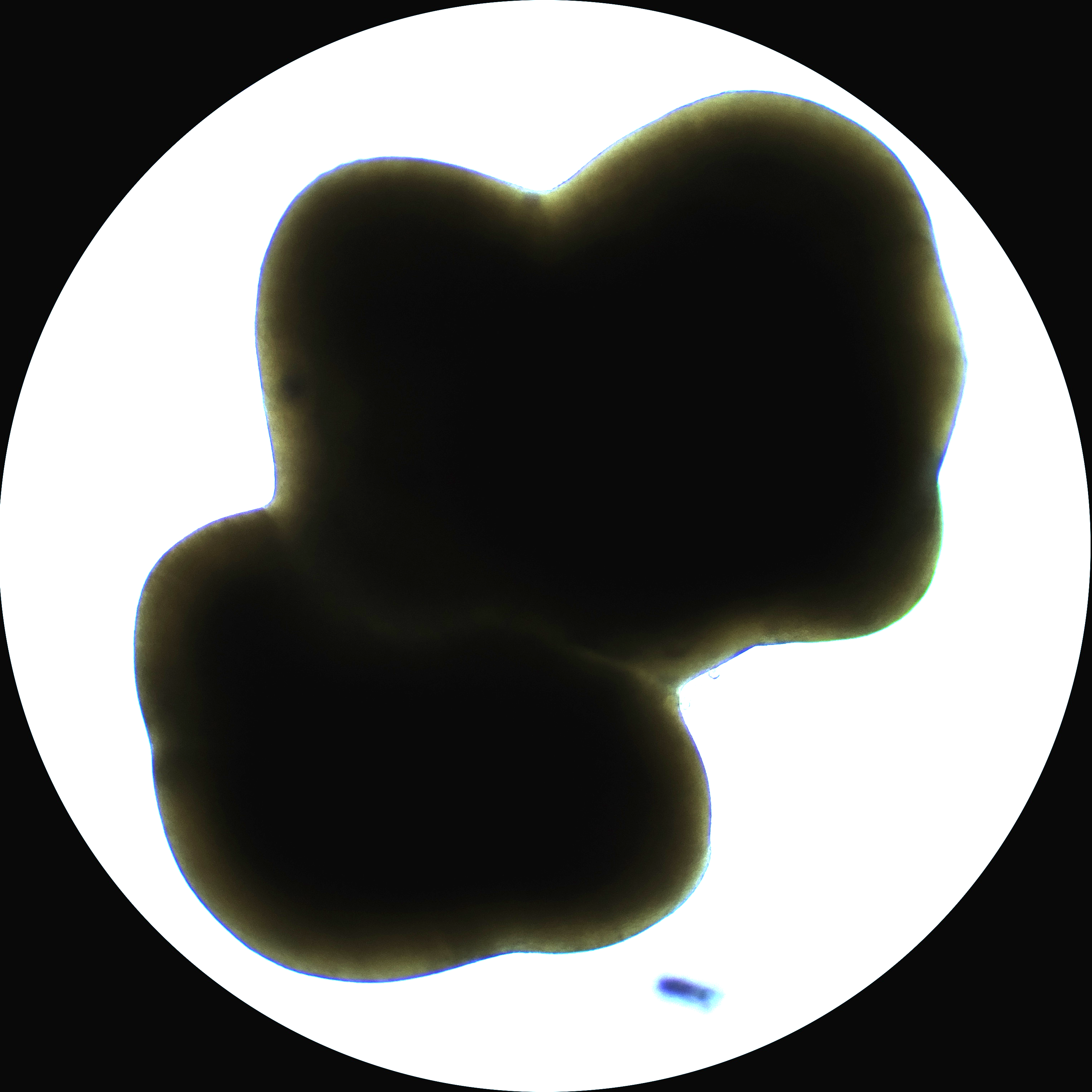

Supplement: Supplementary file 11 — Source data Fig. 3 [file 44319_2025_619_MOESM11_ESM.zip › Figure 3/E/OS/MN_B_12C1_C8_D90_0000 original.tif]

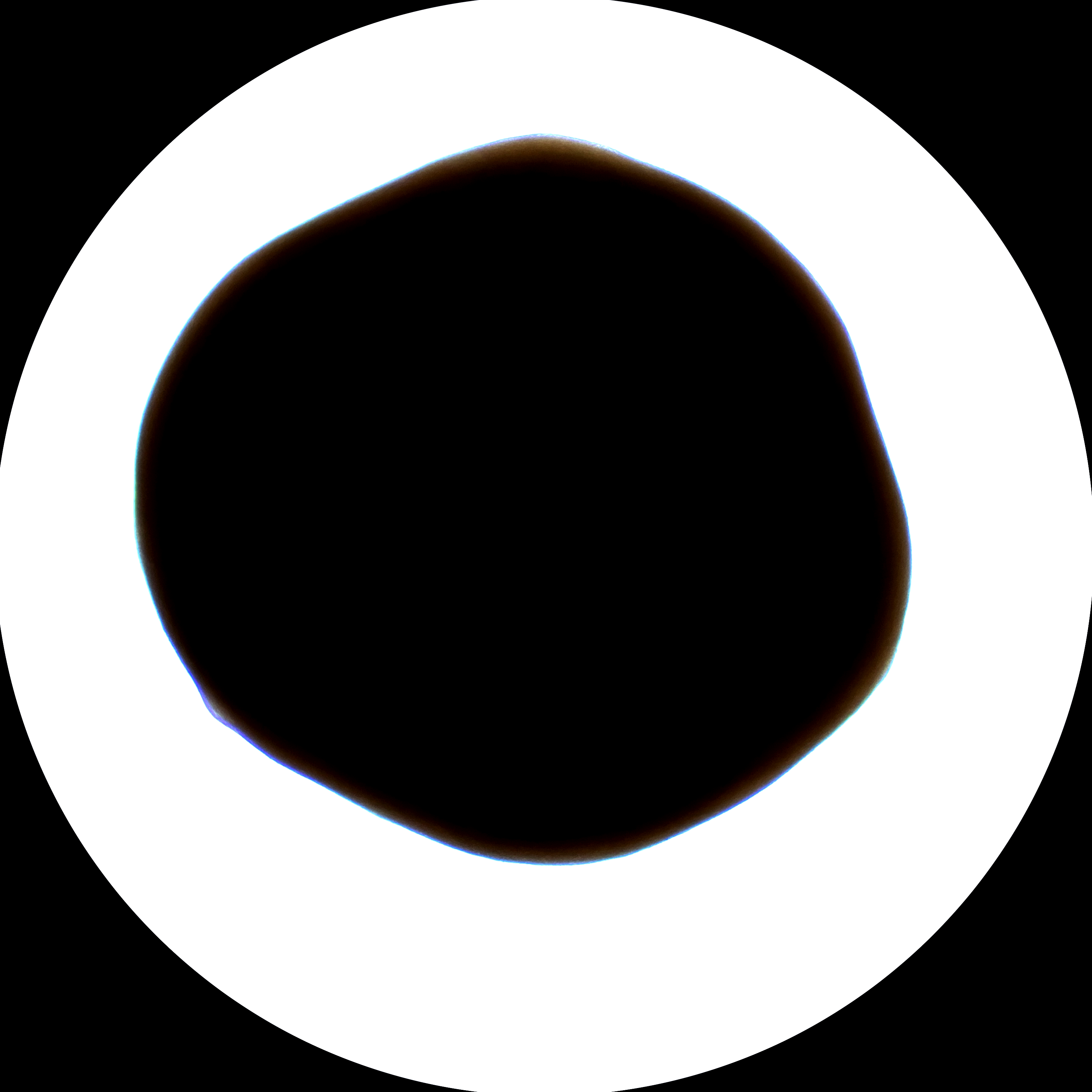

Supplement: Supplementary file 11 — Source data Fig. 3 [file 44319_2025_619_MOESM11_ESM.zip › Figure 3/E/RC/GA_12C1_C61_D90_0003 original.tif]

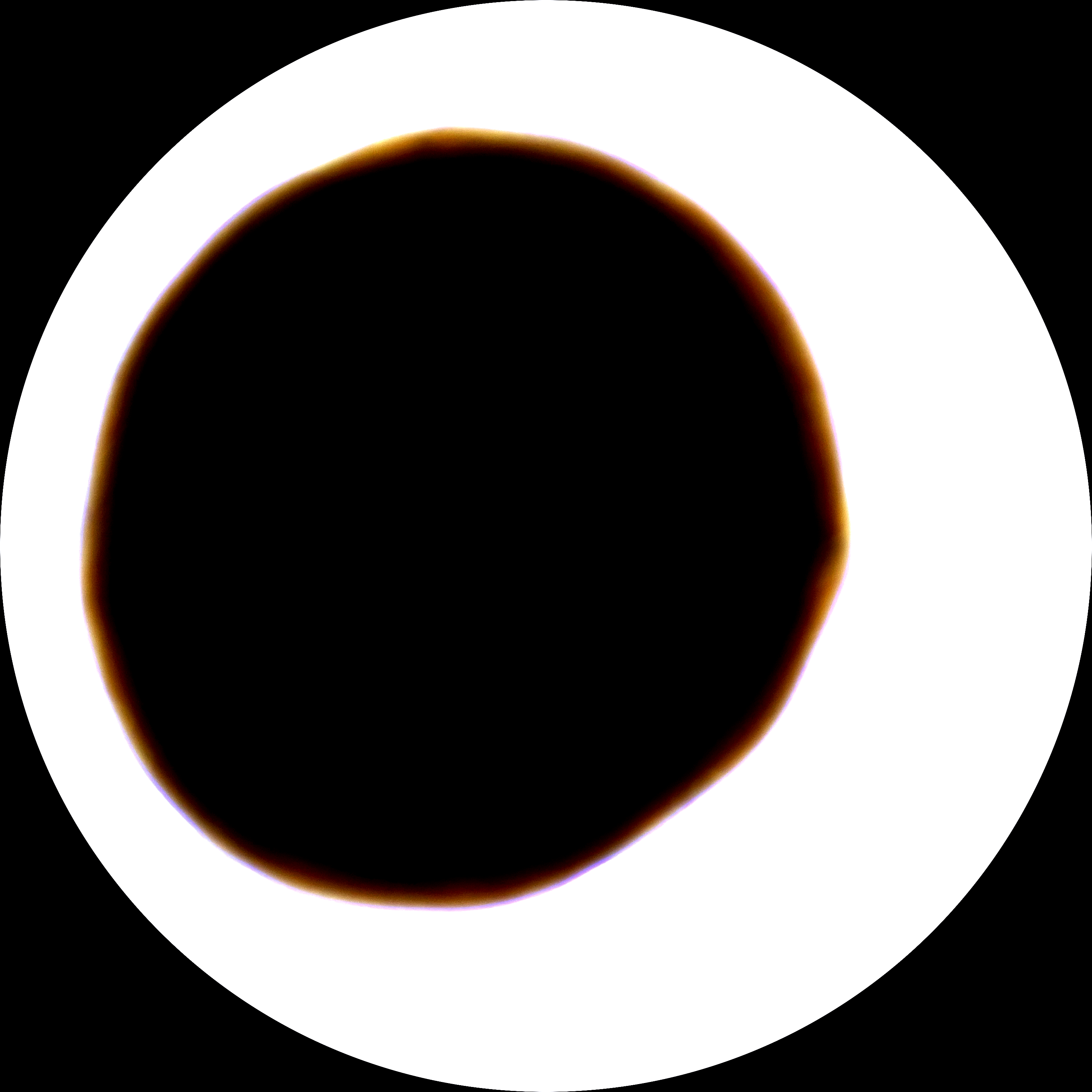

Supplement: Supplementary file 11 — Source data Fig. 3 [file 44319_2025_619_MOESM11_ESM.zip › Figure 3/E/RC/GA_11C1_C71_D90_0007 original.tif]

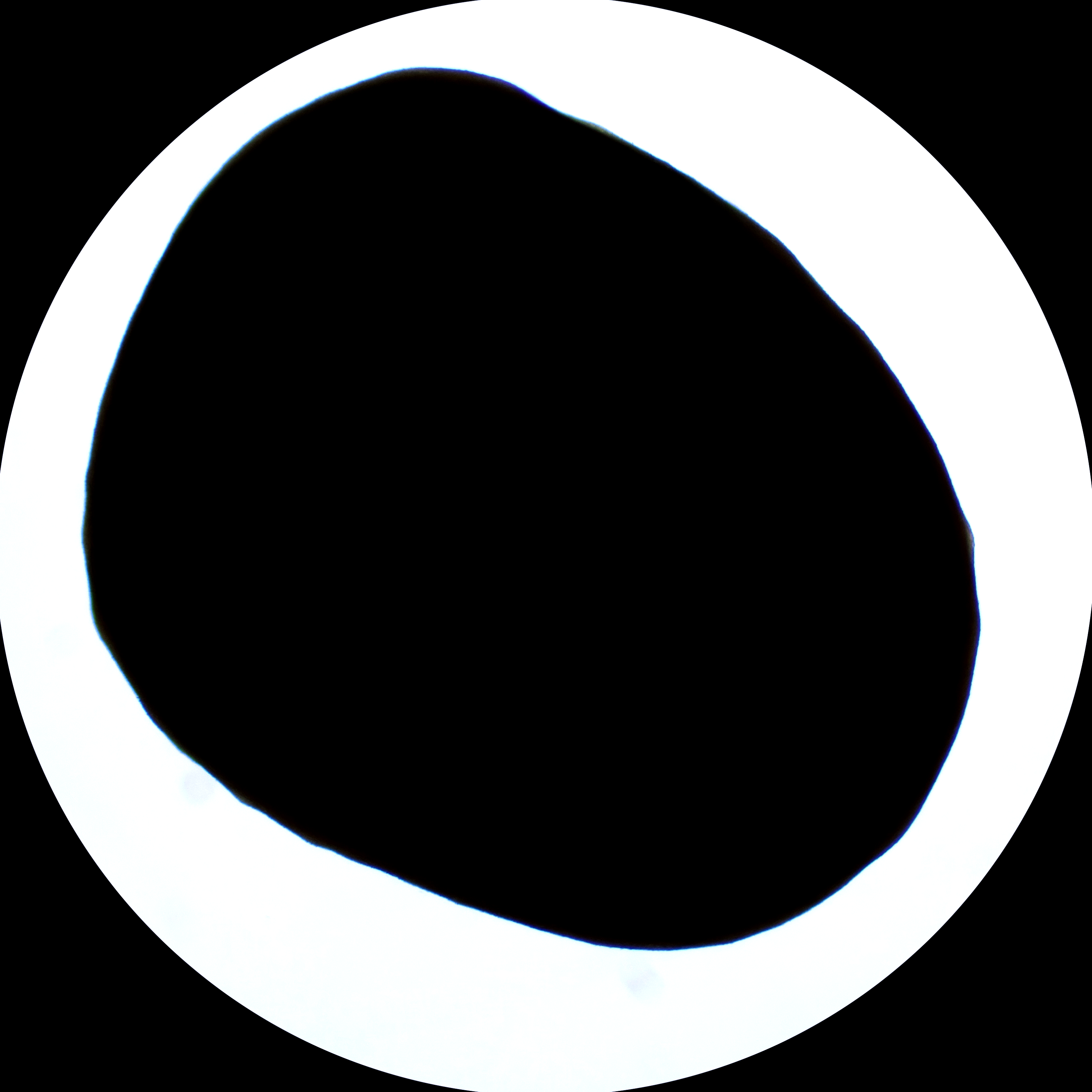

Supplement: Supplementary file 11 — Source data Fig. 3 [file 44319_2025_619_MOESM11_ESM.zip › Figure 3/E/RC/GA_12C1_C52_D90_0003 original.tif]

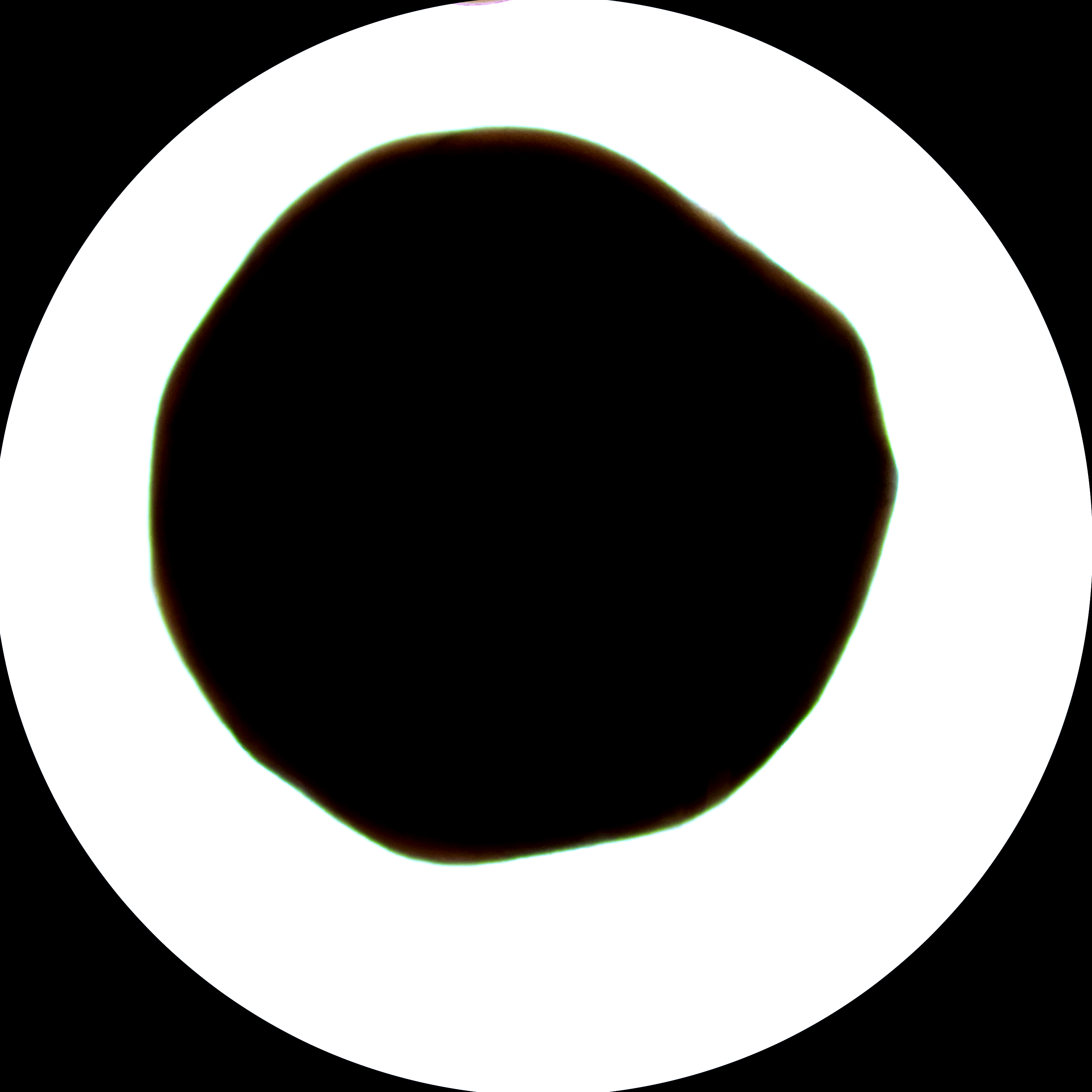

Supplement: Supplementary file 11 — Source data Fig. 3 [file 44319_2025_619_MOESM11_ESM.zip › Figure 3/E/RC/GA_11C1_C63_D90_0002 original.tif]

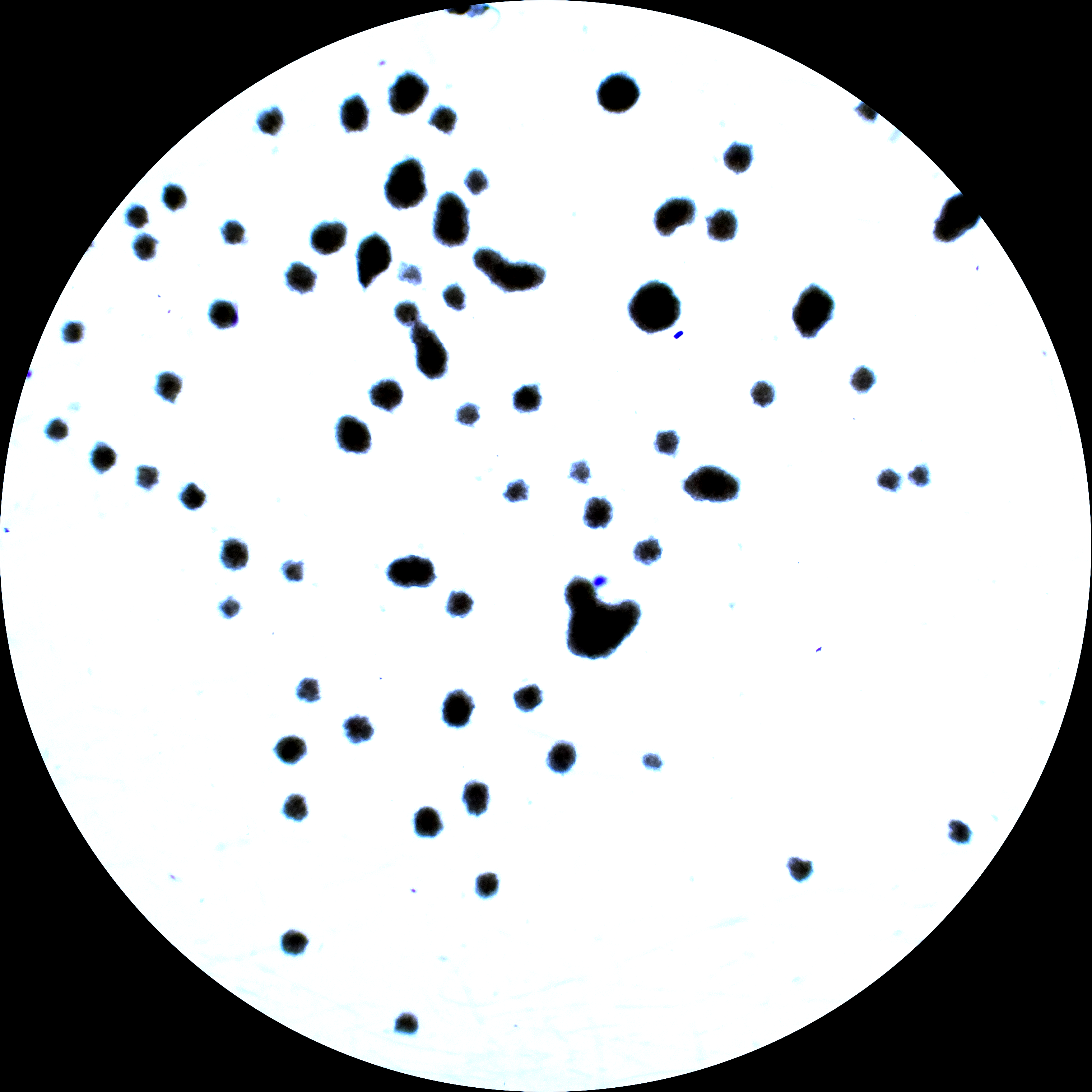

Supplement: Supplementary file 11 — Source data Fig. 3 [file 44319_2025_619_MOESM11_ESM.zip › Figure 3/B/PA/MN_A_12C1_C10_D6_0000 original.tif]

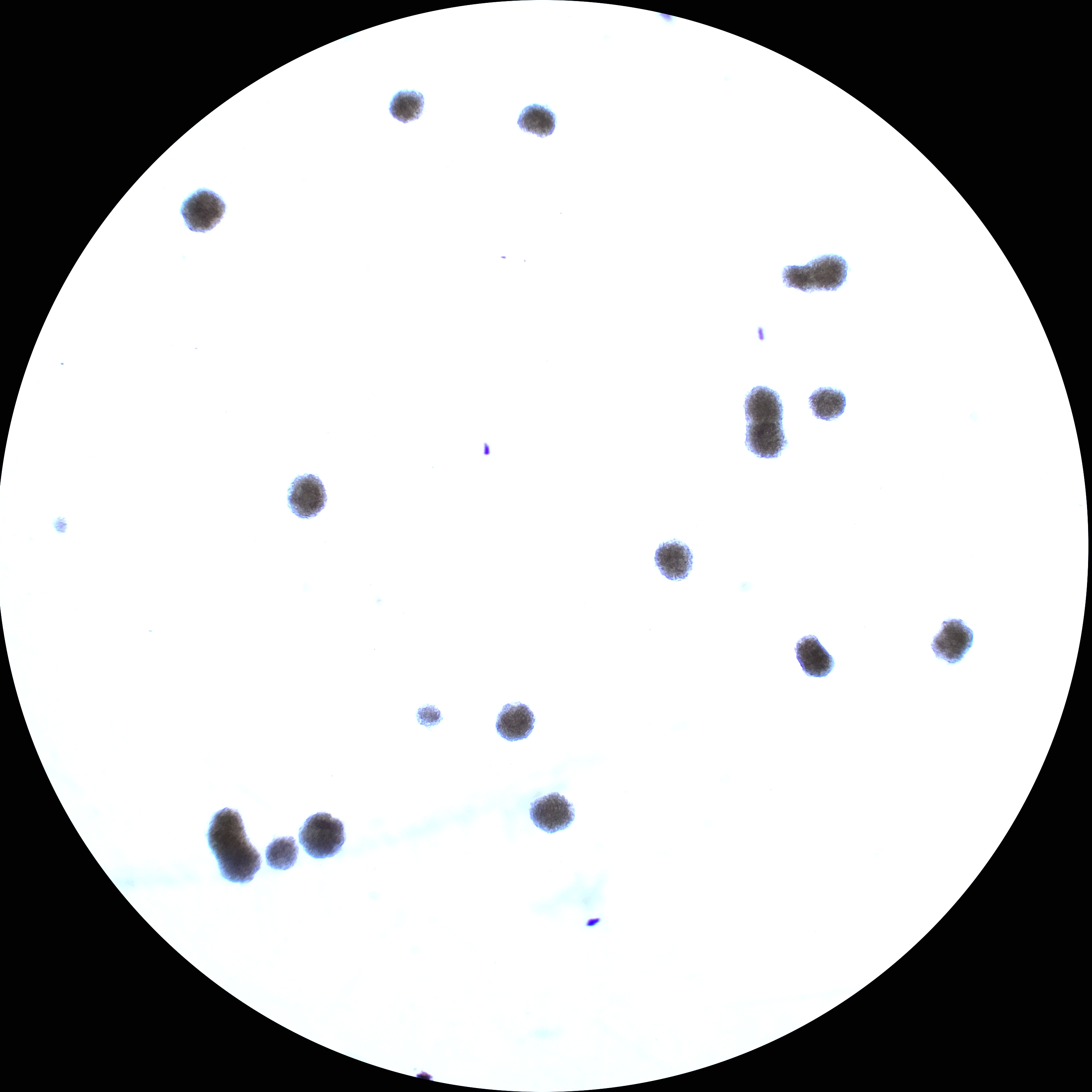

Supplement: Supplementary file 11 — Source data Fig. 3 [file 44319_2025_619_MOESM11_ESM.zip › Figure 3/B/PA/MN_A_11C1_C1_D6_0000 original.tif]

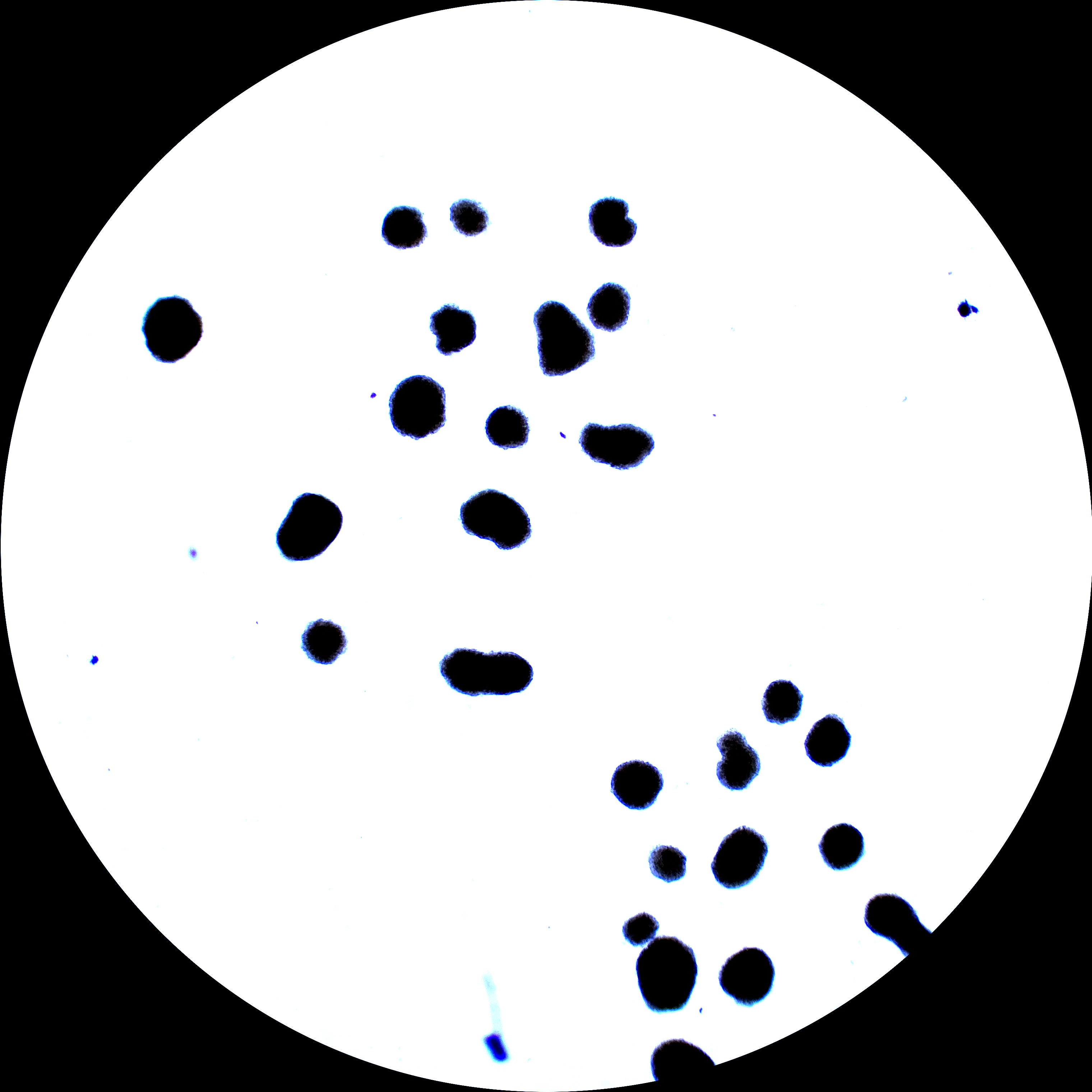

Supplement: Supplementary file 11 — Source data Fig. 3 [file 44319_2025_619_MOESM11_ESM.zip › Figure 3/B/PA/MN_A_12C1_C2_D6_0001 original.tif]

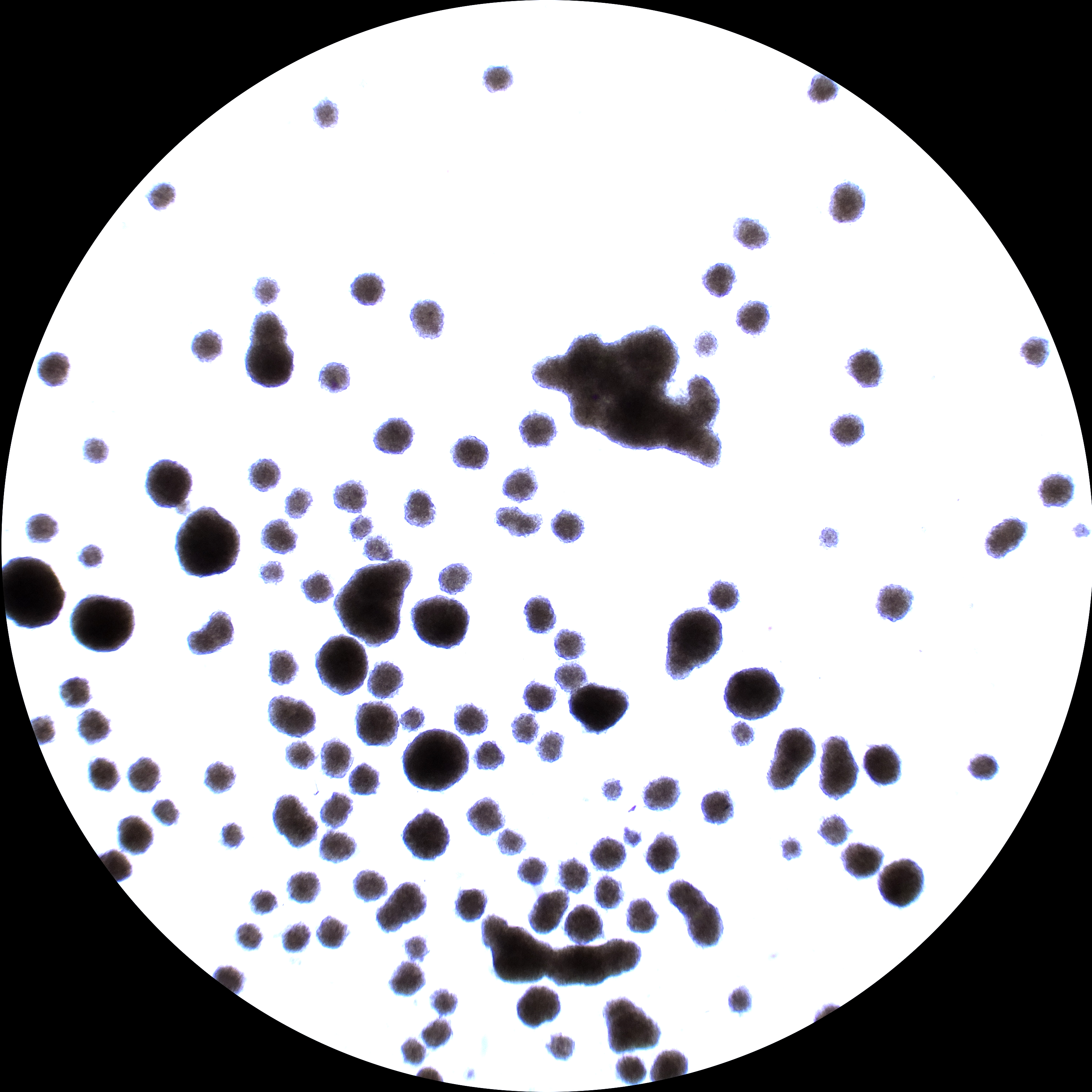

Supplement: Supplementary file 11 — Source data Fig. 3 [file 44319_2025_619_MOESM11_ESM.zip › Figure 3/B/PA/MN_A_11C1_C5_D6_0000 original.tif]

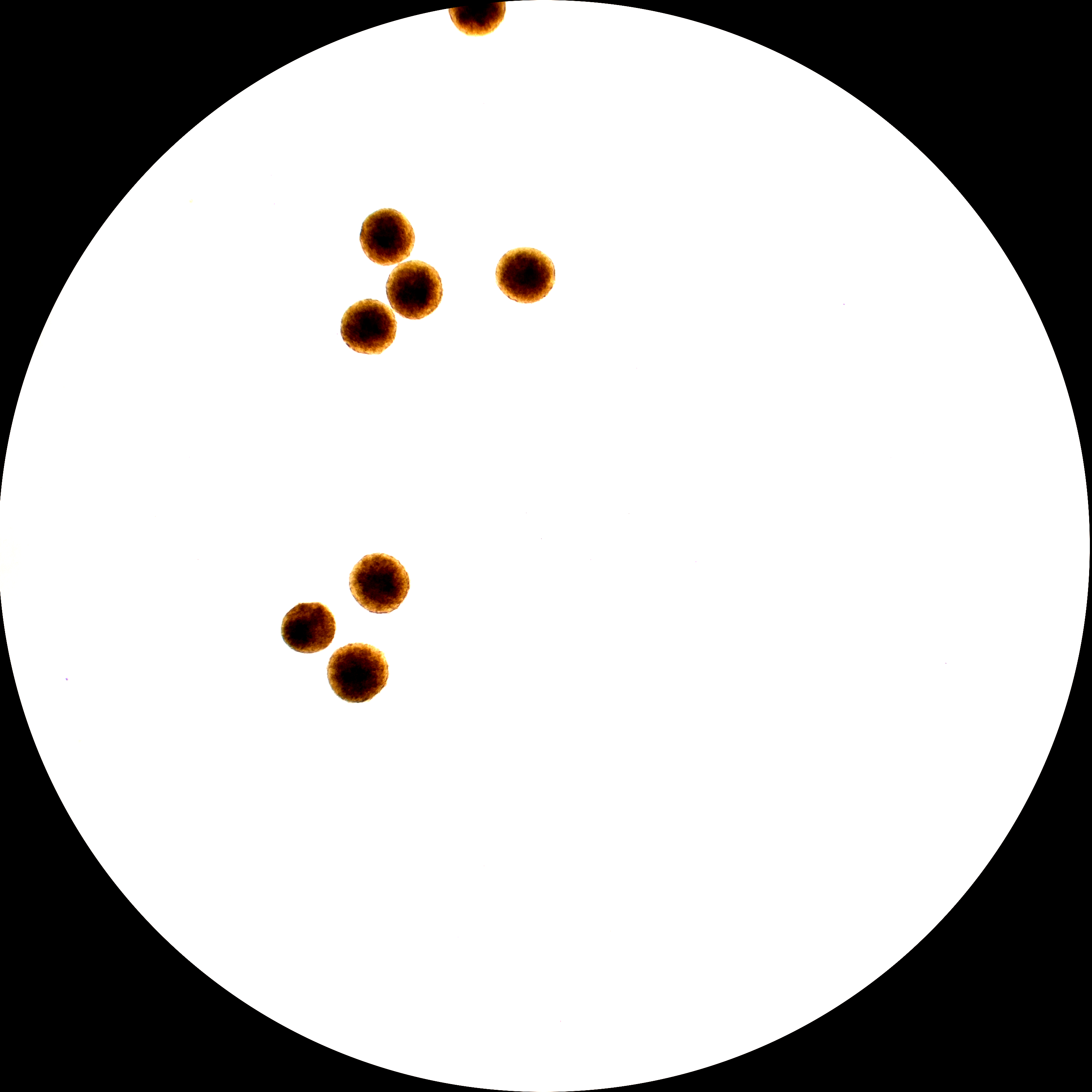

Supplement: Supplementary file 11 — Source data Fig. 3 [file 44319_2025_619_MOESM11_ESM.zip › Figure 3/B/RC/GA_11C1_C71_D6_0002 original.tif]

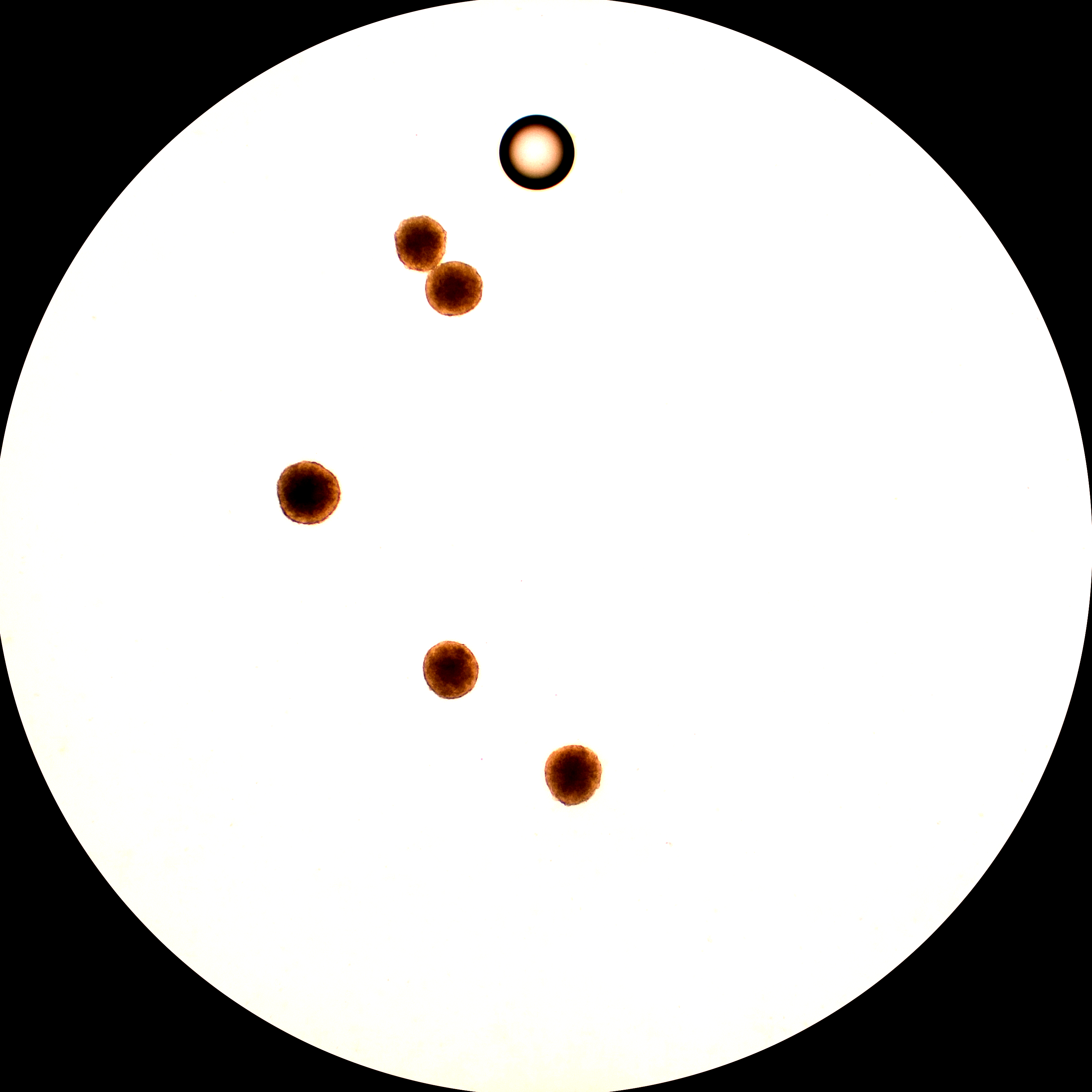

Supplement: Supplementary file 11 — Source data Fig. 3 [file 44319_2025_619_MOESM11_ESM.zip › Figure 3/B/RC/GA_12C1_C52_D6_0011 original.tif]

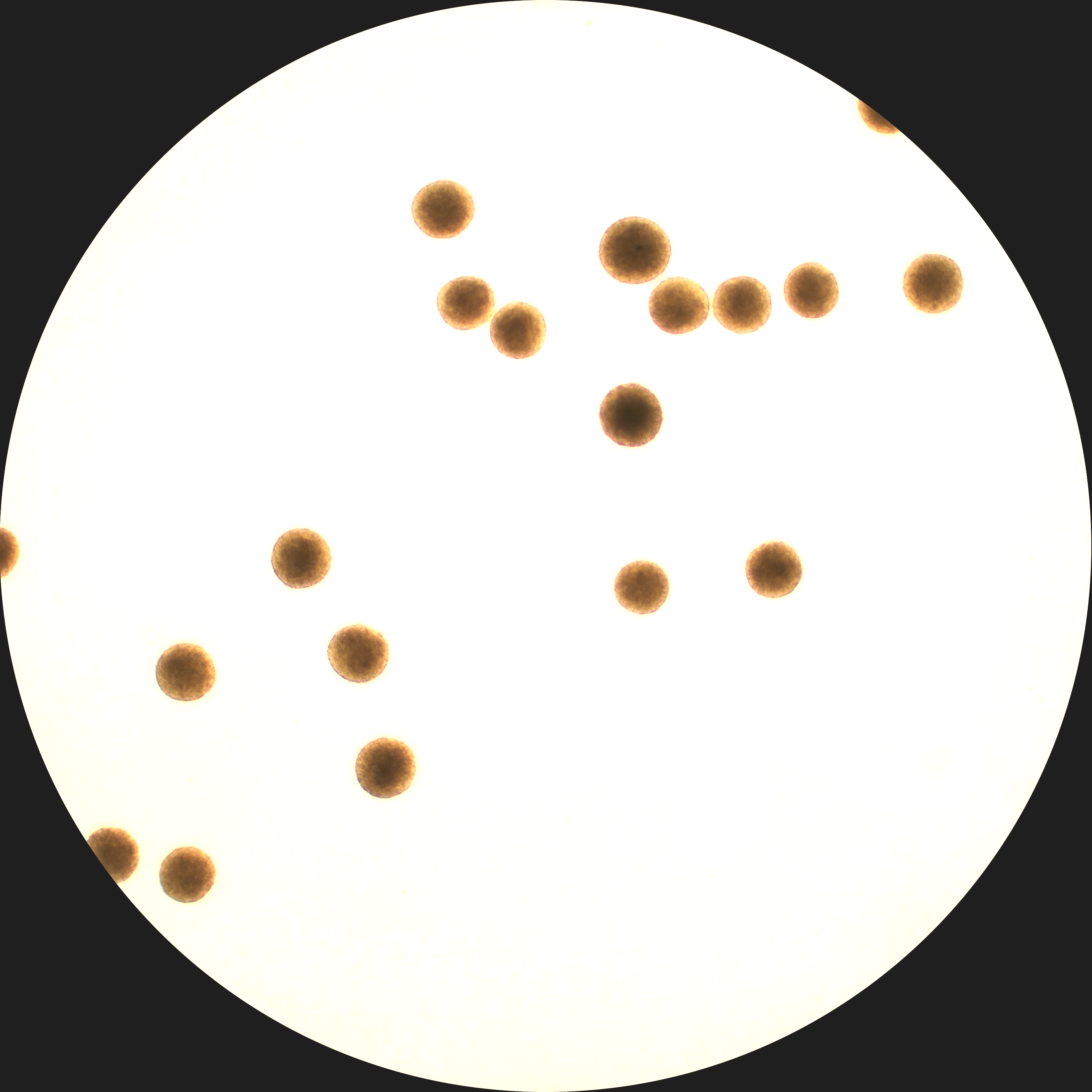

Supplement: Supplementary file 11 — Source data Fig. 3 [file 44319_2025_619_MOESM11_ESM.zip › Figure 3/B/RC/GA_12C1_C69_D6_0004 original.tif]

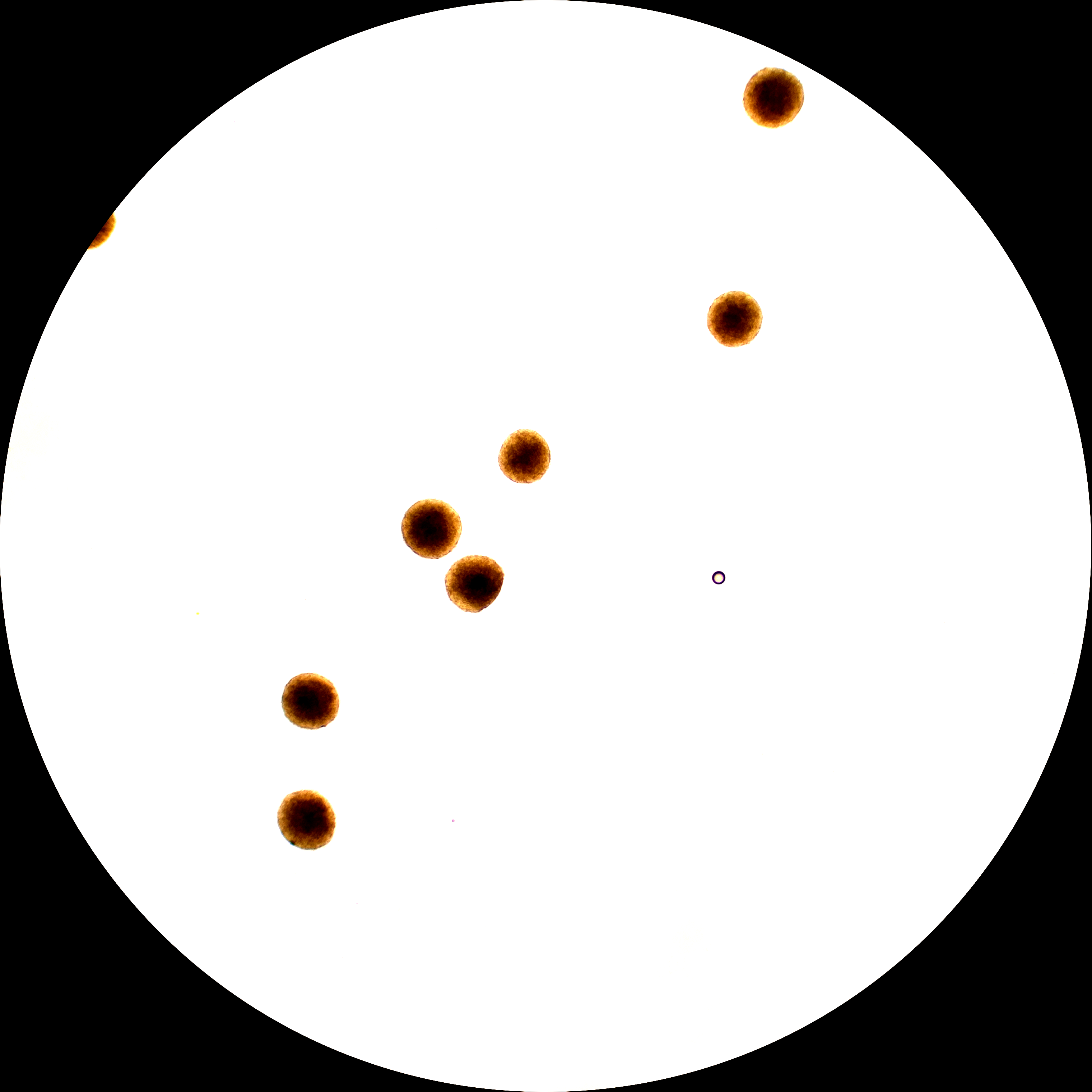

Supplement: Supplementary file 11 — Source data Fig. 3 [file 44319_2025_619_MOESM11_ESM.zip › Figure 3/B/RC/GA_11C1_C71_D6_0000 original.tif]

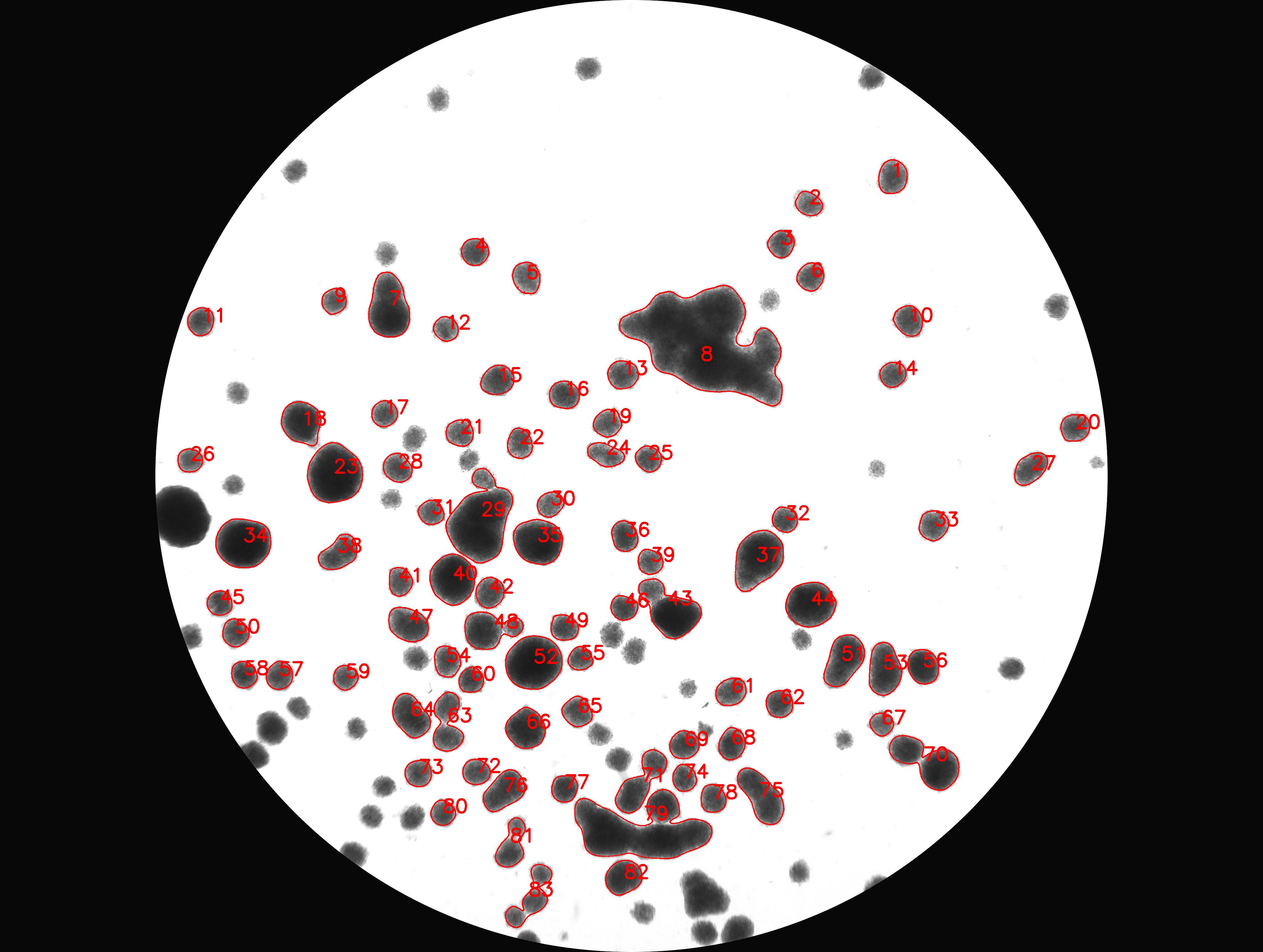

Supplement: Supplementary file 11 — Source data Fig. 3 [file 44319_2025_619_MOESM11_ESM.zip › Figure 3/C,D,F,G/Raw images_mask/PA_day6/MN 11C1 A C5 D06 2x/R_Day6_0000.jpg]

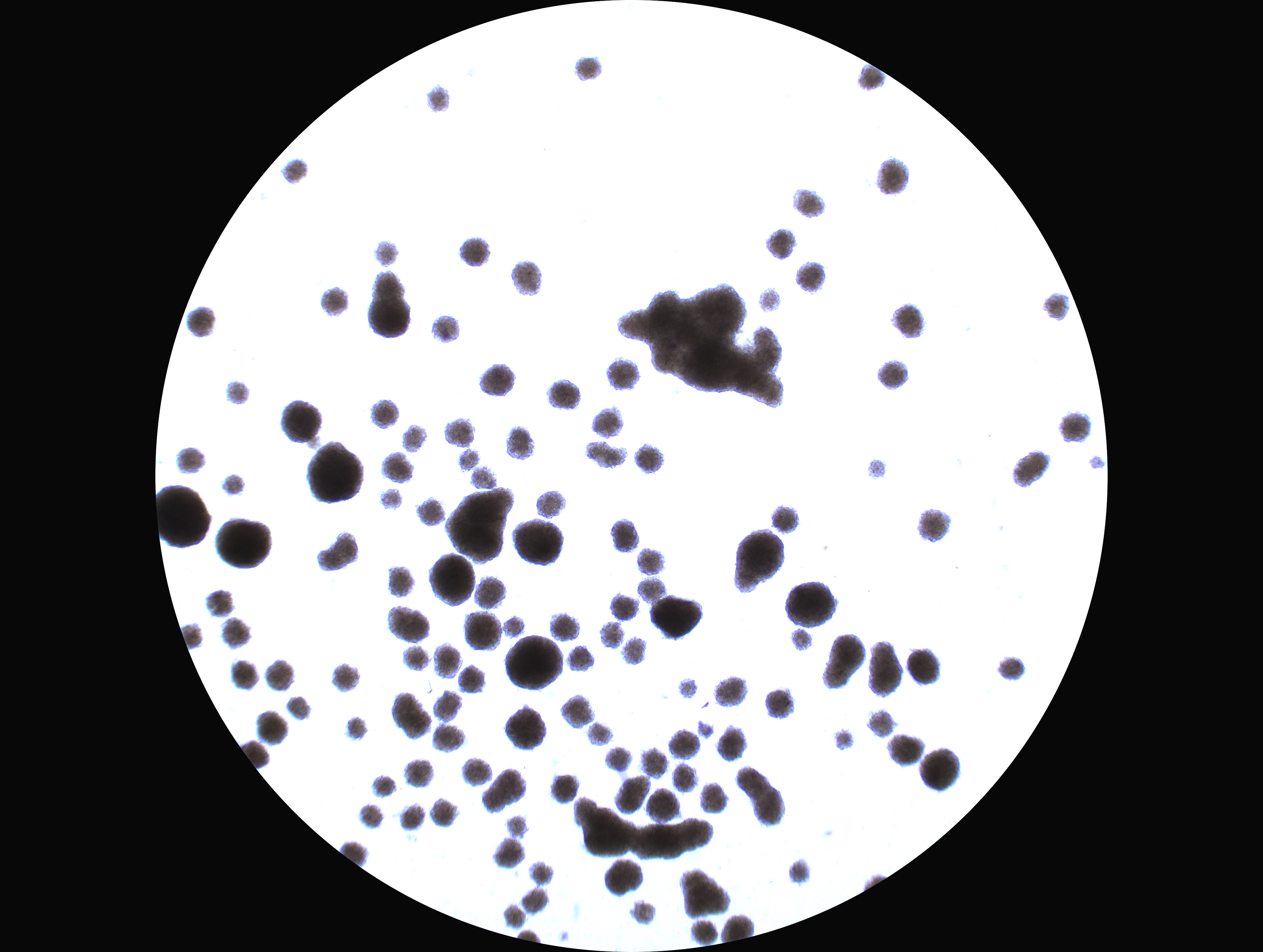

Supplement: Supplementary file 11 — Source data Fig. 3 [file 44319_2025_619_MOESM11_ESM.zip › Figure 3/C,D,F,G/Raw images_mask/PA_day6/MN 11C1 A C5 D06 2x/Day6_0000.jpg]

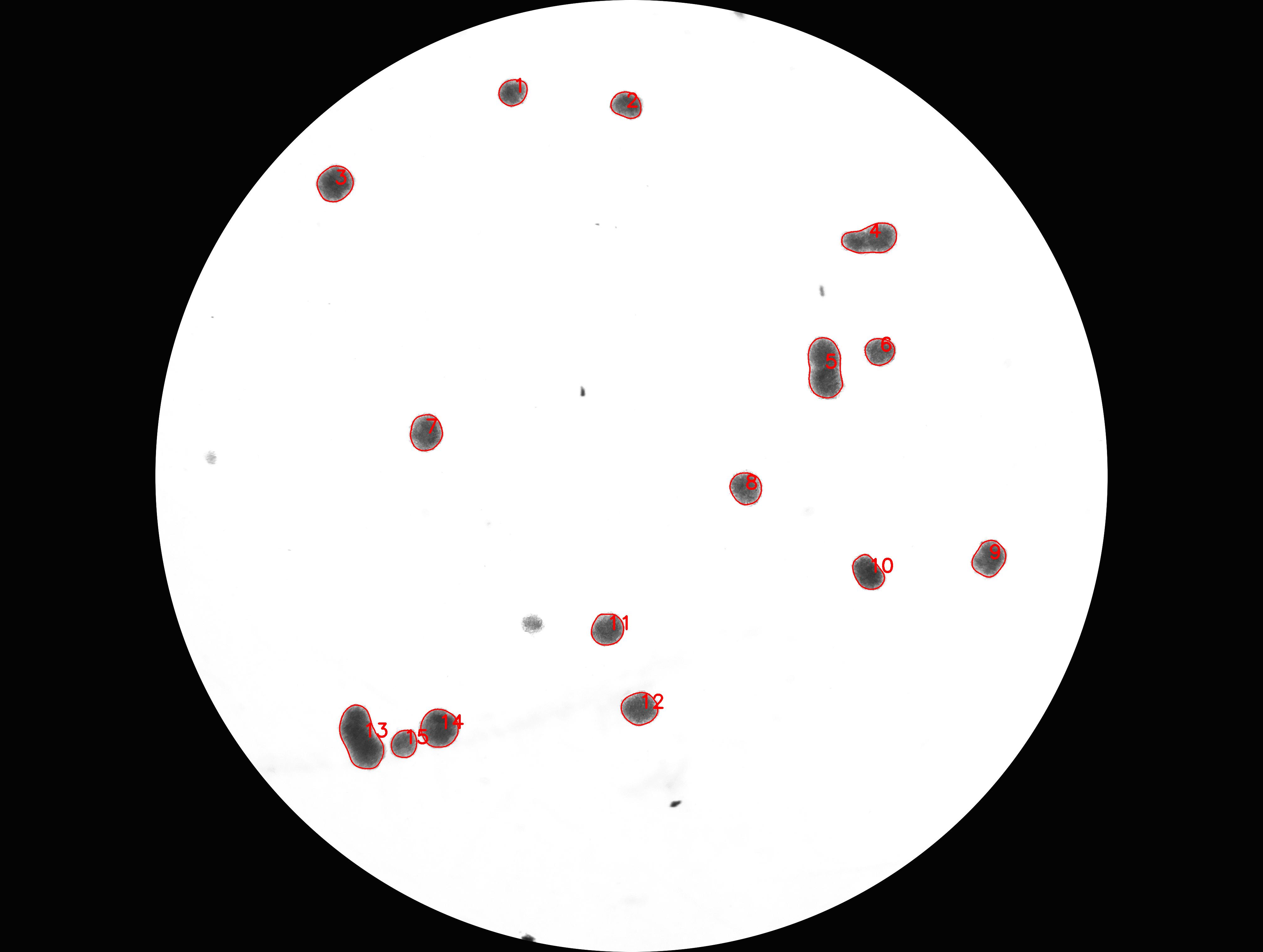

Supplement: Supplementary file 11 — Source data Fig. 3 [file 44319_2025_619_MOESM11_ESM.zip › Figure 3/C,D,F,G/Raw images_mask/PA_day6/MN 11C1 A C1 D06 2x/R_11C1_d6A_0000.jpg]

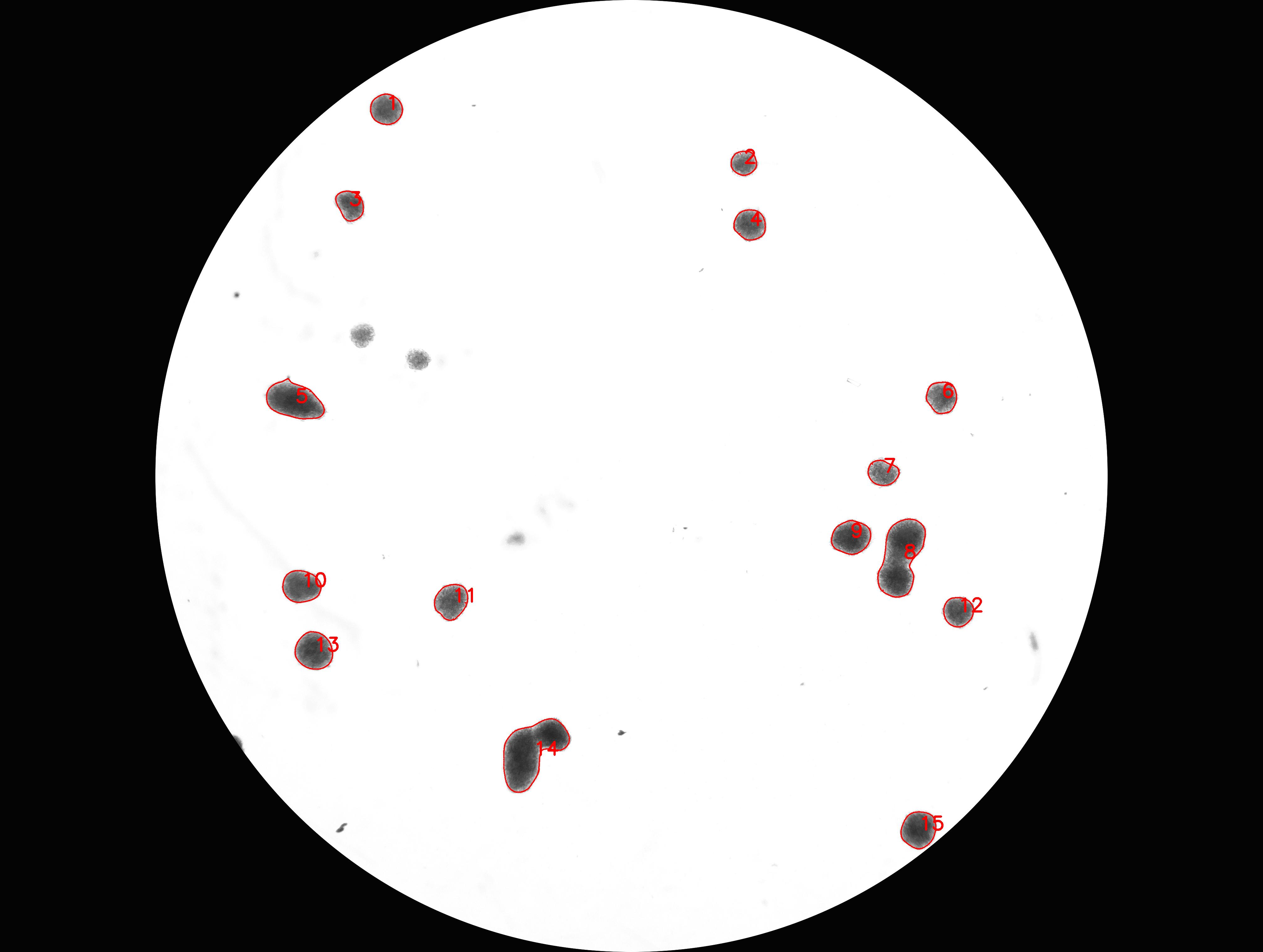

Supplement: Supplementary file 11 — Source data Fig. 3 [file 44319_2025_619_MOESM11_ESM.zip › Figure 3/C,D,F,G/Raw images_mask/PA_day6/MN 11C1 A C1 D06 2x/R_11C1_d6A_0001.jpg]

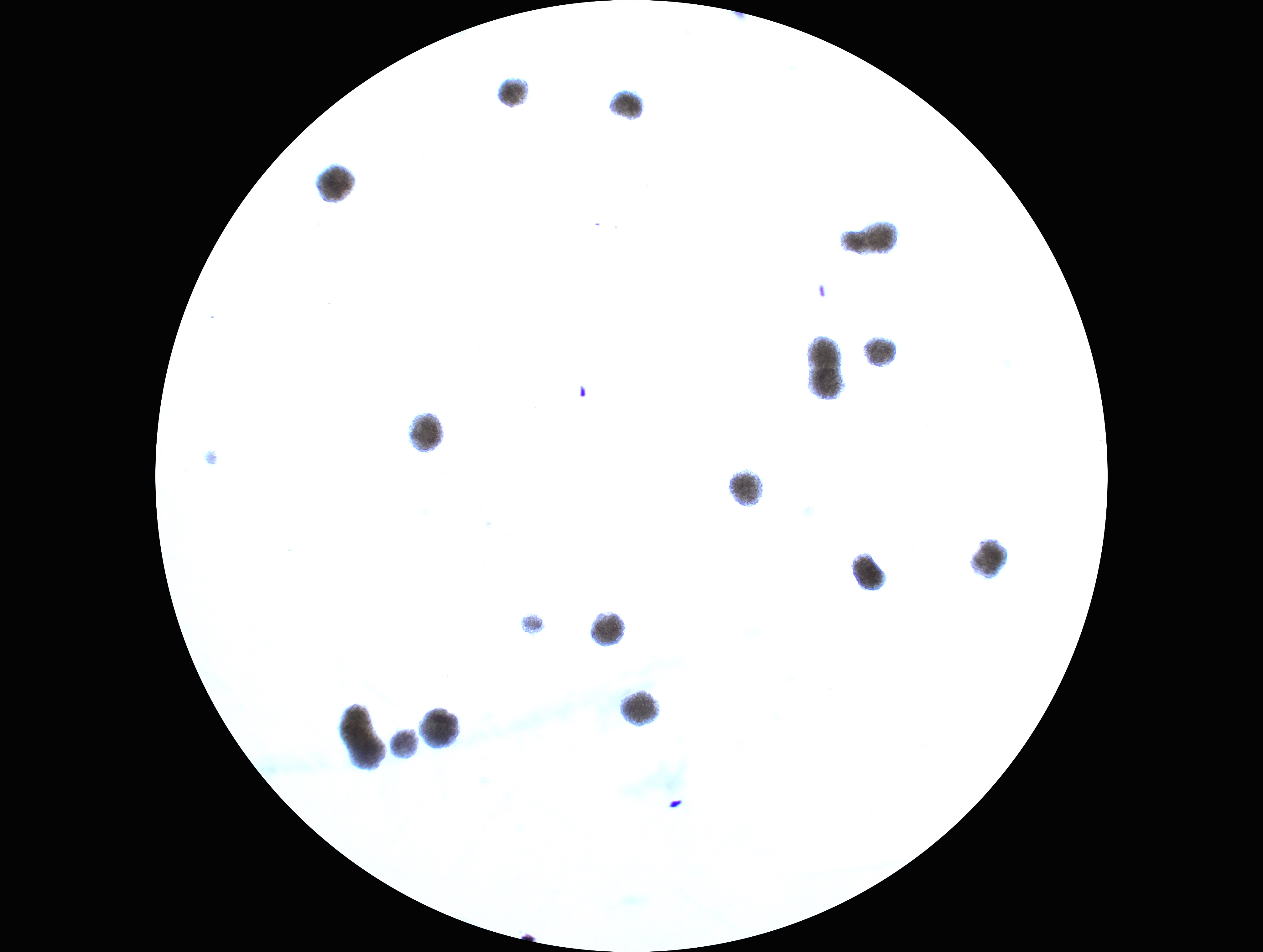

Supplement: Supplementary file 11 — Source data Fig. 3 [file 44319_2025_619_MOESM11_ESM.zip › Figure 3/C,D,F,G/Raw images_mask/PA_day6/MN 11C1 A C1 D06 2x/11C1_d6A_0000.jpg]

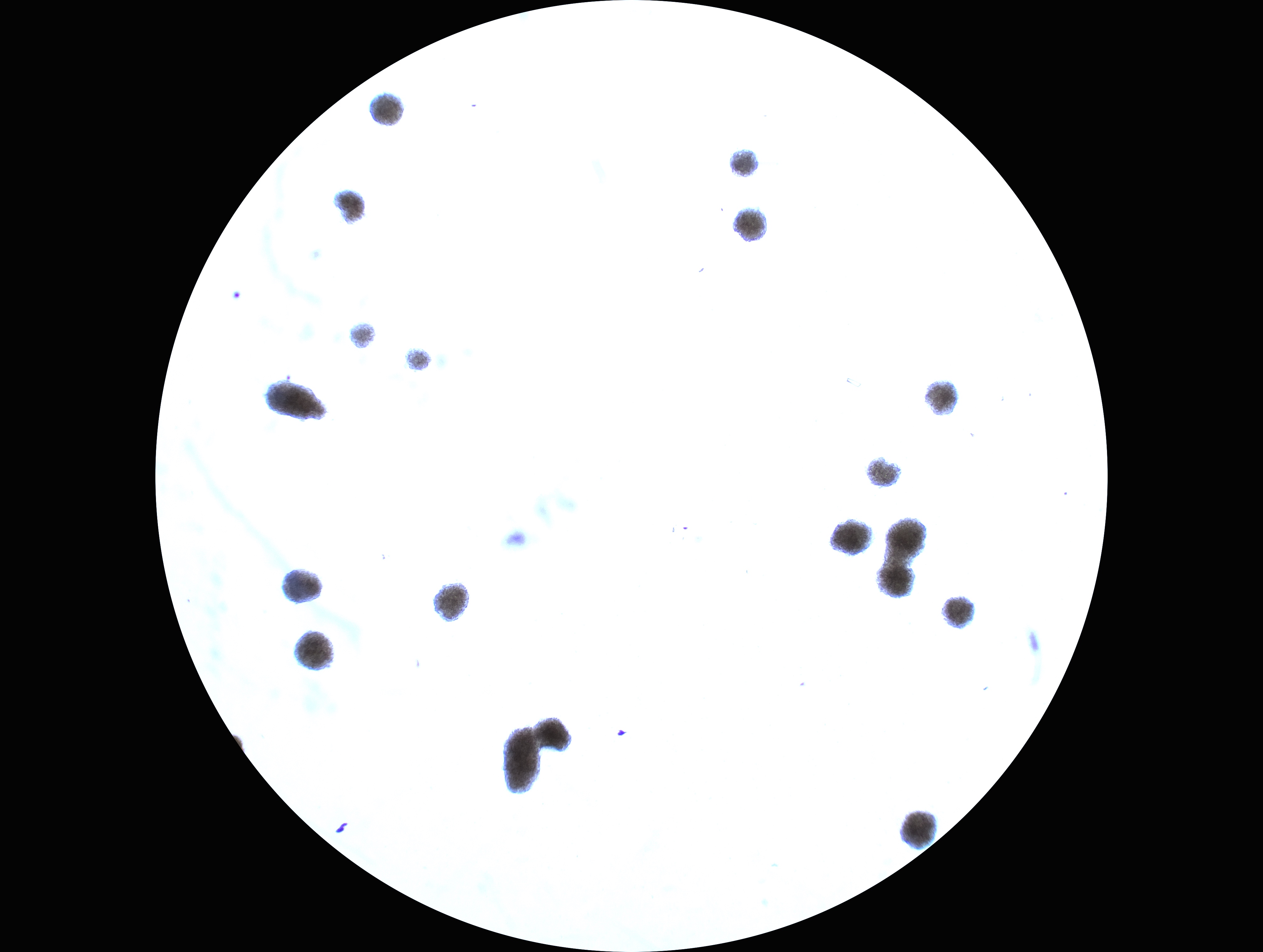

Supplement: Supplementary file 11 — Source data Fig. 3 [file 44319_2025_619_MOESM11_ESM.zip › Figure 3/C,D,F,G/Raw images_mask/PA_day6/MN 11C1 A C1 D06 2x/11C1_d6A_0001.jpg]

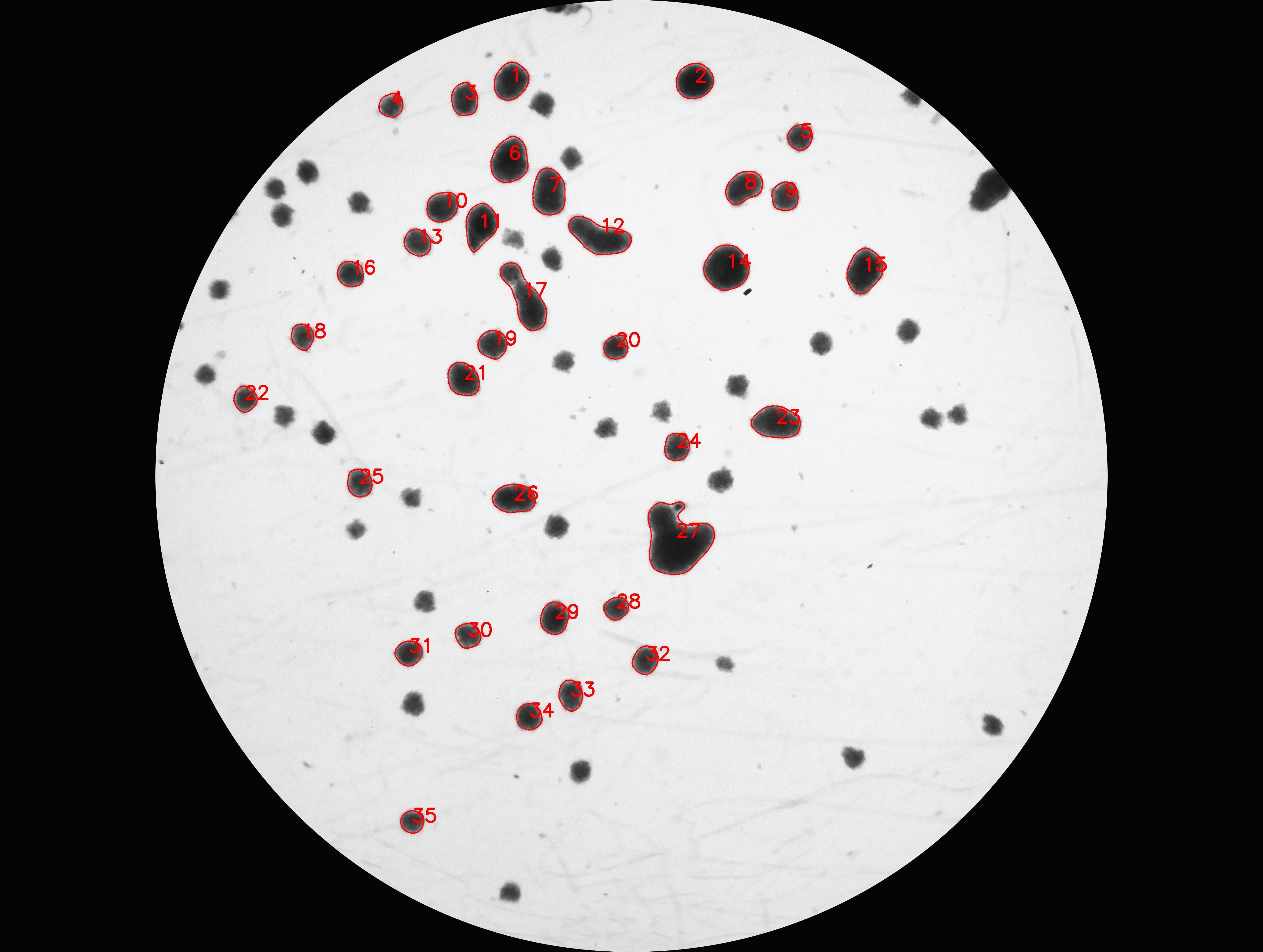

Supplement: Supplementary file 11 — Source data Fig. 3 [file 44319_2025_619_MOESM11_ESM.zip › Figure 3/C,D,F,G/Raw images_mask/PA_day6/MN 12C1 A C10 D06 2x/R_day 6_0000.jpg]

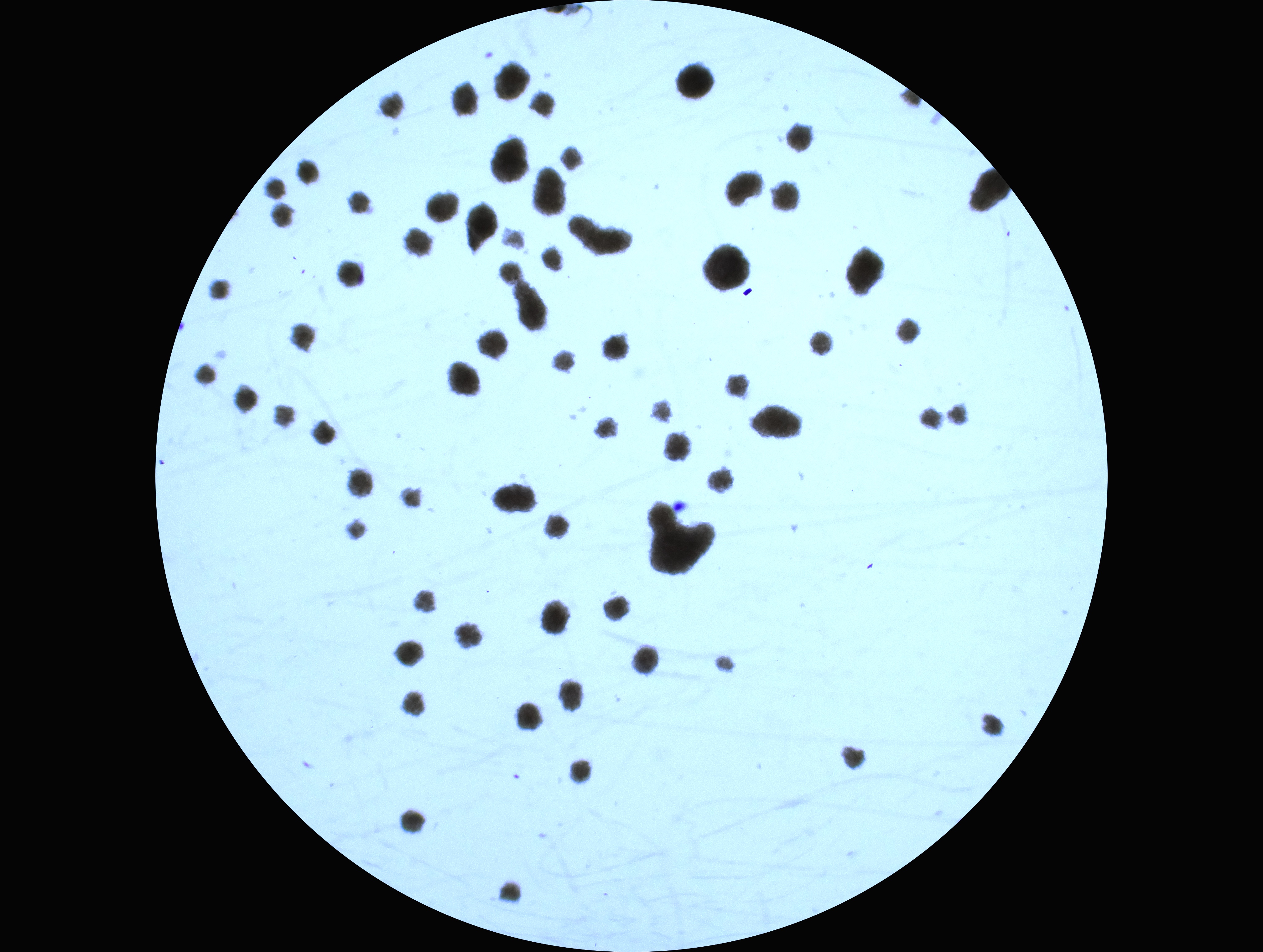

Supplement: Supplementary file 11 — Source data Fig. 3 [file 44319_2025_619_MOESM11_ESM.zip › Figure 3/C,D,F,G/Raw images_mask/PA_day6/MN 12C1 A C10 D06 2x/day 6_0000.jpg]

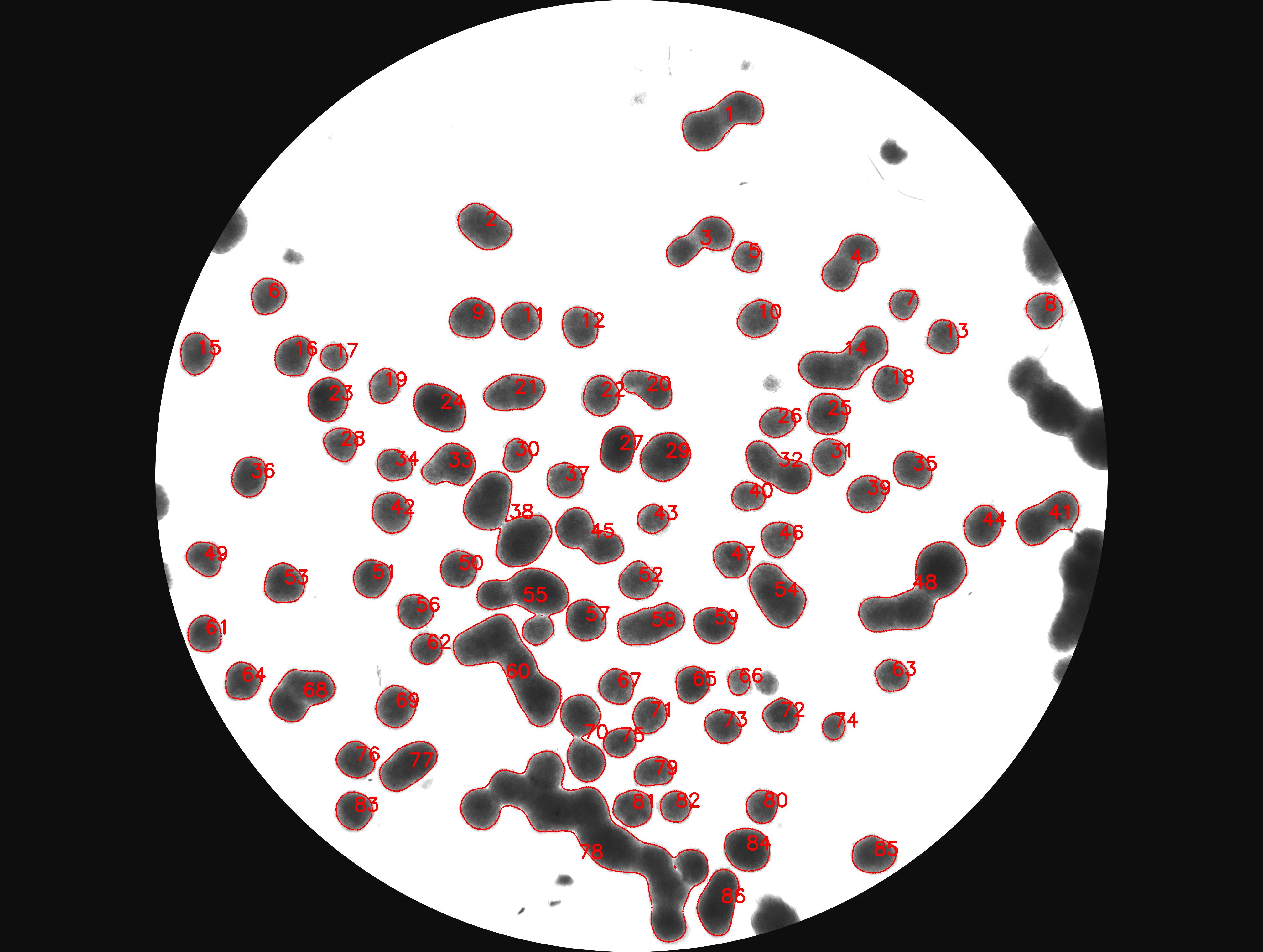

Supplement: Supplementary file 11 — Source data Fig. 3 [file 44319_2025_619_MOESM11_ESM.zip › Figure 3/C,D,F,G/Raw images_mask/PA_day6/MN 12C1 A C6 D06 2x/R_Day6_0001.jpg]

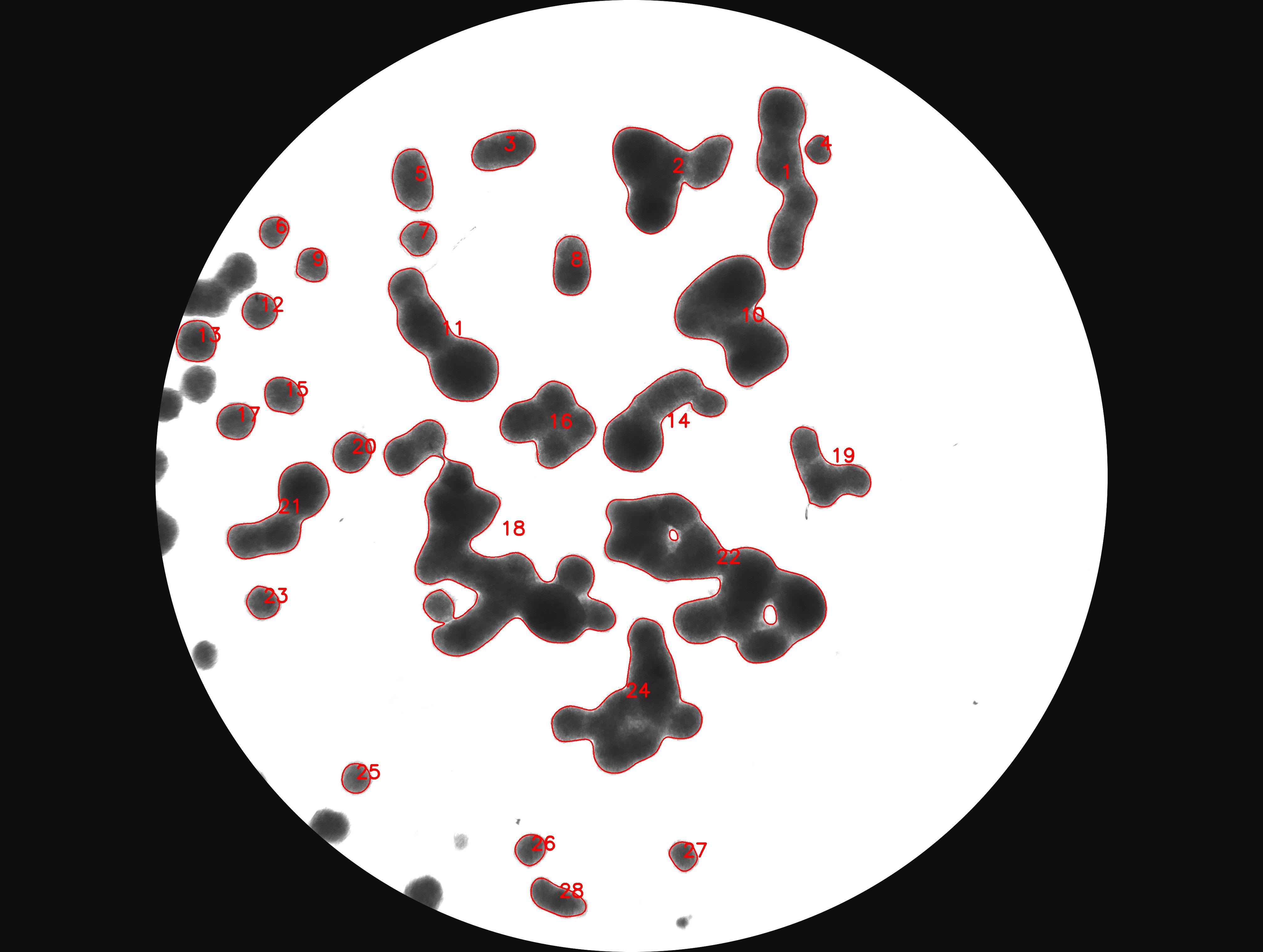

Supplement: Supplementary file 11 — Source data Fig. 3 [file 44319_2025_619_MOESM11_ESM.zip › Figure 3/C,D,F,G/Raw images_mask/PA_day6/MN 12C1 A C6 D06 2x/R_Day6_0000.jpg]

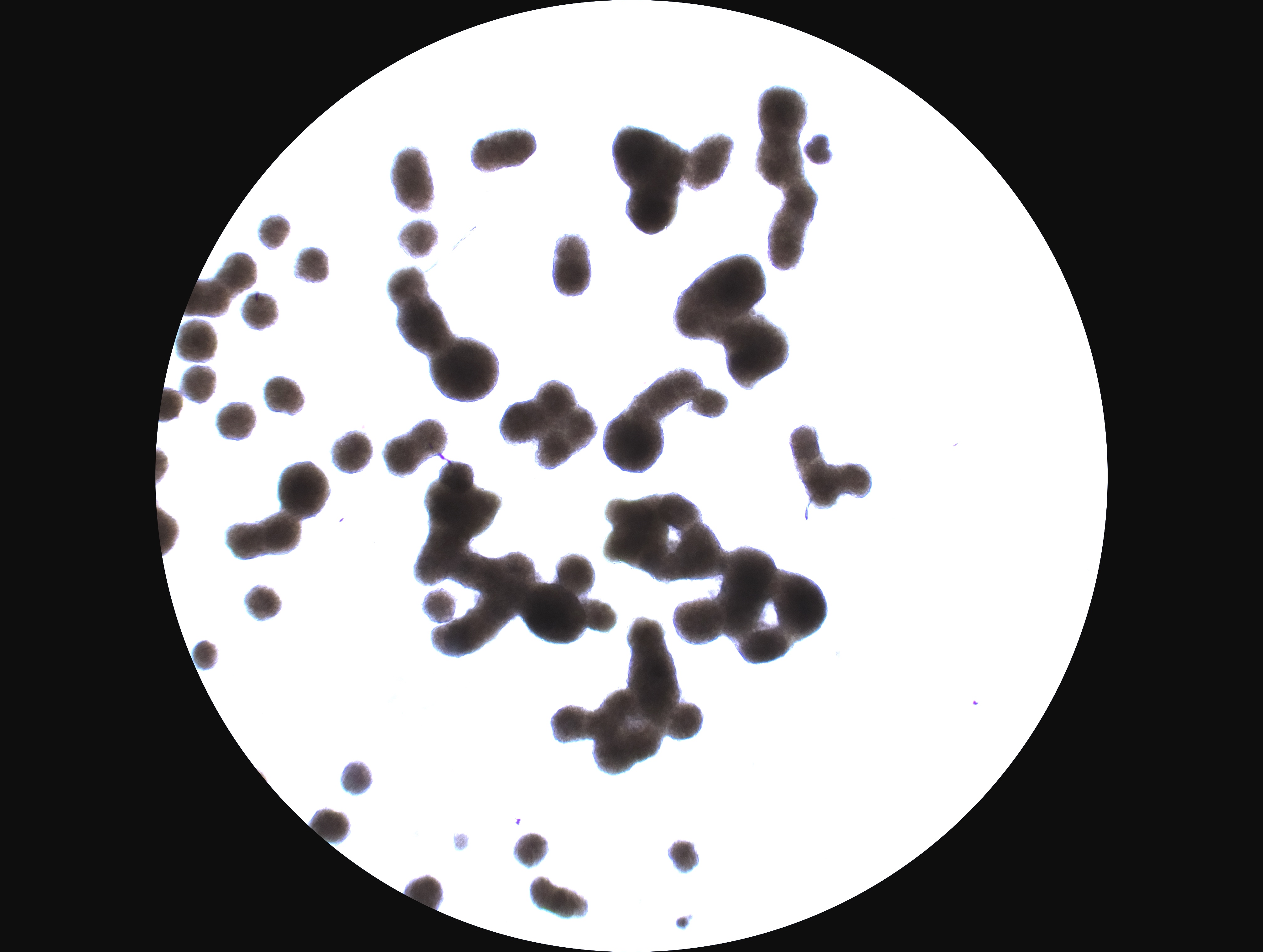

Supplement: Supplementary file 11 — Source data Fig. 3 [file 44319_2025_619_MOESM11_ESM.zip › Figure 3/C,D,F,G/Raw images_mask/PA_day6/MN 12C1 A C6 D06 2x/Day6_0000.jpg]

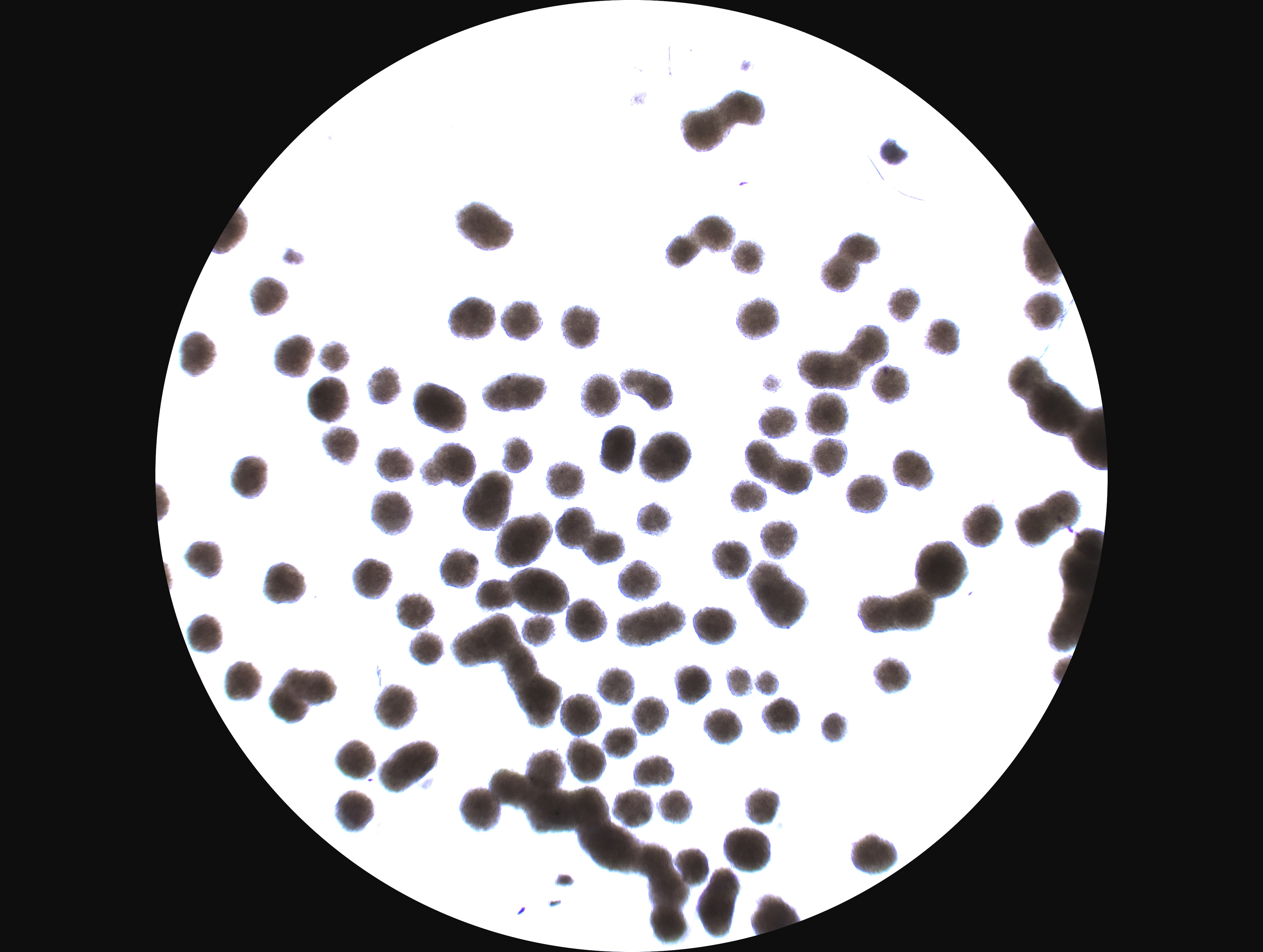

Supplement: Supplementary file 11 — Source data Fig. 3 [file 44319_2025_619_MOESM11_ESM.zip › Figure 3/C,D,F,G/Raw images_mask/PA_day6/MN 12C1 A C6 D06 2x/Day6_0001.jpg]

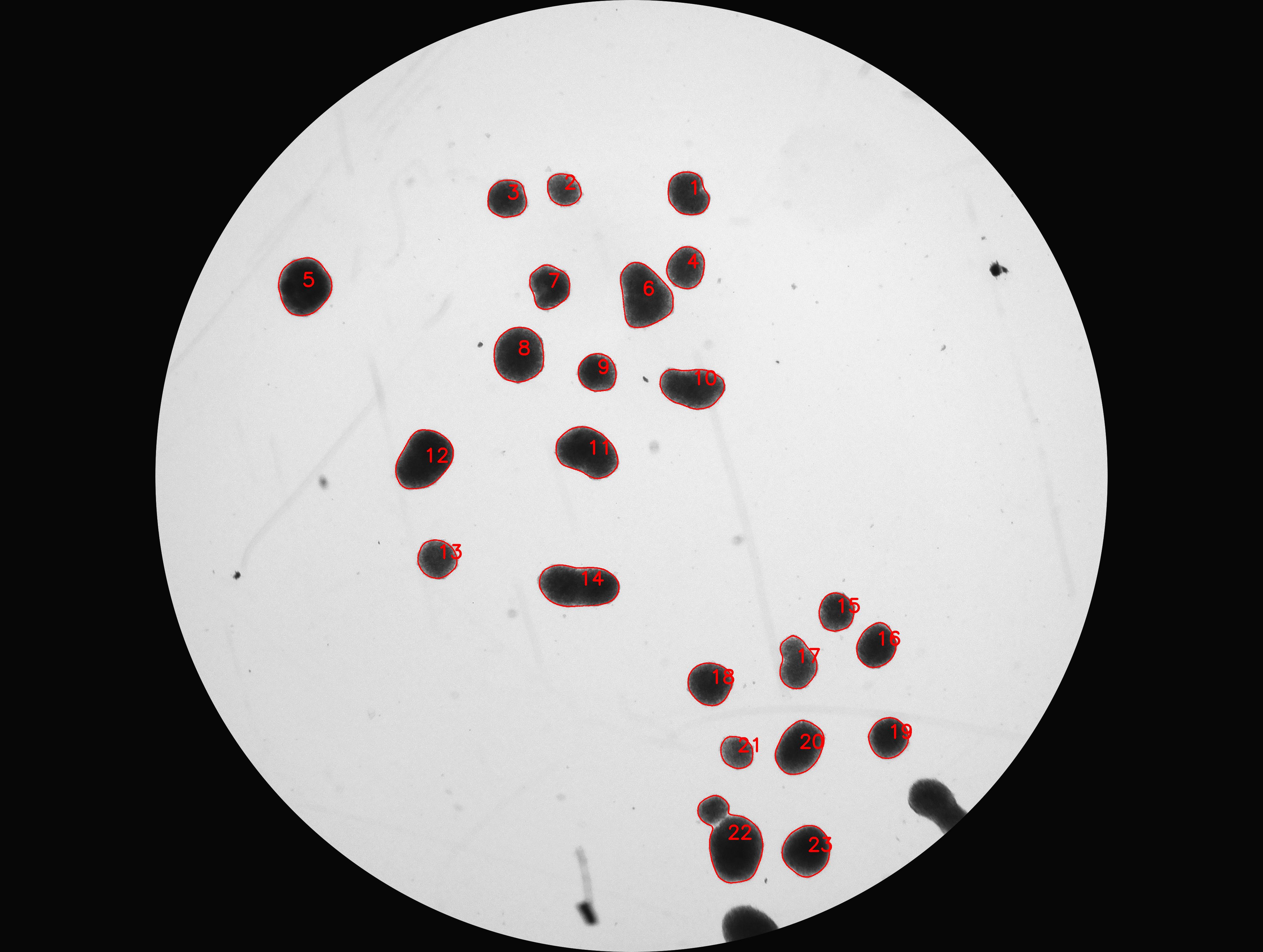

Supplement: Supplementary file 11 — Source data Fig. 3 [file 44319_2025_619_MOESM11_ESM.zip › Figure 3/C,D,F,G/Raw images_mask/PA_day6/MN 12C1 A C2 D06 2x/R_12C1_d6A_0001.jpg]

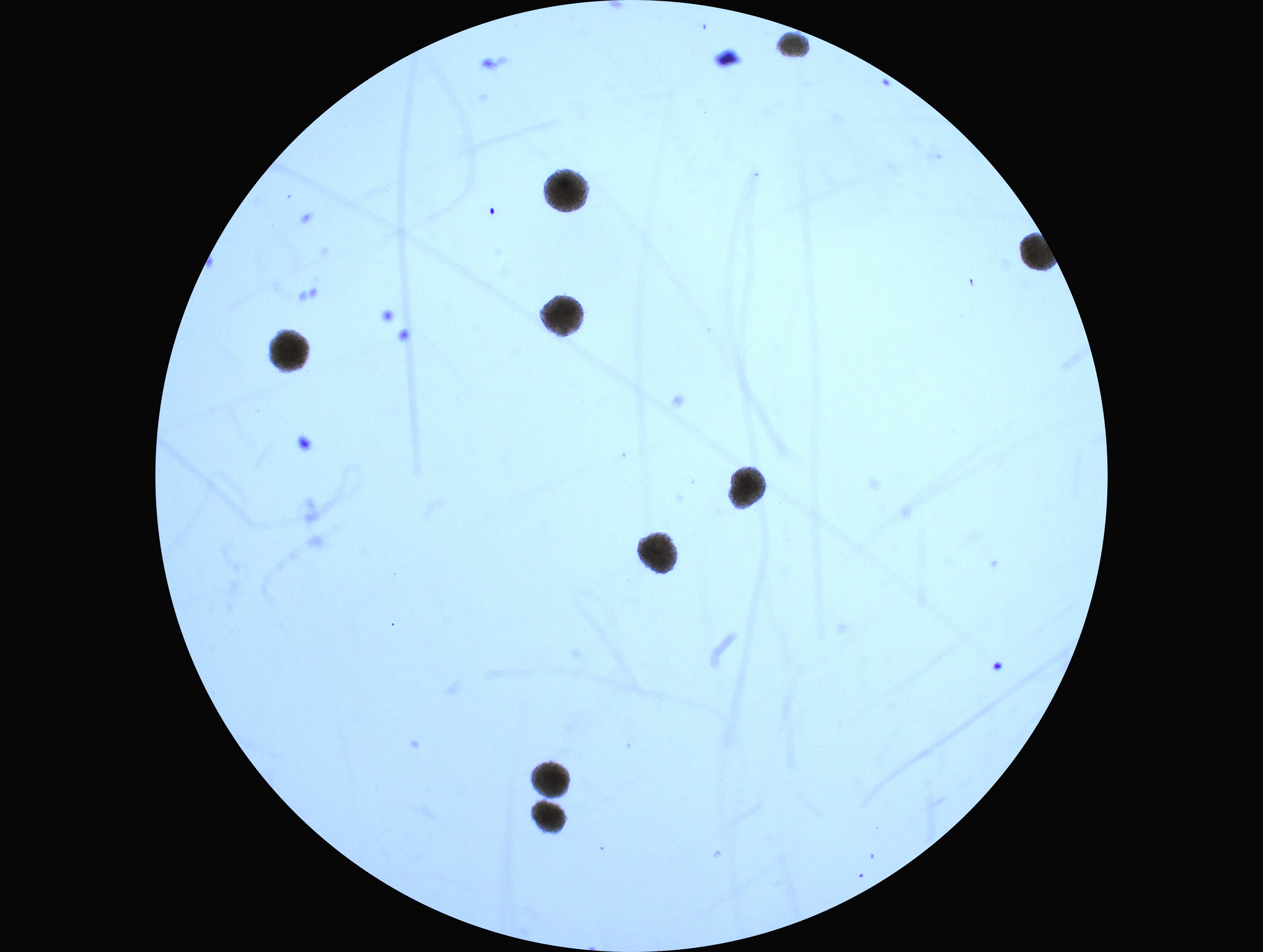

Supplement: Supplementary file 11 — Source data Fig. 3 [file 44319_2025_619_MOESM11_ESM.zip › Figure 3/C,D,F,G/Raw images_mask/PA_day6/MN 12C1 A C2 D06 2x/12C1_d6A_0002.jpg]

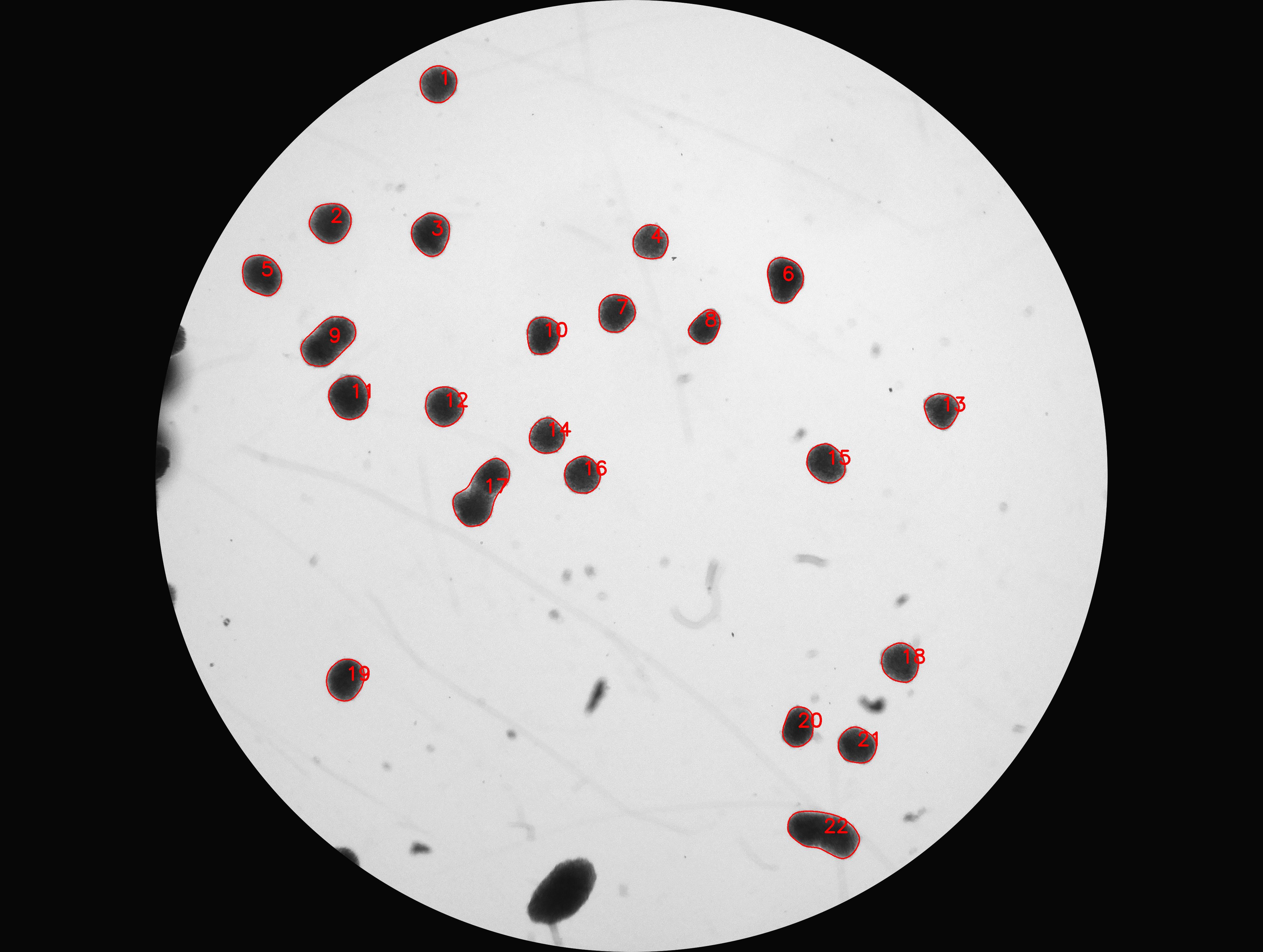

Supplement: Supplementary file 11 — Source data Fig. 3 [file 44319_2025_619_MOESM11_ESM.zip › Figure 3/C,D,F,G/Raw images_mask/PA_day6/MN 12C1 A C2 D06 2x/R_12C1_d6A_0000.jpg]

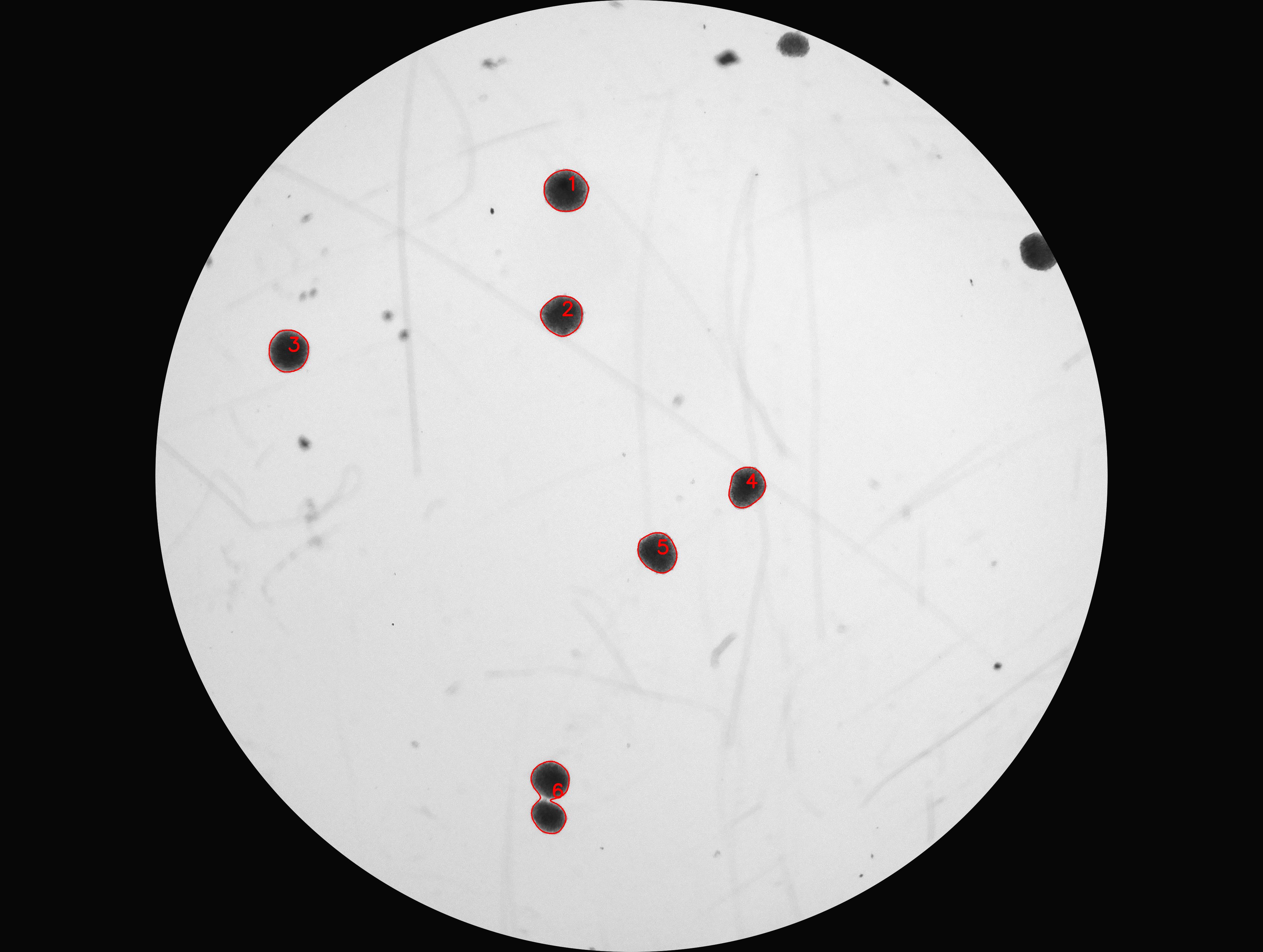

Supplement: Supplementary file 11 — Source data Fig. 3 [file 44319_2025_619_MOESM11_ESM.zip › Figure 3/C,D,F,G/Raw images_mask/PA_day6/MN 12C1 A C2 D06 2x/R_12C1_d6A_0002.jpg]

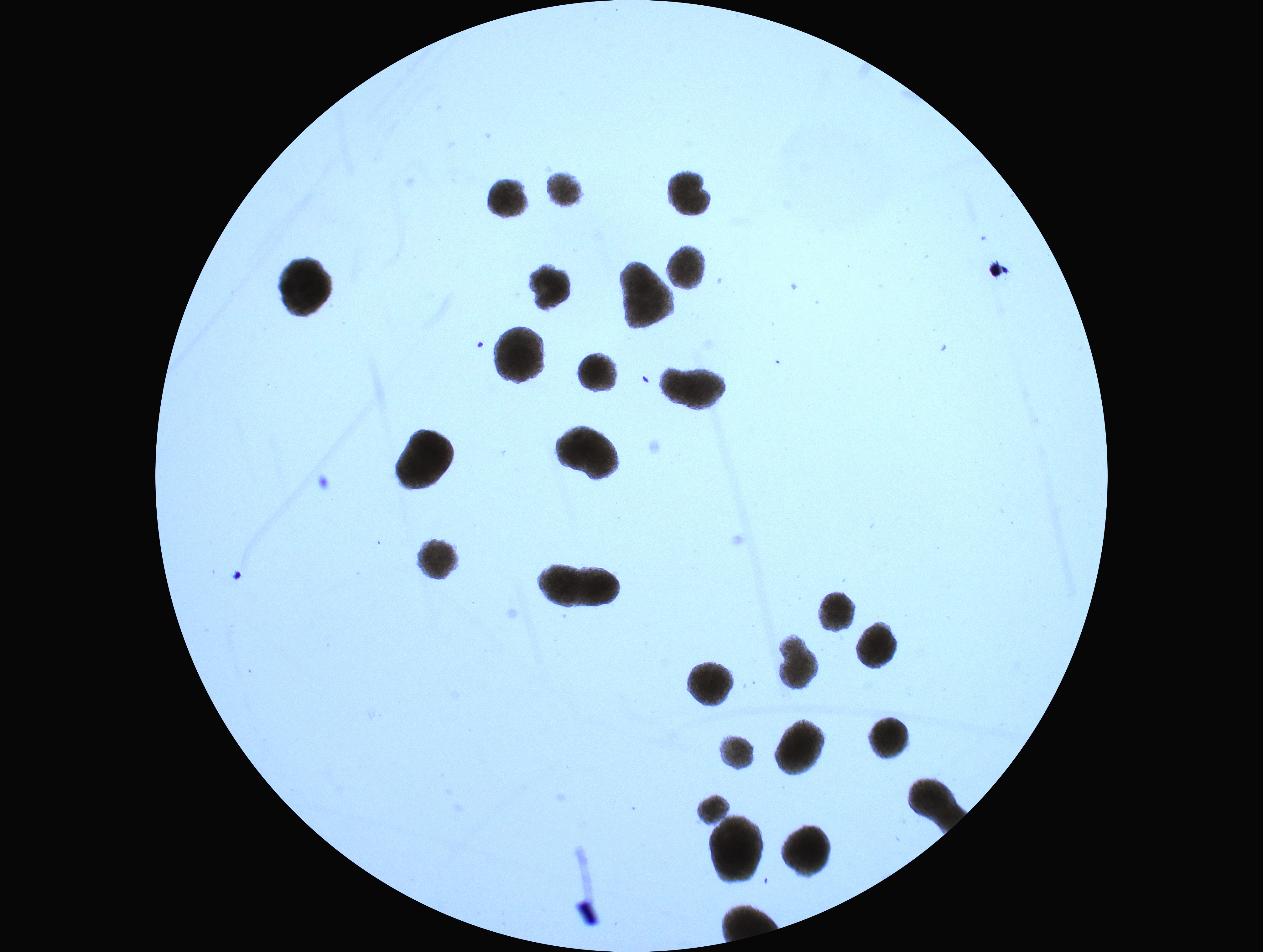

Supplement: Supplementary file 11 — Source data Fig. 3 [file 44319_2025_619_MOESM11_ESM.zip › Figure 3/C,D,F,G/Raw images_mask/PA_day6/MN 12C1 A C2 D06 2x/12C1_d6A_0001.jpg]

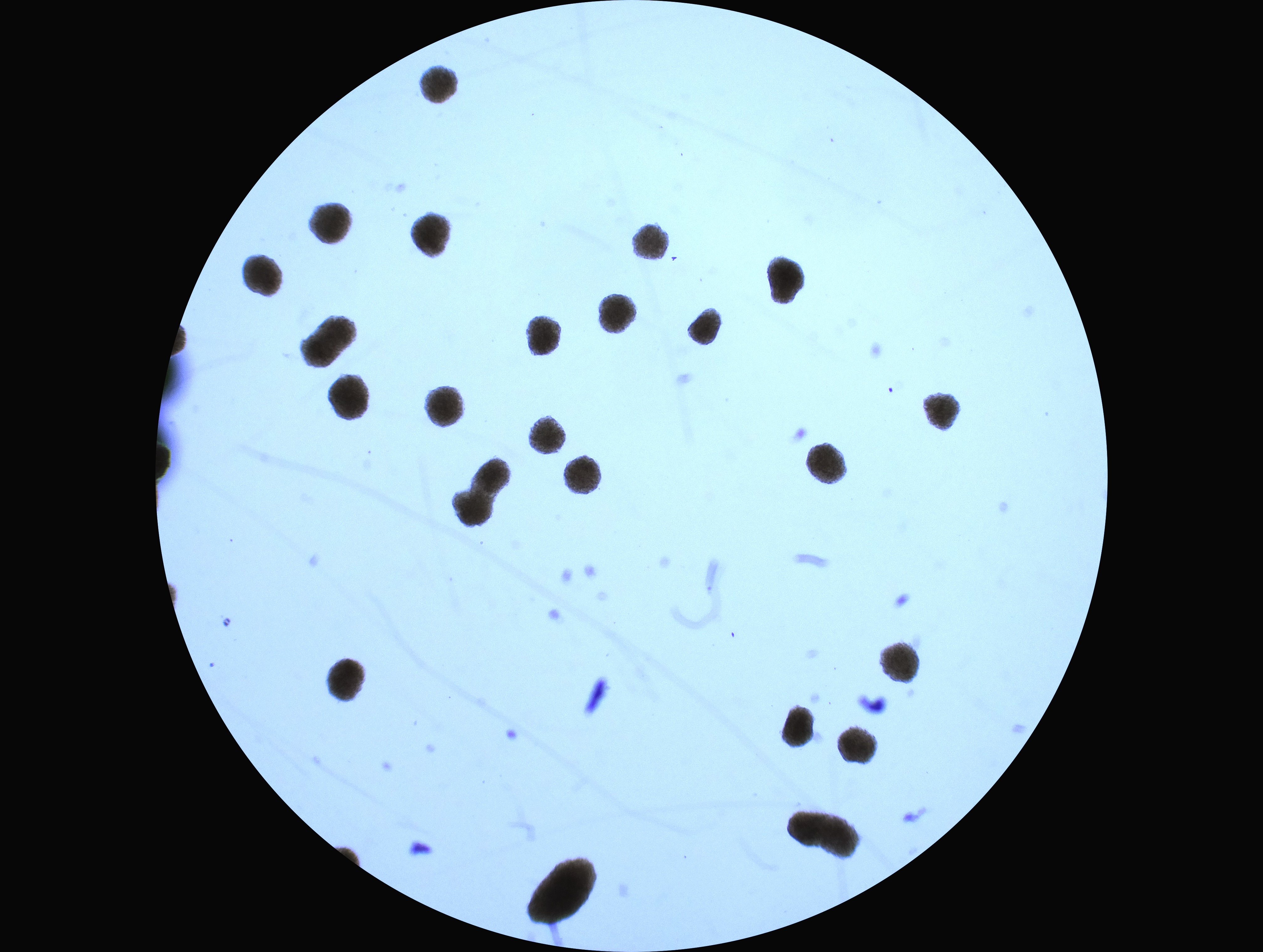

Supplement: Supplementary file 11 — Source data Fig. 3 [file 44319_2025_619_MOESM11_ESM.zip › Figure 3/C,D,F,G/Raw images_mask/PA_day6/MN 12C1 A C2 D06 2x/12C1_d6A_0000.jpg]

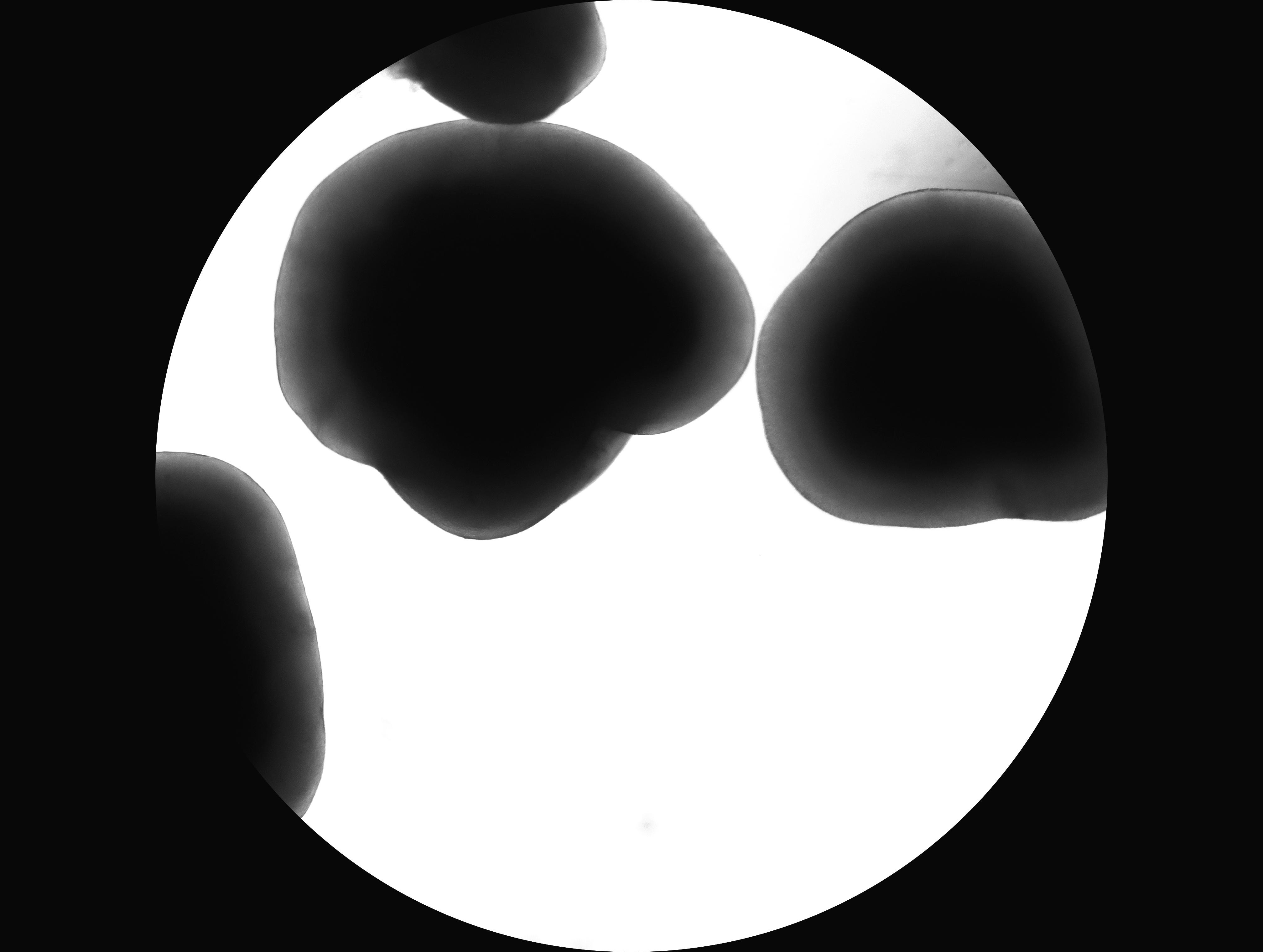

Supplement: Supplementary file 11 — Source data Fig. 3 [file 44319_2025_619_MOESM11_ESM.zip › Figure 3/C,D,F,G/Raw images_mask/OS_day90/MN 12C1 B C4 D90 2x/R_Day90_0004.jpg]

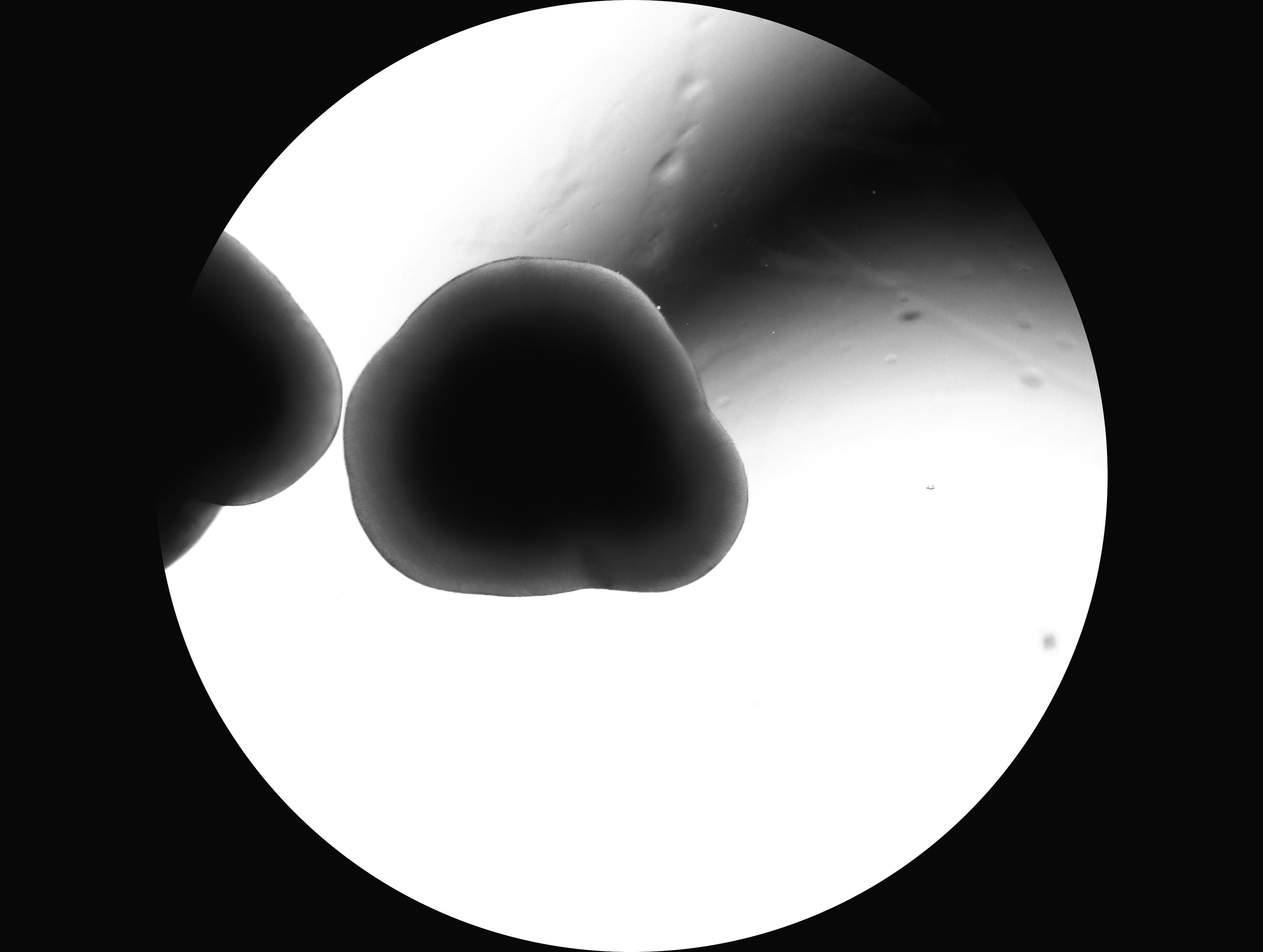

Supplement: Supplementary file 11 — Source data Fig. 3 [file 44319_2025_619_MOESM11_ESM.zip › Figure 3/C,D,F,G/Raw images_mask/OS_day90/MN 12C1 B C4 D90 2x/R_Day90_0005.jpg]

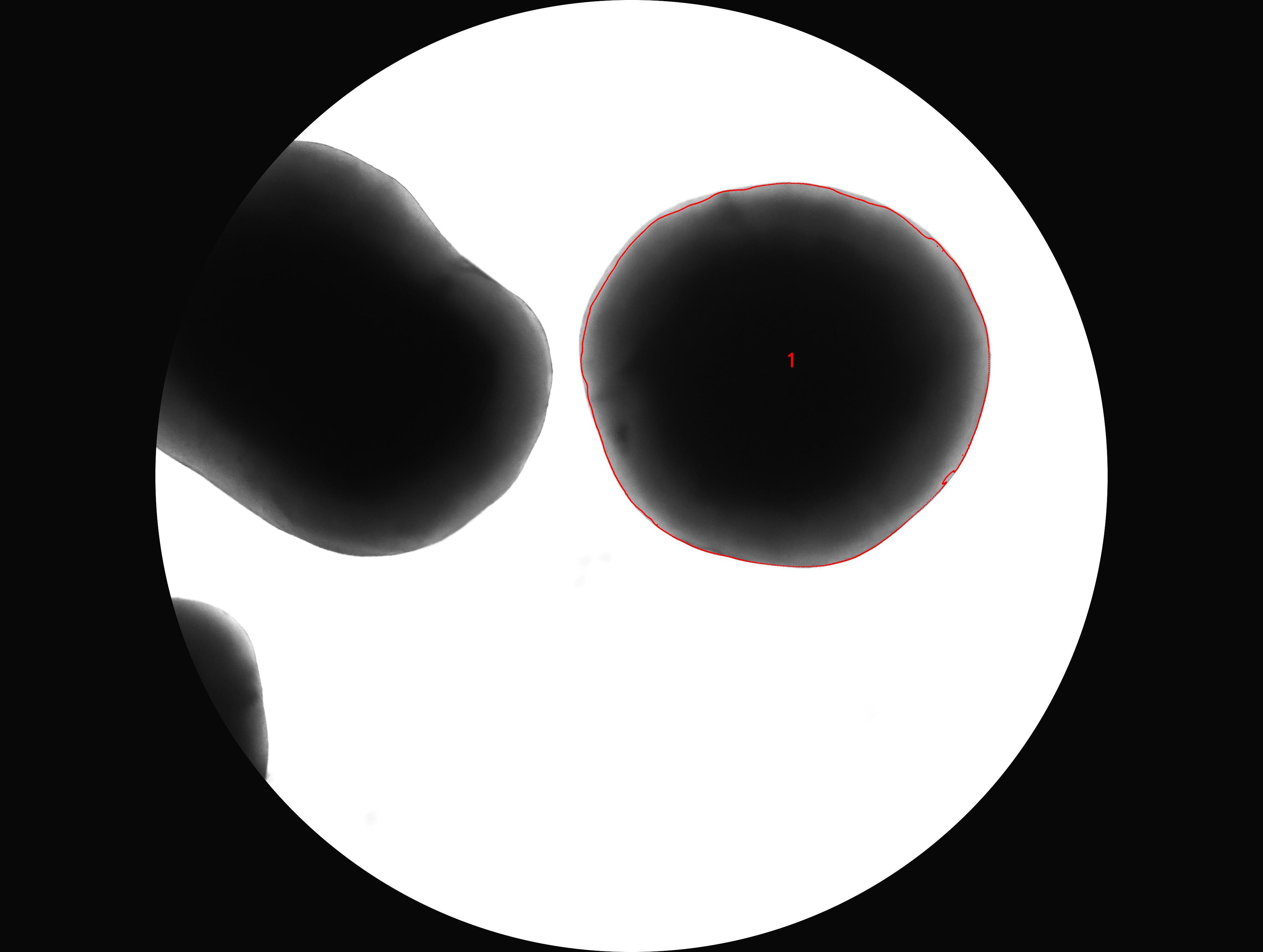

Supplement: Supplementary file 11 — Source data Fig. 3 [file 44319_2025_619_MOESM11_ESM.zip › Figure 3/C,D,F,G/Raw images_mask/OS_day90/MN 12C1 B C4 D90 2x/R_Day90_0006.jpg]

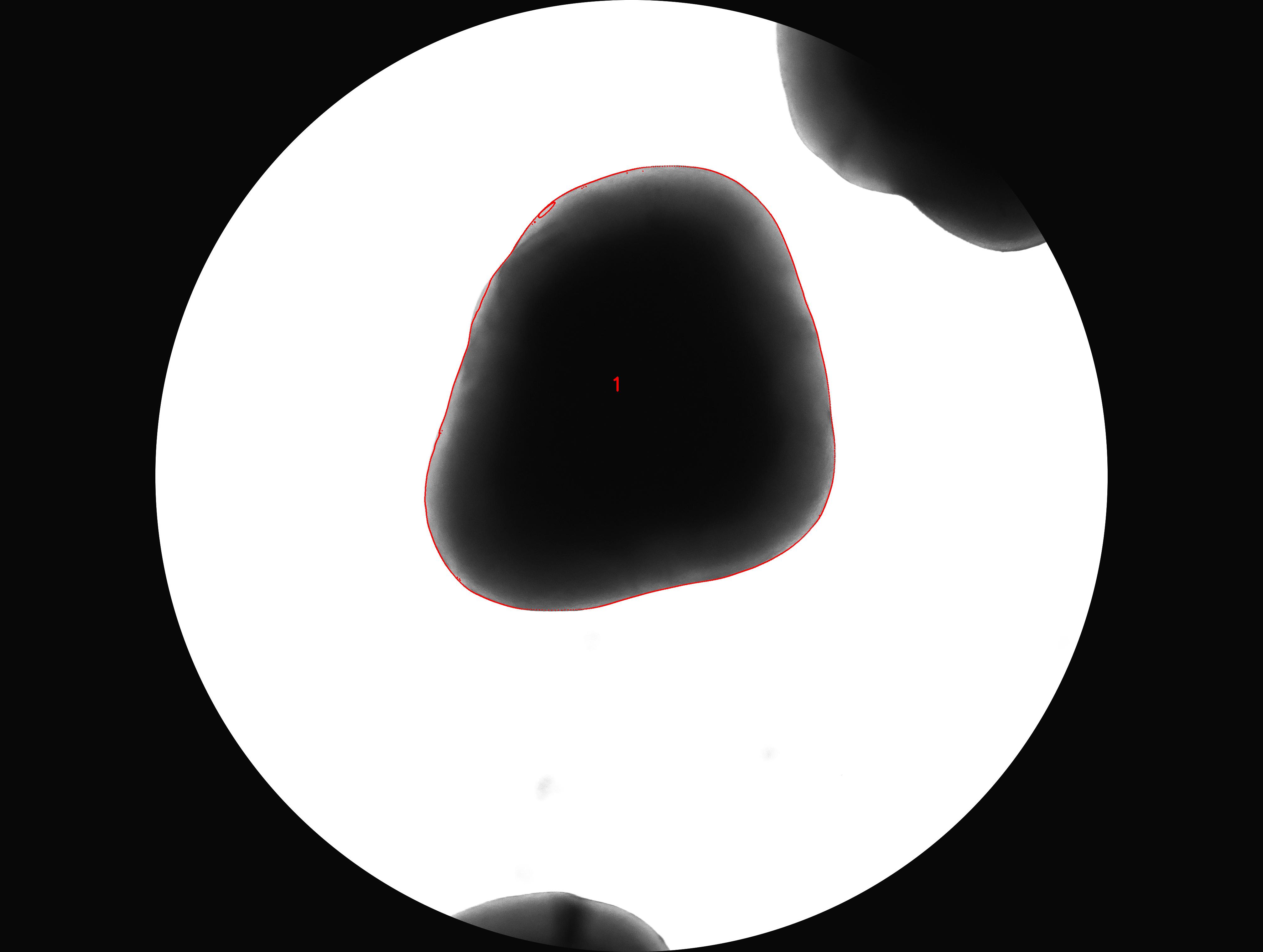

Supplement: Supplementary file 11 — Source data Fig. 3 [file 44319_2025_619_MOESM11_ESM.zip › Figure 3/C,D,F,G/Raw images_mask/OS_day90/MN 12C1 B C4 D90 2x/R_Day90_0003.jpg]

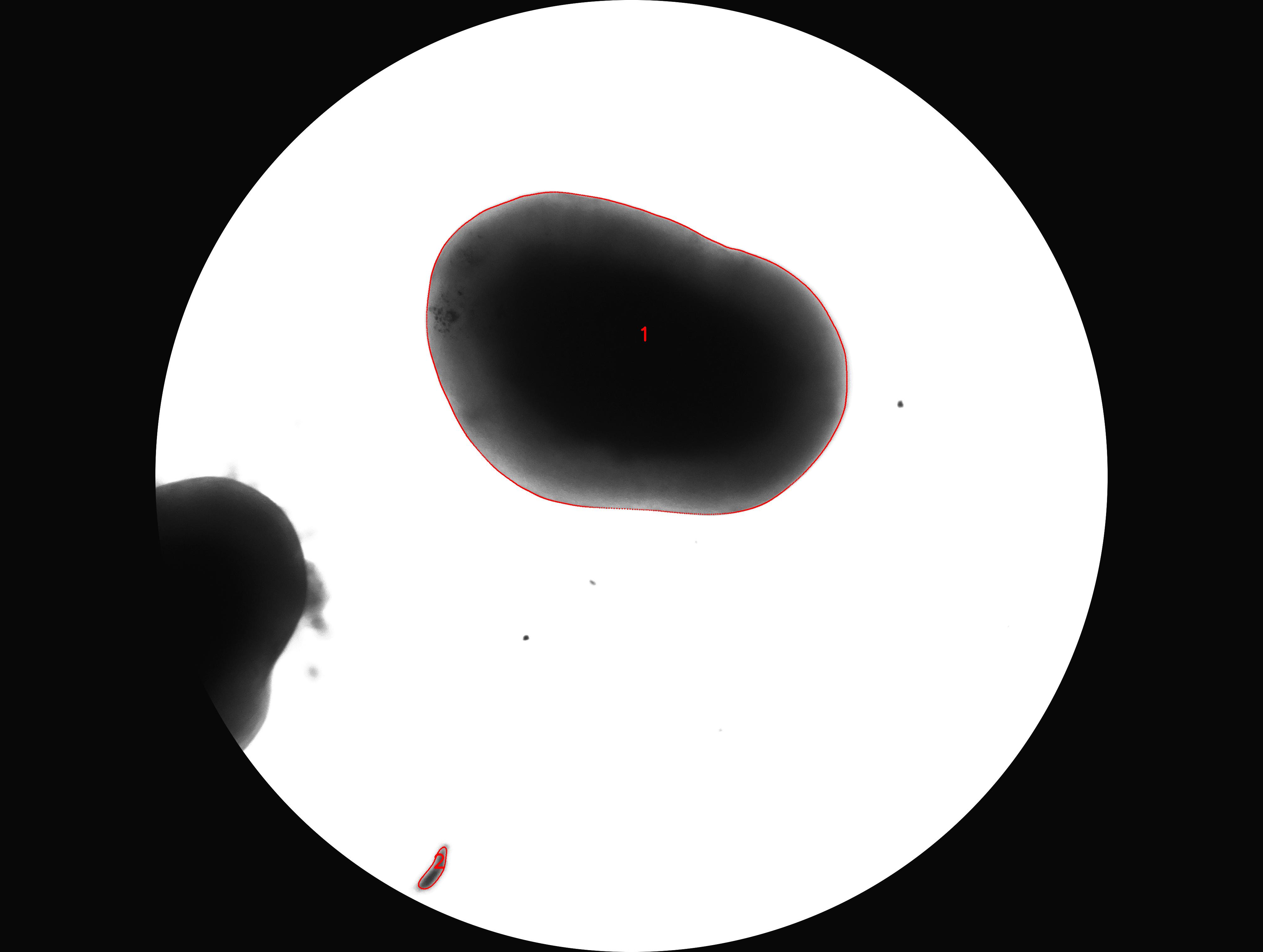

Supplement: Supplementary file 11 — Source data Fig. 3 [file 44319_2025_619_MOESM11_ESM.zip › Figure 3/C,D,F,G/Raw images_mask/OS_day90/MN 12C1 B C4 D90 2x/R_Day90_0001.jpg]

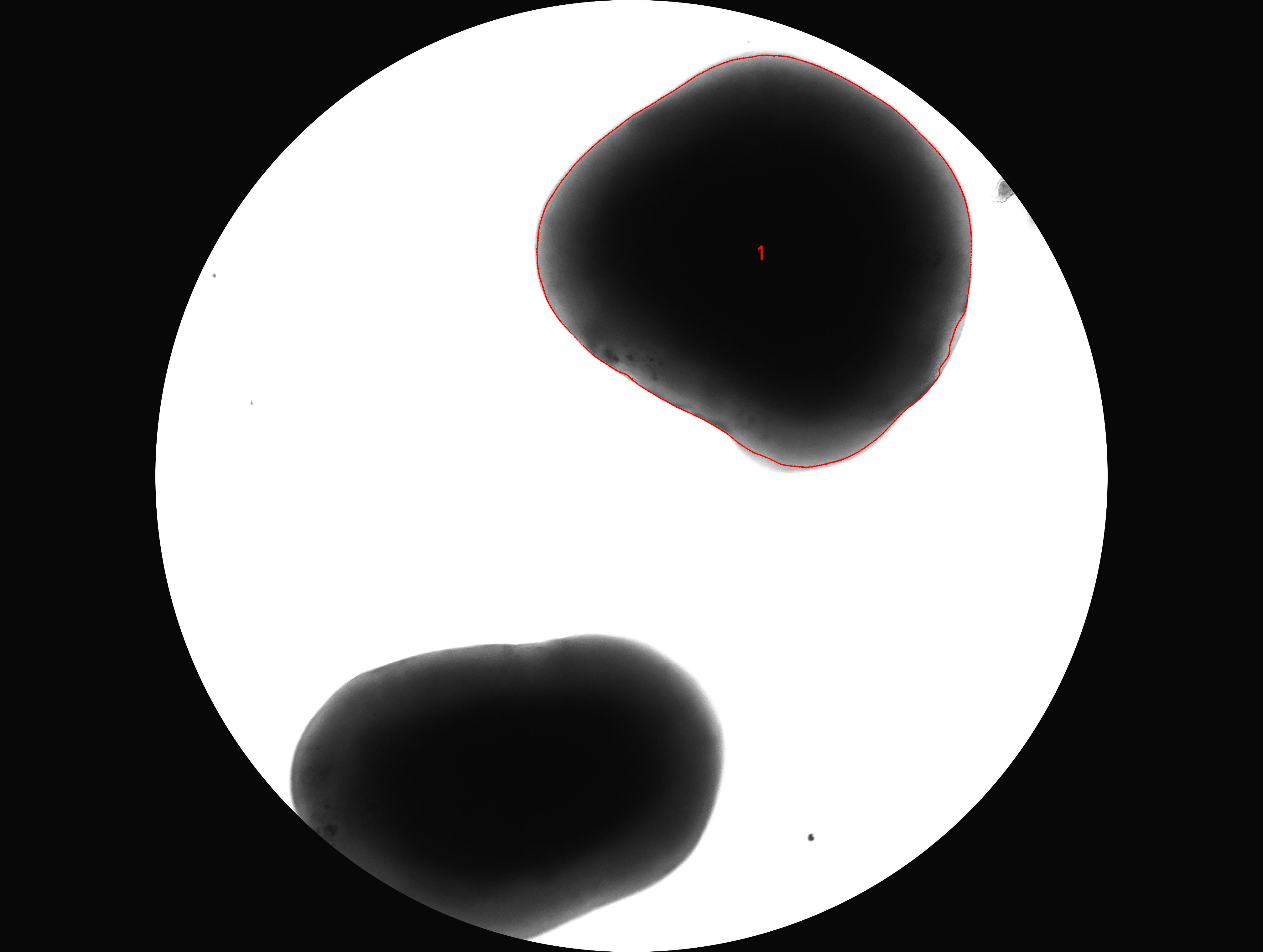

Supplement: Supplementary file 11 — Source data Fig. 3 [file 44319_2025_619_MOESM11_ESM.zip › Figure 3/C,D,F,G/Raw images_mask/OS_day90/MN 12C1 B C4 D90 2x/R_Day90_0000.jpg]

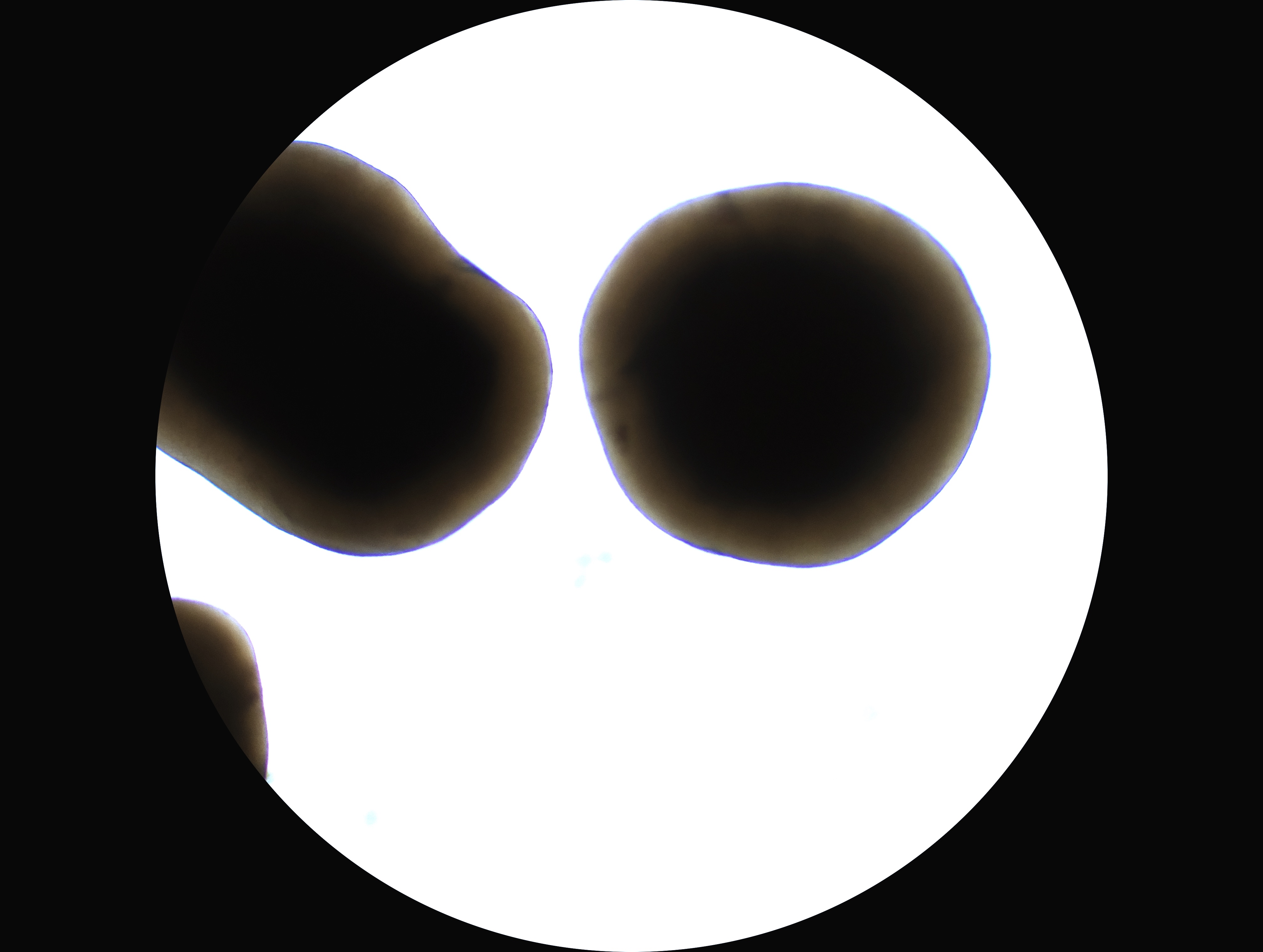

Supplement: Supplementary file 11 — Source data Fig. 3 [file 44319_2025_619_MOESM11_ESM.zip › Figure 3/C,D,F,G/Raw images_mask/OS_day90/MN 12C1 B C4 D90 2x/Day90_0006.jpg]

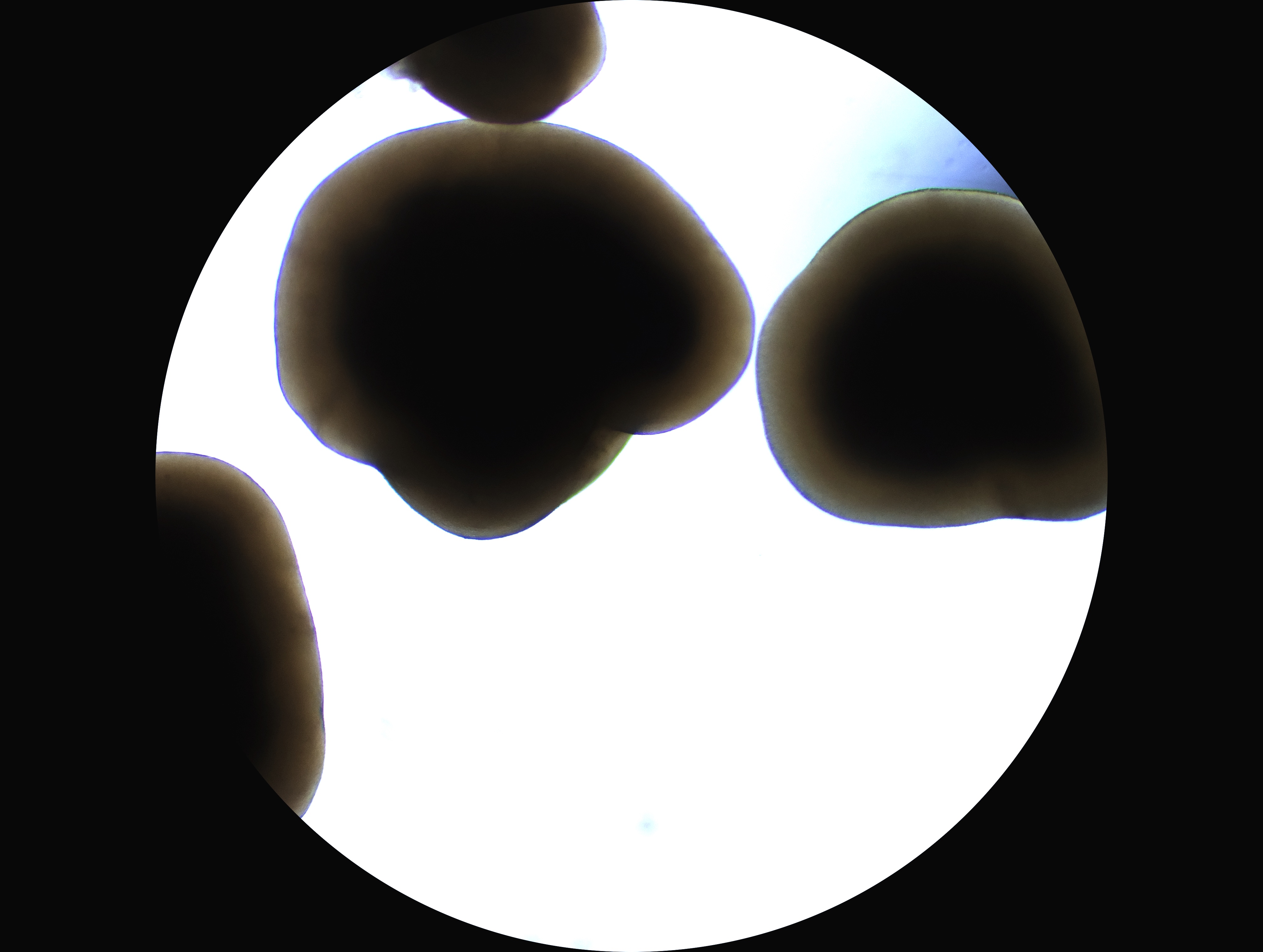

Supplement: Supplementary file 11 — Source data Fig. 3 [file 44319_2025_619_MOESM11_ESM.zip › Figure 3/C,D,F,G/Raw images_mask/OS_day90/MN 12C1 B C4 D90 2x/Day90_0004.jpg]

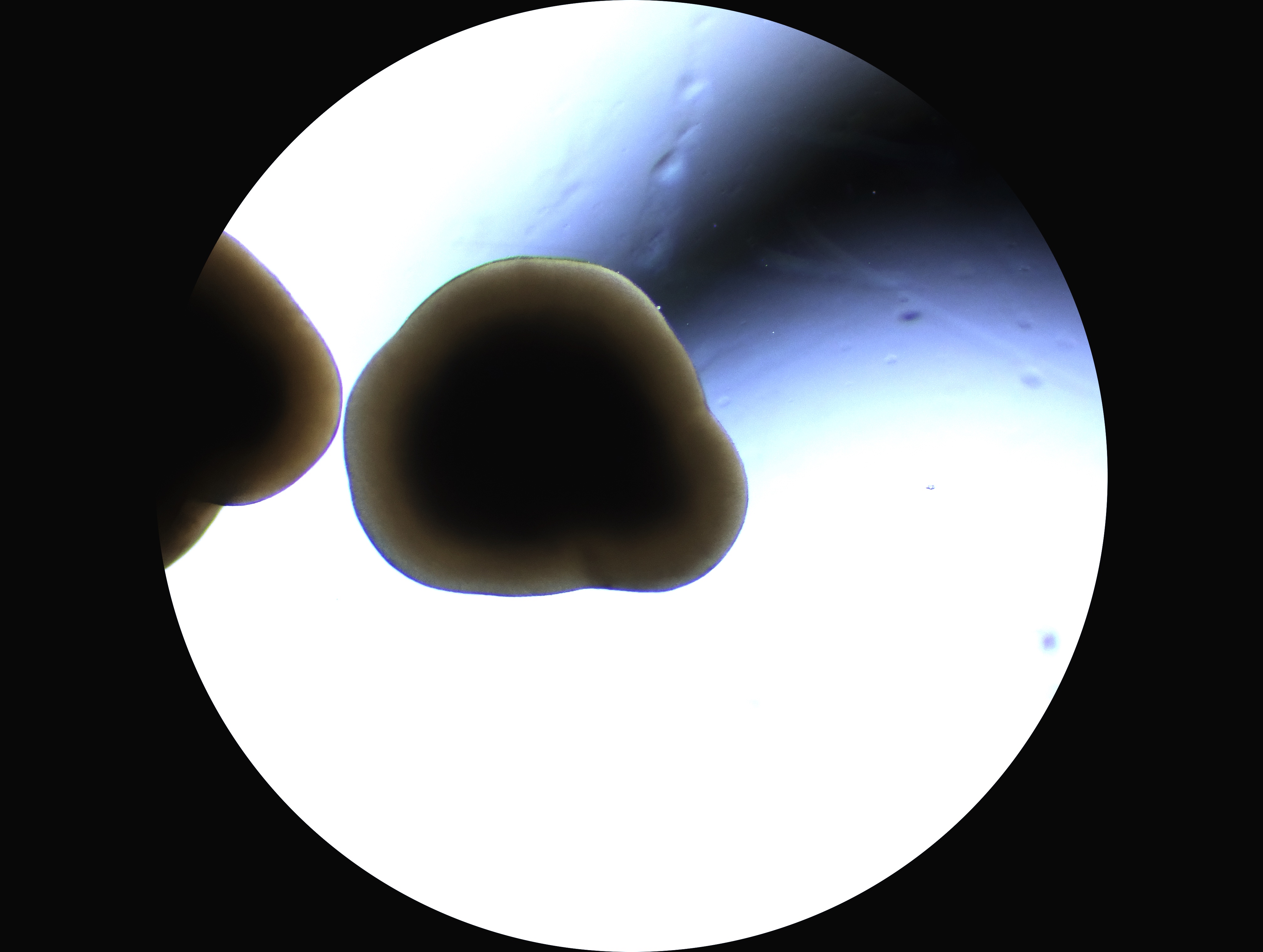

Supplement: Supplementary file 11 — Source data Fig. 3 [file 44319_2025_619_MOESM11_ESM.zip › Figure 3/C,D,F,G/Raw images_mask/OS_day90/MN 12C1 B C4 D90 2x/Day90_0005.jpg]

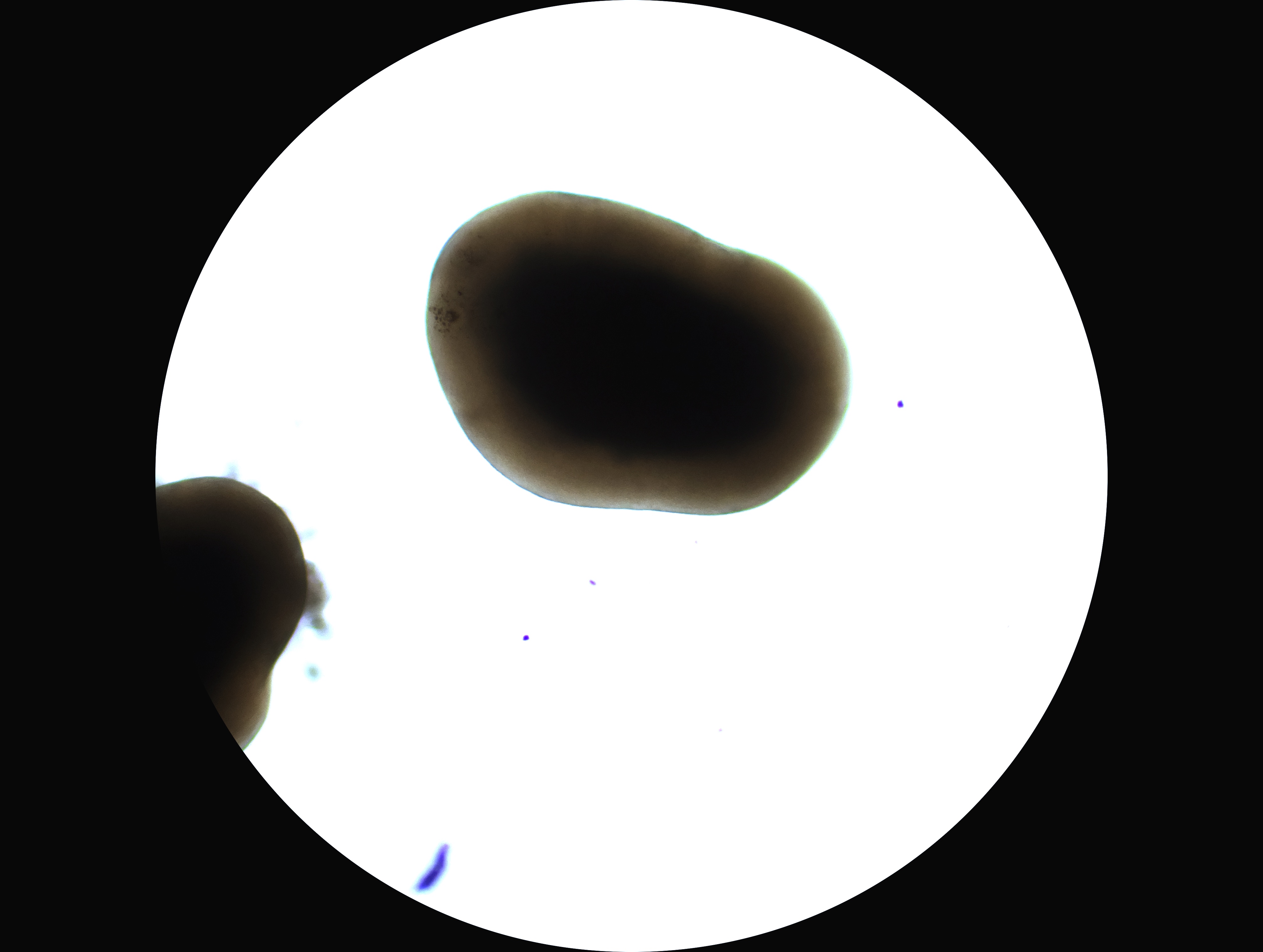

Supplement: Supplementary file 11 — Source data Fig. 3 [file 44319_2025_619_MOESM11_ESM.zip › Figure 3/C,D,F,G/Raw images_mask/OS_day90/MN 12C1 B C4 D90 2x/Day90_0001.jpg]

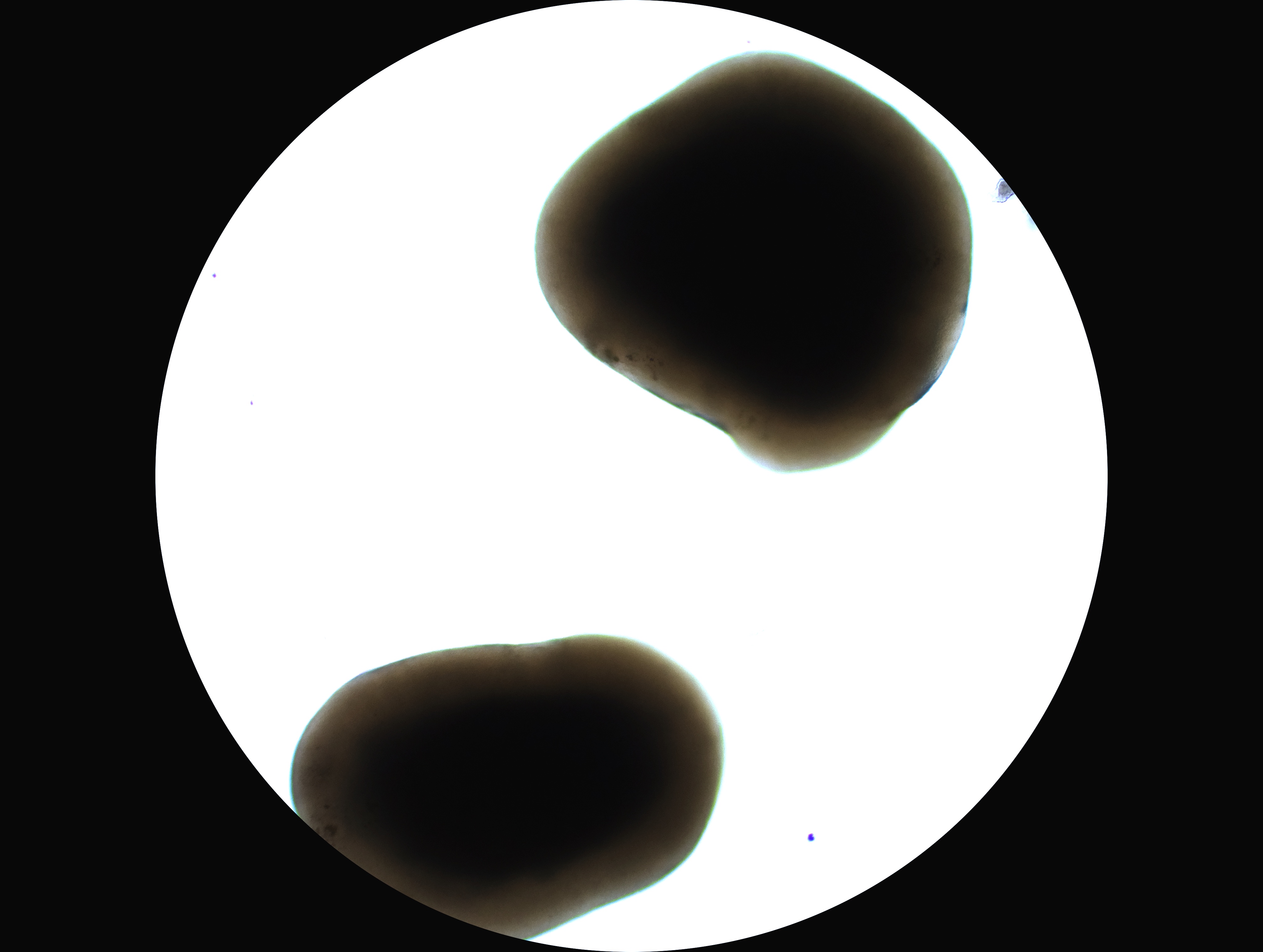

Supplement: Supplementary file 11 — Source data Fig. 3 [file 44319_2025_619_MOESM11_ESM.zip › Figure 3/C,D,F,G/Raw images_mask/OS_day90/MN 12C1 B C4 D90 2x/Day90_0000.jpg]

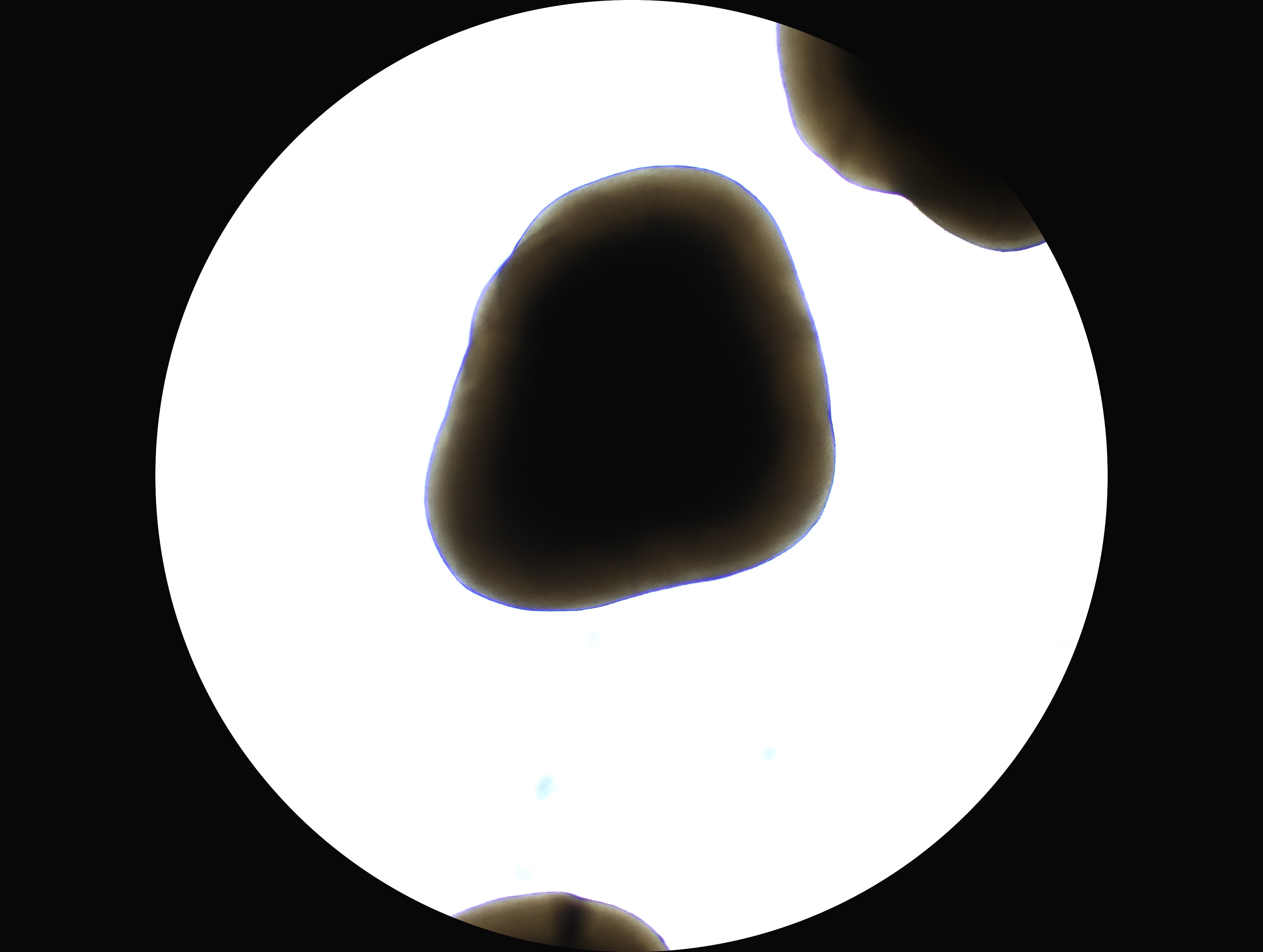

Supplement: Supplementary file 11 — Source data Fig. 3 [file 44319_2025_619_MOESM11_ESM.zip › Figure 3/C,D,F,G/Raw images_mask/OS_day90/MN 12C1 B C4 D90 2x/Day90_0003.jpg]

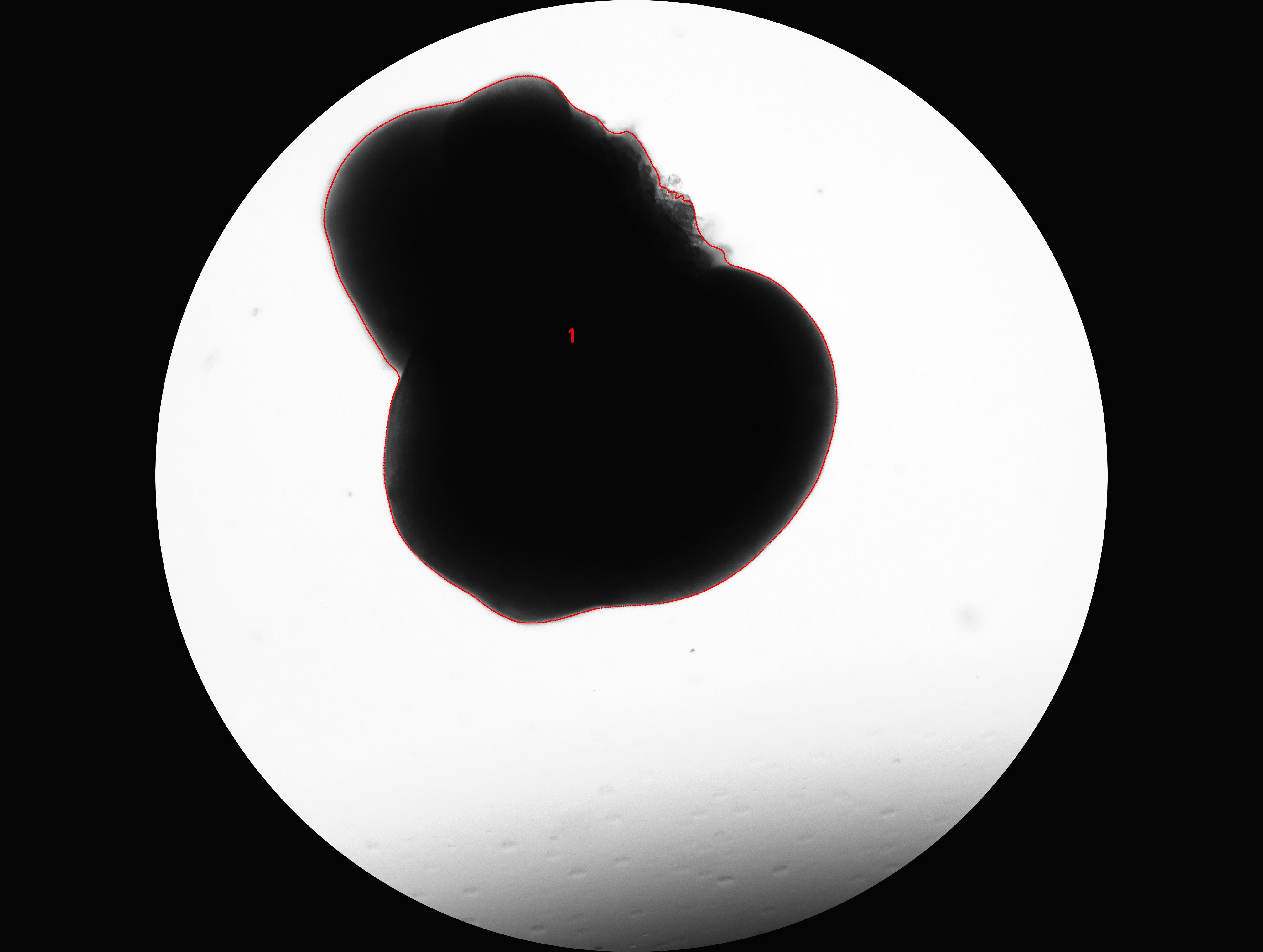

Supplement: Supplementary file 11 — Source data Fig. 3 [file 44319_2025_619_MOESM11_ESM.zip › Figure 3/C,D,F,G/Raw images_mask/OS_day90/MN 12C1 B C8 D90 2x/R_Day 90_0032.jpg]

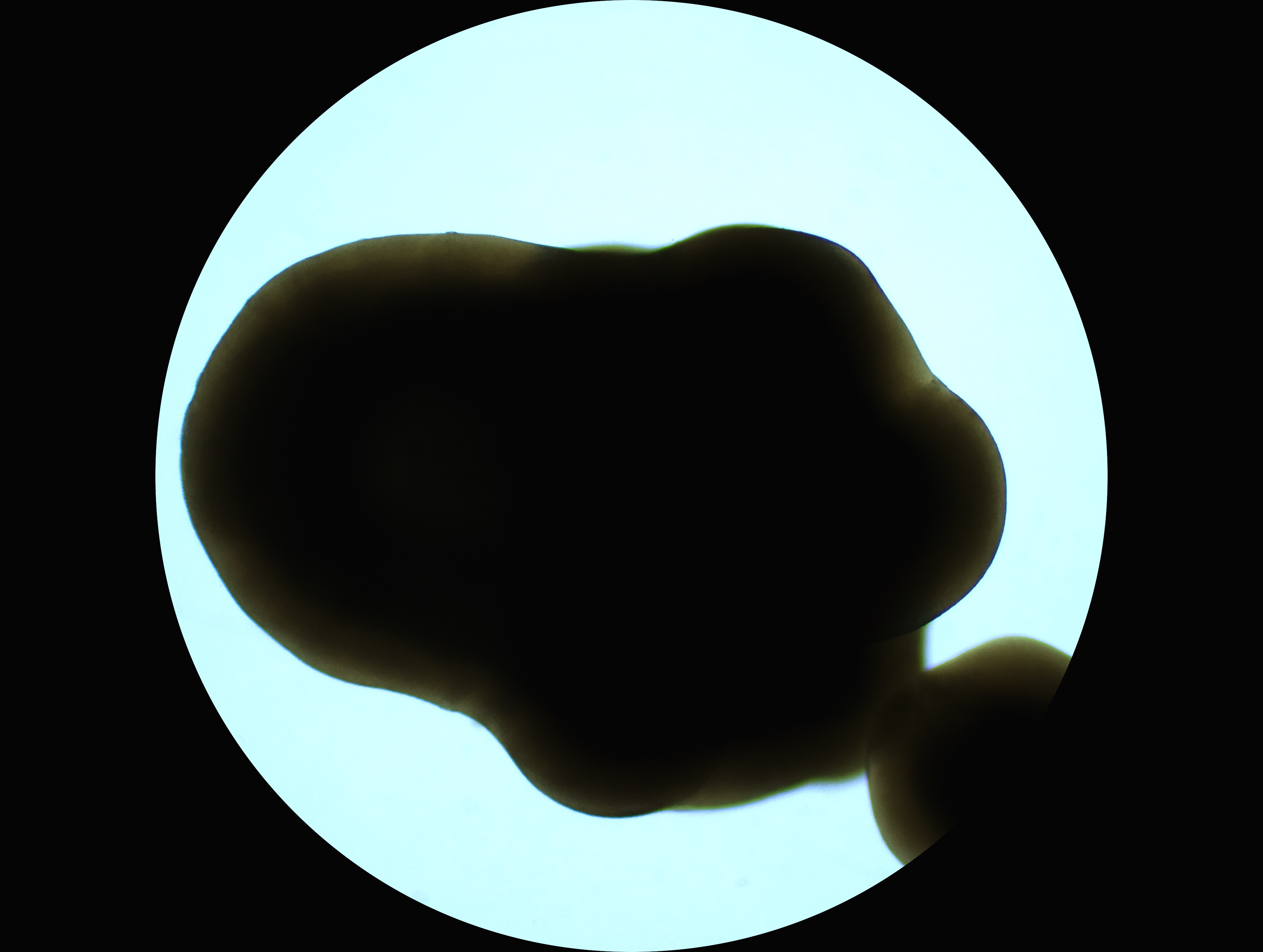

Supplement: Supplementary file 11 — Source data Fig. 3 [file 44319_2025_619_MOESM11_ESM.zip › Figure 3/C,D,F,G/Raw images_mask/OS_day90/MN 12C1 B C8 D90 2x/Day 90_0015.jpg]

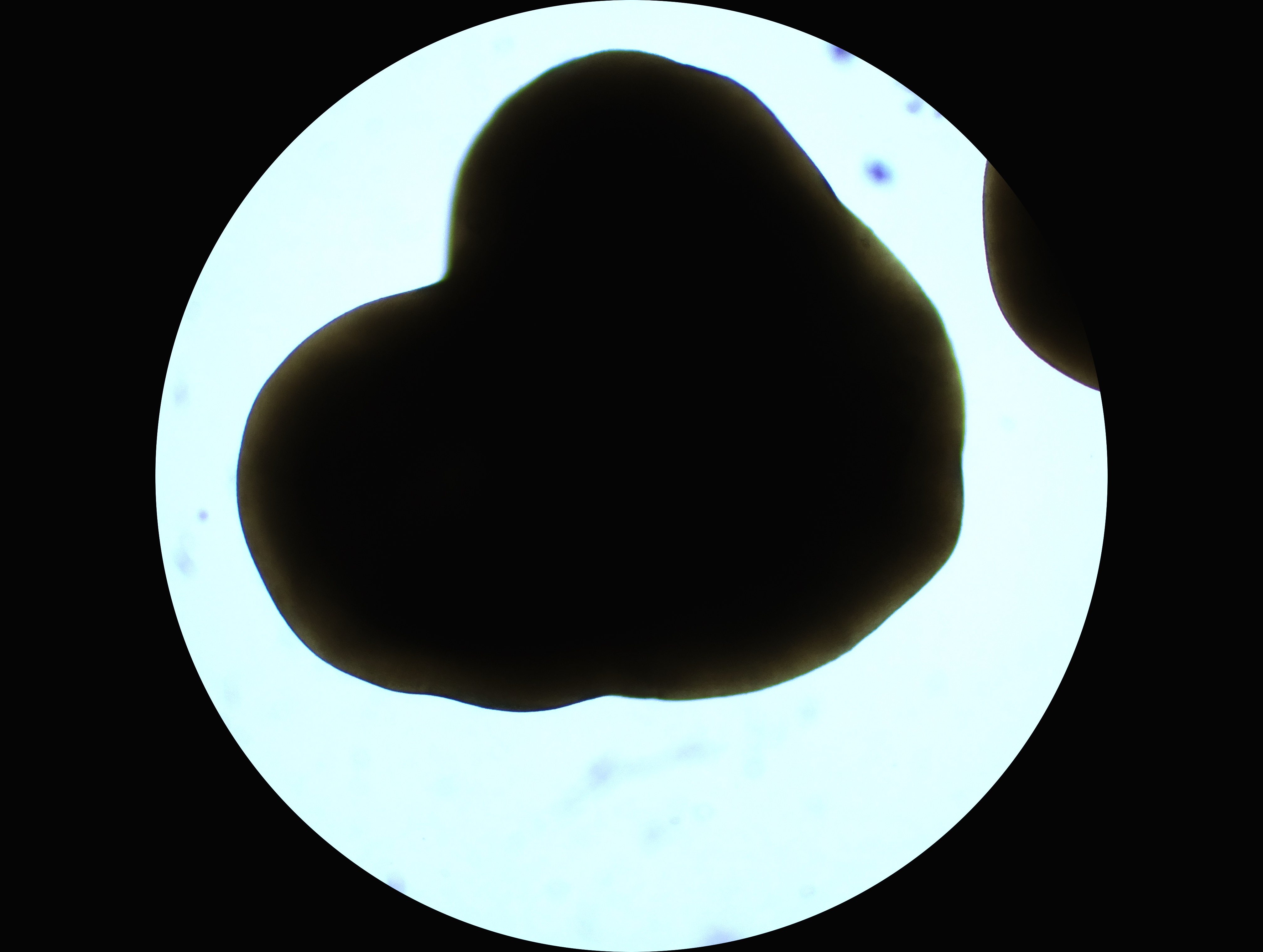

Supplement: Supplementary file 11 — Source data Fig. 3 [file 44319_2025_619_MOESM11_ESM.zip › Figure 3/C,D,F,G/Raw images_mask/OS_day90/MN 12C1 B C8 D90 2x/Day 90_0001.jpg]

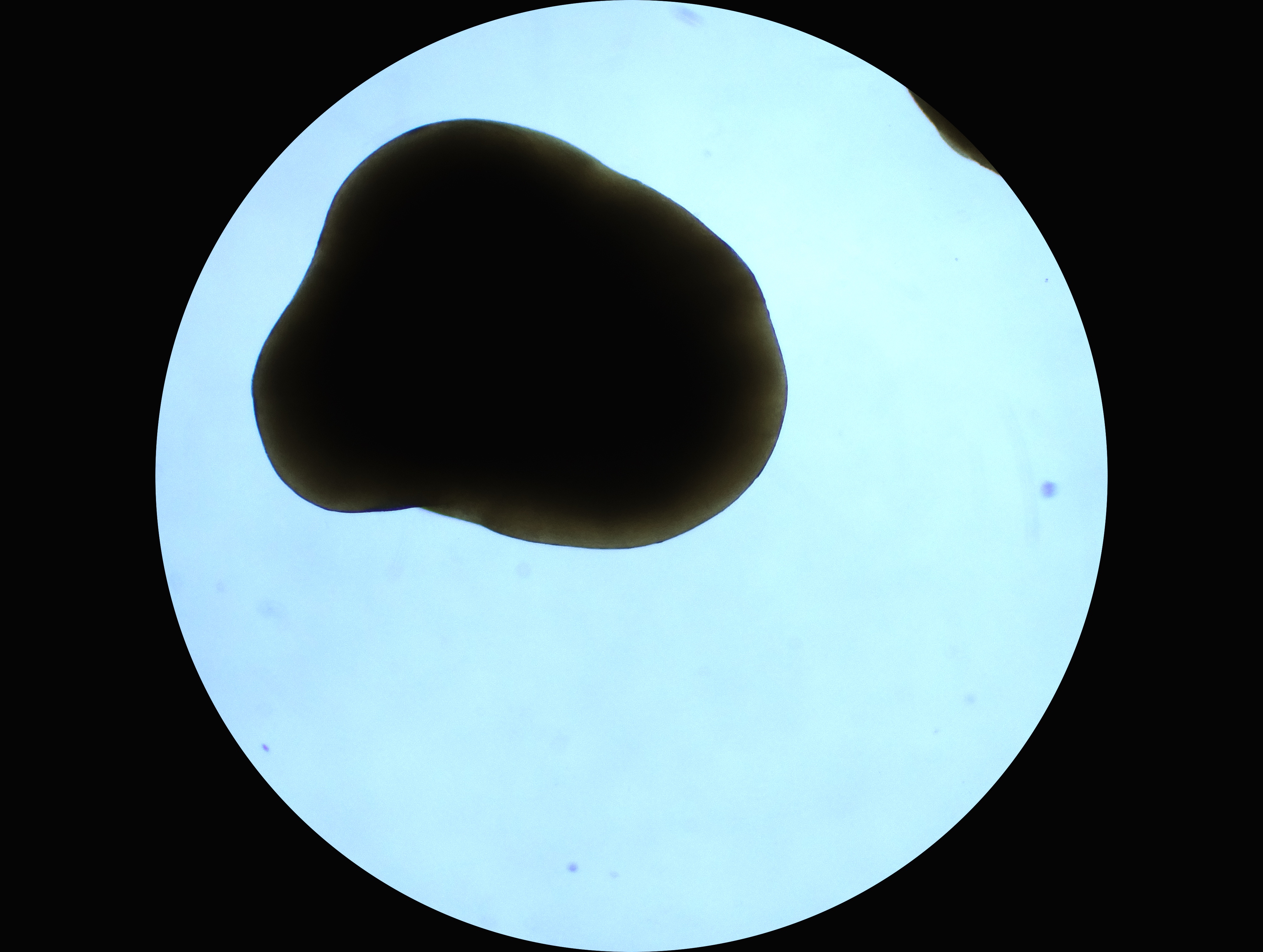

Supplement: Supplementary file 11 — Source data Fig. 3 [file 44319_2025_619_MOESM11_ESM.zip › Figure 3/C,D,F,G/Raw images_mask/OS_day90/MN 12C1 B C8 D90 2x/Day 90_0029.jpg]

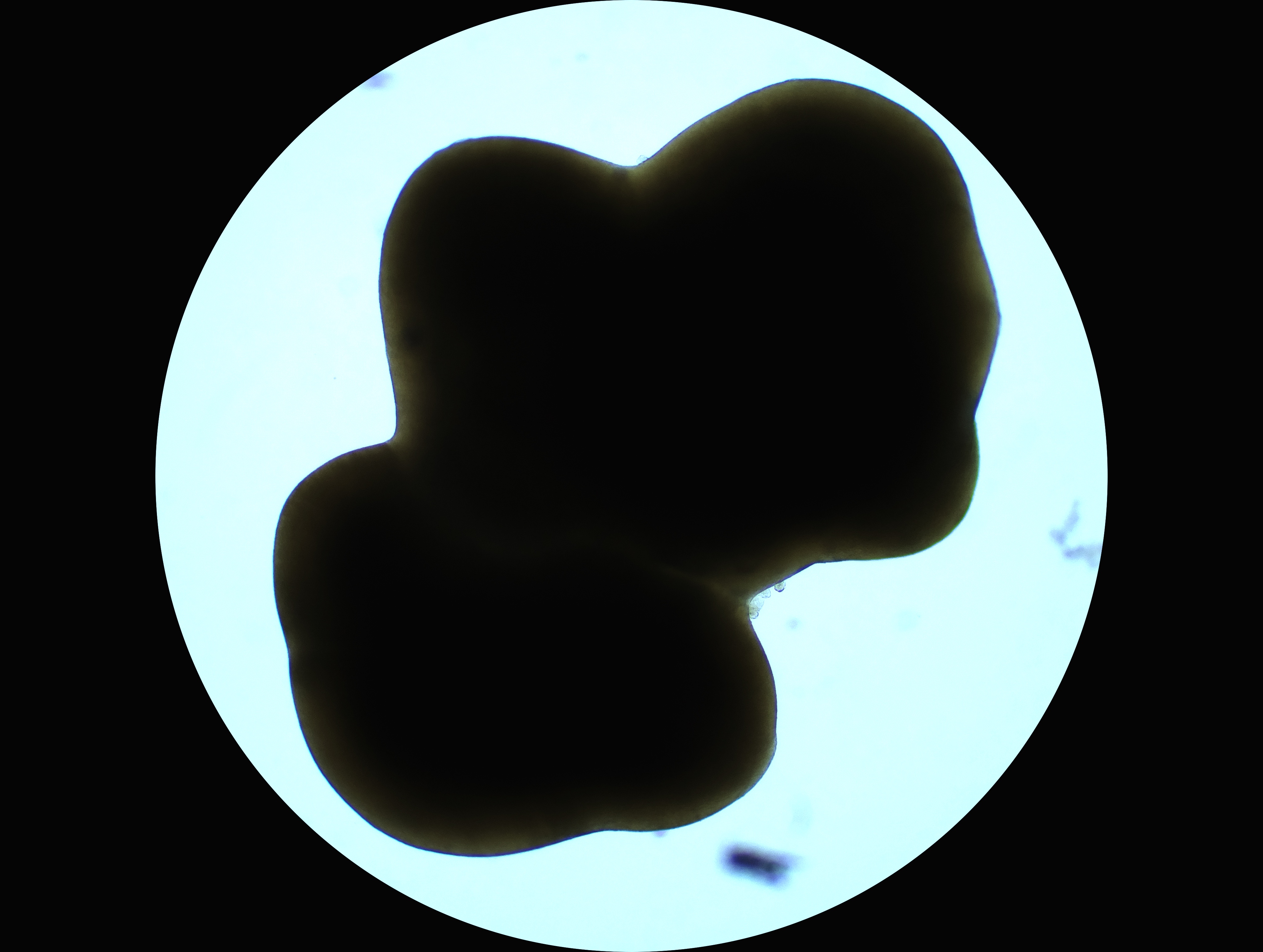

Supplement: Supplementary file 11 — Source data Fig. 3 [file 44319_2025_619_MOESM11_ESM.zip › Figure 3/C,D,F,G/Raw images_mask/OS_day90/MN 12C1 B C8 D90 2x/Day 90_0000.jpg]

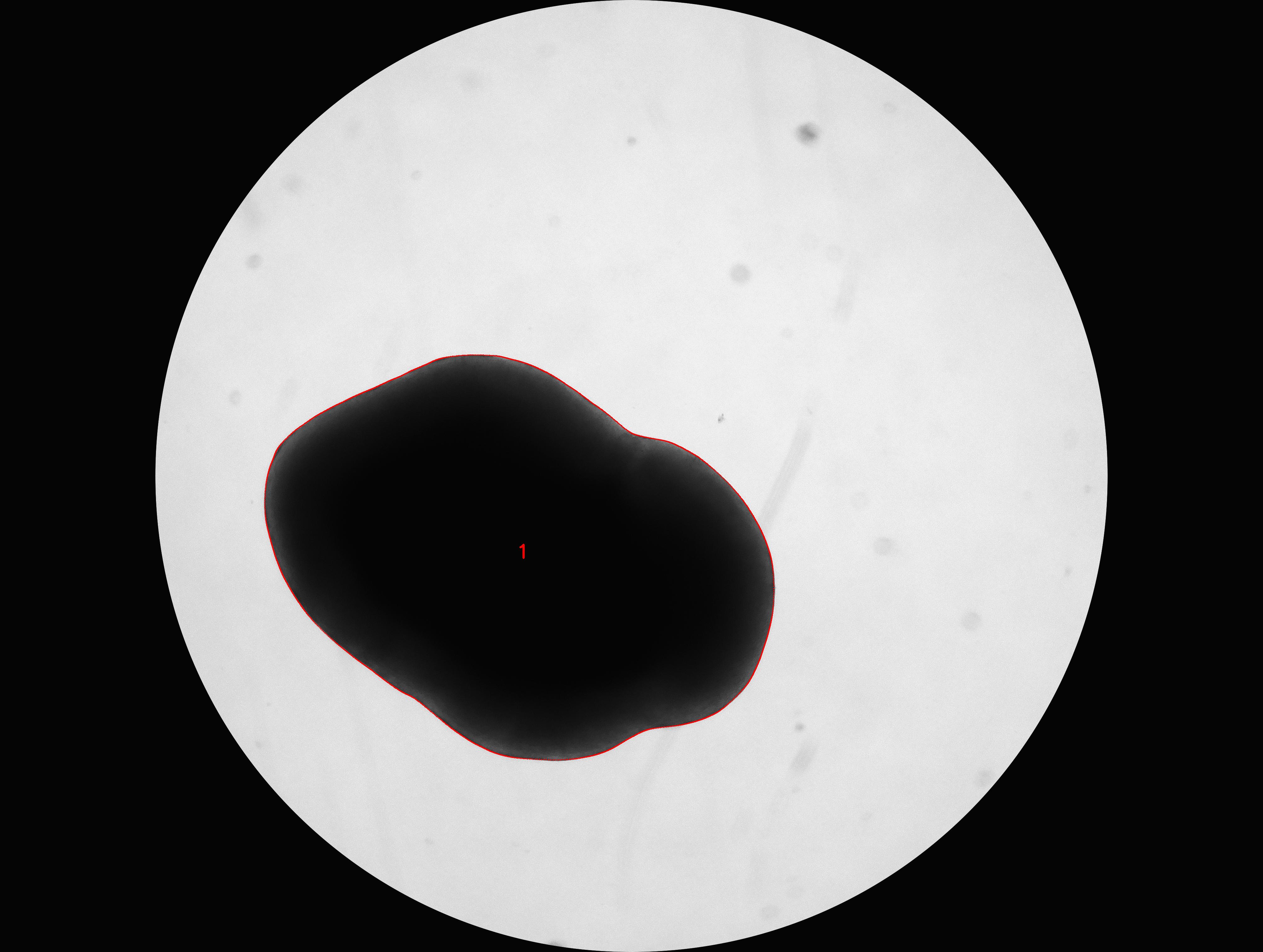

Supplement: Supplementary file 11 — Source data Fig. 3 [file 44319_2025_619_MOESM11_ESM.zip › Figure 3/C,D,F,G/Raw images_mask/OS_day90/MN 12C1 B C8 D90 2x/R_Day 90_0027.jpg]

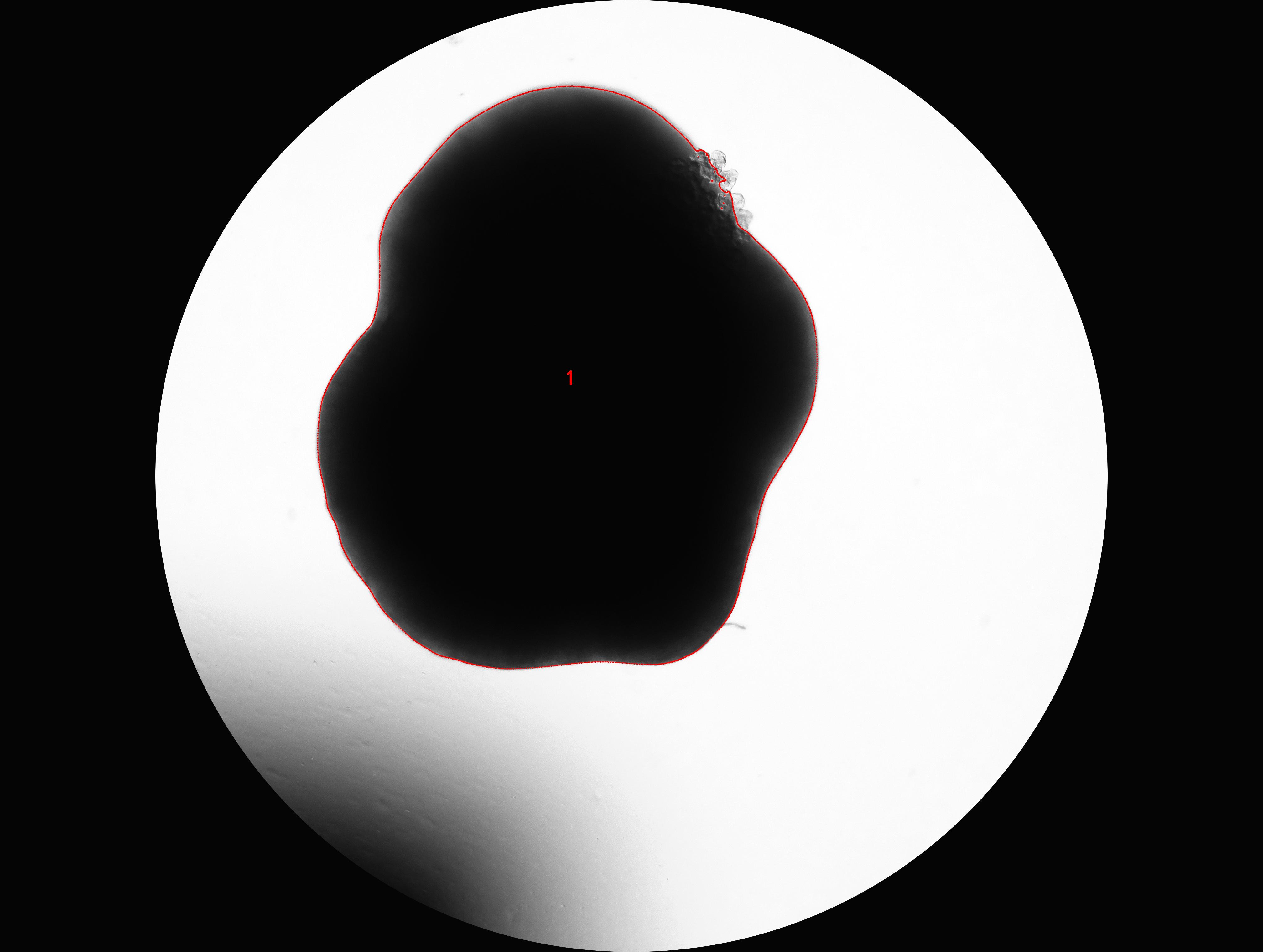

Supplement: Supplementary file 11 — Source data Fig. 3 [file 44319_2025_619_MOESM11_ESM.zip › Figure 3/C,D,F,G/Raw images_mask/OS_day90/MN 12C1 B C8 D90 2x/R_Day 90_0033.jpg]

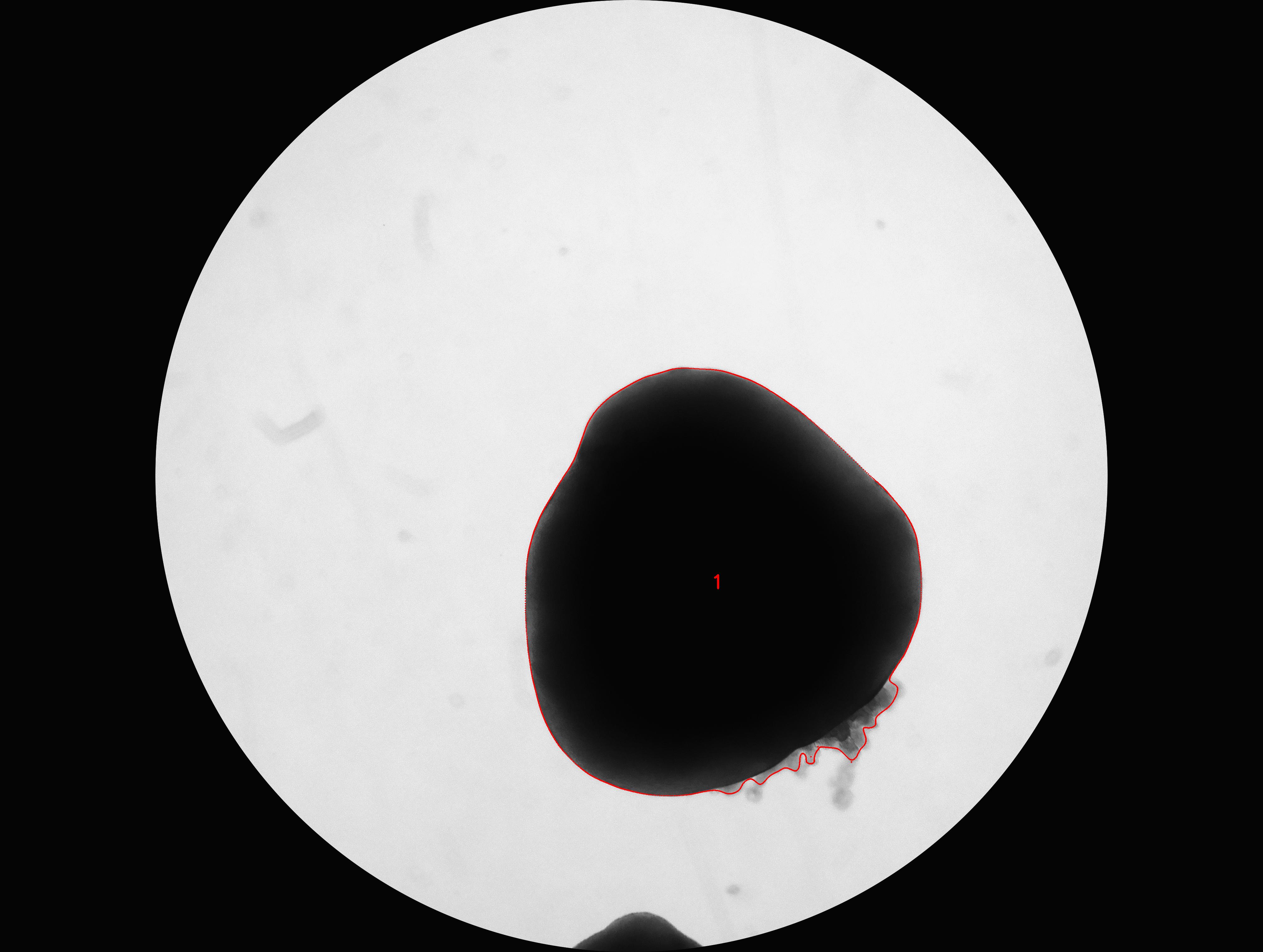

Supplement: Supplementary file 11 — Source data Fig. 3 [file 44319_2025_619_MOESM11_ESM.zip › Figure 3/C,D,F,G/Raw images_mask/OS_day90/MN 12C1 B C8 D90 2x/R_Day 90_0025.jpg]

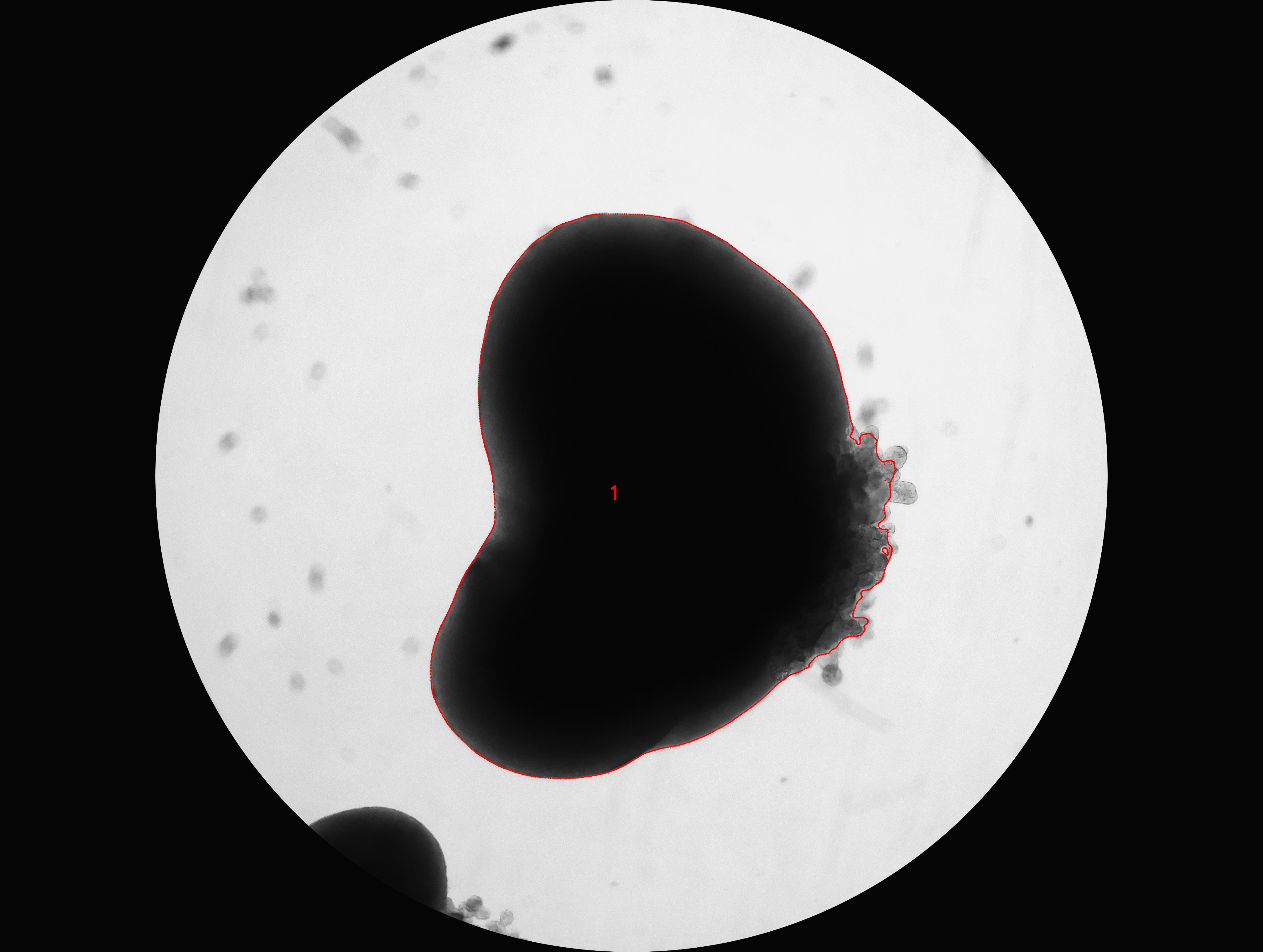

Supplement: Supplementary file 11 — Source data Fig. 3 [file 44319_2025_619_MOESM11_ESM.zip › Figure 3/C,D,F,G/Raw images_mask/OS_day90/MN 12C1 B C8 D90 2x/R_Day 90_0031.jpg]

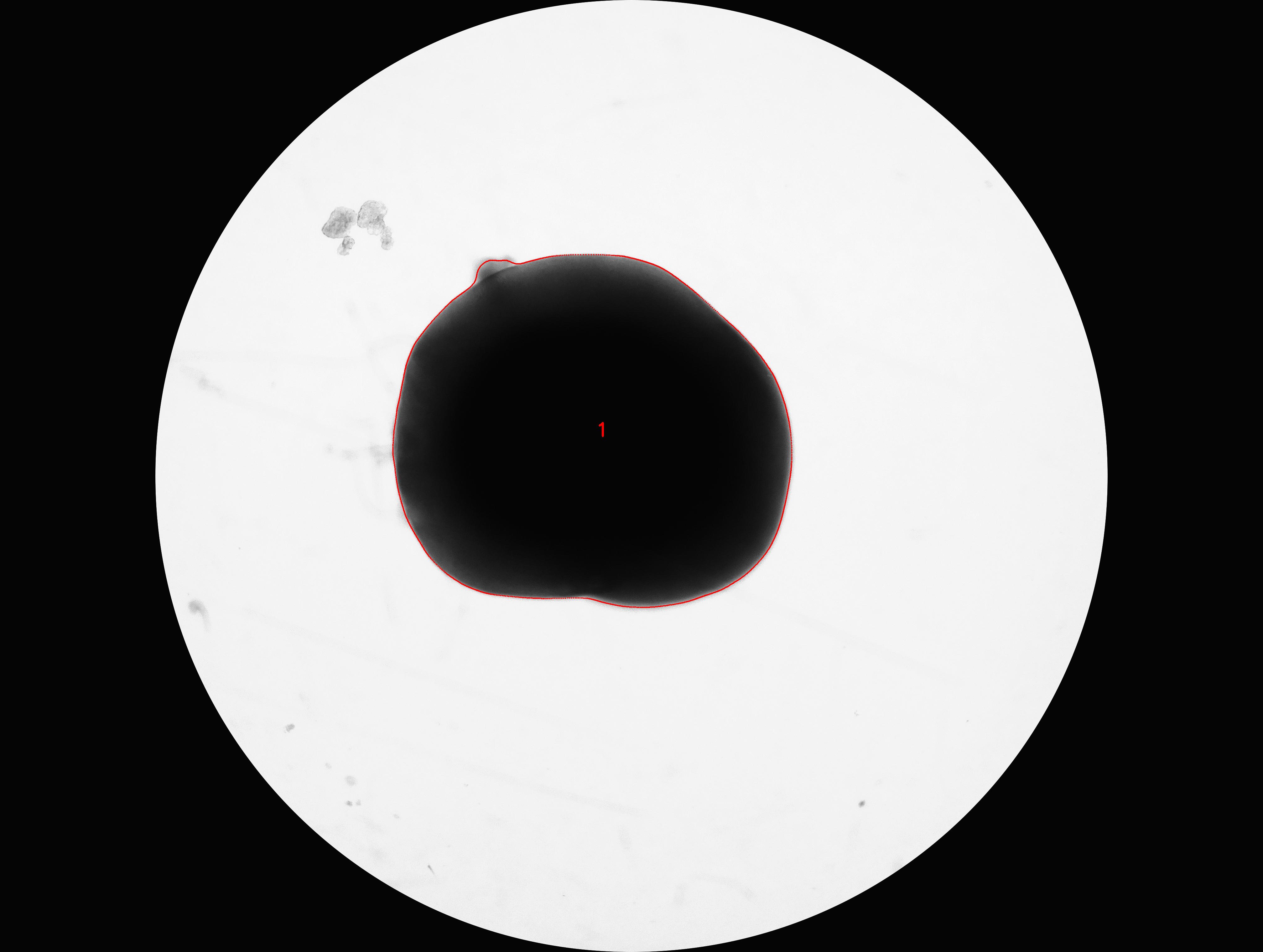

Supplement: Supplementary file 11 — Source data Fig. 3 [file 44319_2025_619_MOESM11_ESM.zip › Figure 3/C,D,F,G/Raw images_mask/OS_day90/MN 12C1 B C8 D90 2x/R_Day 90_0019.jpg]

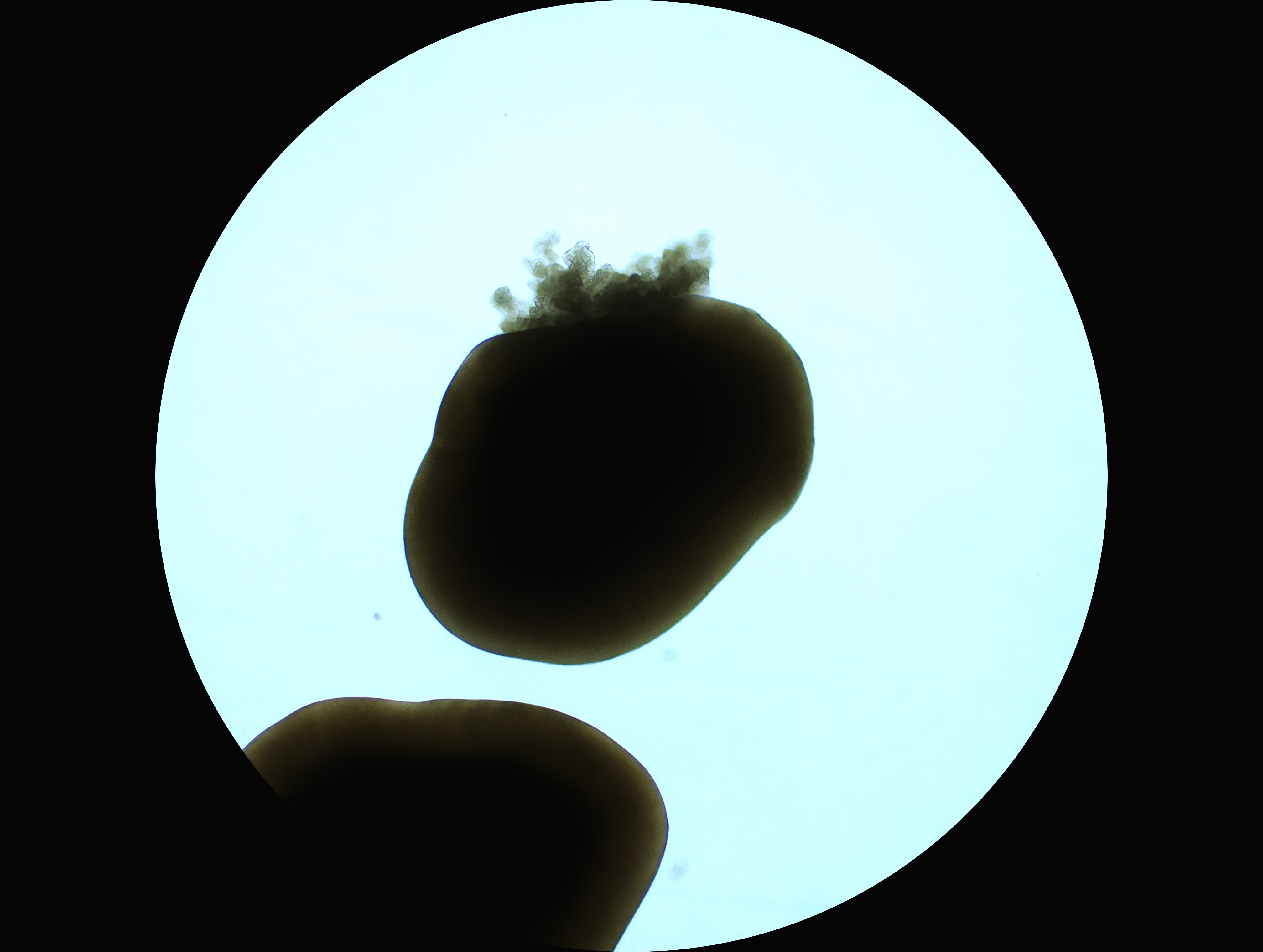

Supplement: Supplementary file 11 — Source data Fig. 3 [file 44319_2025_619_MOESM11_ESM.zip › Figure 3/C,D,F,G/Raw images_mask/OS_day90/MN 12C1 B C8 D90 2x/Day 90_0017.jpg]

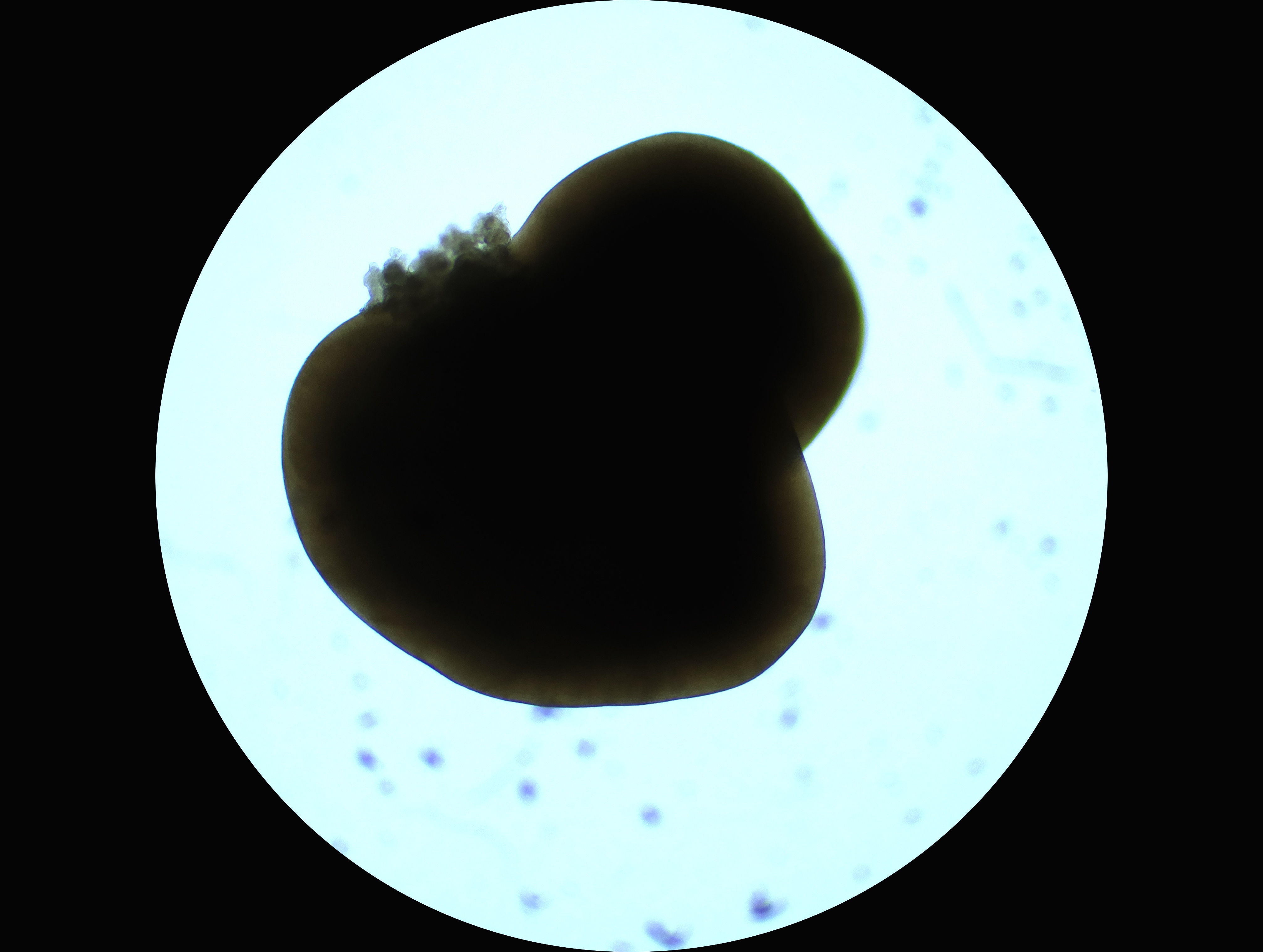

Supplement: Supplementary file 11 — Source data Fig. 3 [file 44319_2025_619_MOESM11_ESM.zip › Figure 3/C,D,F,G/Raw images_mask/OS_day90/MN 12C1 B C8 D90 2x/Day 90_0003.jpg]

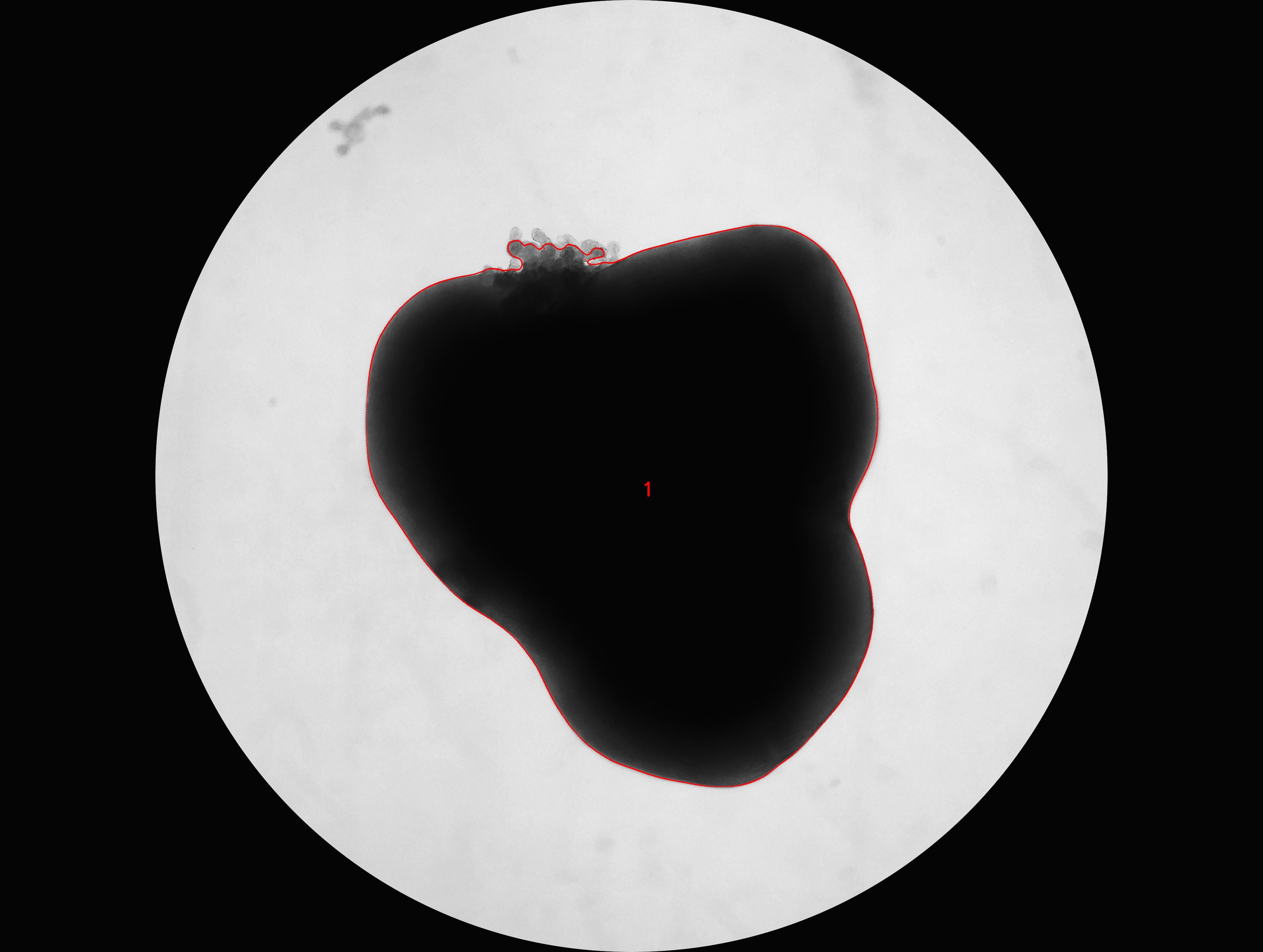

Supplement: Supplementary file 11 — Source data Fig. 3 [file 44319_2025_619_MOESM11_ESM.zip › Figure 3/C,D,F,G/Raw images_mask/OS_day90/MN 12C1 B C8 D90 2x/R_Day 90_0030.jpg]

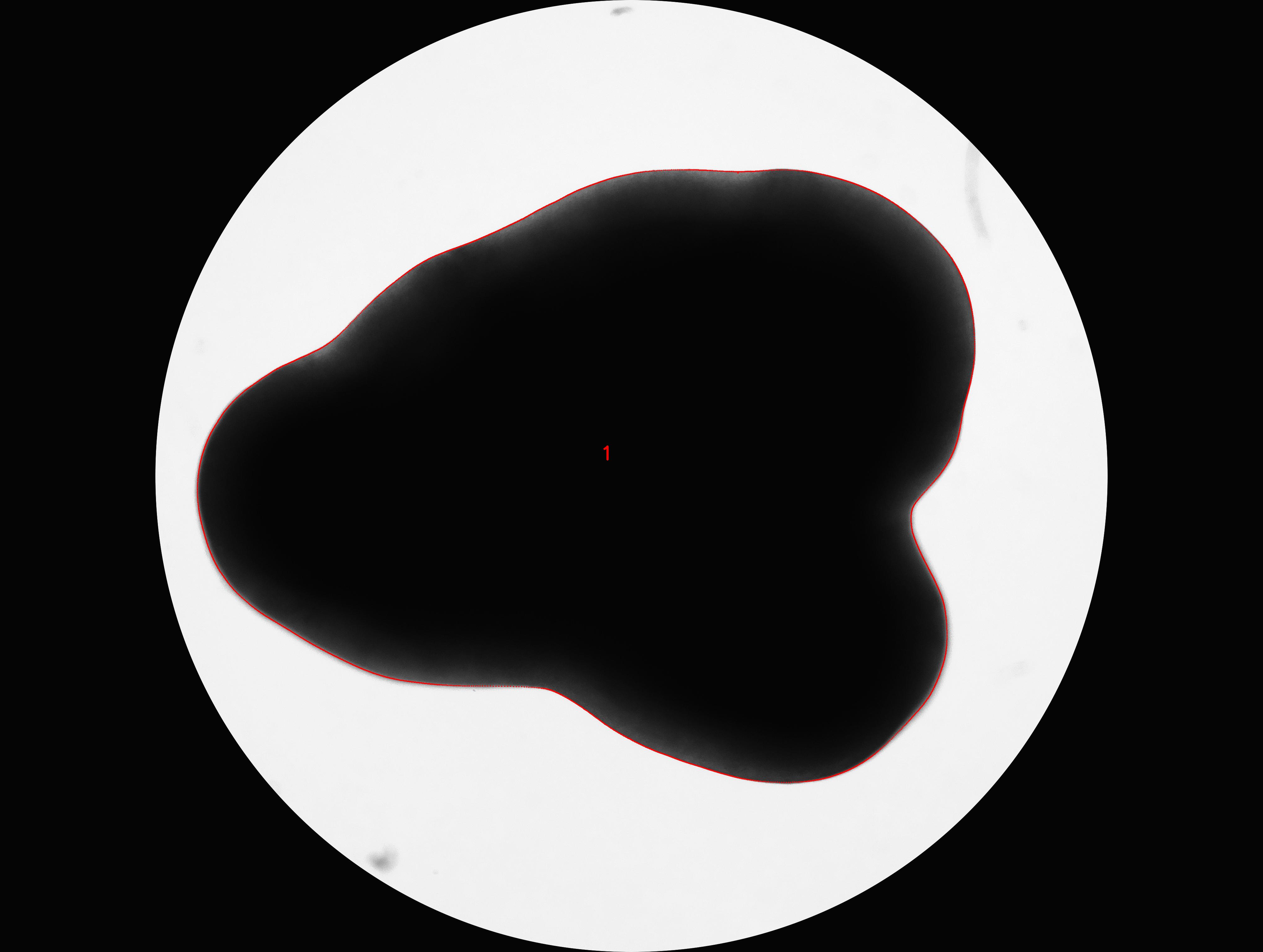

Supplement: Supplementary file 11 — Source data Fig. 3 [file 44319_2025_619_MOESM11_ESM.zip › Figure 3/C,D,F,G/Raw images_mask/OS_day90/MN 12C1 B C8 D90 2x/R_Day 90_0008.jpg]

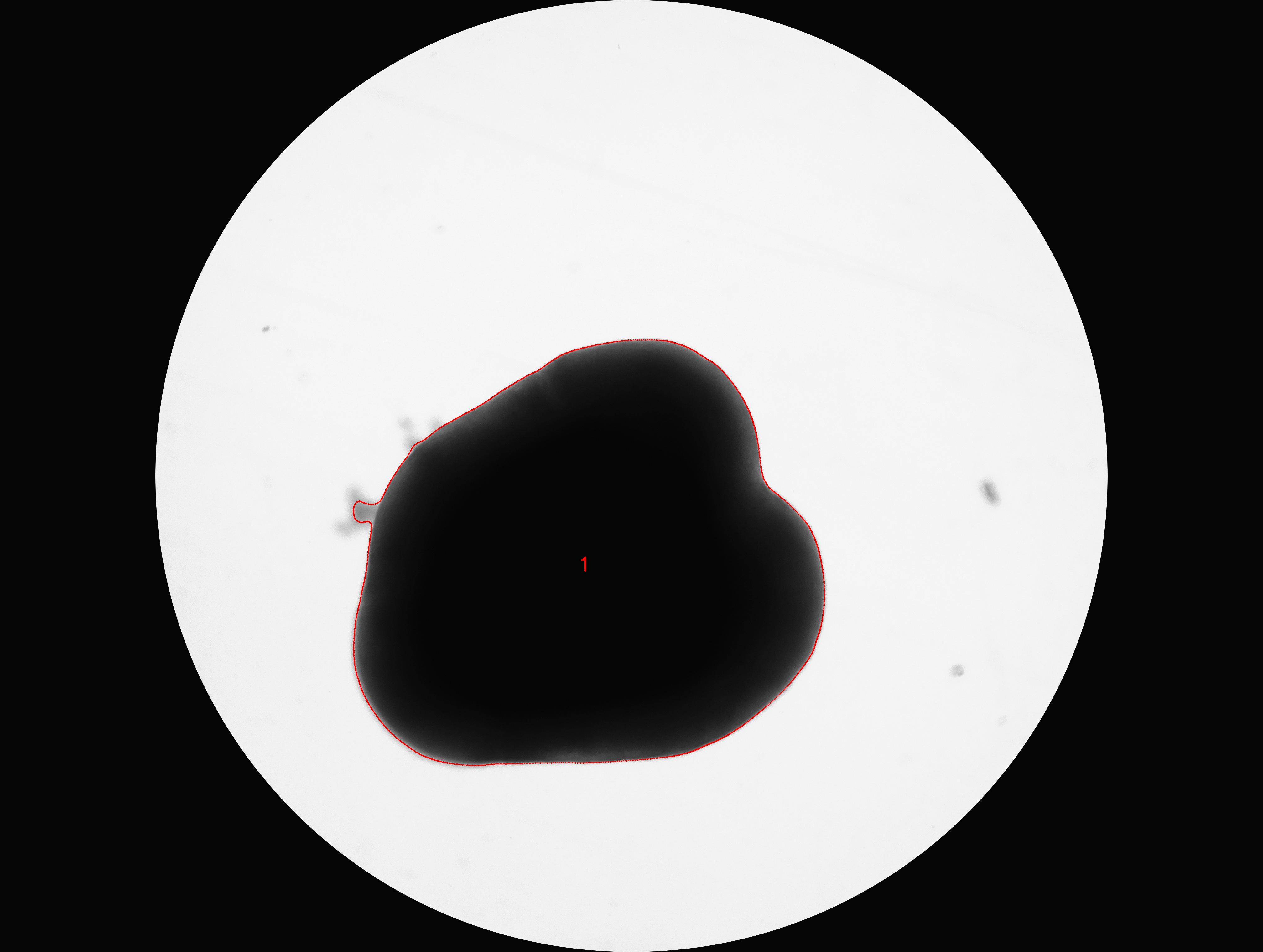

Supplement: Supplementary file 11 — Source data Fig. 3 [file 44319_2025_619_MOESM11_ESM.zip › Figure 3/C,D,F,G/Raw images_mask/OS_day90/MN 12C1 B C8 D90 2x/R_Day 90_0020.jpg]

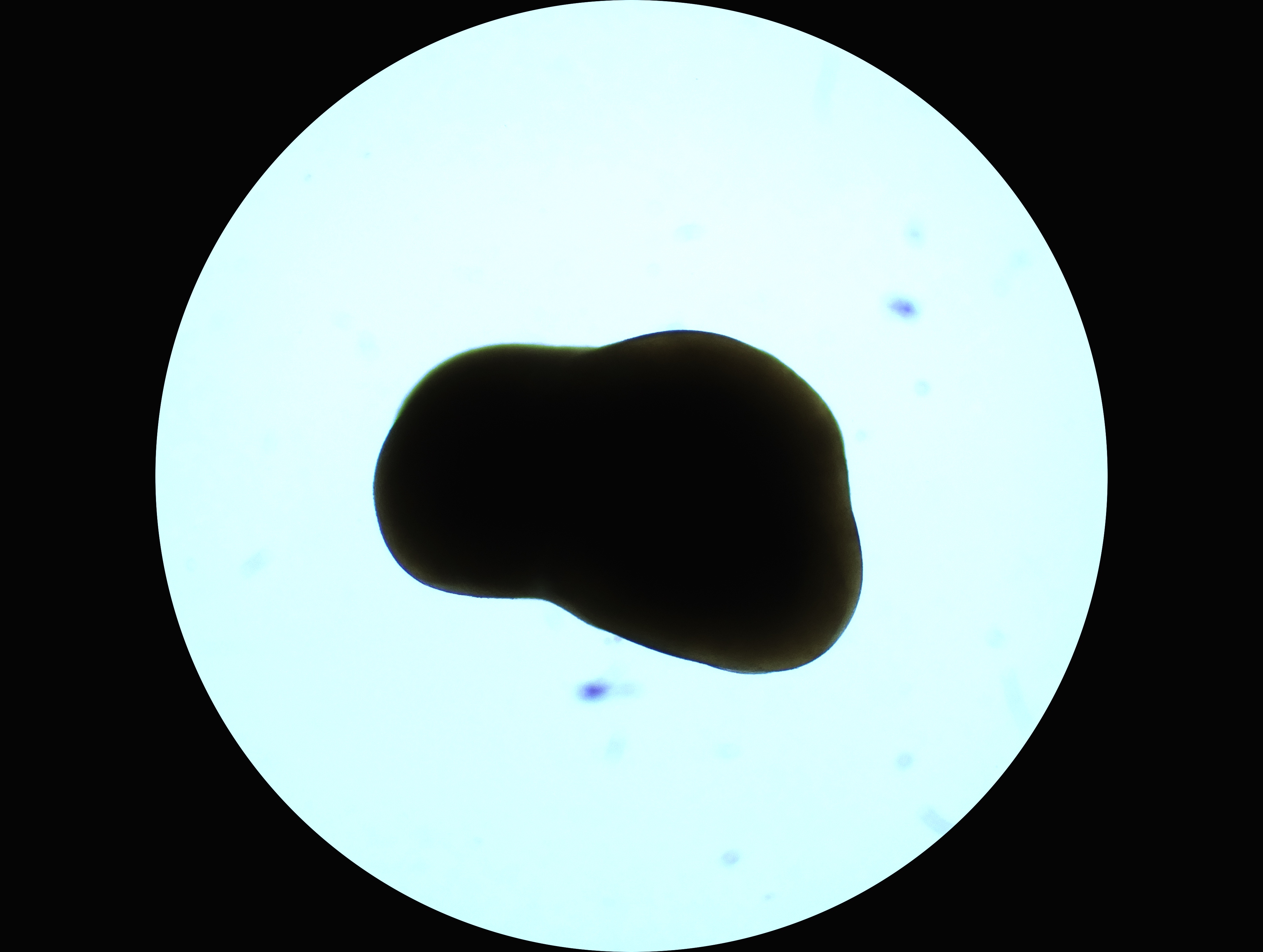

Supplement: Supplementary file 11 — Source data Fig. 3 [file 44319_2025_619_MOESM11_ESM.zip › Figure 3/C,D,F,G/Raw images_mask/OS_day90/MN 12C1 B C8 D90 2x/Day 90_0007.jpg]

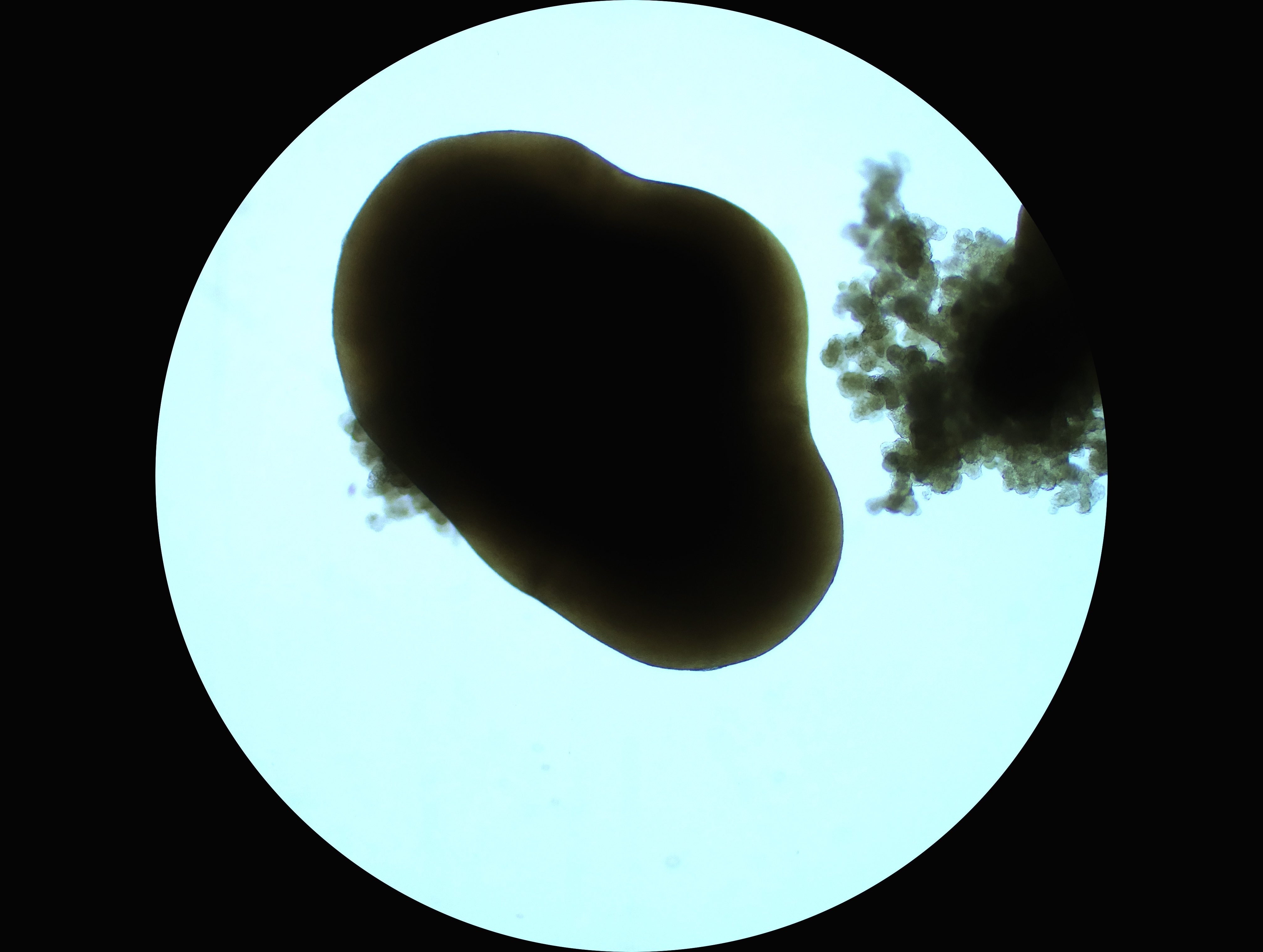

Supplement: Supplementary file 11 — Source data Fig. 3 [file 44319_2025_619_MOESM11_ESM.zip › Figure 3/C,D,F,G/Raw images_mask/OS_day90/MN 12C1 B C8 D90 2x/Day 90_0012.jpg]

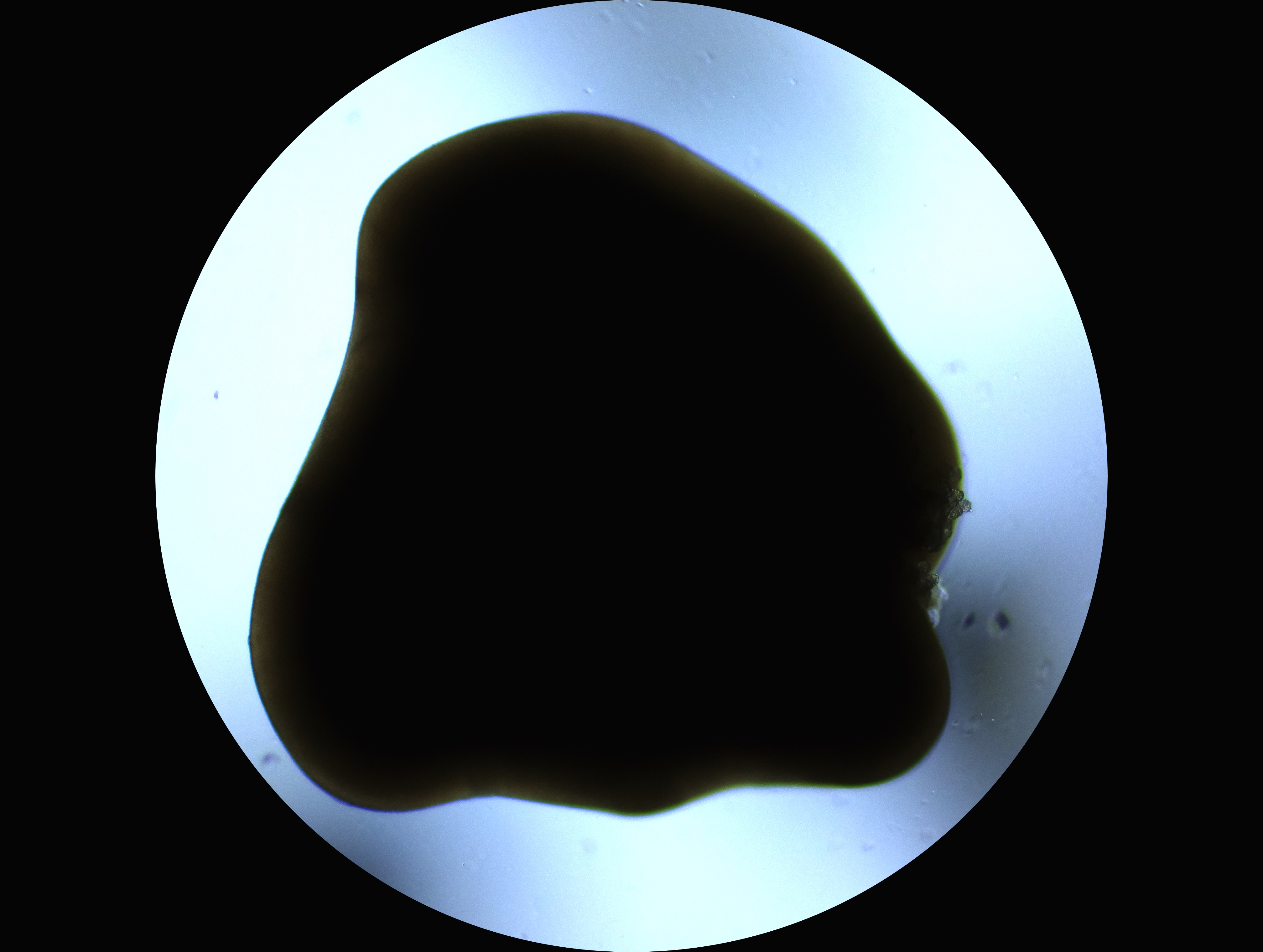

Supplement: Supplementary file 11 — Source data Fig. 3 [file 44319_2025_619_MOESM11_ESM.zip › Figure 3/C,D,F,G/Raw images_mask/OS_day90/MN 12C1 B C8 D90 2x/Day 90_0006.jpg]

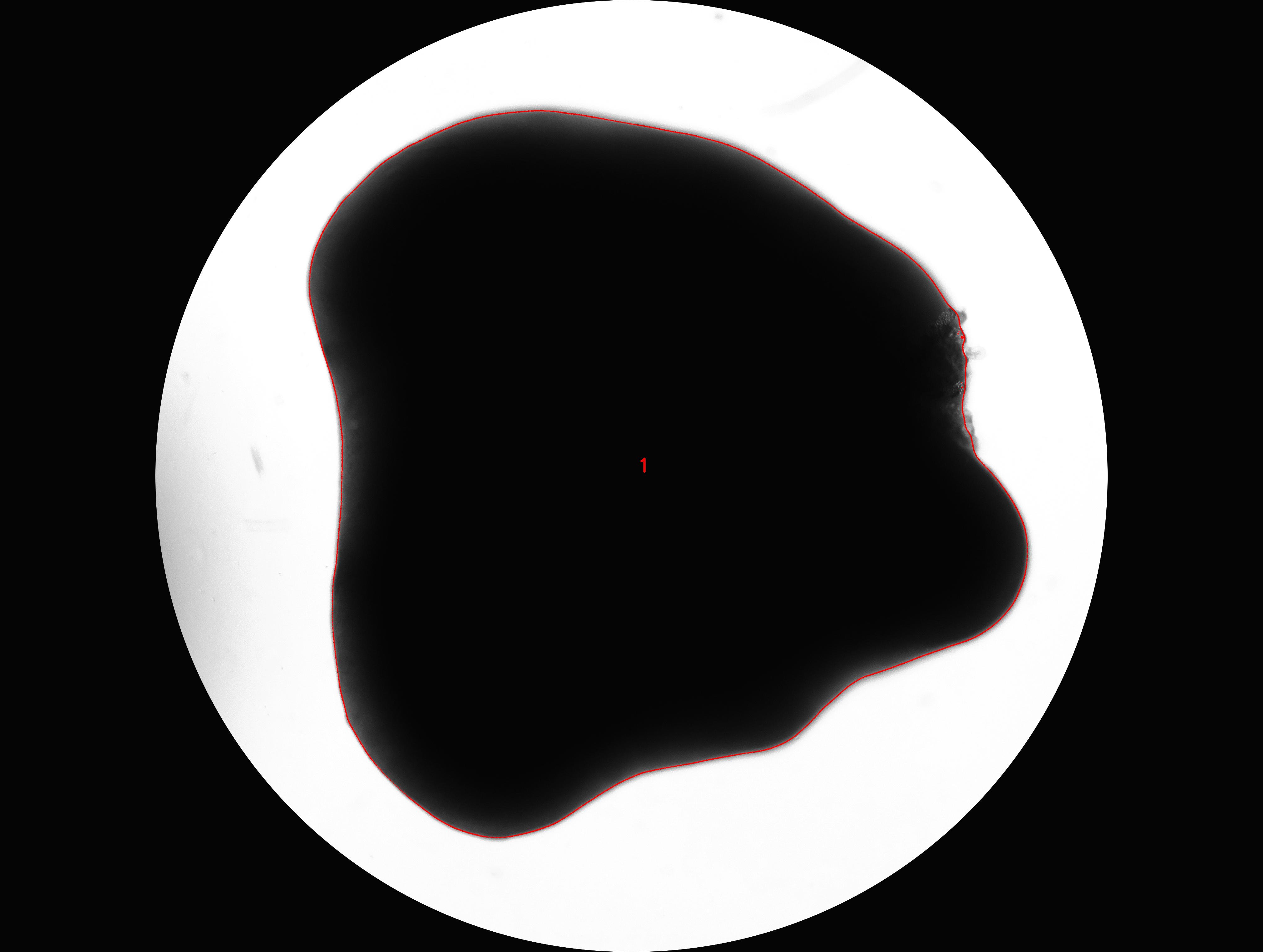

Supplement: Supplementary file 11 — Source data Fig. 3 [file 44319_2025_619_MOESM11_ESM.zip › Figure 3/C,D,F,G/Raw images_mask/OS_day90/MN 12C1 B C8 D90 2x/R_Day 90_0035.jpg]

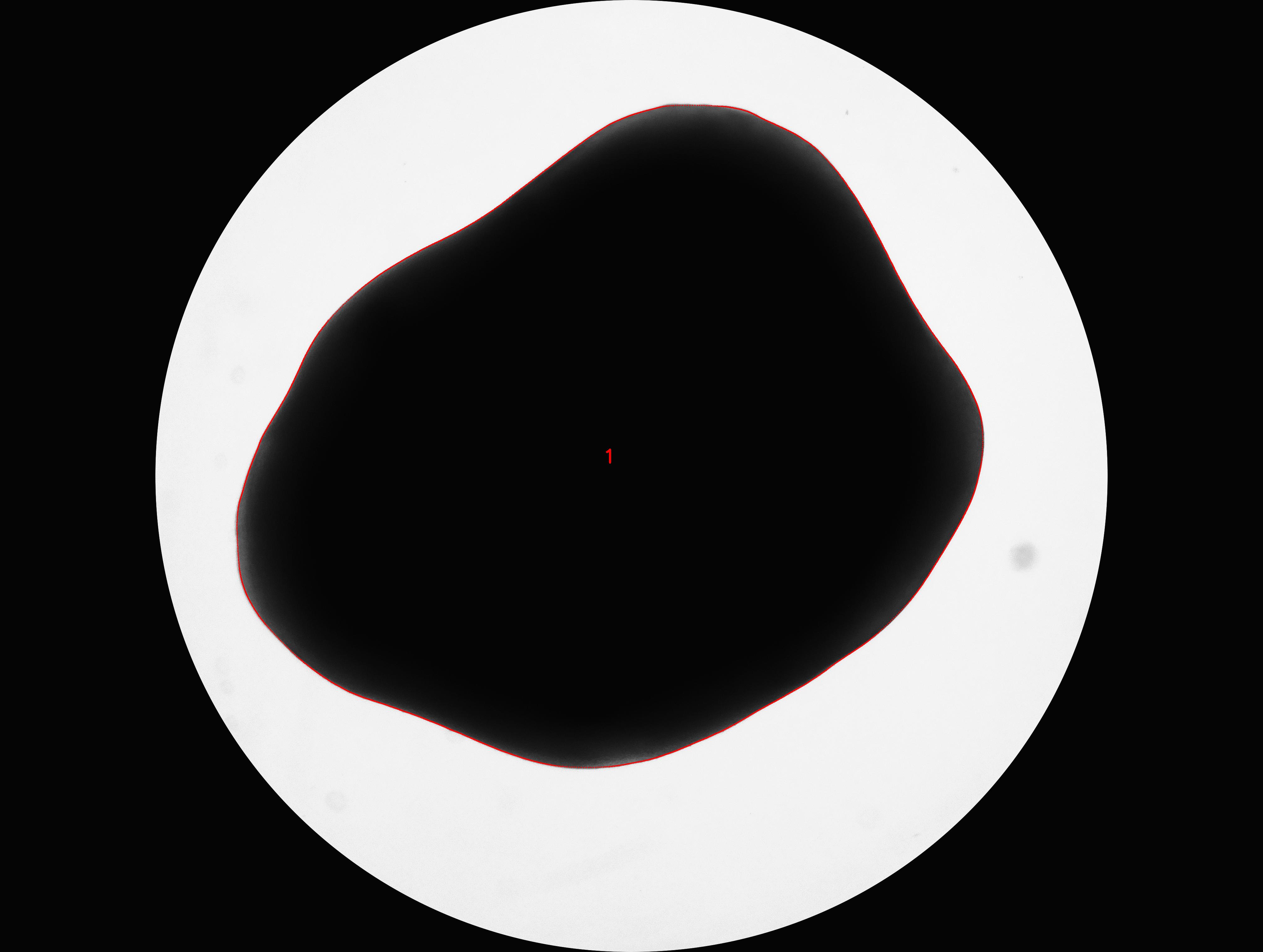

Supplement: Supplementary file 11 — Source data Fig. 3 [file 44319_2025_619_MOESM11_ESM.zip › Figure 3/C,D,F,G/Raw images_mask/OS_day90/MN 12C1 B C8 D90 2x/R_Day 90_0021.jpg]

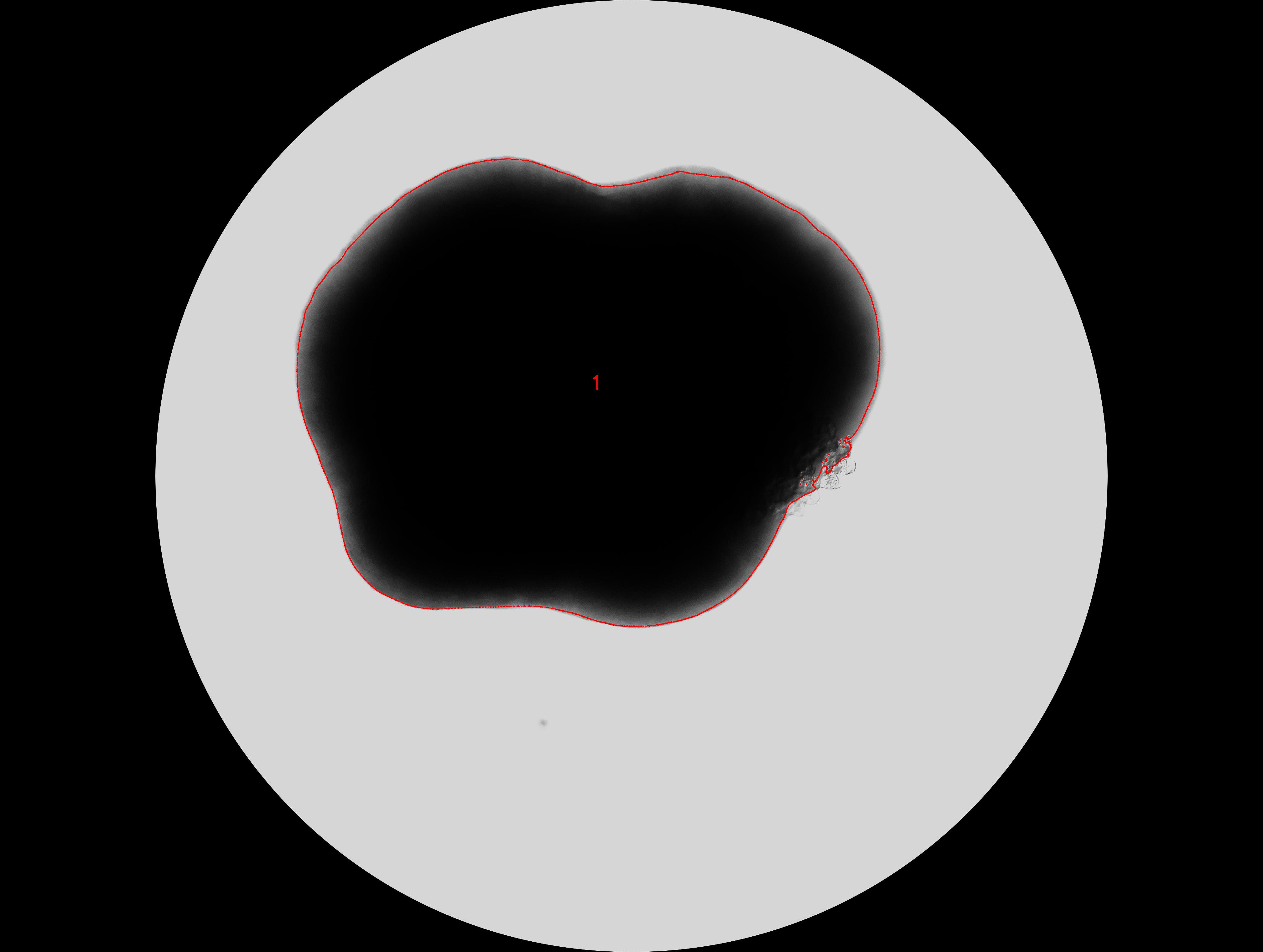

Supplement: Supplementary file 11 — Source data Fig. 3 [file 44319_2025_619_MOESM11_ESM.zip › Figure 3/C,D,F,G/Raw images_mask/OS_day90/MN 12C1 B C8 D90 2x/R_Day 90_0037.jpg]

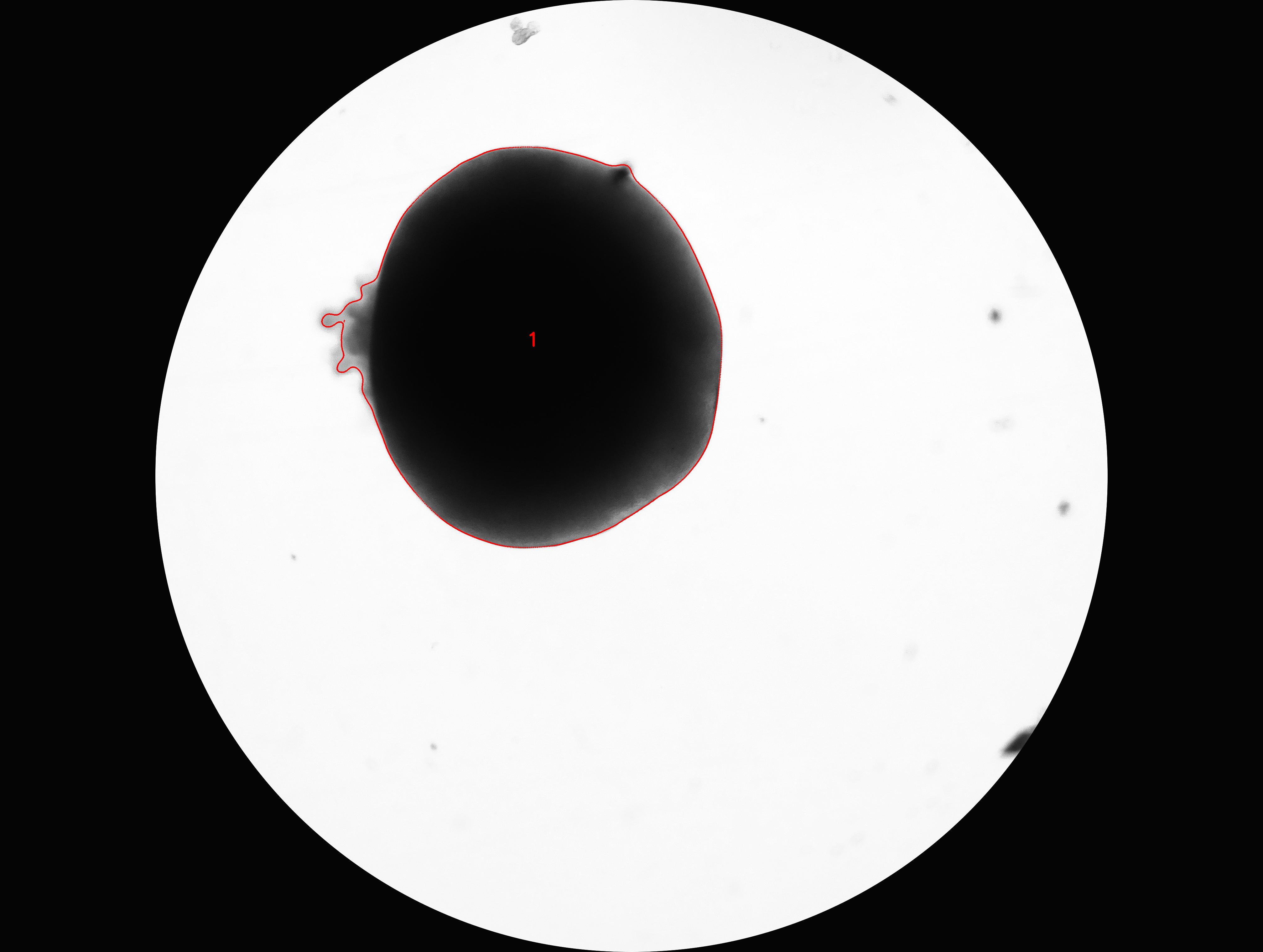

Supplement: Supplementary file 11 — Source data Fig. 3 [file 44319_2025_619_MOESM11_ESM.zip › Figure 3/C,D,F,G/Raw images_mask/OS_day90/MN 12C1 B C8 D90 2x/R_Day 90_0023.jpg]

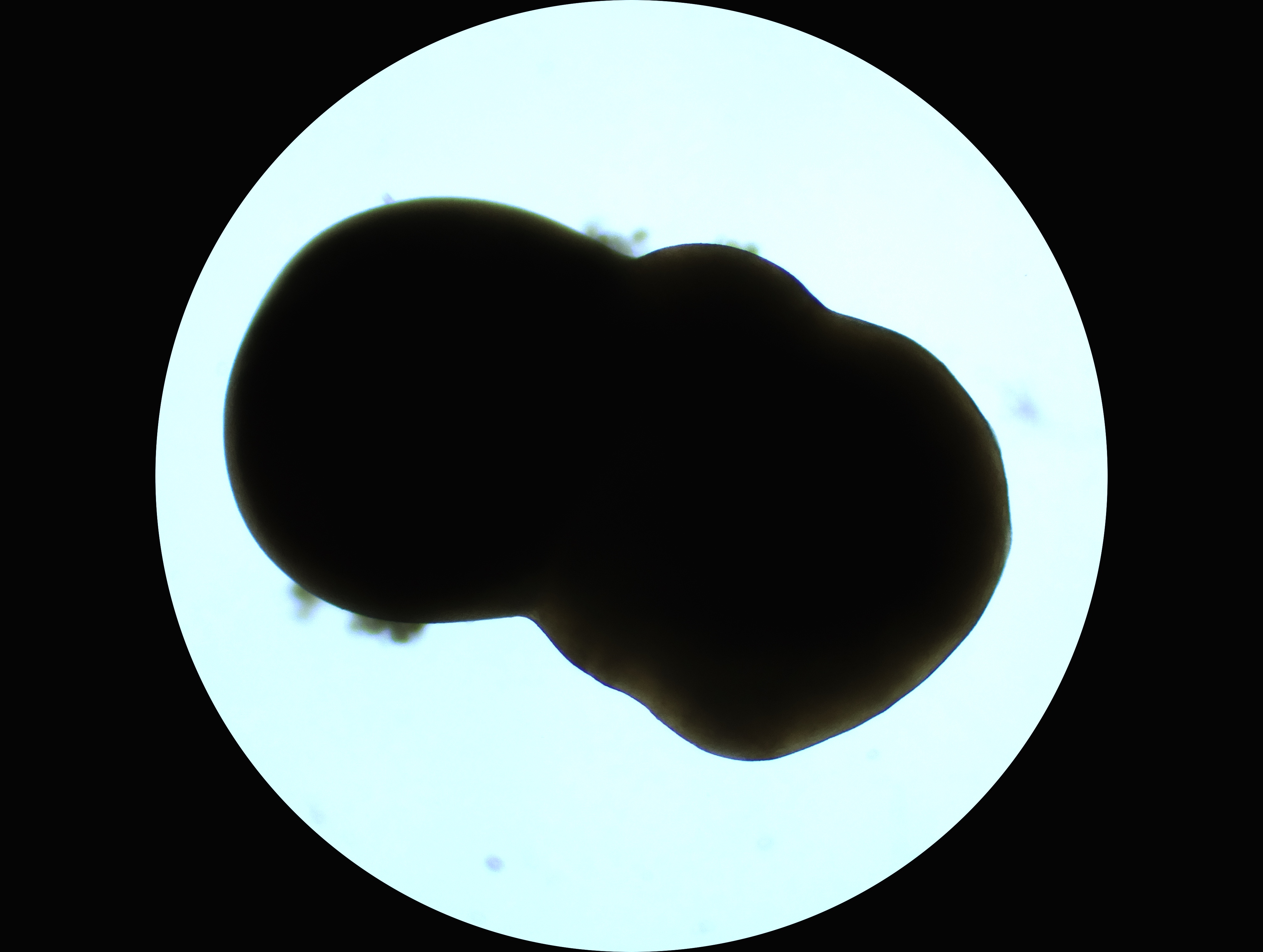

Supplement: Supplementary file 11 — Source data Fig. 3 [file 44319_2025_619_MOESM11_ESM.zip › Figure 3/C,D,F,G/Raw images_mask/OS_day90/MN 12C1 B C8 D90 2x/Day 90_0010.jpg]

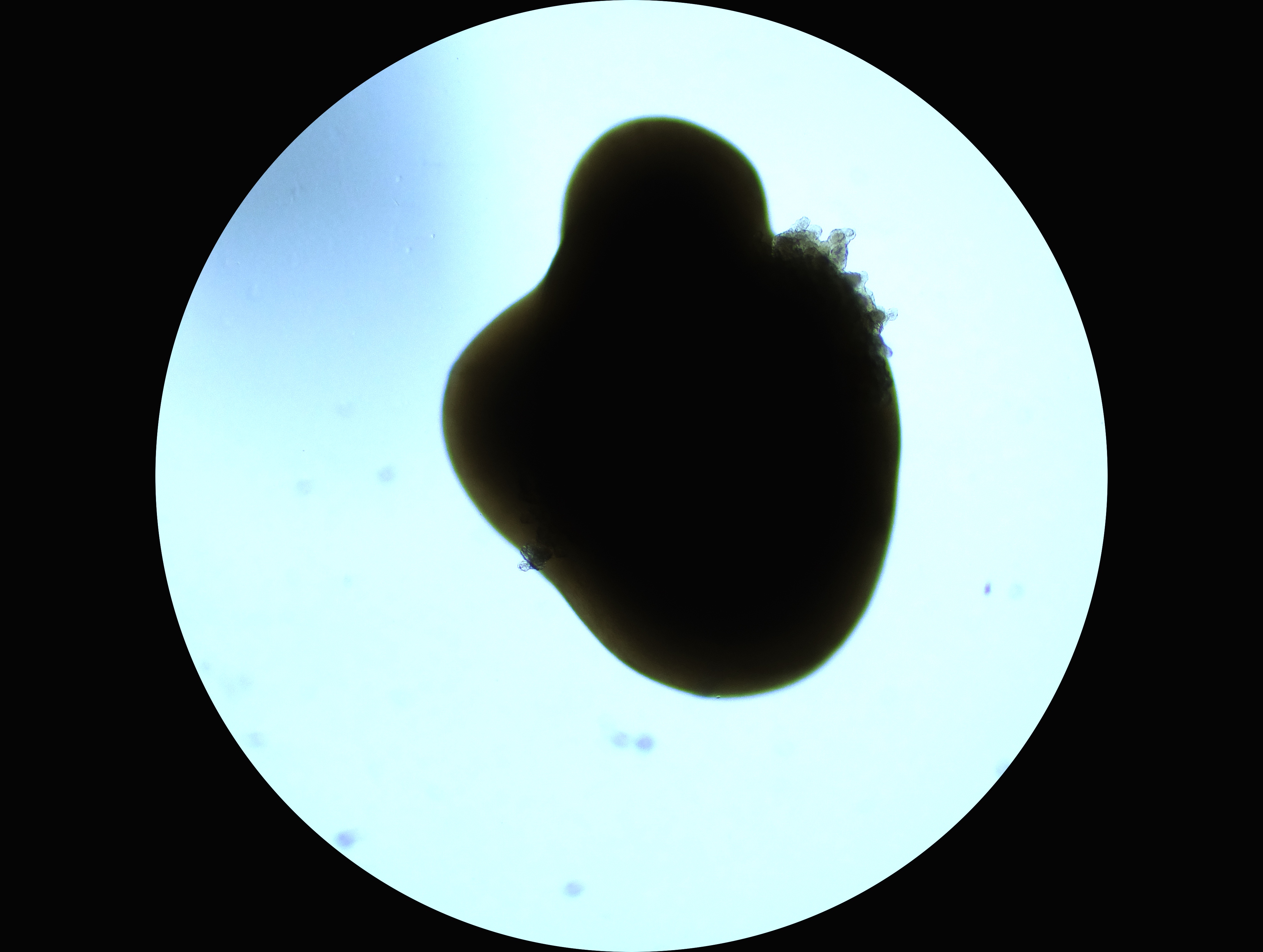

Supplement: Supplementary file 11 — Source data Fig. 3 [file 44319_2025_619_MOESM11_ESM.zip › Figure 3/C,D,F,G/Raw images_mask/OS_day90/MN 12C1 B C8 D90 2x/Day 90_0005.jpg]

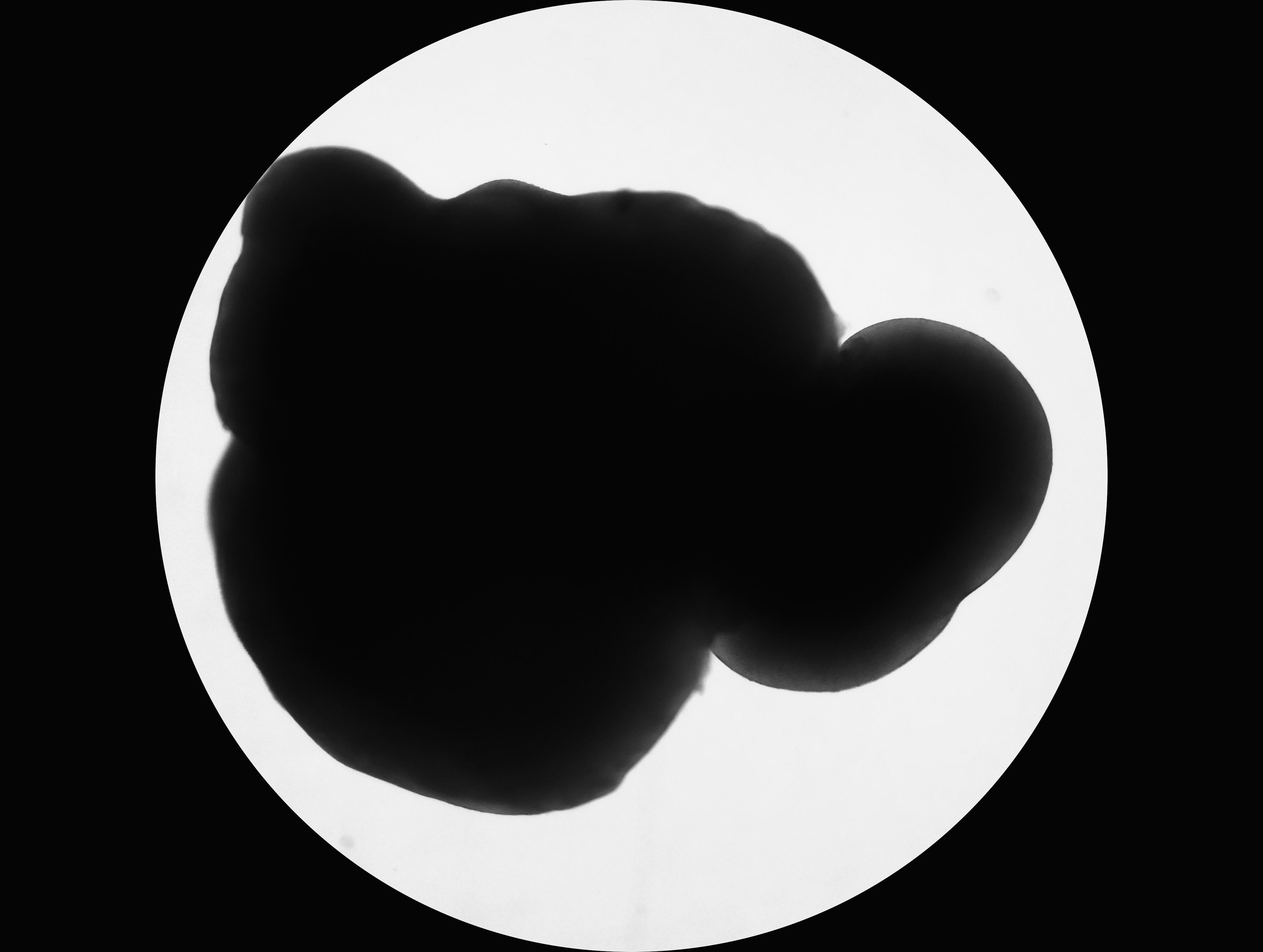

Supplement: Supplementary file 11 — Source data Fig. 3 [file 44319_2025_619_MOESM11_ESM.zip › Figure 3/C,D,F,G/Raw images_mask/OS_day90/MN 12C1 B C8 D90 2x/R_Day 90_0022.jpg]

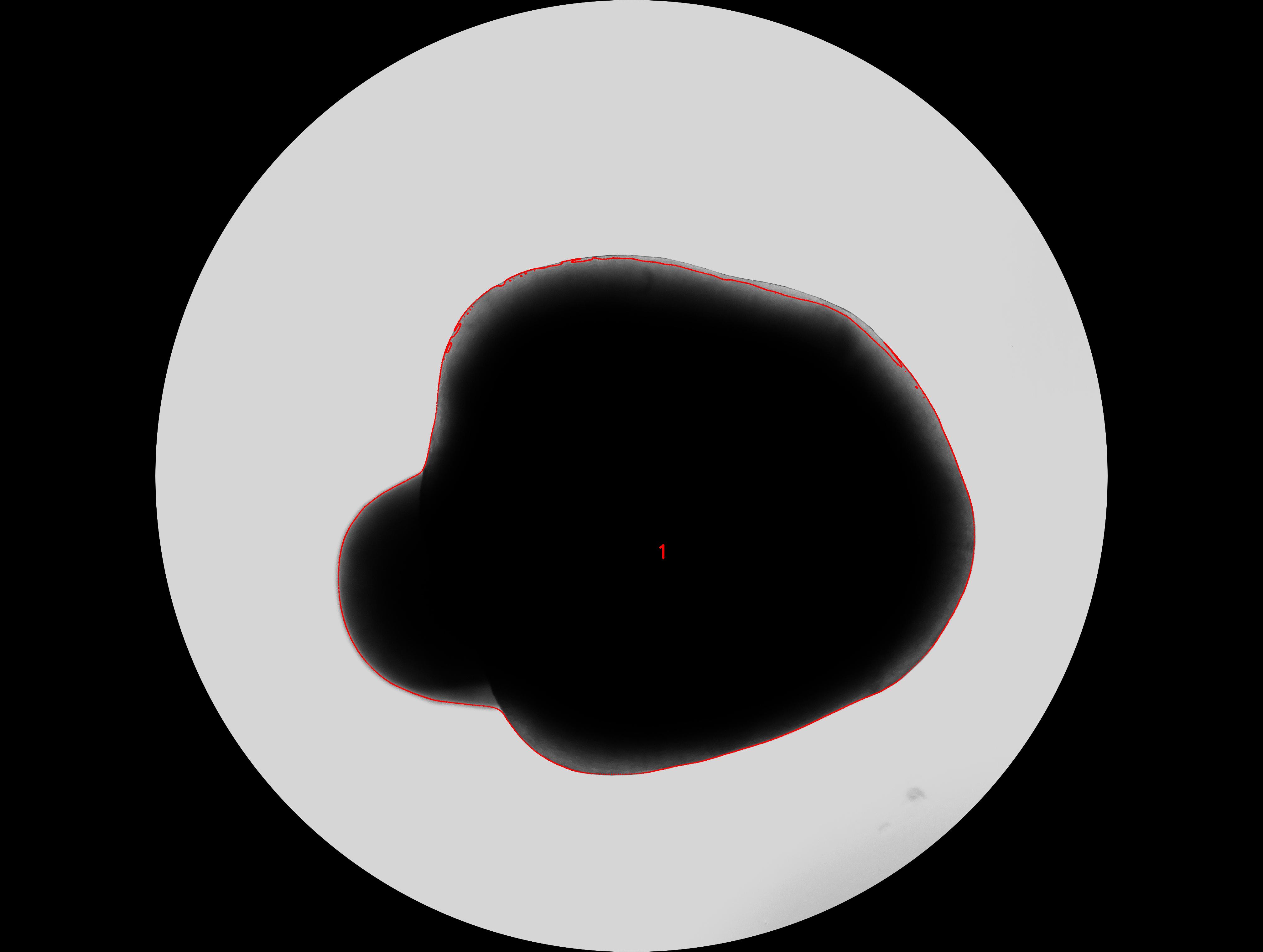

Supplement: Supplementary file 11 — Source data Fig. 3 [file 44319_2025_619_MOESM11_ESM.zip › Figure 3/C,D,F,G/Raw images_mask/OS_day90/MN 12C1 B C8 D90 2x/R_Day 90_0042.jpg]

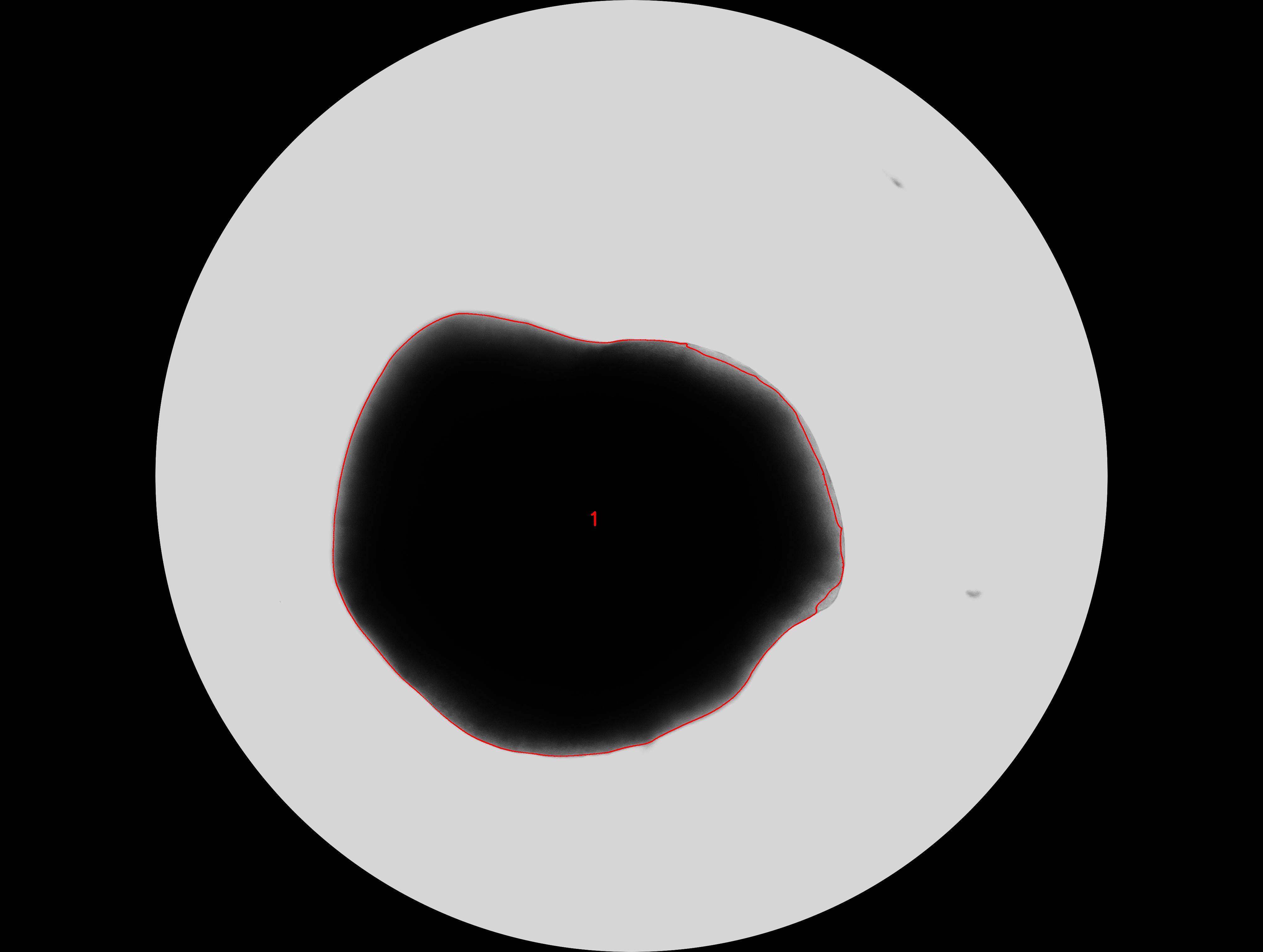

Supplement: Supplementary file 11 — Source data Fig. 3 [file 44319_2025_619_MOESM11_ESM.zip › Figure 3/C,D,F,G/Raw images_mask/OS_day90/MN 12C1 B C8 D90 2x/R_Day 90_0040.jpg]

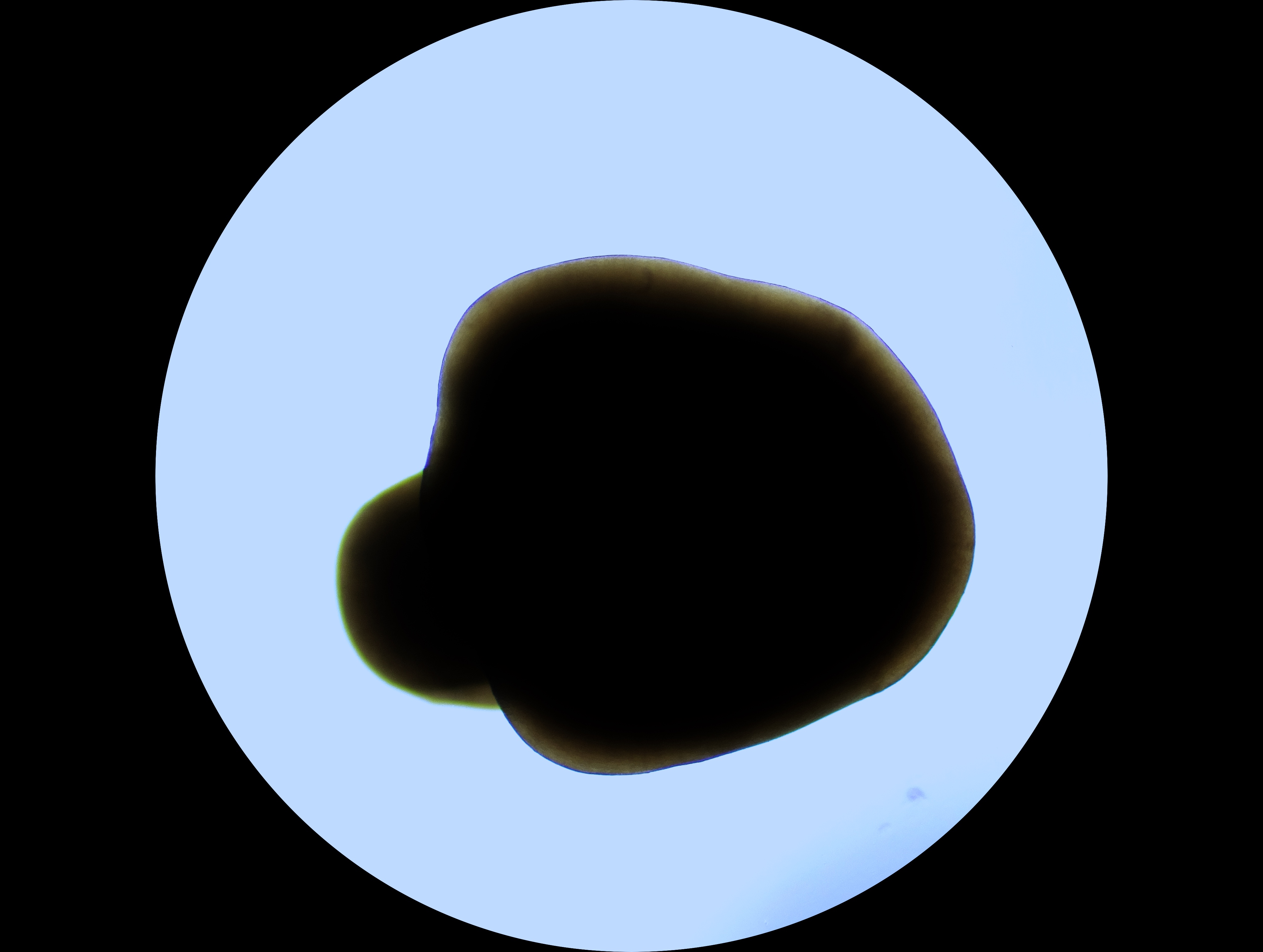

Supplement: Supplementary file 11 — Source data Fig. 3 [file 44319_2025_619_MOESM11_ESM.zip › Figure 3/C,D,F,G/Raw images_mask/OS_day90/MN 12C1 B C8 D90 2x/Day 90_0042.jpg]

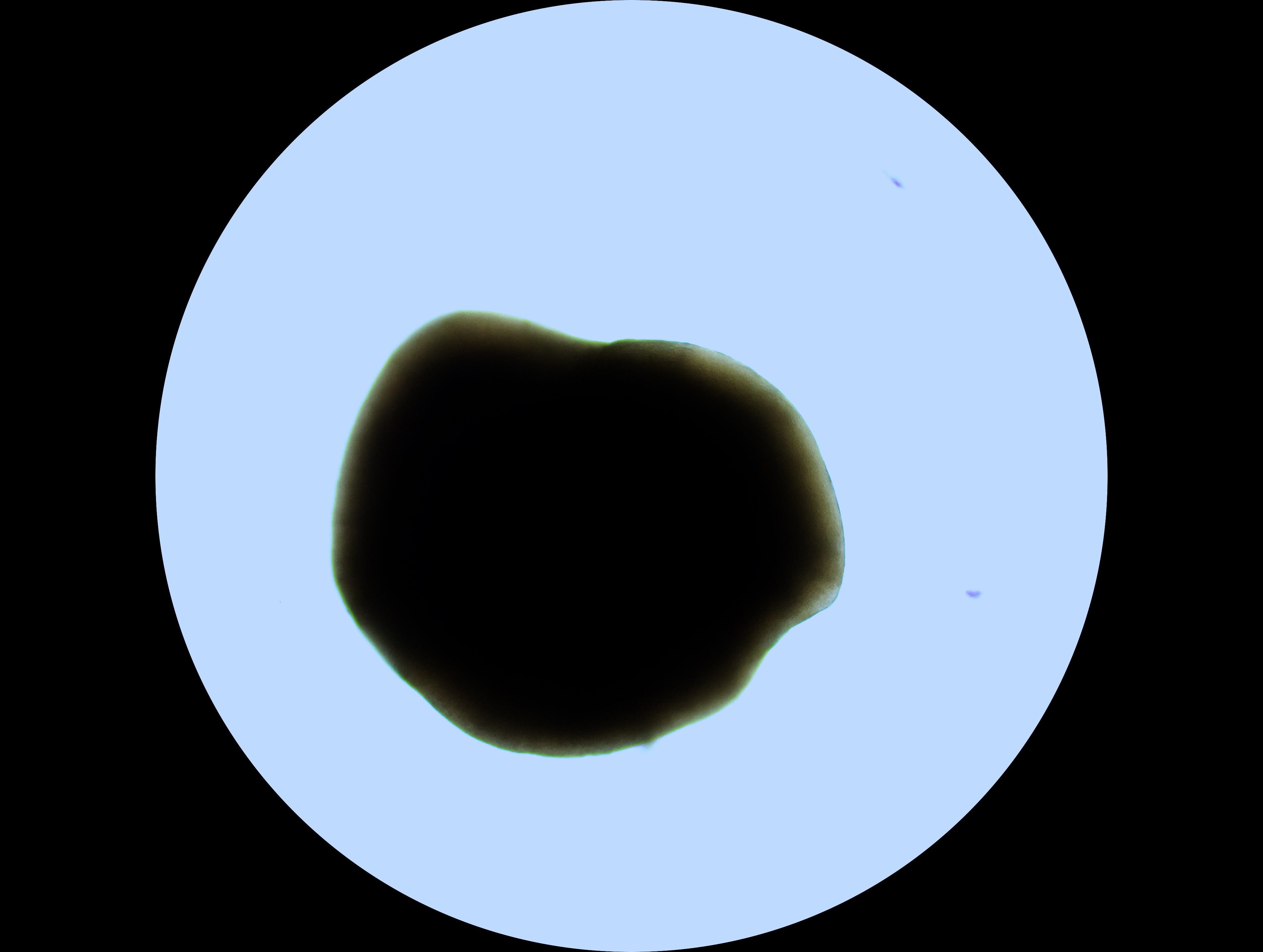

Supplement: Supplementary file 11 — Source data Fig. 3 [file 44319_2025_619_MOESM11_ESM.zip › Figure 3/C,D,F,G/Raw images_mask/OS_day90/MN 12C1 B C8 D90 2x/Day 90_0040.jpg]

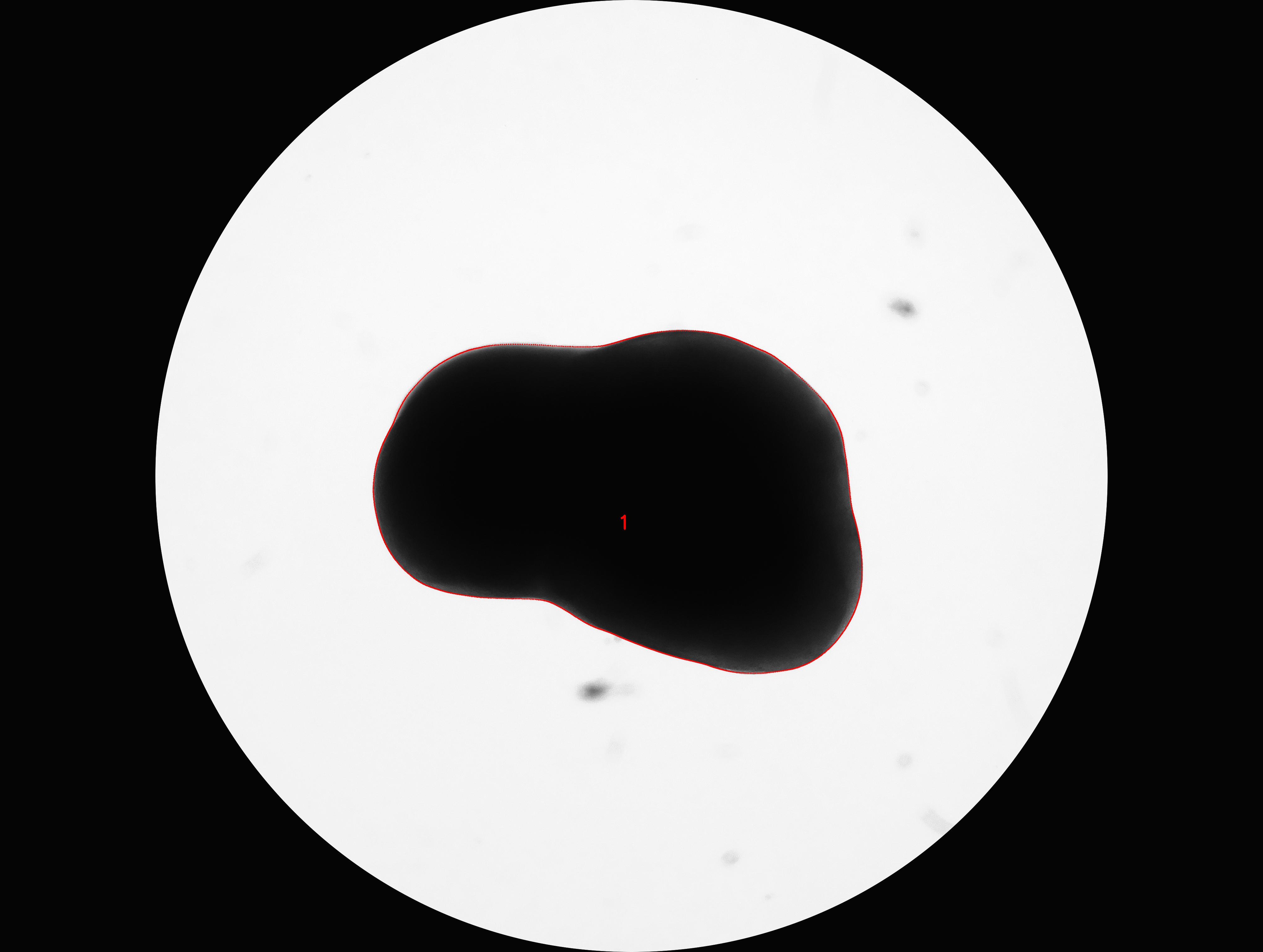

Supplement: Supplementary file 11 — Source data Fig. 3 [file 44319_2025_619_MOESM11_ESM.zip › Figure 3/C,D,F,G/Raw images_mask/OS_day90/MN 12C1 B C8 D90 2x/R_Day 90_0007.jpg]

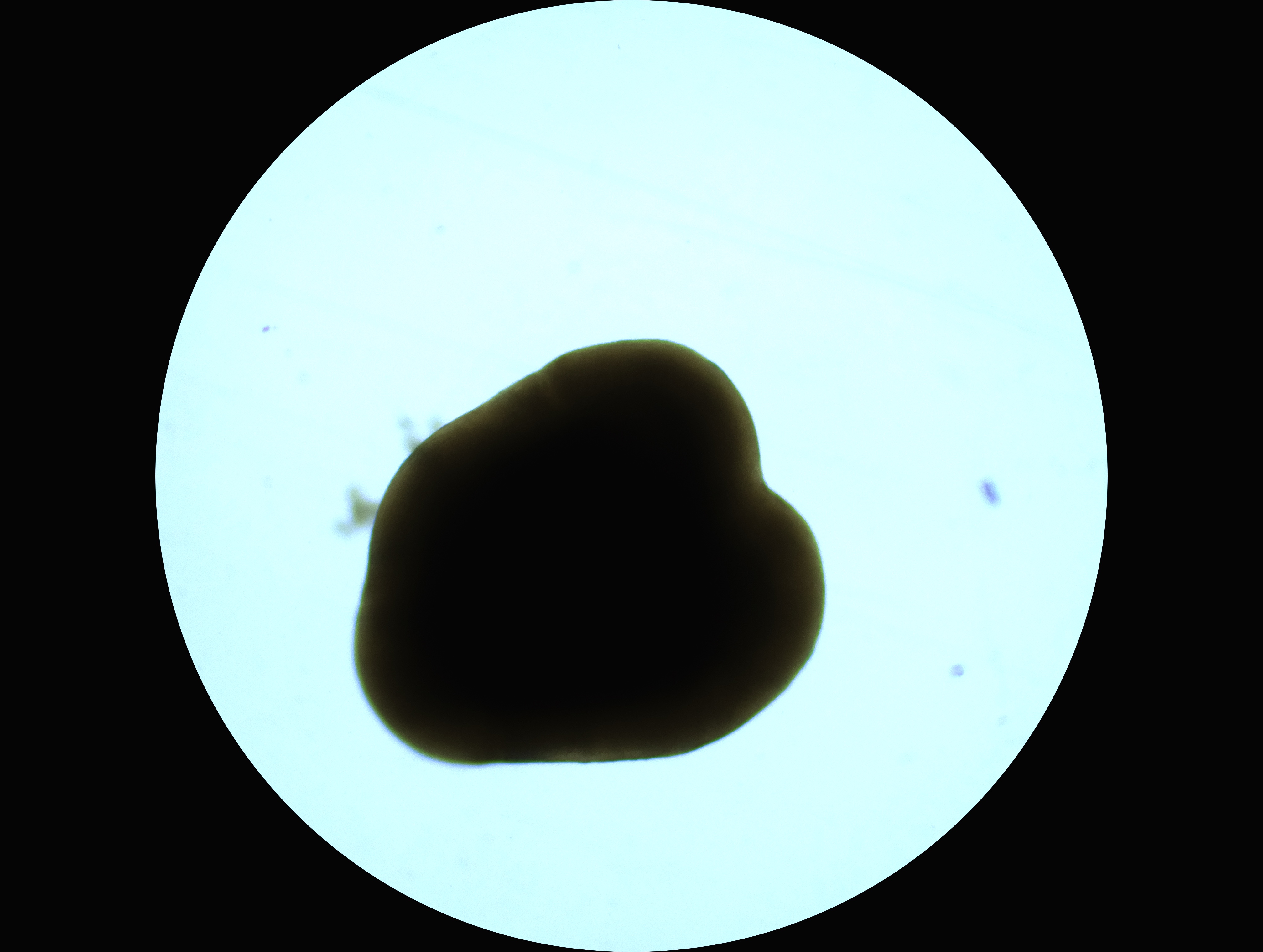

Supplement: Supplementary file 11 — Source data Fig. 3 [file 44319_2025_619_MOESM11_ESM.zip › Figure 3/C,D,F,G/Raw images_mask/OS_day90/MN 12C1 B C8 D90 2x/Day 90_0020.jpg]

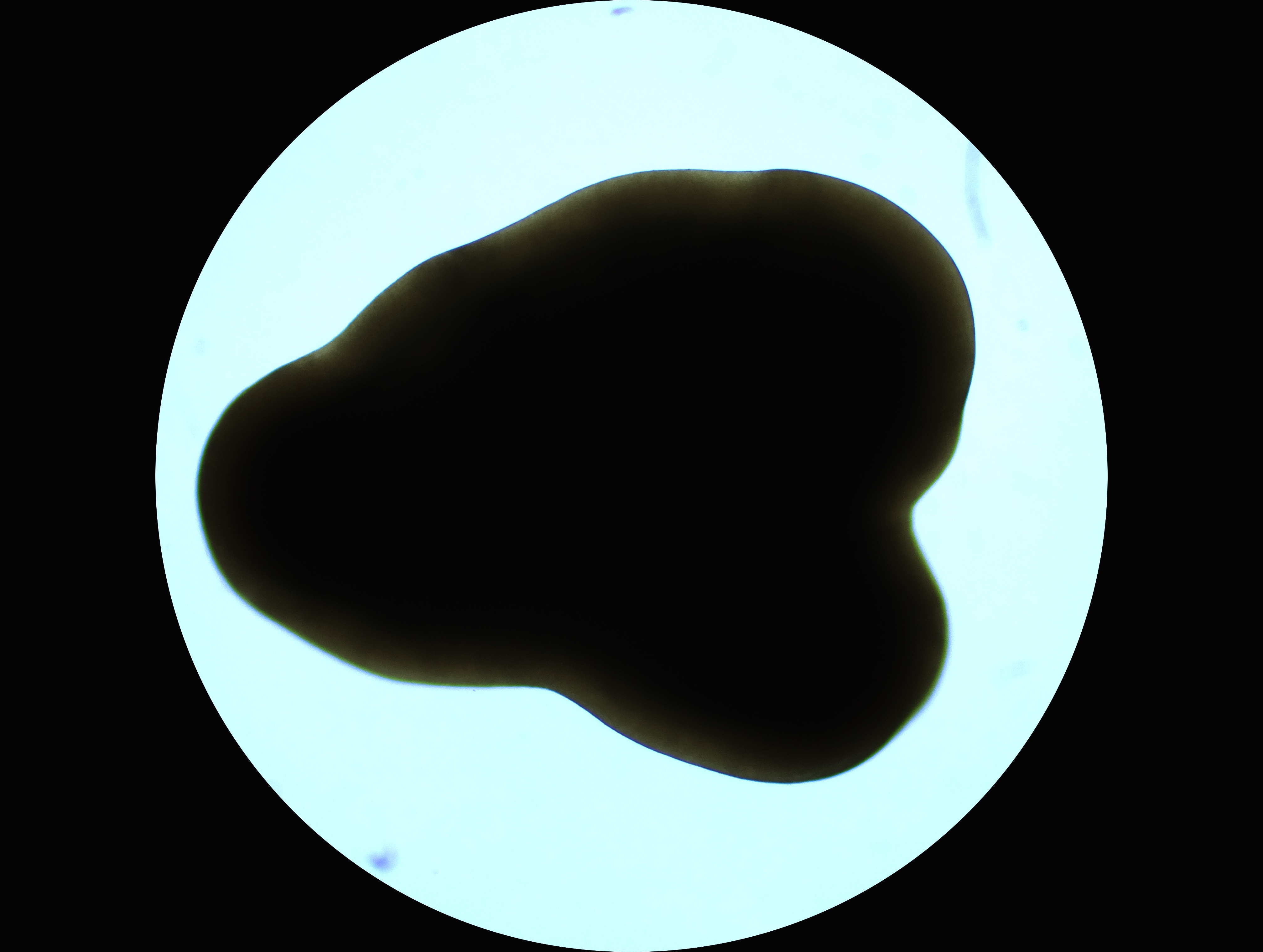

Supplement: Supplementary file 11 — Source data Fig. 3 [file 44319_2025_619_MOESM11_ESM.zip › Figure 3/C,D,F,G/Raw images_mask/OS_day90/MN 12C1 B C8 D90 2x/Day 90_0008.jpg]

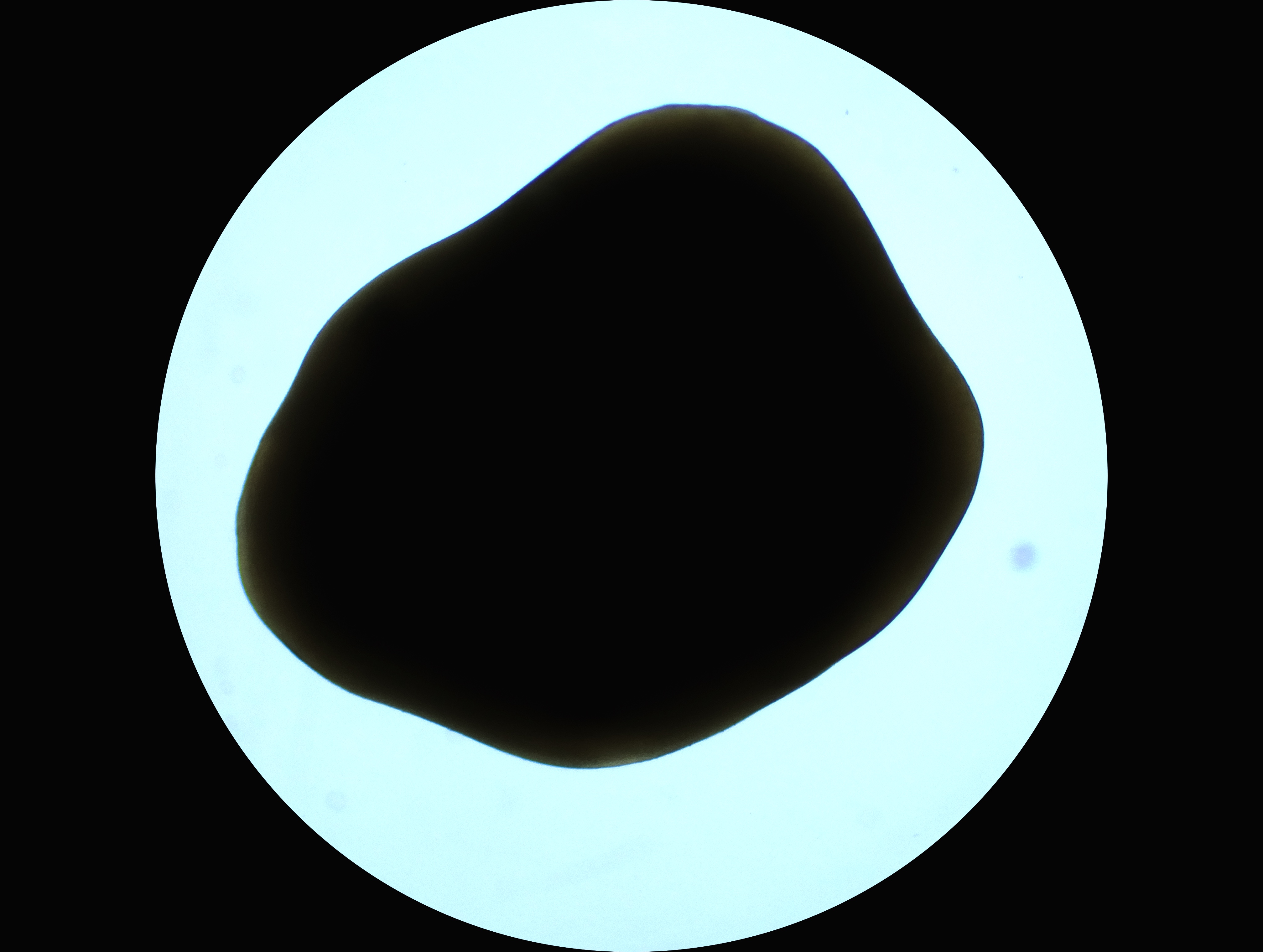

Supplement: Supplementary file 11 — Source data Fig. 3 [file 44319_2025_619_MOESM11_ESM.zip › Figure 3/C,D,F,G/Raw images_mask/OS_day90/MN 12C1 B C8 D90 2x/Day 90_0021.jpg]

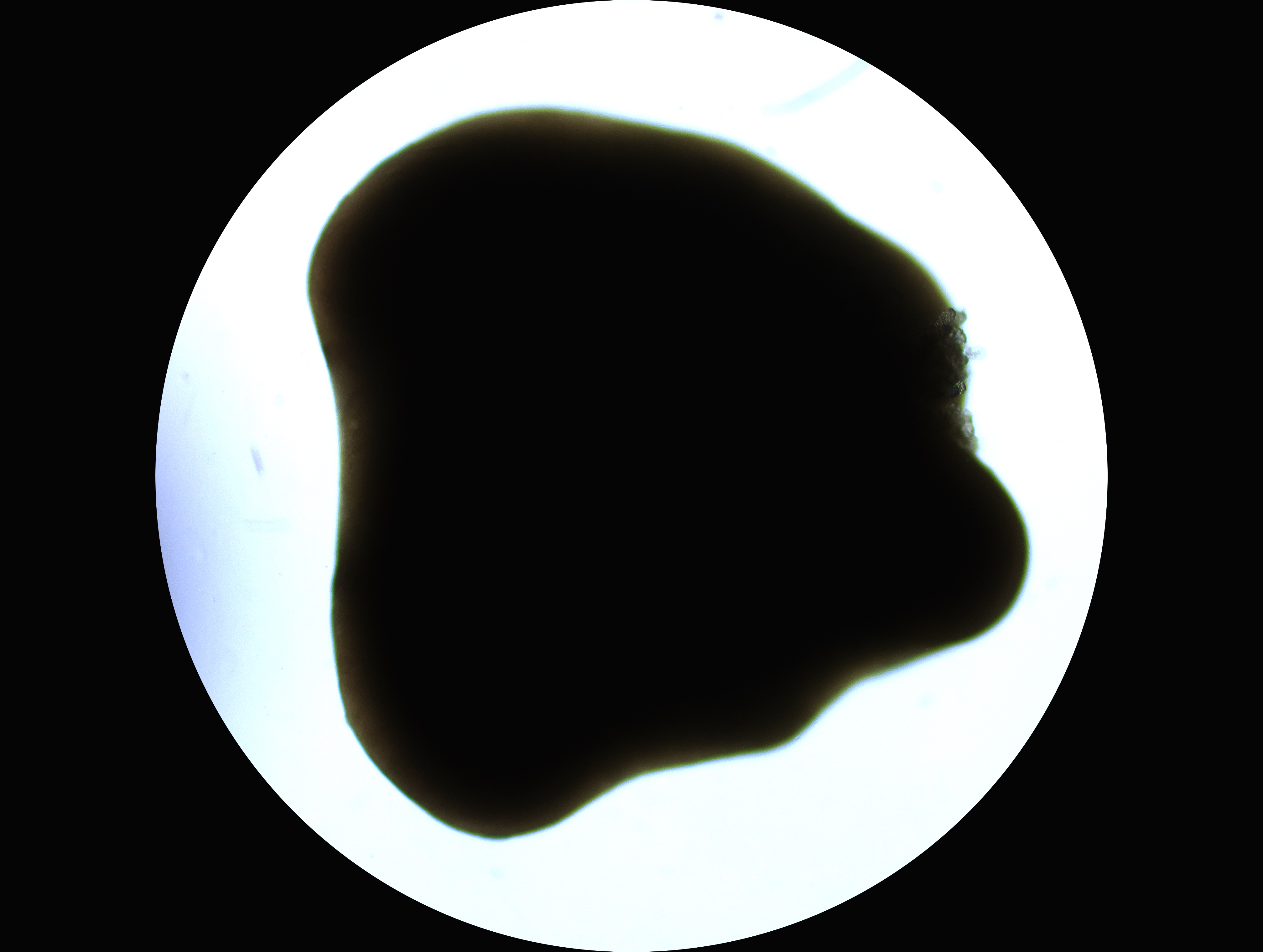

Supplement: Supplementary file 11 — Source data Fig. 3 [file 44319_2025_619_MOESM11_ESM.zip › Figure 3/C,D,F,G/Raw images_mask/OS_day90/MN 12C1 B C8 D90 2x/Day 90_0035.jpg]

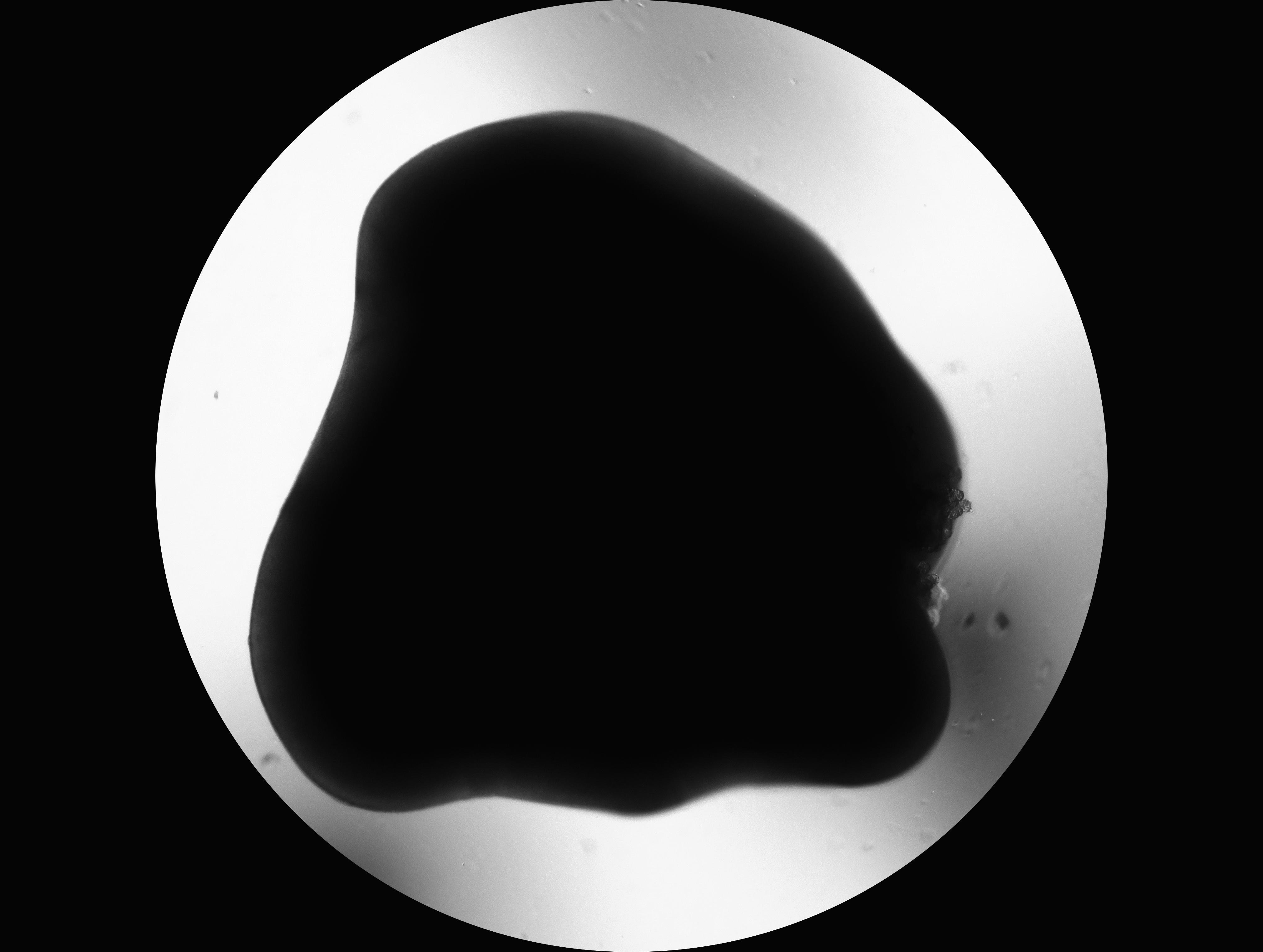

Supplement: Supplementary file 11 — Source data Fig. 3 [file 44319_2025_619_MOESM11_ESM.zip › Figure 3/C,D,F,G/Raw images_mask/OS_day90/MN 12C1 B C8 D90 2x/R_Day 90_0006.jpg]

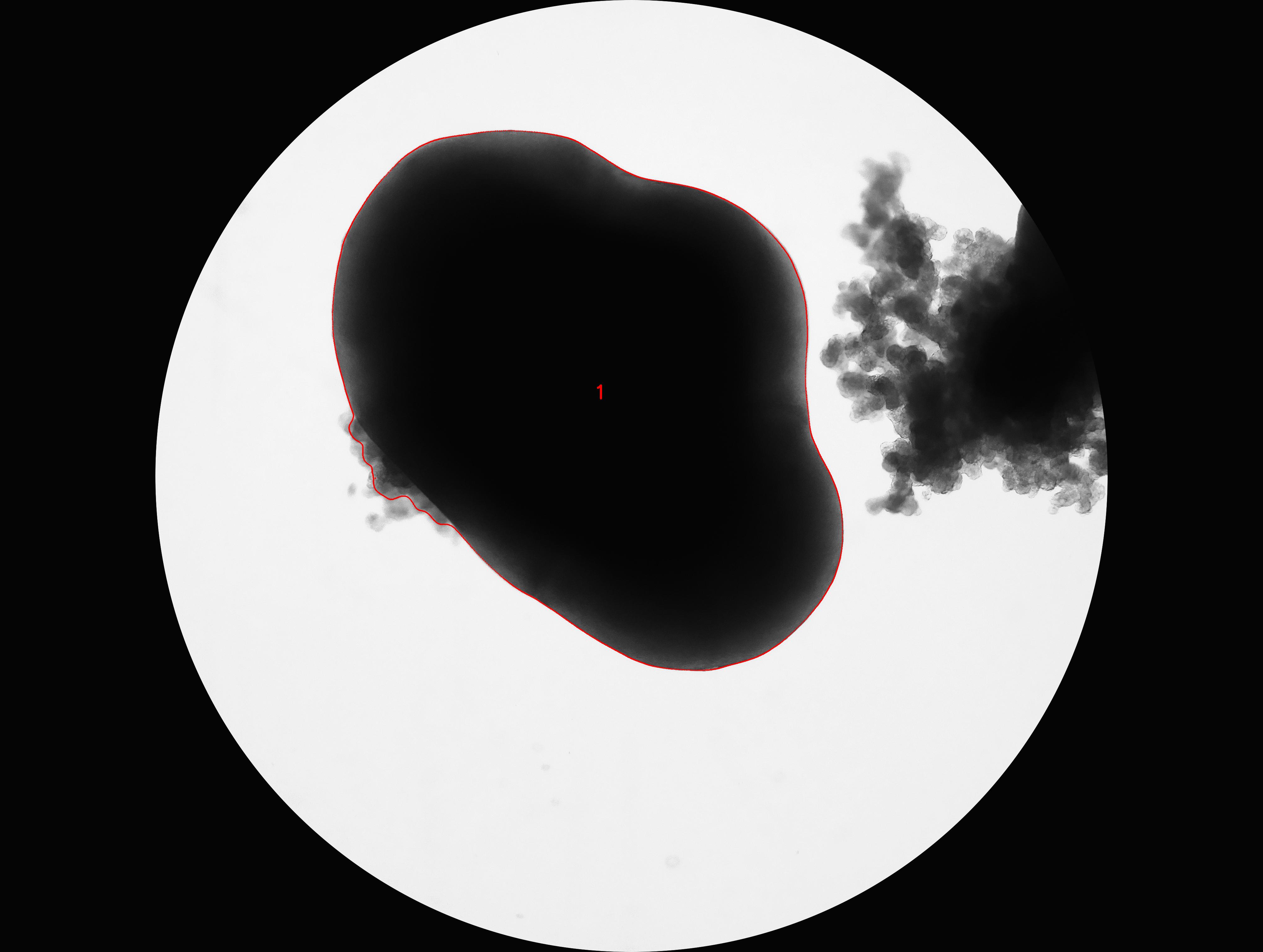

Supplement: Supplementary file 11 — Source data Fig. 3 [file 44319_2025_619_MOESM11_ESM.zip › Figure 3/C,D,F,G/Raw images_mask/OS_day90/MN 12C1 B C8 D90 2x/R_Day 90_0012.jpg]

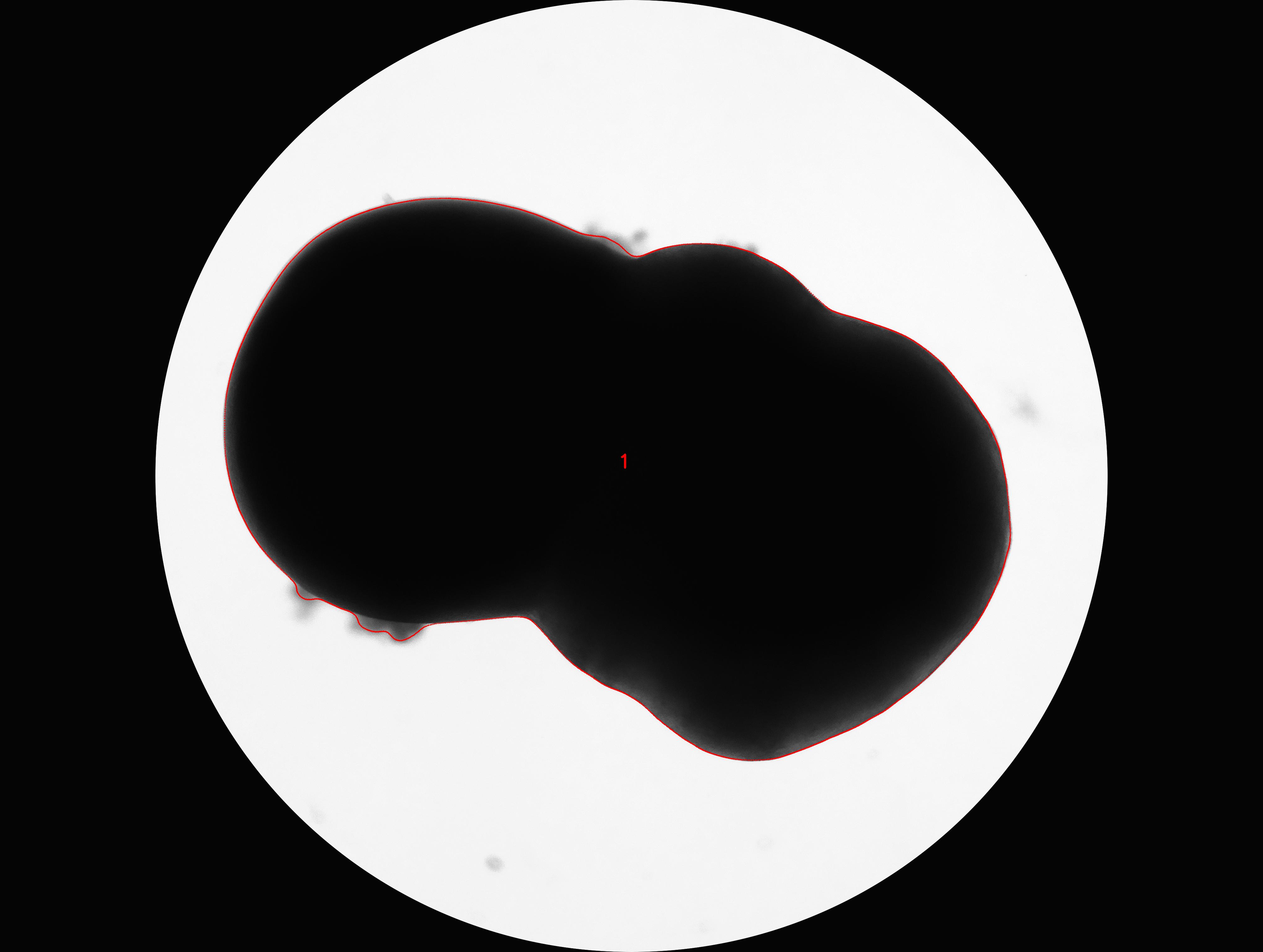

Supplement: Supplementary file 11 — Source data Fig. 3 [file 44319_2025_619_MOESM11_ESM.zip › Figure 3/C,D,F,G/Raw images_mask/OS_day90/MN 12C1 B C8 D90 2x/R_Day 90_0010.jpg]

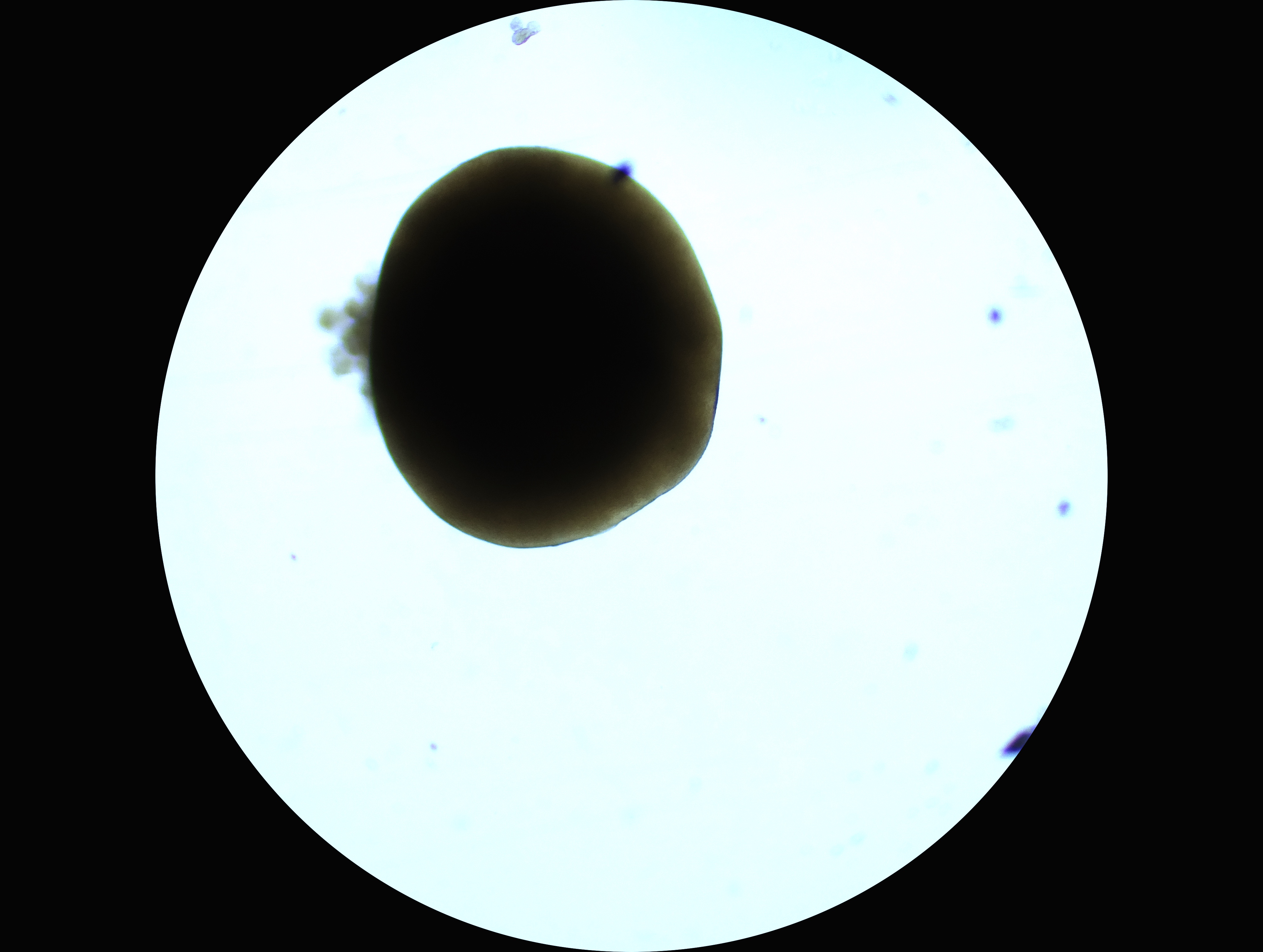

Supplement: Supplementary file 11 — Source data Fig. 3 [file 44319_2025_619_MOESM11_ESM.zip › Figure 3/C,D,F,G/Raw images_mask/OS_day90/MN 12C1 B C8 D90 2x/Day 90_0023.jpg]

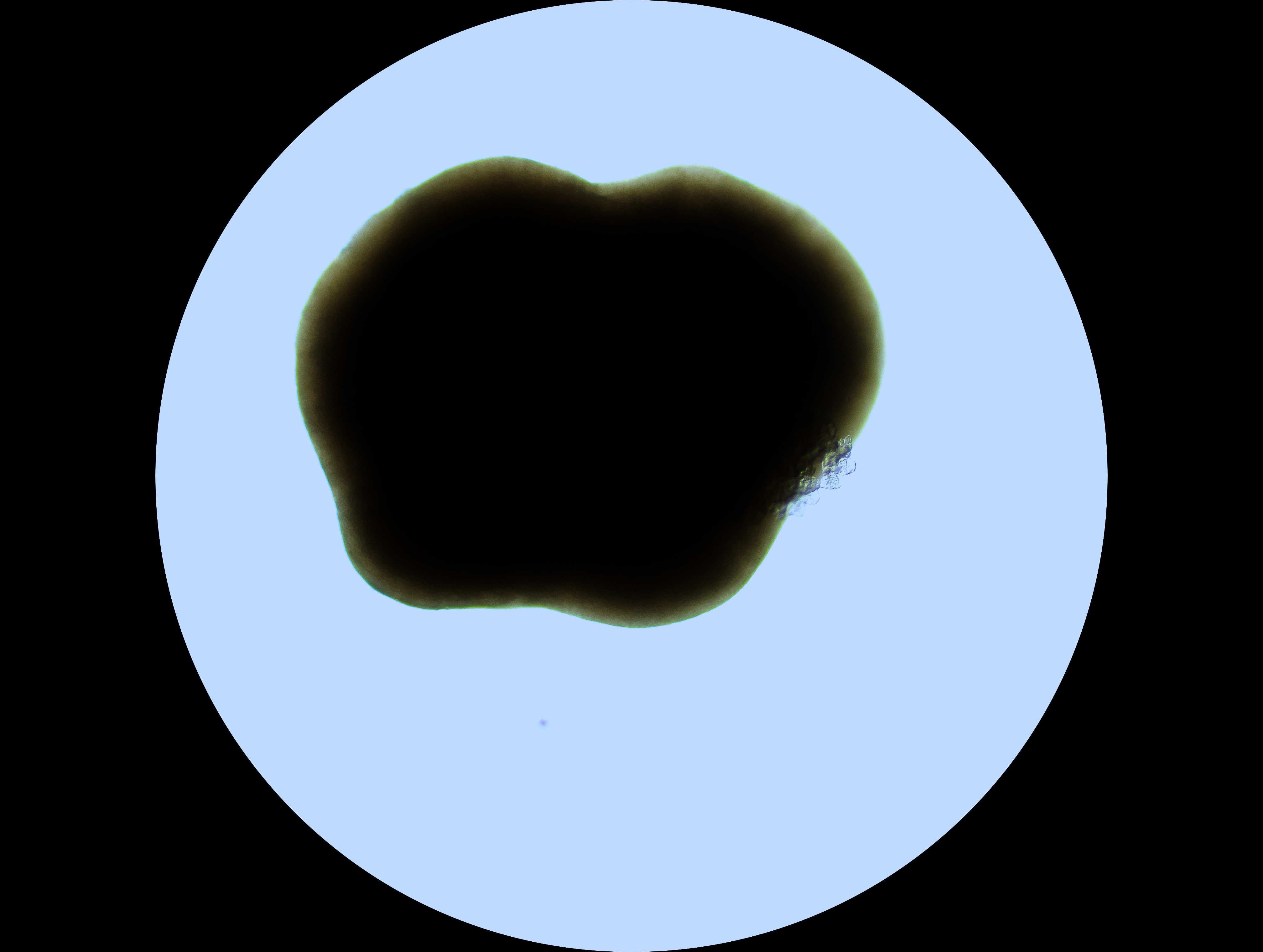

Supplement: Supplementary file 11 — Source data Fig. 3 [file 44319_2025_619_MOESM11_ESM.zip › Figure 3/C,D,F,G/Raw images_mask/OS_day90/MN 12C1 B C8 D90 2x/Day 90_0037.jpg]

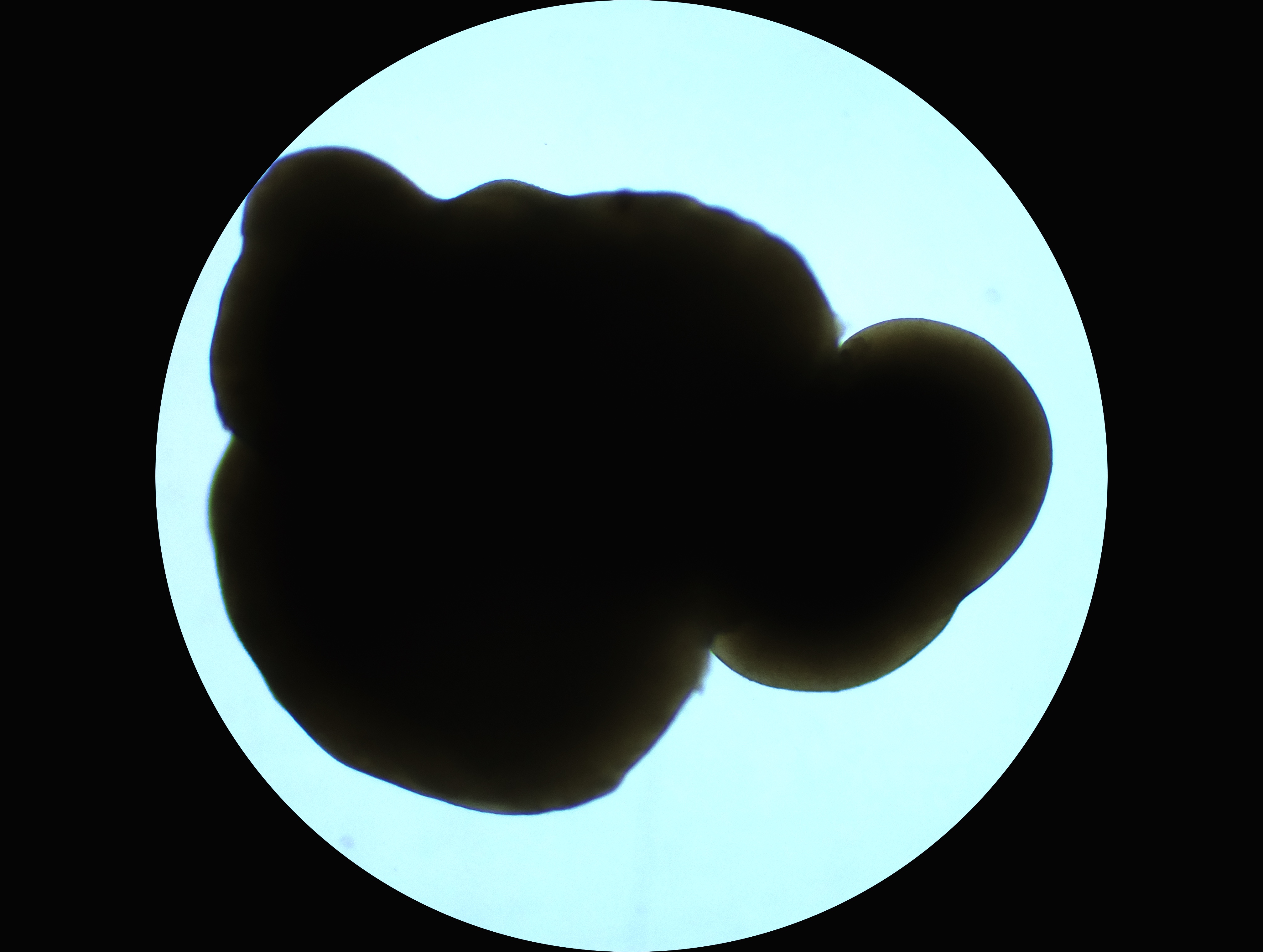

Supplement: Supplementary file 11 — Source data Fig. 3 [file 44319_2025_619_MOESM11_ESM.zip › Figure 3/C,D,F,G/Raw images_mask/OS_day90/MN 12C1 B C8 D90 2x/Day 90_0022.jpg]

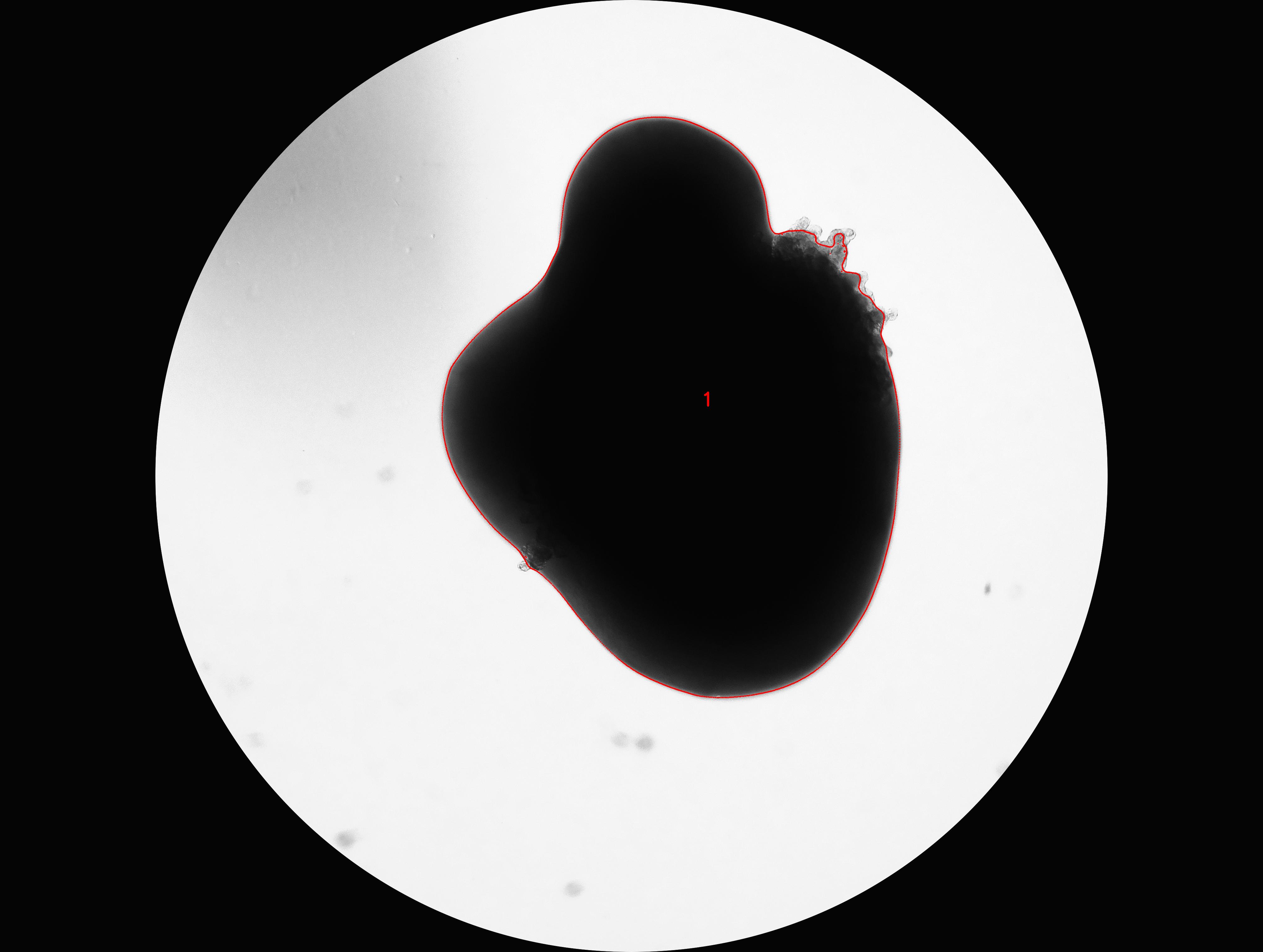

Supplement: Supplementary file 11 — Source data Fig. 3 [file 44319_2025_619_MOESM11_ESM.zip › Figure 3/C,D,F,G/Raw images_mask/OS_day90/MN 12C1 B C8 D90 2x/R_Day 90_0005.jpg]

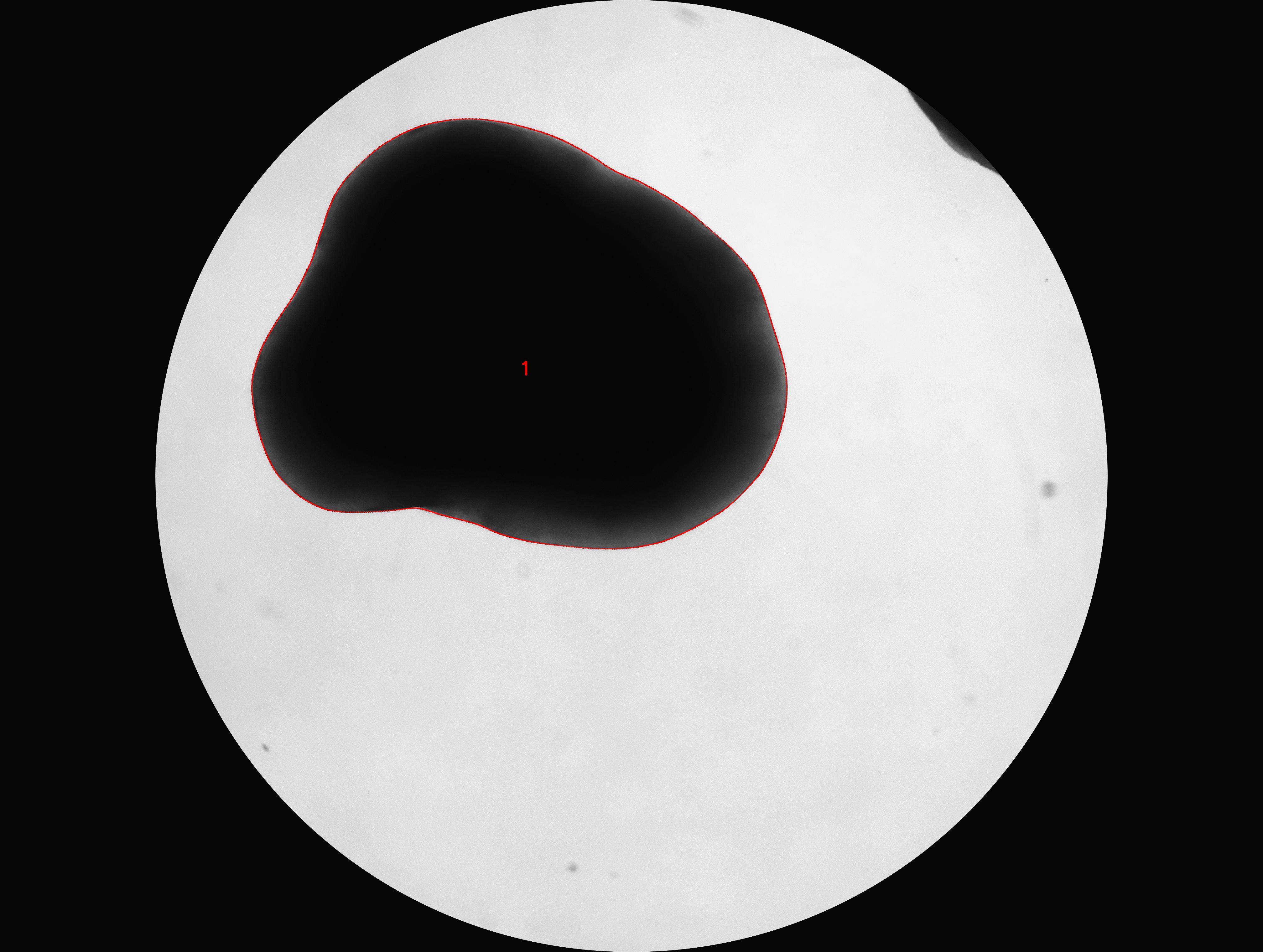

Supplement: Supplementary file 11 — Source data Fig. 3 [file 44319_2025_619_MOESM11_ESM.zip › Figure 3/C,D,F,G/Raw images_mask/OS_day90/MN 12C1 B C8 D90 2x/R_Day 90_0029.jpg]

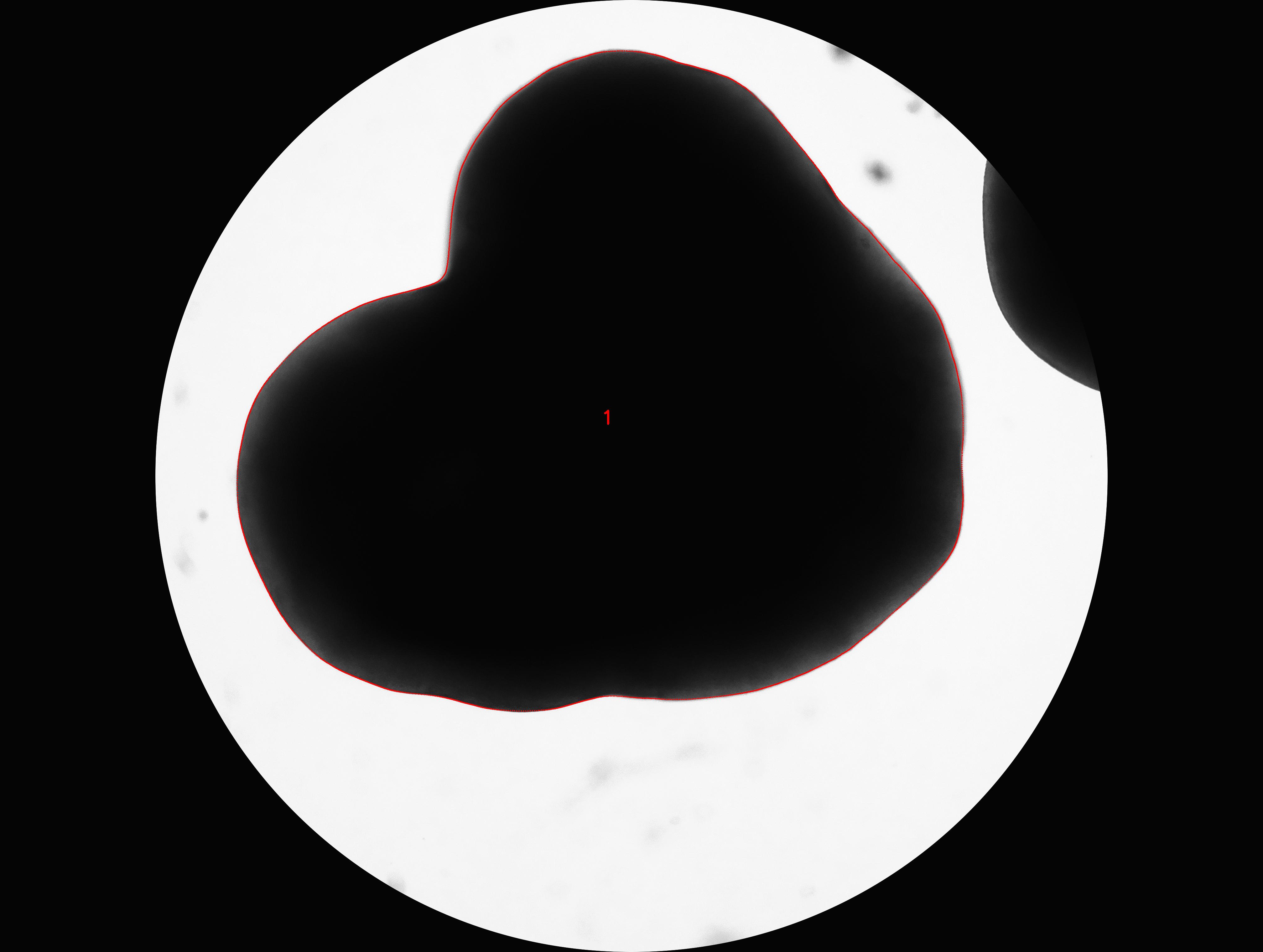

Supplement: Supplementary file 11 — Source data Fig. 3 [file 44319_2025_619_MOESM11_ESM.zip › Figure 3/C,D,F,G/Raw images_mask/OS_day90/MN 12C1 B C8 D90 2x/R_Day 90_0001.jpg]

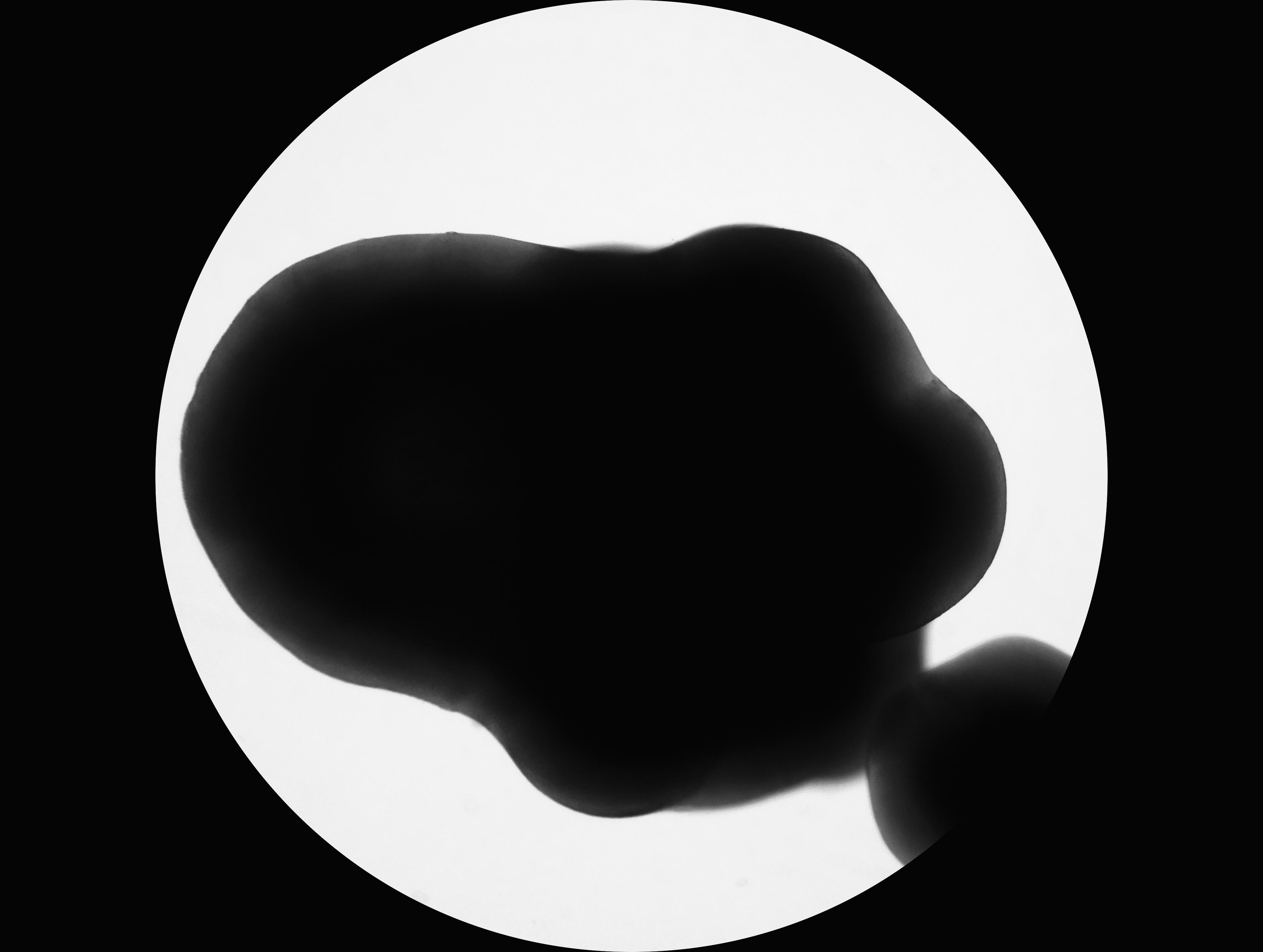

Supplement: Supplementary file 11 — Source data Fig. 3 [file 44319_2025_619_MOESM11_ESM.zip › Figure 3/C,D,F,G/Raw images_mask/OS_day90/MN 12C1 B C8 D90 2x/R_Day 90_0015.jpg]

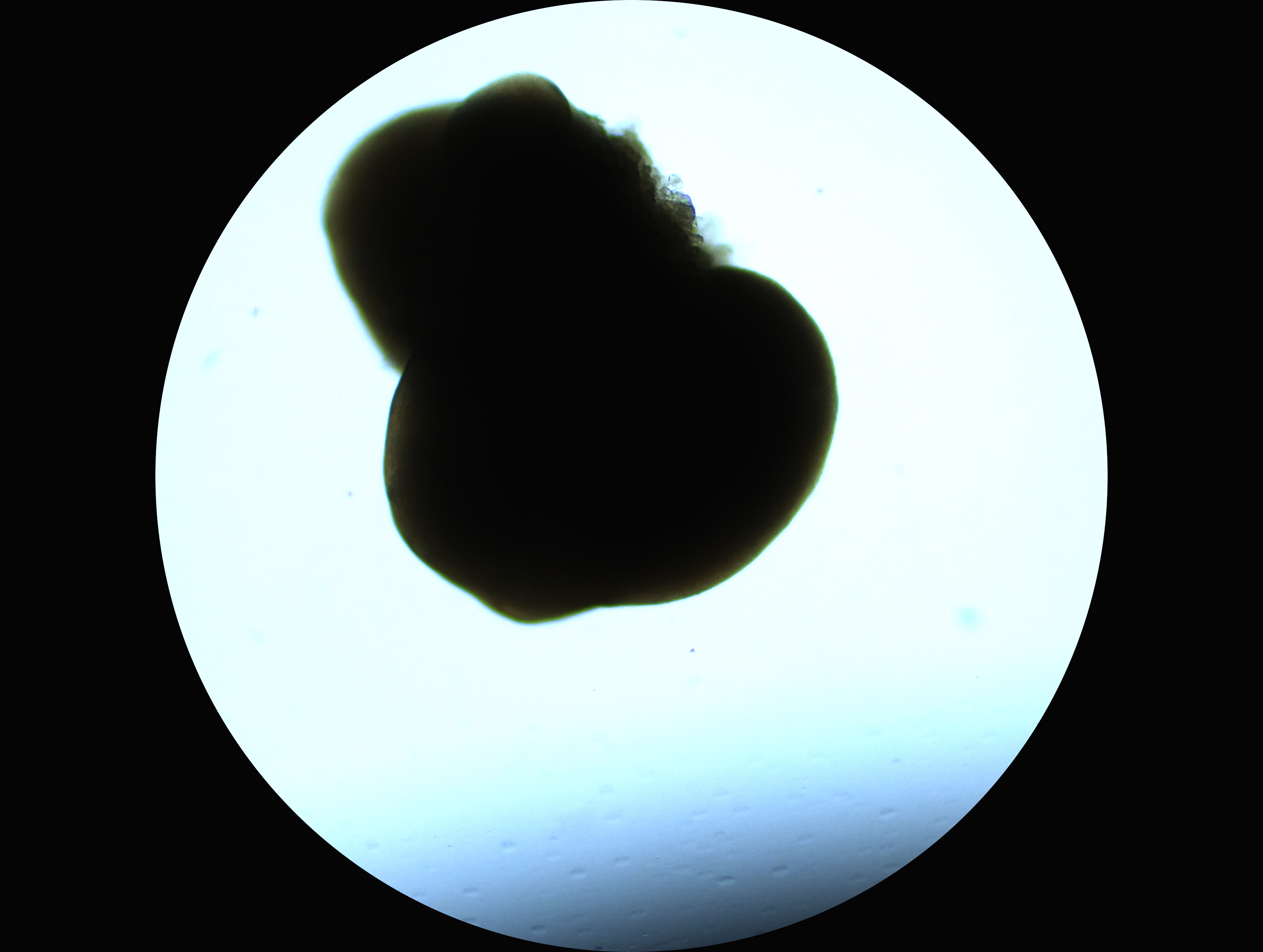

Supplement: Supplementary file 11 — Source data Fig. 3 [file 44319_2025_619_MOESM11_ESM.zip › Figure 3/C,D,F,G/Raw images_mask/OS_day90/MN 12C1 B C8 D90 2x/Day 90_0032.jpg]

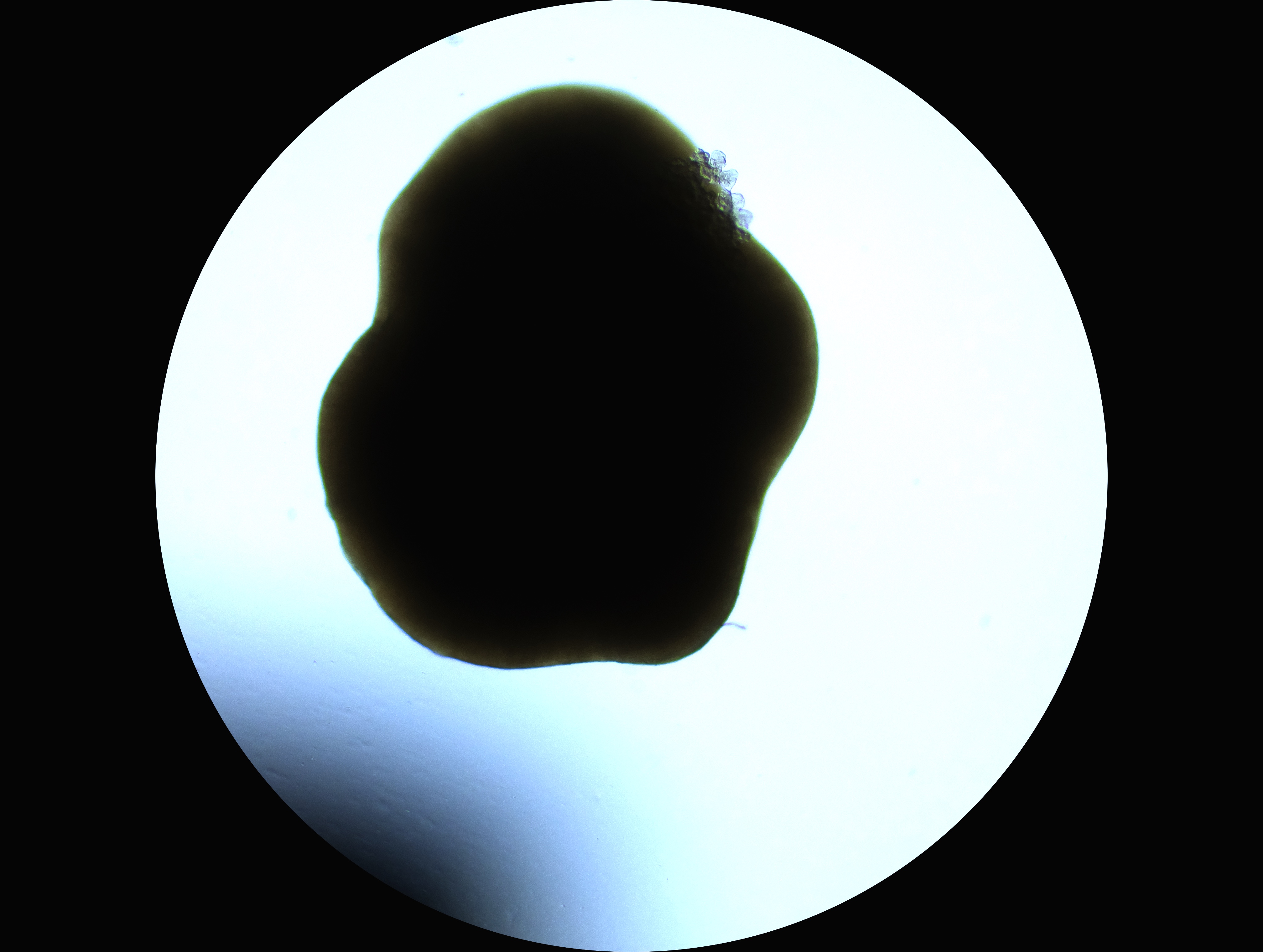

Supplement: Supplementary file 11 — Source data Fig. 3 [file 44319_2025_619_MOESM11_ESM.zip › Figure 3/C,D,F,G/Raw images_mask/OS_day90/MN 12C1 B C8 D90 2x/Day 90_0033.jpg]

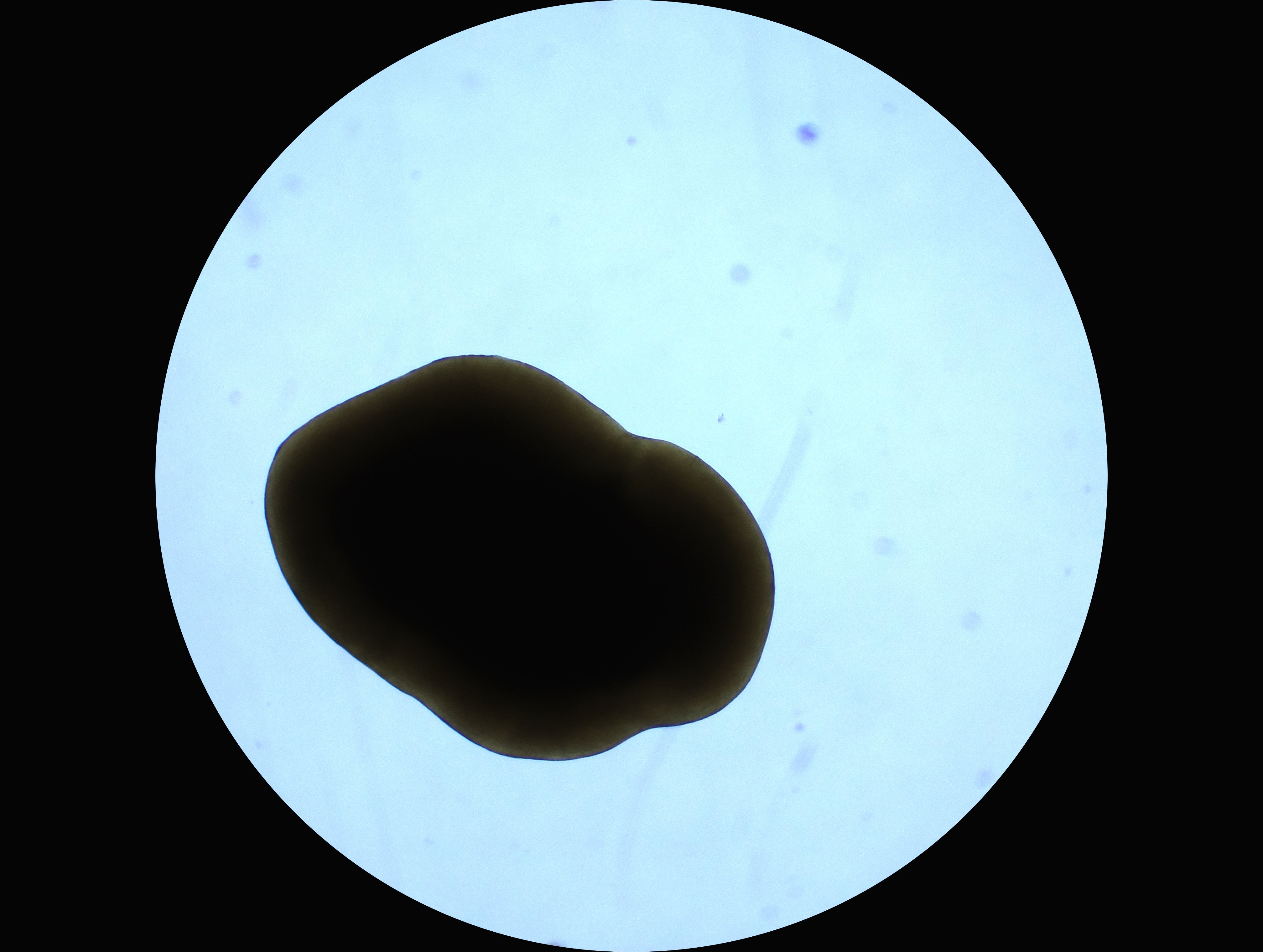

Supplement: Supplementary file 11 — Source data Fig. 3 [file 44319_2025_619_MOESM11_ESM.zip › Figure 3/C,D,F,G/Raw images_mask/OS_day90/MN 12C1 B C8 D90 2x/Day 90_0027.jpg]

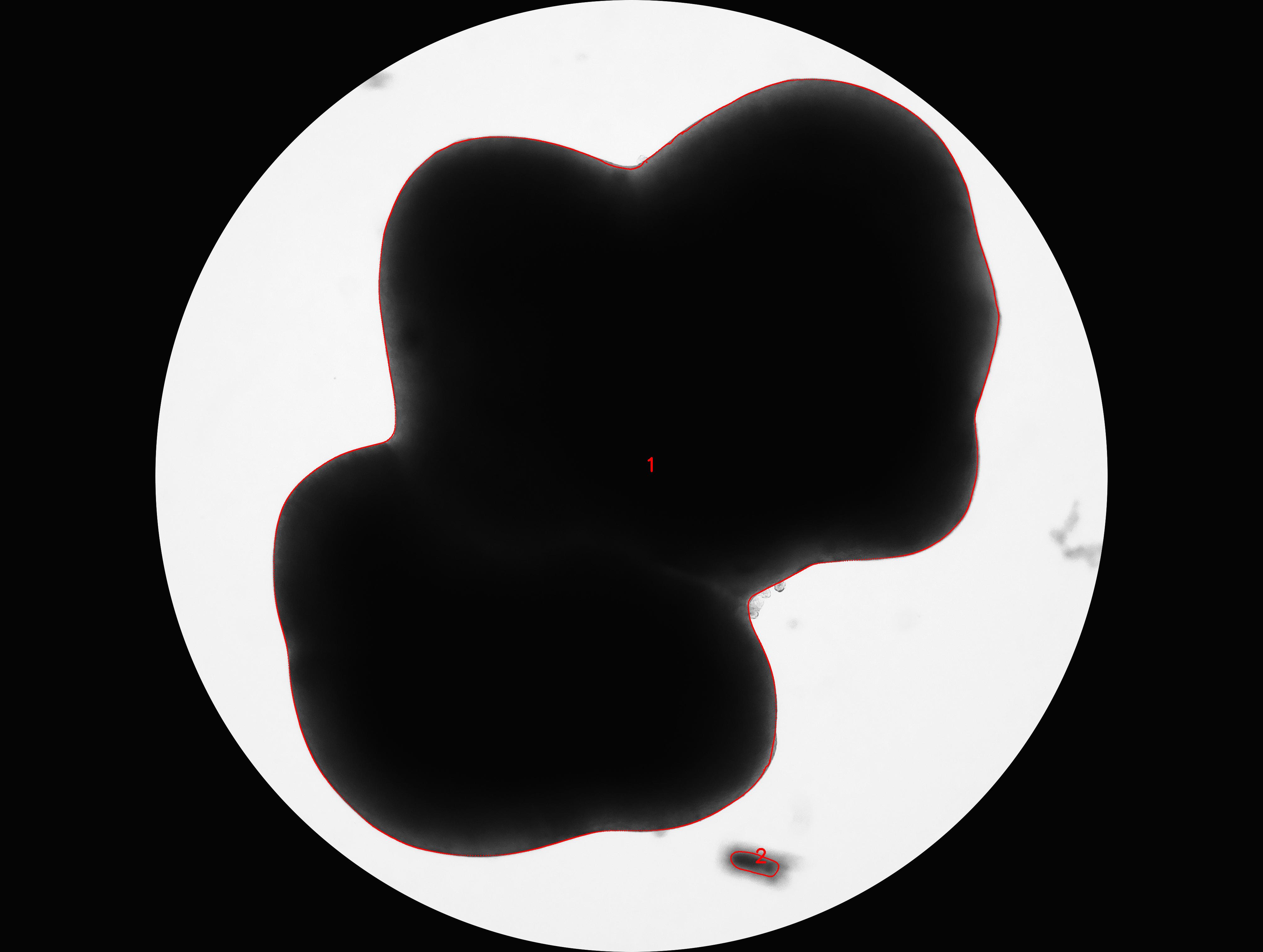

Supplement: Supplementary file 11 — Source data Fig. 3 [file 44319_2025_619_MOESM11_ESM.zip › Figure 3/C,D,F,G/Raw images_mask/OS_day90/MN 12C1 B C8 D90 2x/R_Day 90_0000.jpg]

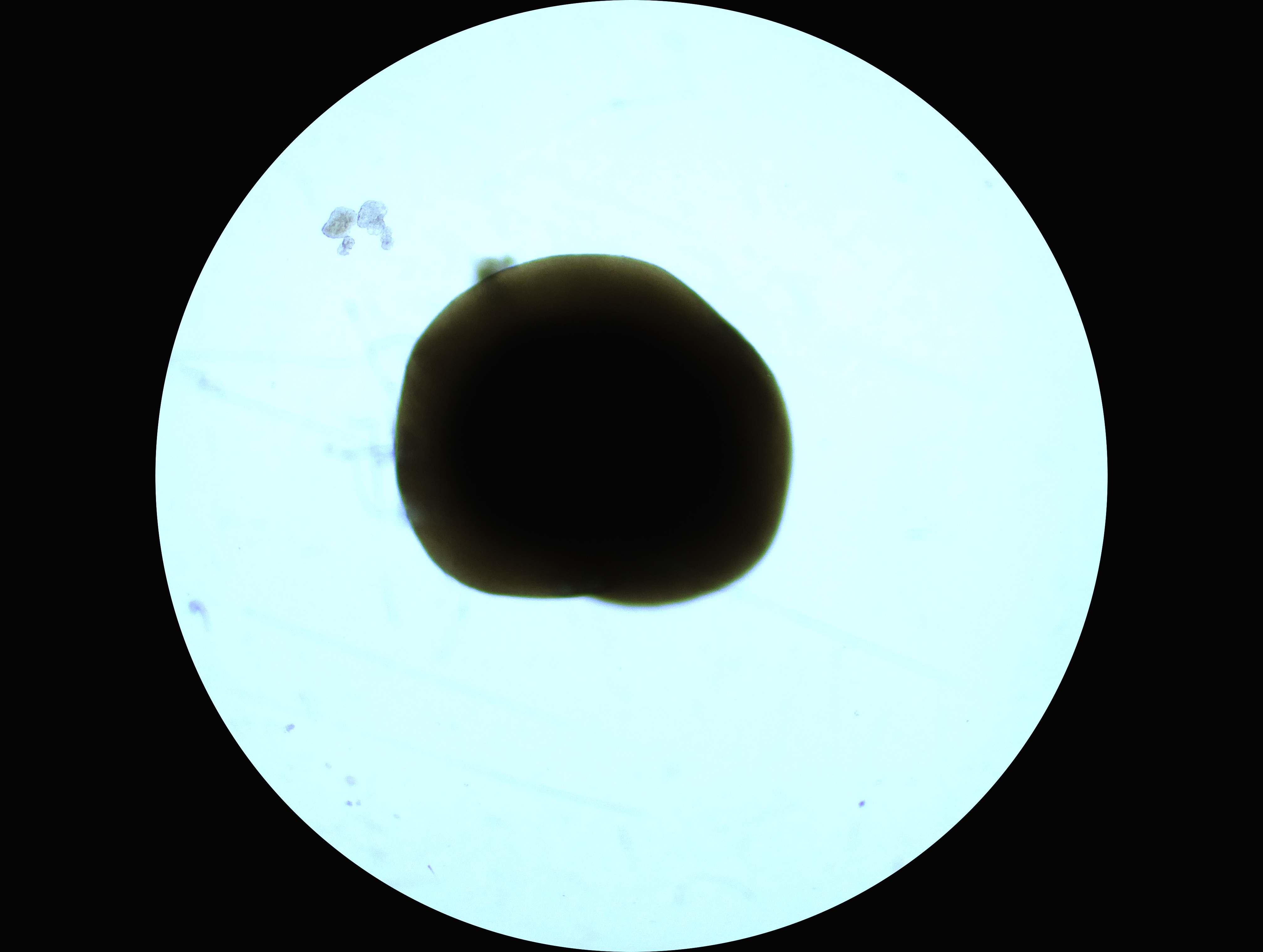

Supplement: Supplementary file 11 — Source data Fig. 3 [file 44319_2025_619_MOESM11_ESM.zip › Figure 3/C,D,F,G/Raw images_mask/OS_day90/MN 12C1 B C8 D90 2x/Day 90_0019.jpg]
