# Supplementary figures and images for: Increased reproducibility of brain organoids through controlled fluid dynamics (part 2 of 2)
Source: EMBO Rep. 2025 Nov 19;26(24):6209–39. doi: 10.1038/s44319-025-00619-x (PMC12715241; doi:10.1038/s44319-025-00619-x)

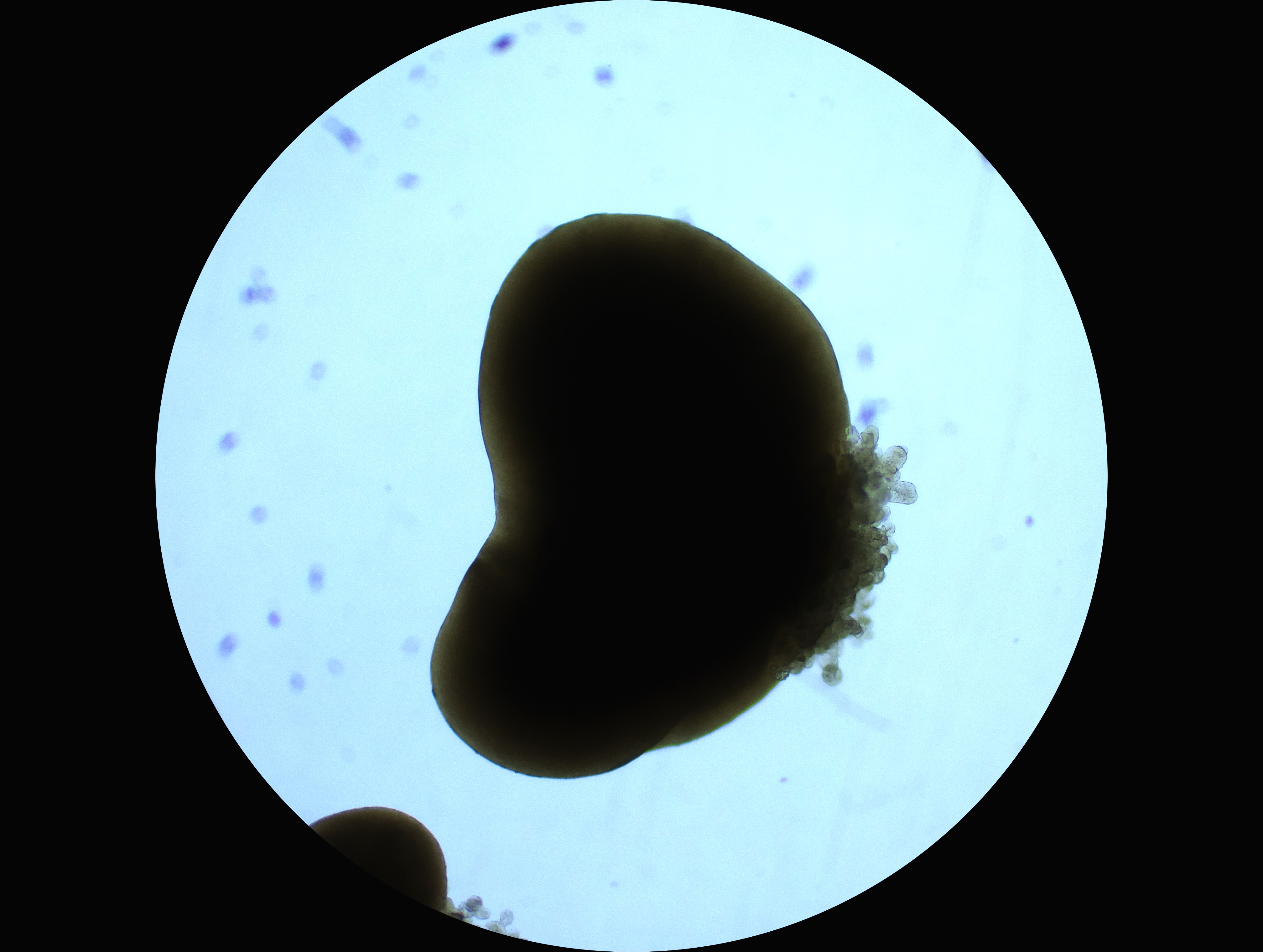

Supplement: Supplementary file 11 — Source data Fig. 3 [file 44319_2025_619_MOESM11_ESM.zip › Figure 3/C,D,F,G/Raw images_mask/OS_day90/MN 12C1 B C8 D90 2x/Day 90_0031.jpg]

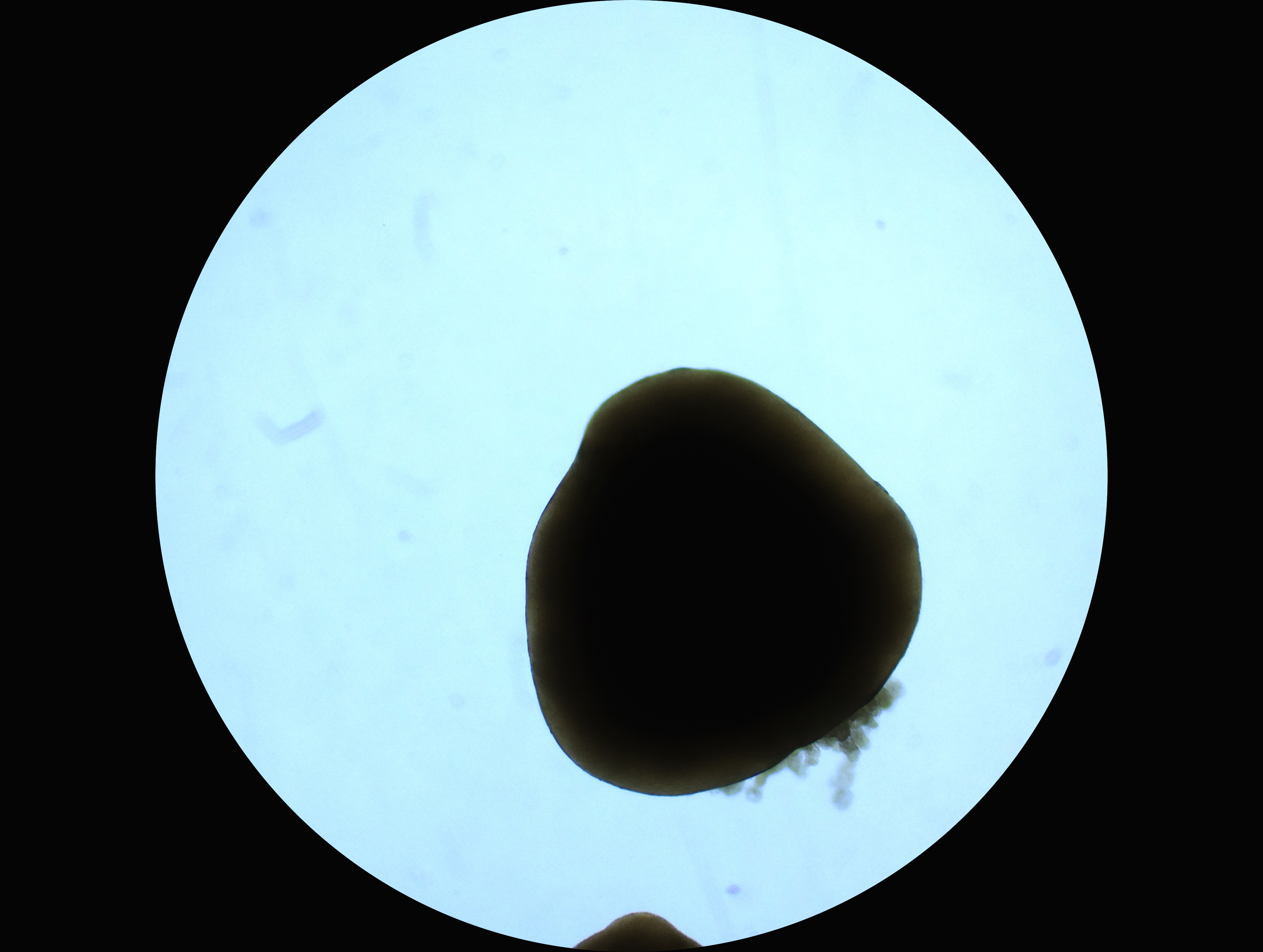

Supplement: Supplementary file 11 — Source data Fig. 3 [file 44319_2025_619_MOESM11_ESM.zip › Figure 3/C,D,F,G/Raw images_mask/OS_day90/MN 12C1 B C8 D90 2x/Day 90_0025.jpg]

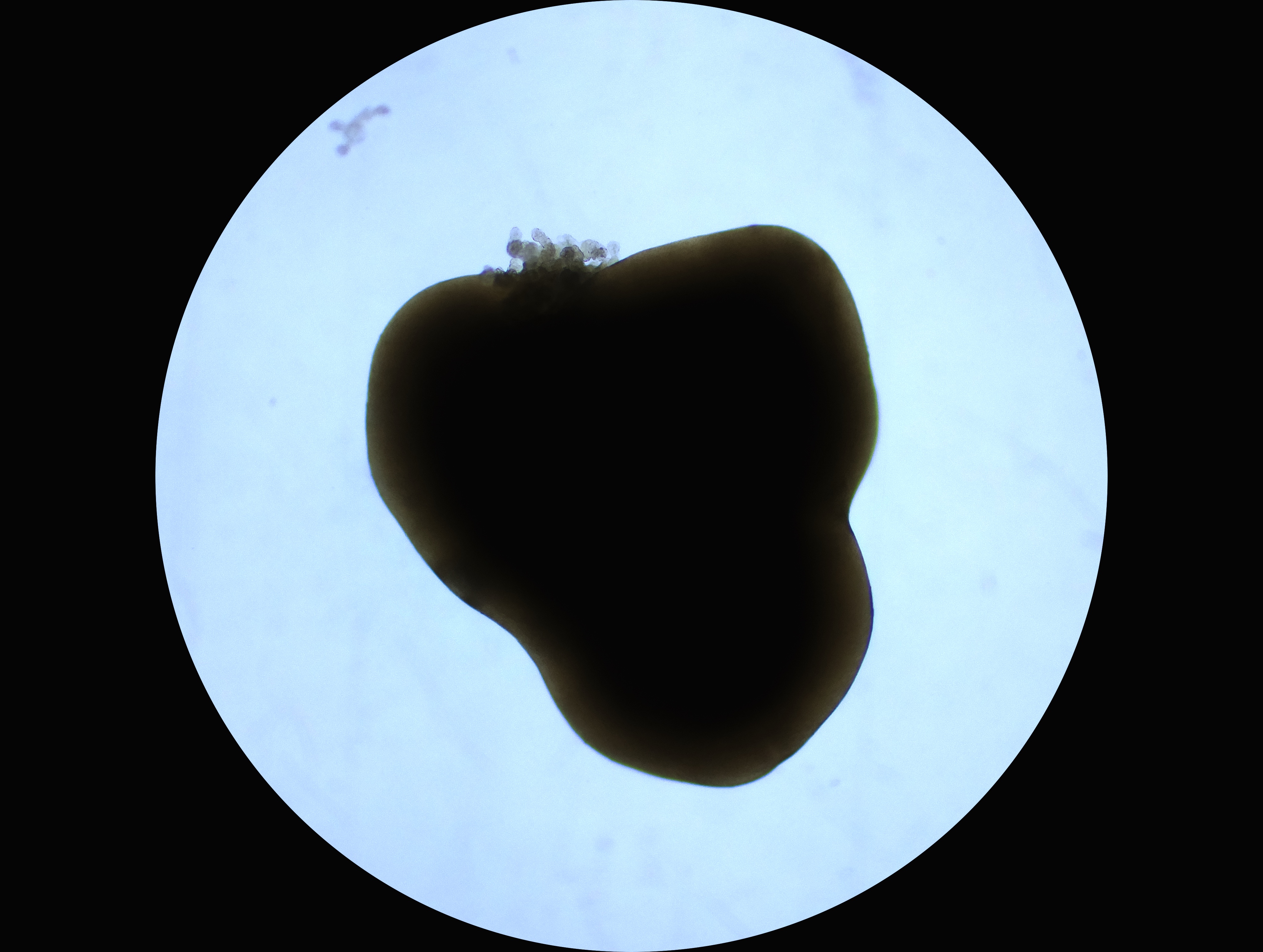

Supplement: Supplementary file 11 — Source data Fig. 3 [file 44319_2025_619_MOESM11_ESM.zip › Figure 3/C,D,F,G/Raw images_mask/OS_day90/MN 12C1 B C8 D90 2x/Day 90_0030.jpg]

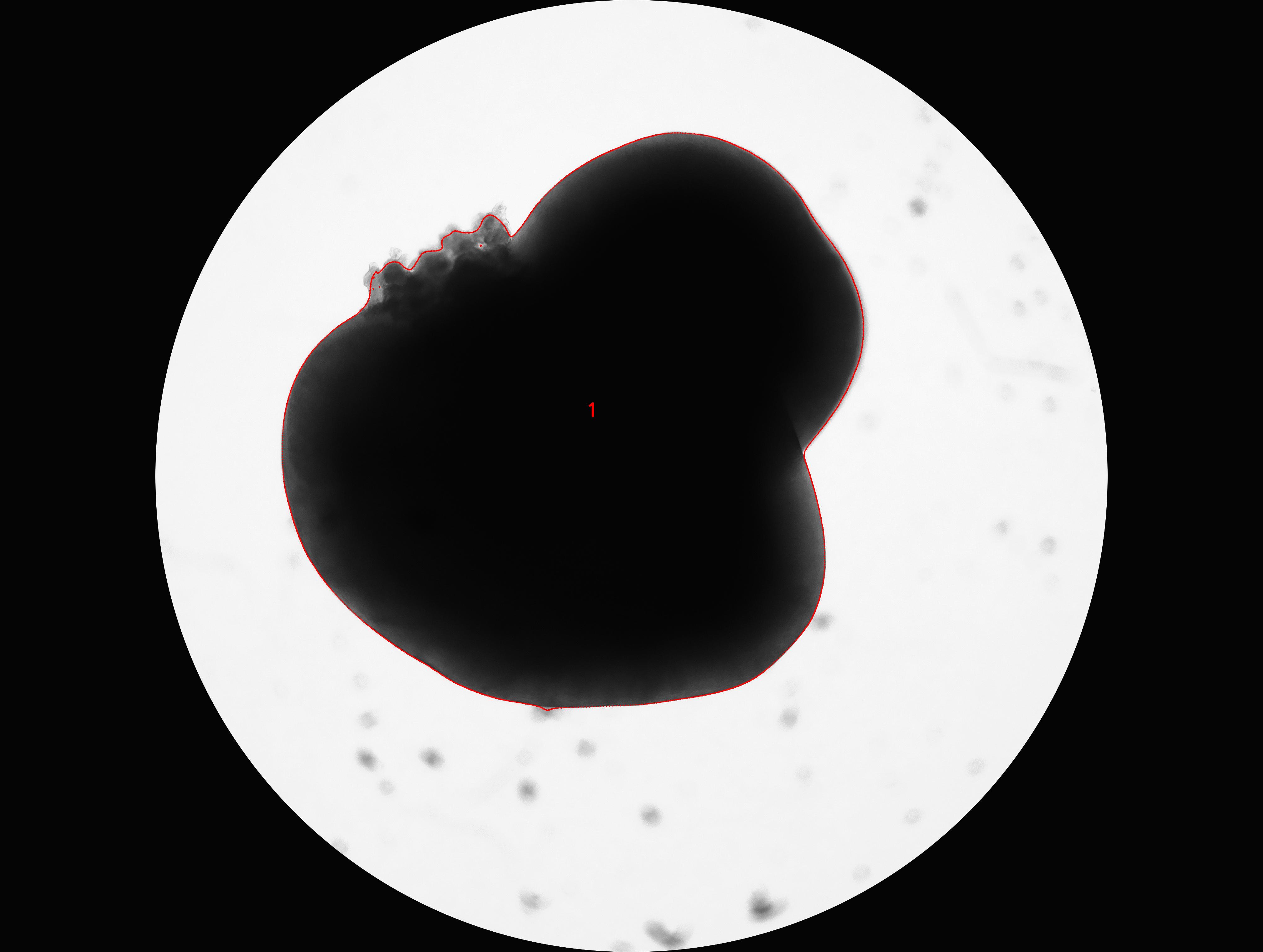

Supplement: Supplementary file 11 — Source data Fig. 3 [file 44319_2025_619_MOESM11_ESM.zip › Figure 3/C,D,F,G/Raw images_mask/OS_day90/MN 12C1 B C8 D90 2x/R_Day 90_0003.jpg]

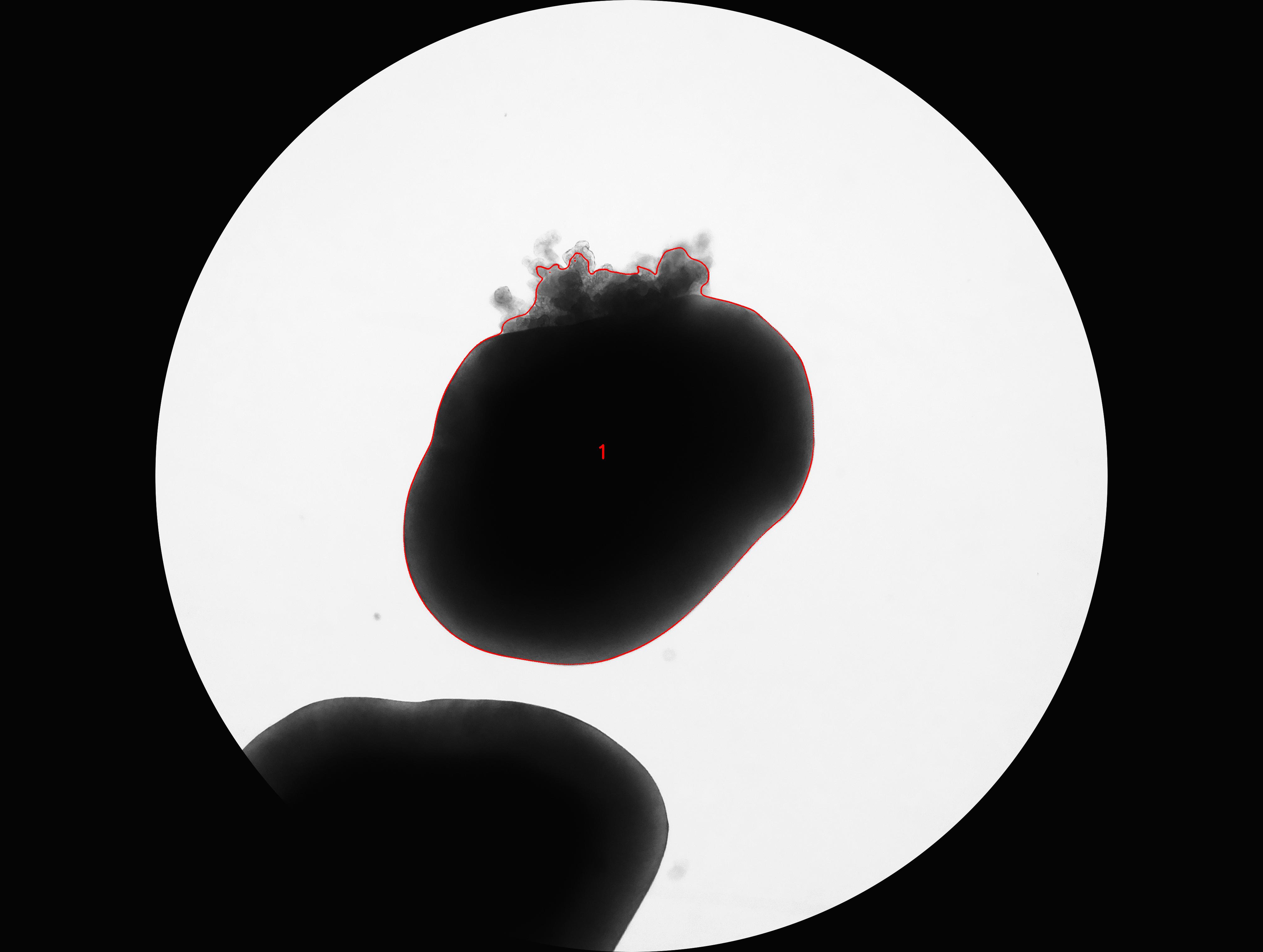

Supplement: Supplementary file 11 — Source data Fig. 3 [file 44319_2025_619_MOESM11_ESM.zip › Figure 3/C,D,F,G/Raw images_mask/OS_day90/MN 12C1 B C8 D90 2x/R_Day 90_0017.jpg]

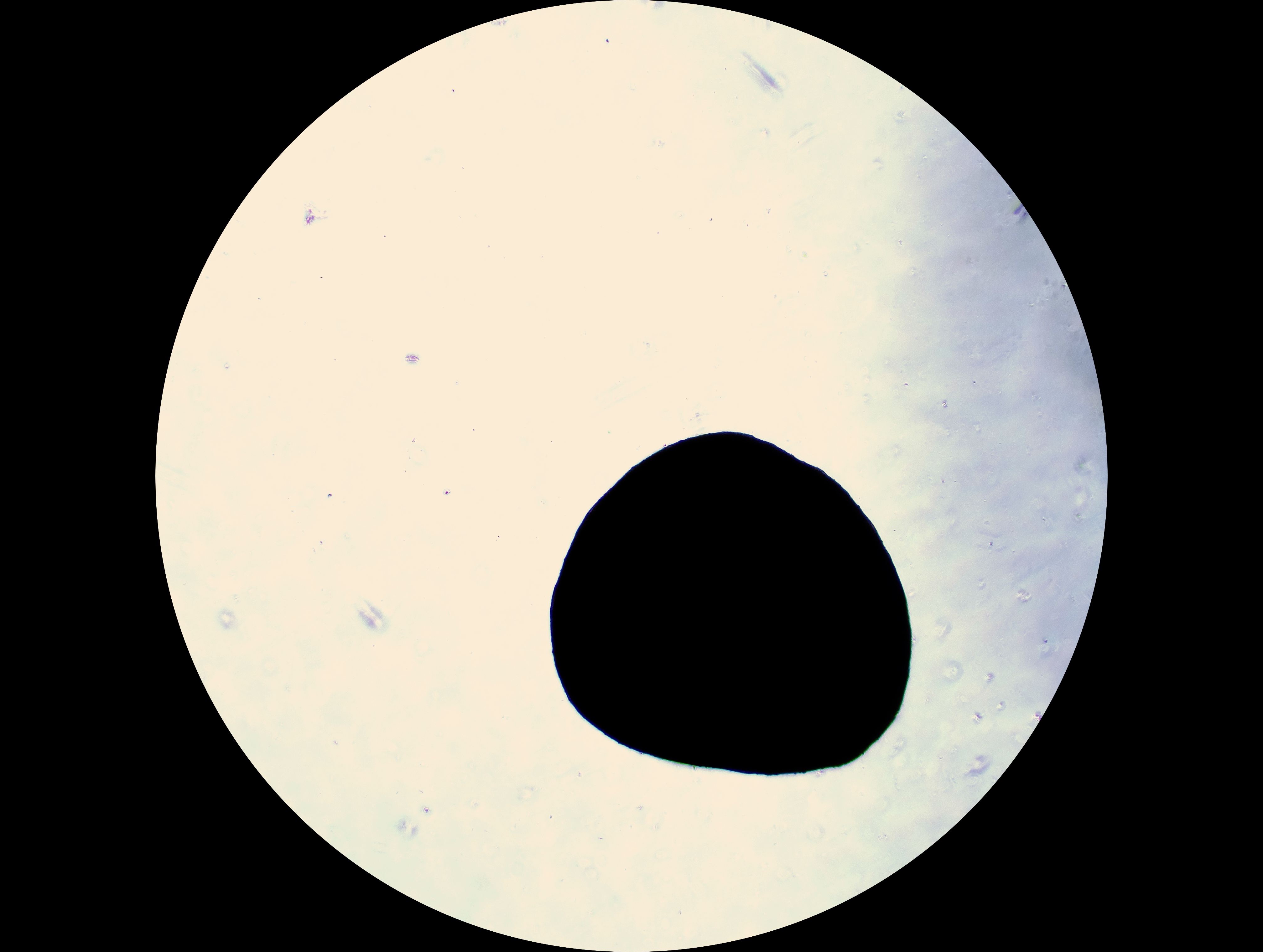

Supplement: Supplementary file 11 — Source data Fig. 3 [file 44319_2025_619_MOESM11_ESM.zip › Figure 3/C,D,F,G/Raw images_mask/OS_day90/MN 12C1 B C12 D90 2x/day 90_0015.jpg]

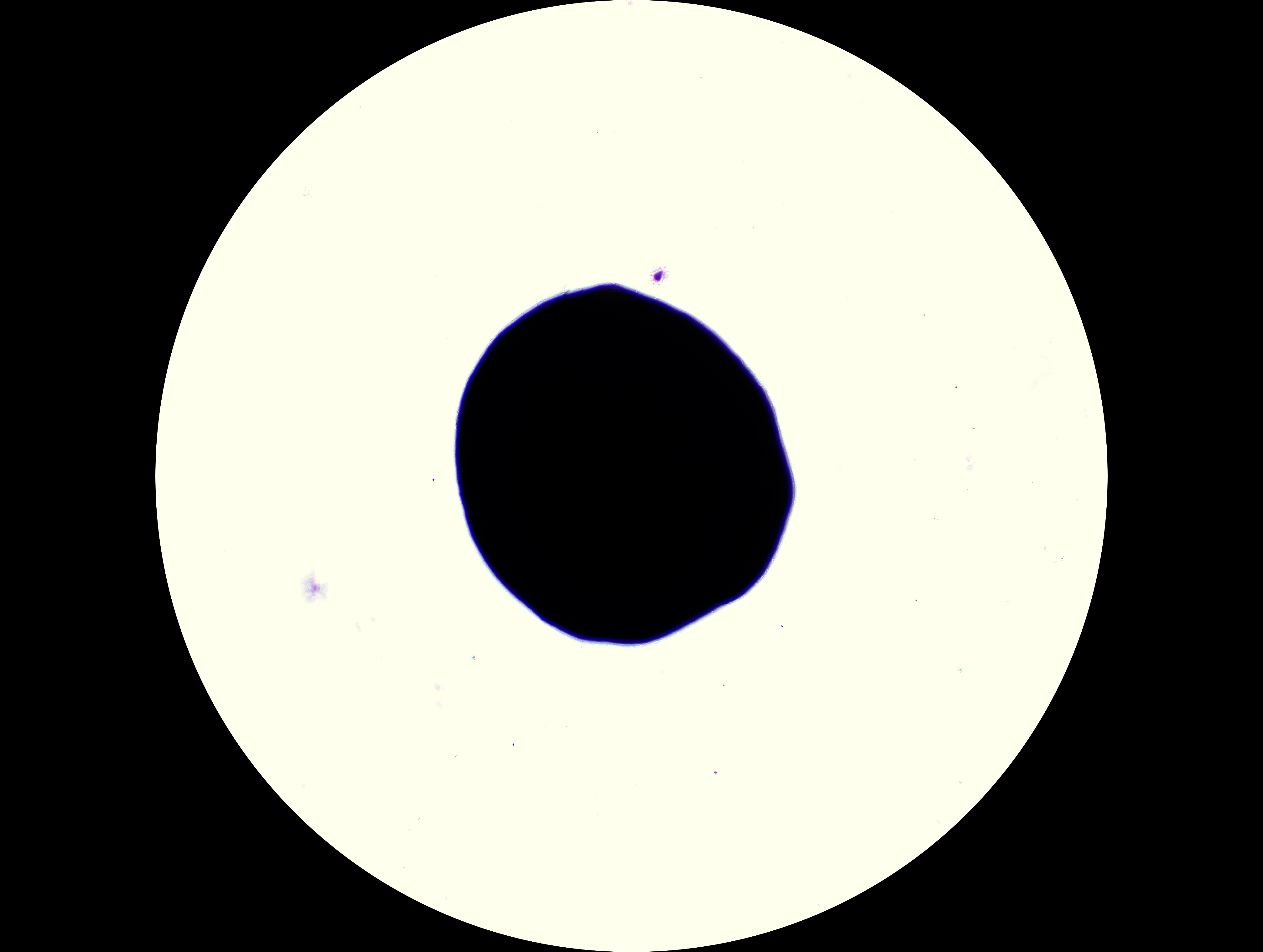

Supplement: Supplementary file 11 — Source data Fig. 3 [file 44319_2025_619_MOESM11_ESM.zip › Figure 3/C,D,F,G/Raw images_mask/OS_day90/MN 12C1 B C12 D90 2x/day 90_0000.jpg]

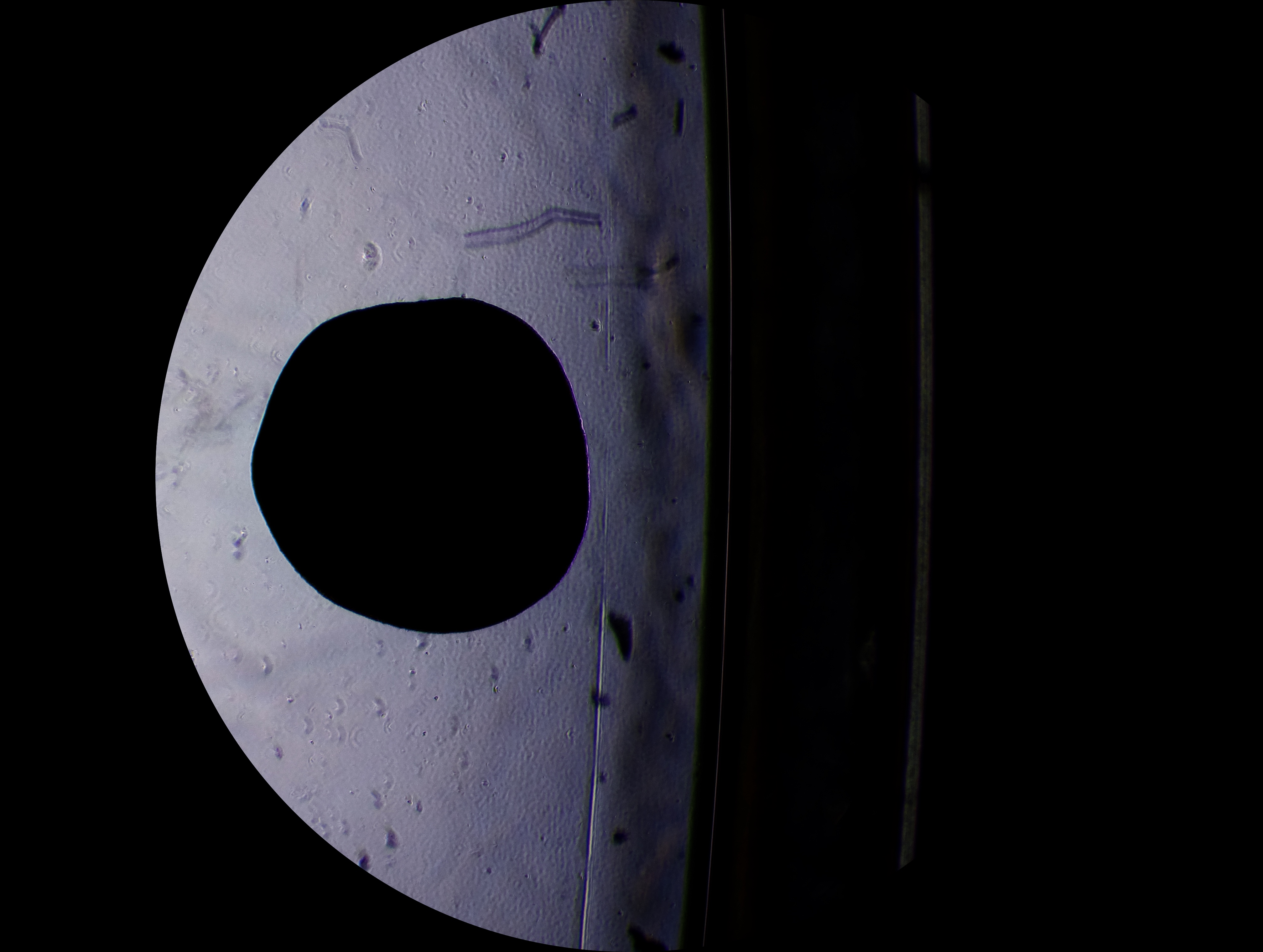

Supplement: Supplementary file 11 — Source data Fig. 3 [file 44319_2025_619_MOESM11_ESM.zip › Figure 3/C,D,F,G/Raw images_mask/OS_day90/MN 12C1 B C12 D90 2x/day 90_0014.jpg]

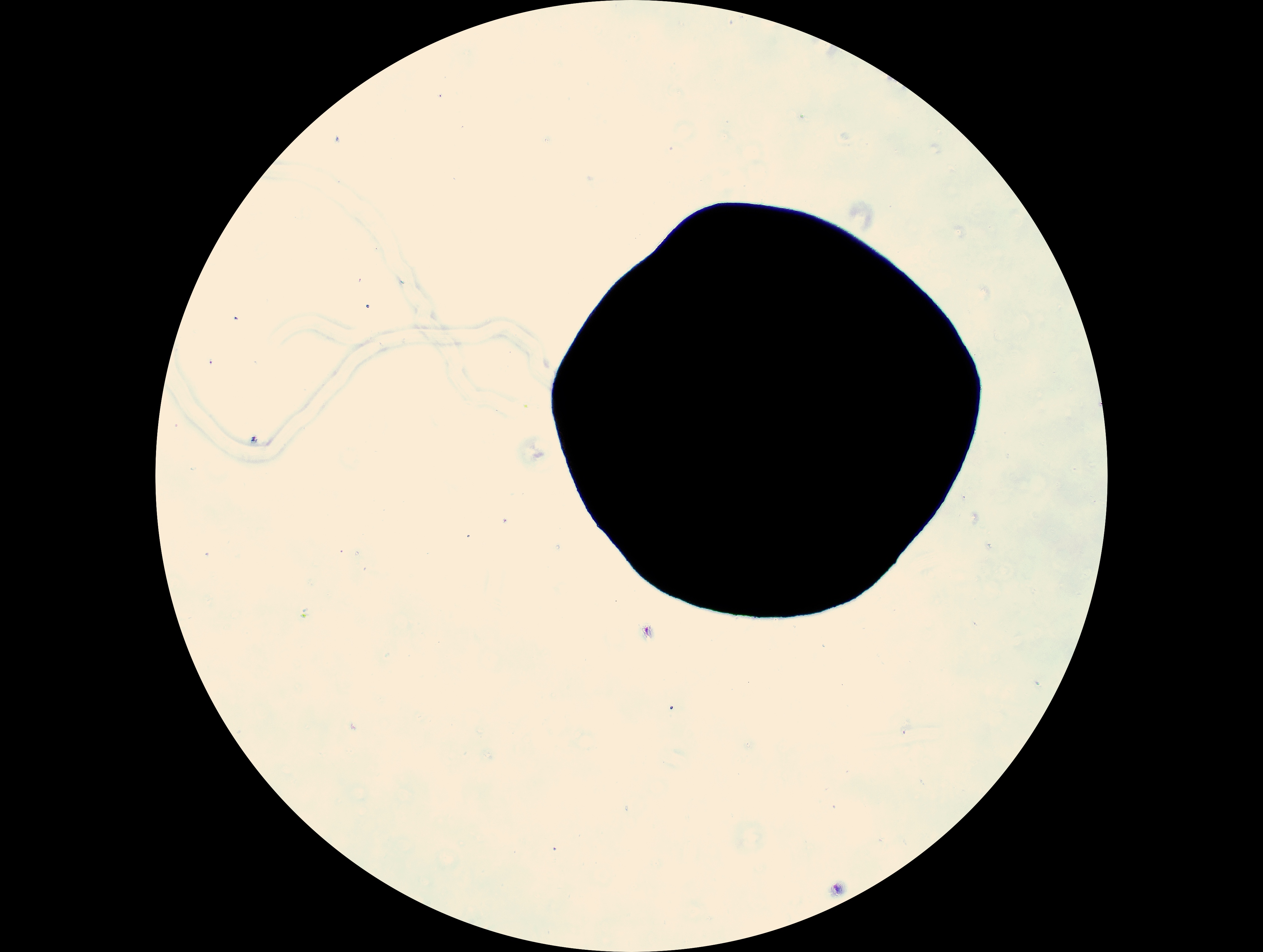

Supplement: Supplementary file 11 — Source data Fig. 3 [file 44319_2025_619_MOESM11_ESM.zip › Figure 3/C,D,F,G/Raw images_mask/OS_day90/MN 12C1 B C12 D90 2x/day 90_0002.jpg]

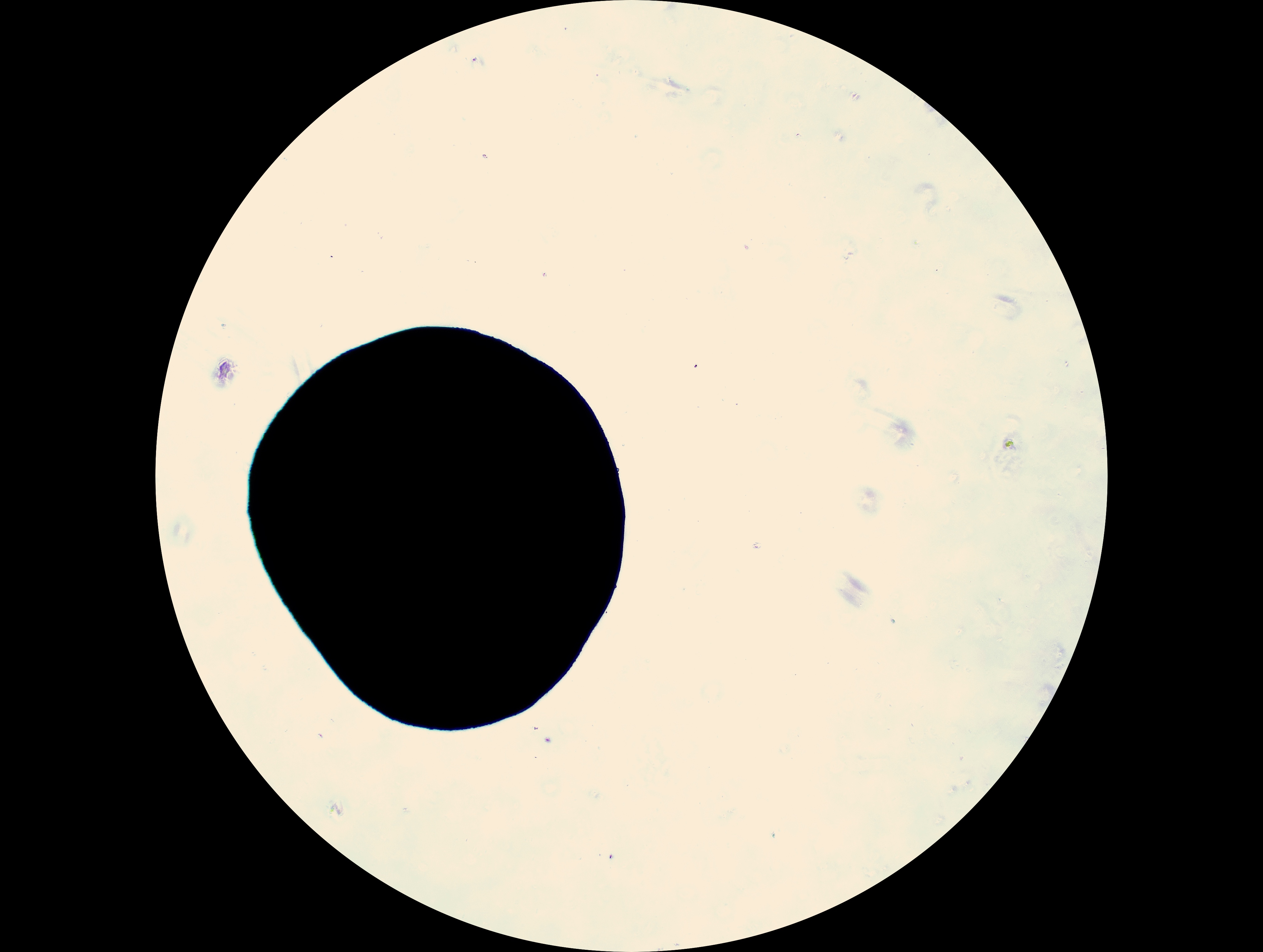

Supplement: Supplementary file 11 — Source data Fig. 3 [file 44319_2025_619_MOESM11_ESM.zip › Figure 3/C,D,F,G/Raw images_mask/OS_day90/MN 12C1 B C12 D90 2x/day 90_0003.jpg]

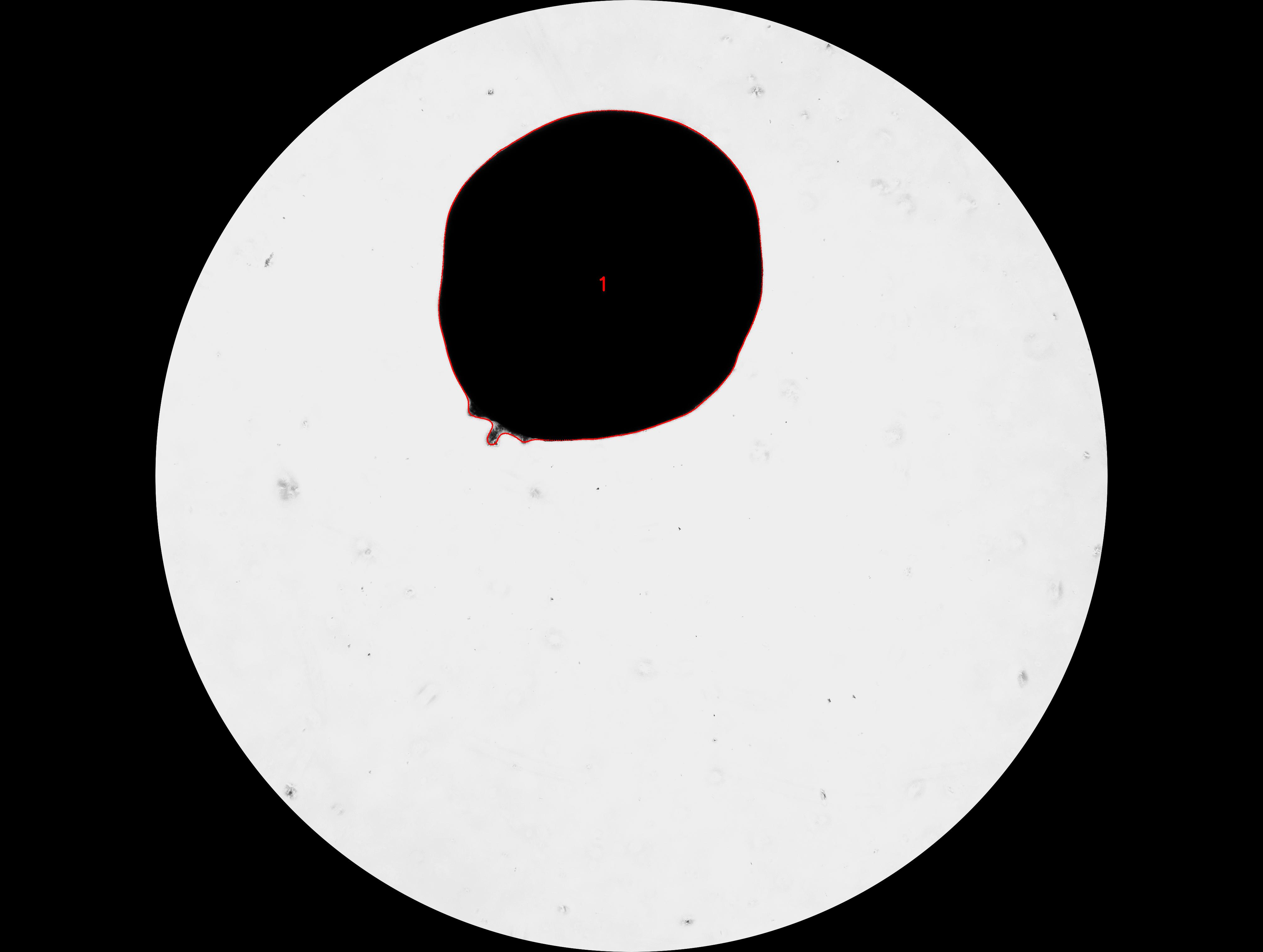

Supplement: Supplementary file 11 — Source data Fig. 3 [file 44319_2025_619_MOESM11_ESM.zip › Figure 3/C,D,F,G/Raw images_mask/OS_day90/MN 12C1 B C12 D90 2x/R_day 90_0008.jpg]

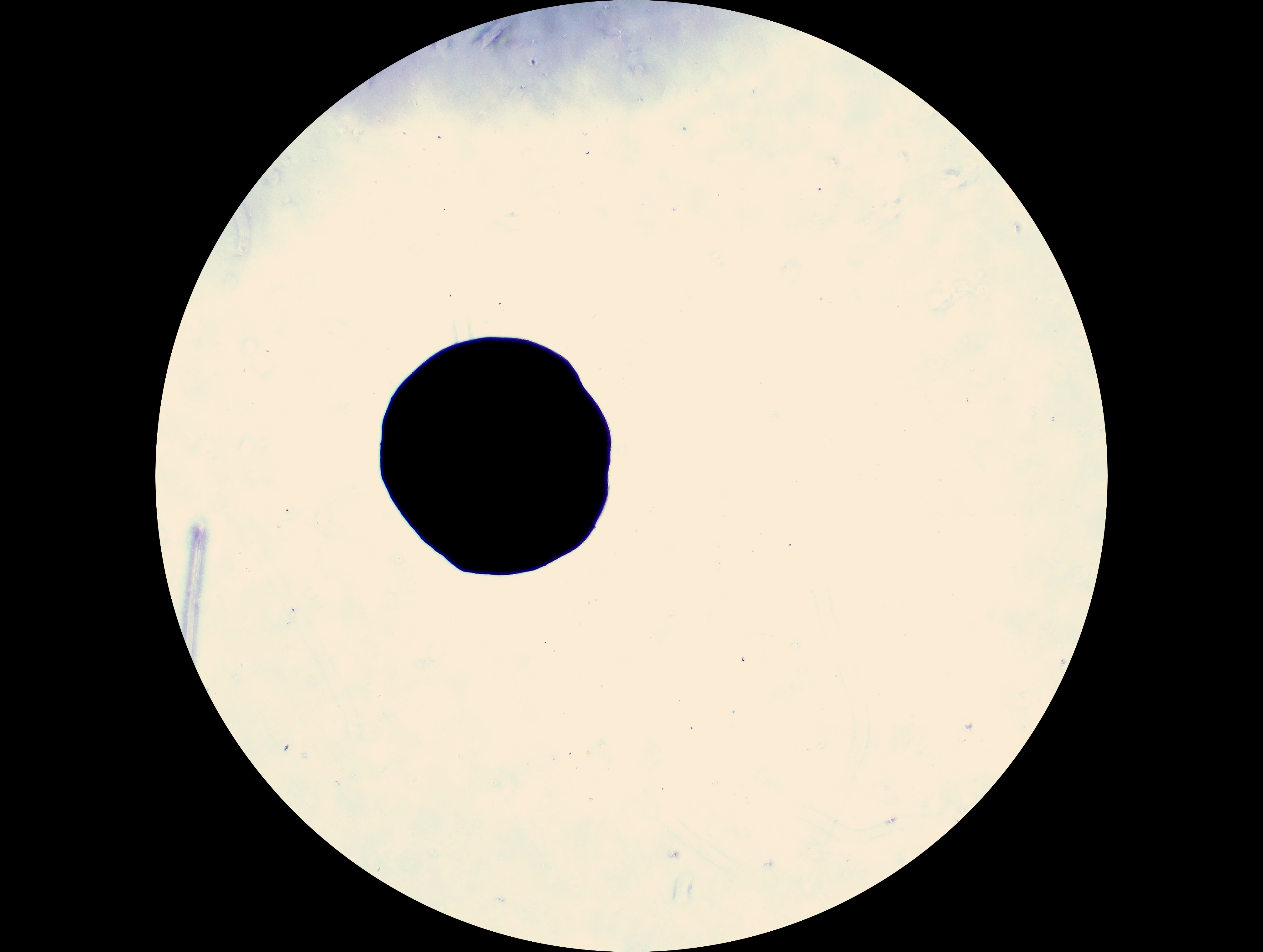

Supplement: Supplementary file 11 — Source data Fig. 3 [file 44319_2025_619_MOESM11_ESM.zip › Figure 3/C,D,F,G/Raw images_mask/OS_day90/MN 12C1 B C12 D90 2x/day 90_0007.jpg]

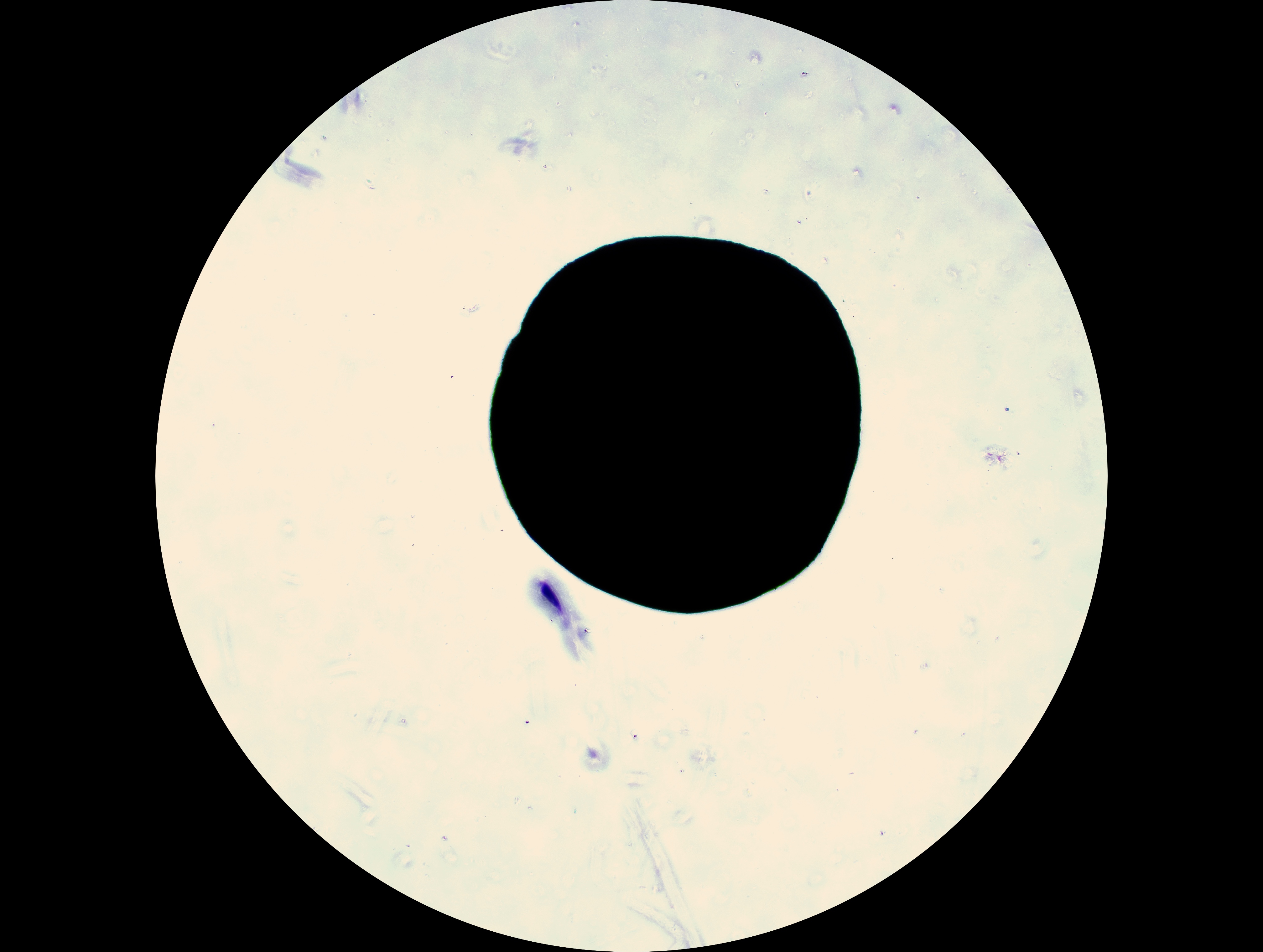

Supplement: Supplementary file 11 — Source data Fig. 3 [file 44319_2025_619_MOESM11_ESM.zip › Figure 3/C,D,F,G/Raw images_mask/OS_day90/MN 12C1 B C12 D90 2x/day 90_0013.jpg]

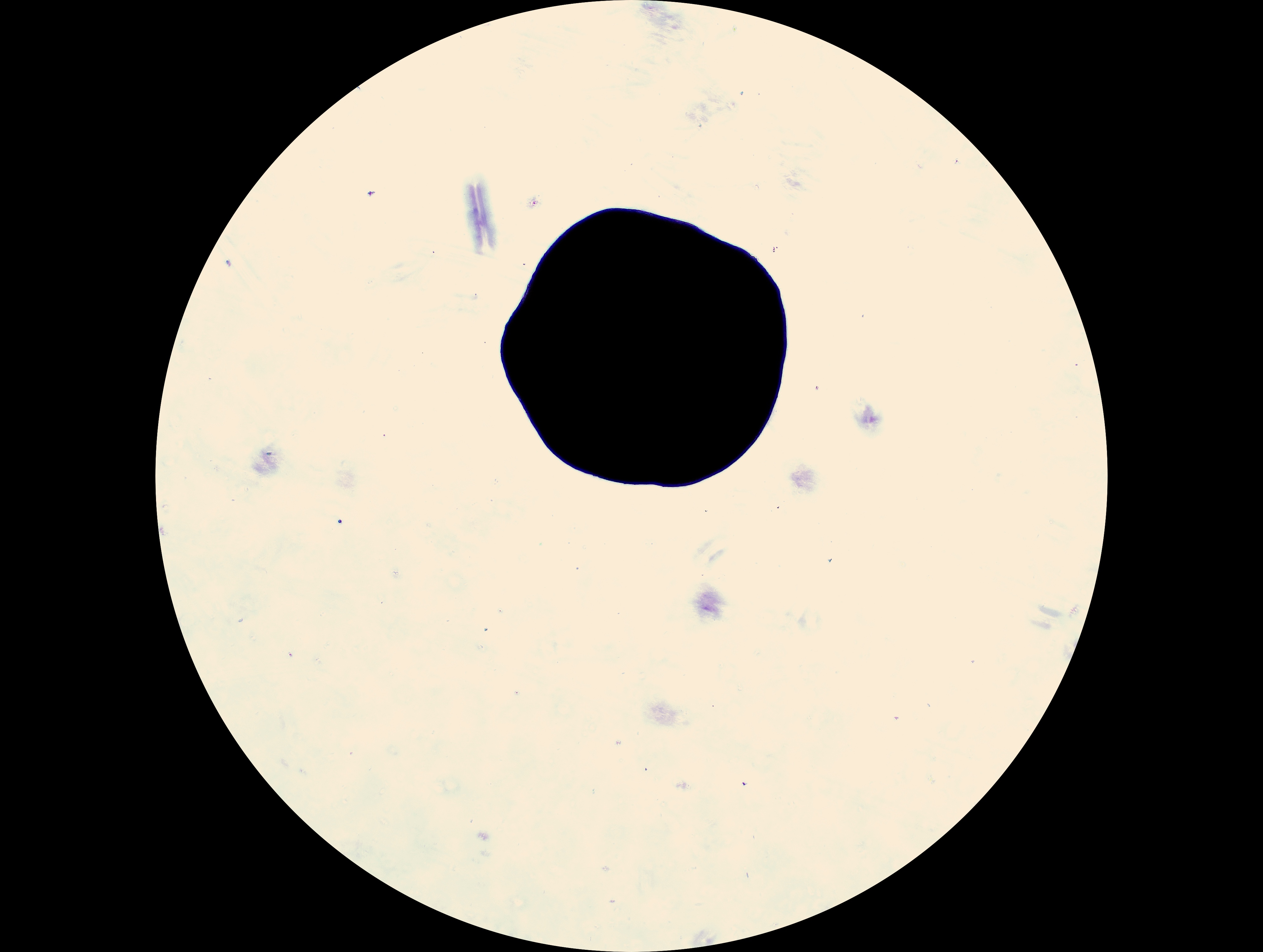

Supplement: Supplementary file 11 — Source data Fig. 3 [file 44319_2025_619_MOESM11_ESM.zip › Figure 3/C,D,F,G/Raw images_mask/OS_day90/MN 12C1 B C12 D90 2x/day 90_0012.jpg]

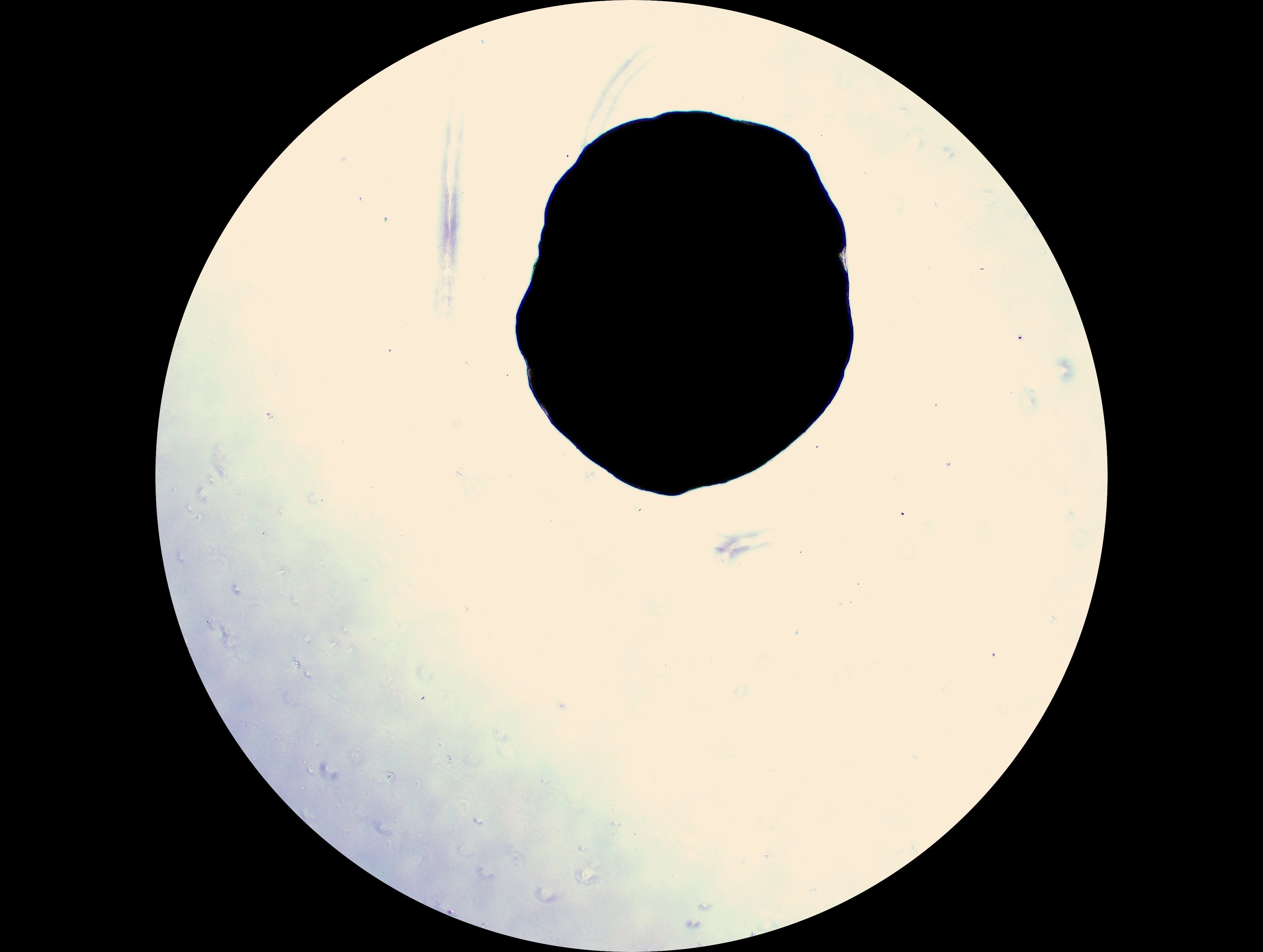

Supplement: Supplementary file 11 — Source data Fig. 3 [file 44319_2025_619_MOESM11_ESM.zip › Figure 3/C,D,F,G/Raw images_mask/OS_day90/MN 12C1 B C12 D90 2x/day 90_0006.jpg]

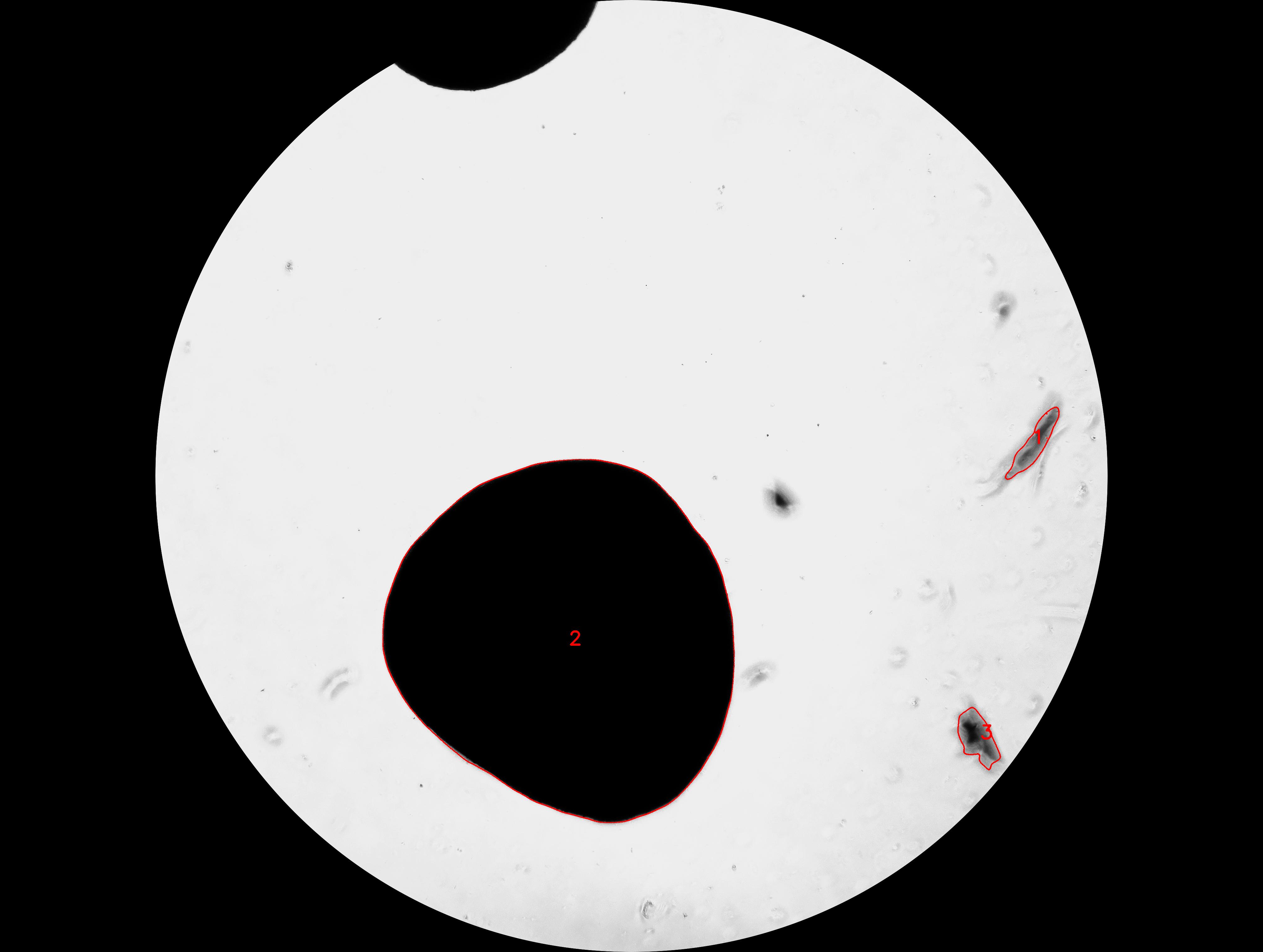

Supplement: Supplementary file 11 — Source data Fig. 3 [file 44319_2025_619_MOESM11_ESM.zip › Figure 3/C,D,F,G/Raw images_mask/OS_day90/MN 12C1 B C12 D90 2x/R_day 90_0009.jpg]

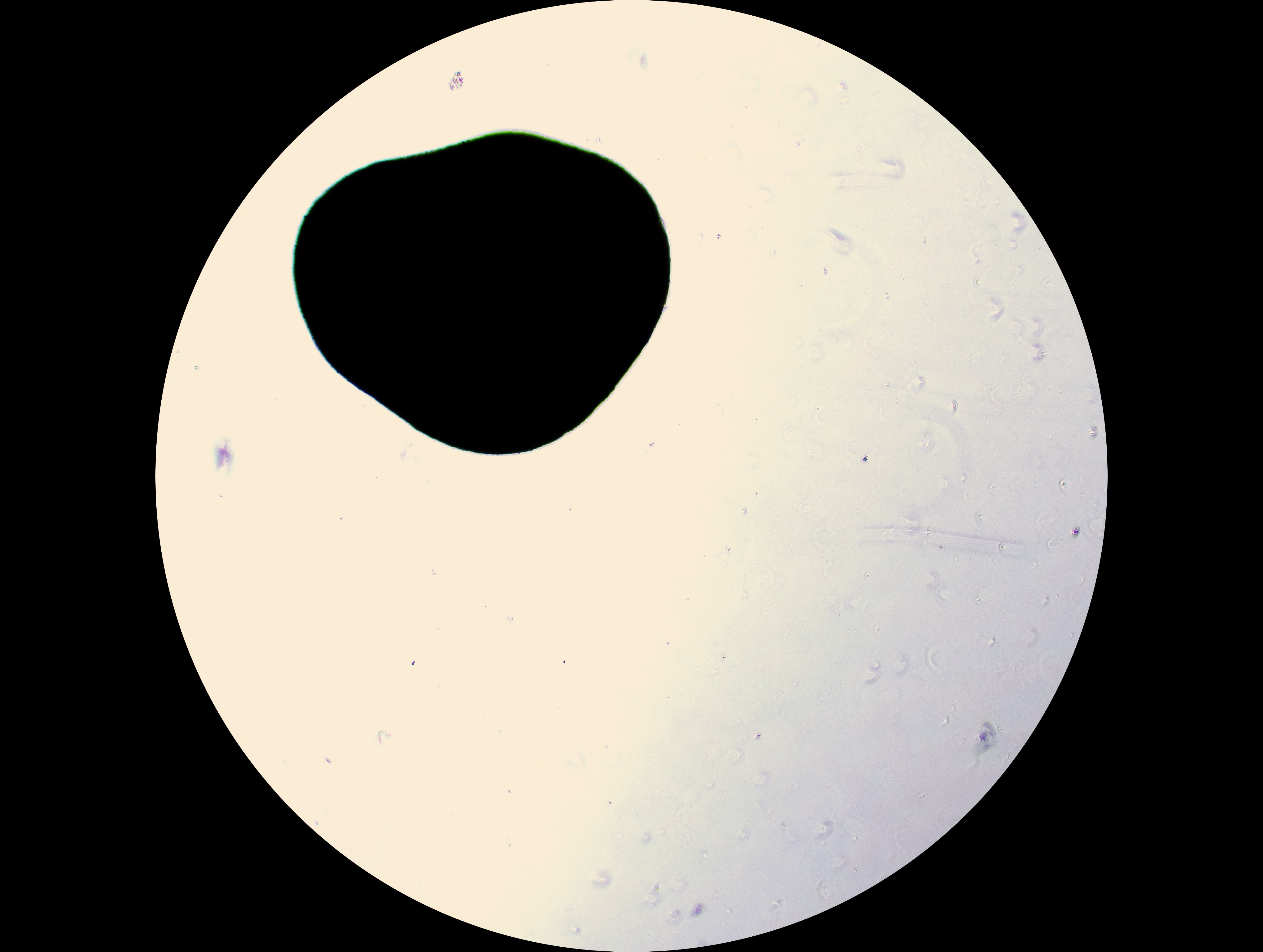

Supplement: Supplementary file 11 — Source data Fig. 3 [file 44319_2025_619_MOESM11_ESM.zip › Figure 3/C,D,F,G/Raw images_mask/OS_day90/MN 12C1 B C12 D90 2x/day 90_0010.jpg]

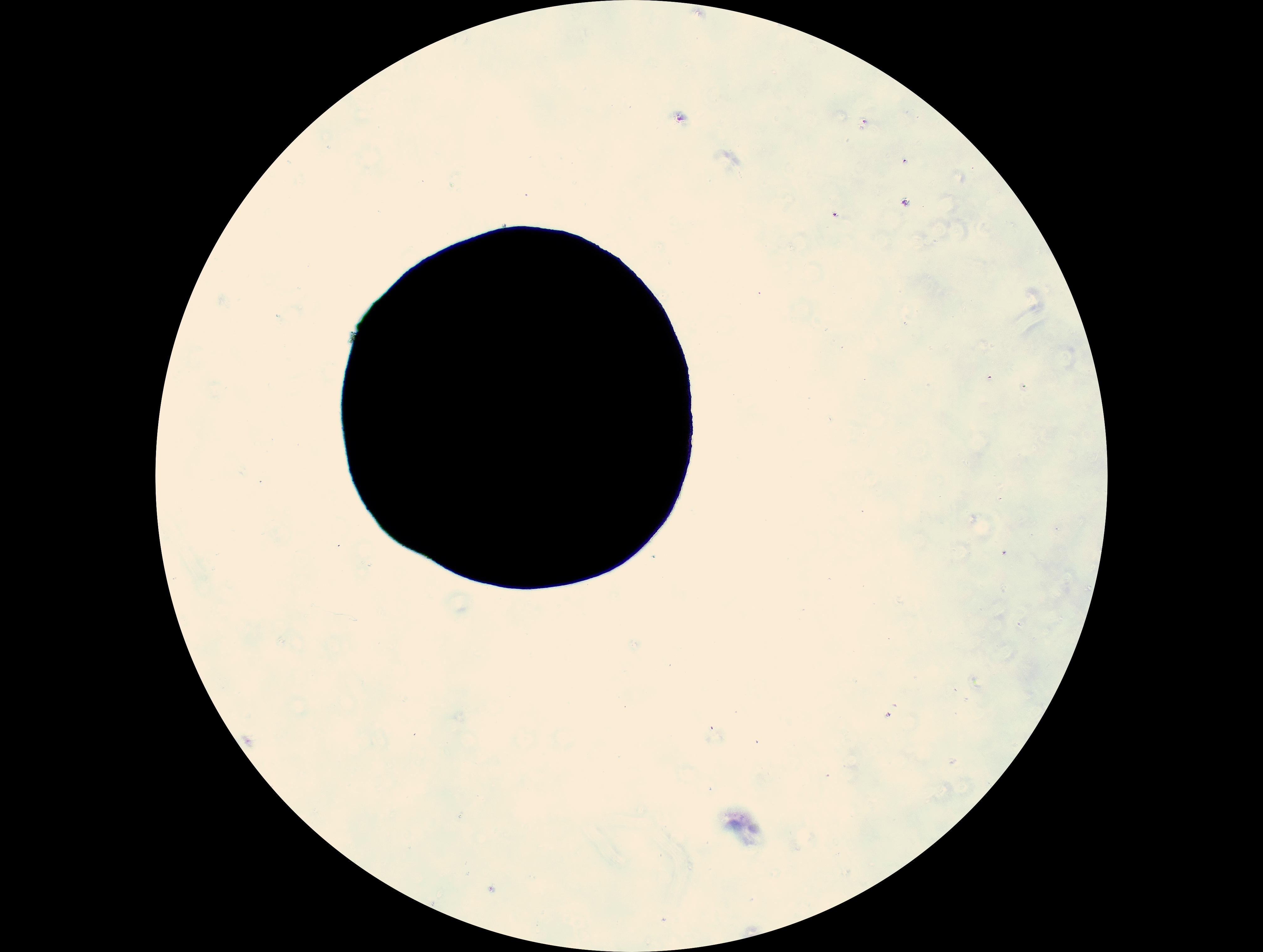

Supplement: Supplementary file 11 — Source data Fig. 3 [file 44319_2025_619_MOESM11_ESM.zip › Figure 3/C,D,F,G/Raw images_mask/OS_day90/MN 12C1 B C12 D90 2x/day 90_0004.jpg]

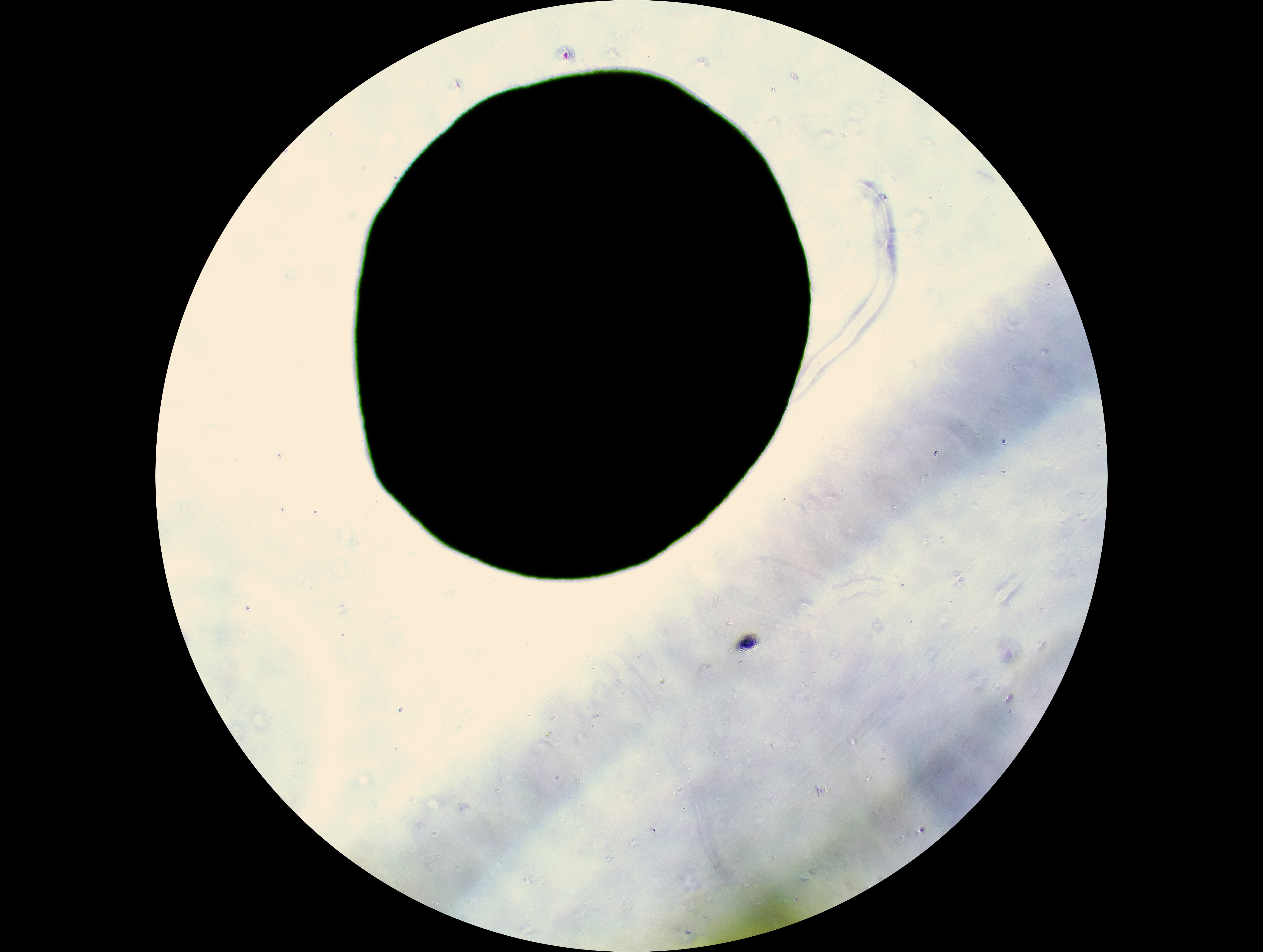

Supplement: Supplementary file 11 — Source data Fig. 3 [file 44319_2025_619_MOESM11_ESM.zip › Figure 3/C,D,F,G/Raw images_mask/OS_day90/MN 12C1 B C12 D90 2x/day 90_0005.jpg]

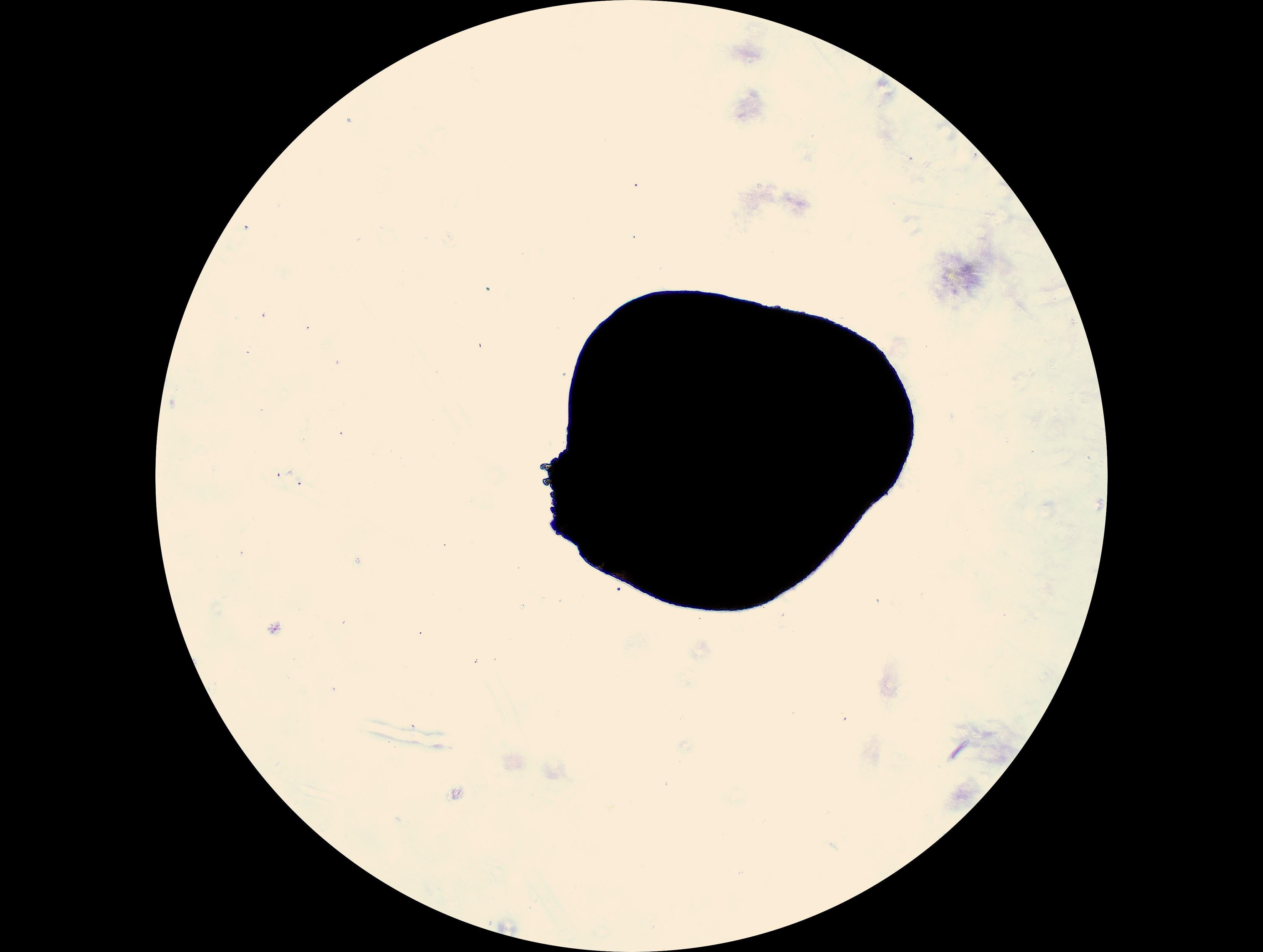

Supplement: Supplementary file 11 — Source data Fig. 3 [file 44319_2025_619_MOESM11_ESM.zip › Figure 3/C,D,F,G/Raw images_mask/OS_day90/MN 12C1 B C12 D90 2x/day 90_0011.jpg]

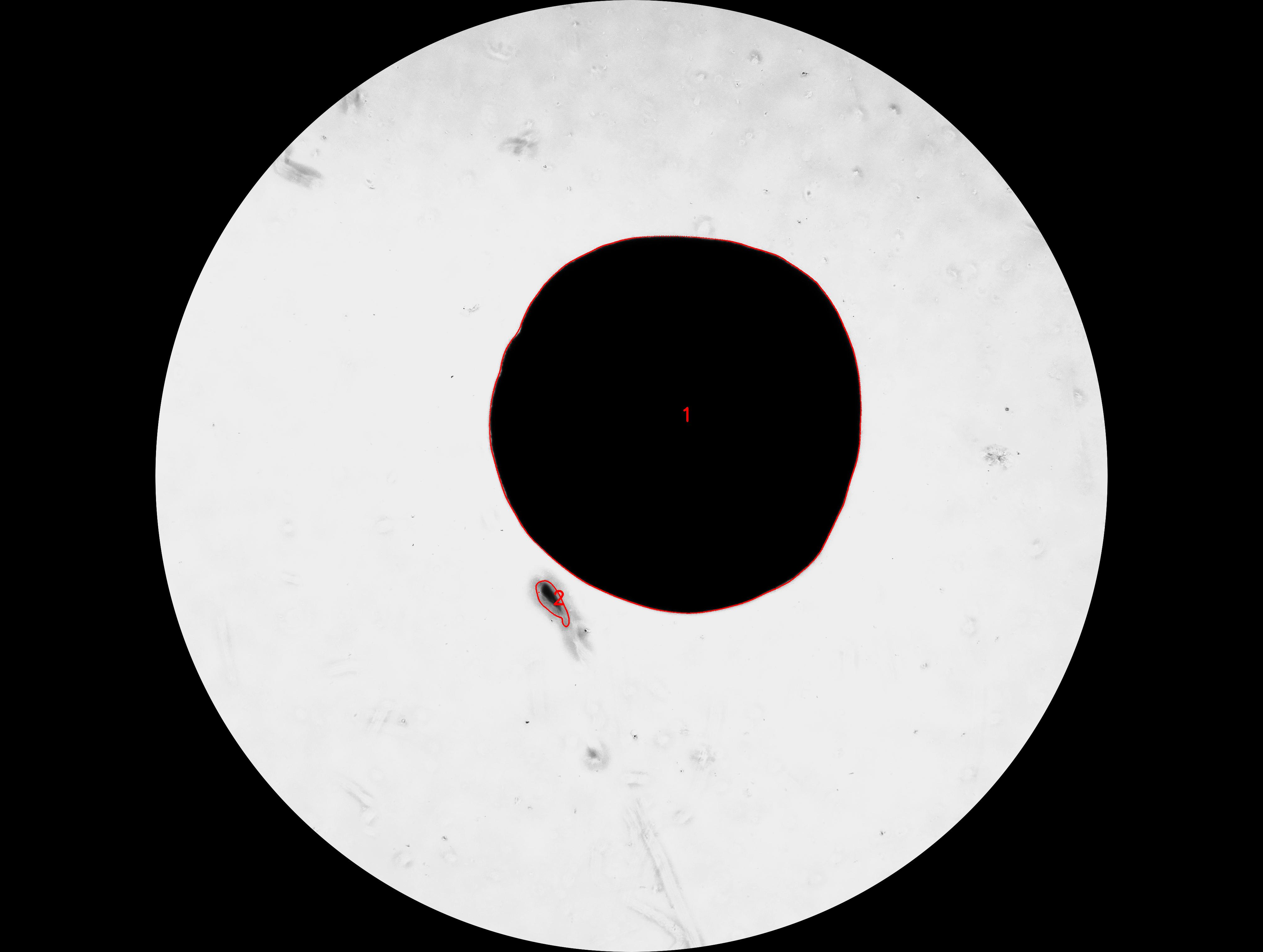

Supplement: Supplementary file 11 — Source data Fig. 3 [file 44319_2025_619_MOESM11_ESM.zip › Figure 3/C,D,F,G/Raw images_mask/OS_day90/MN 12C1 B C12 D90 2x/R_day 90_0013.jpg]

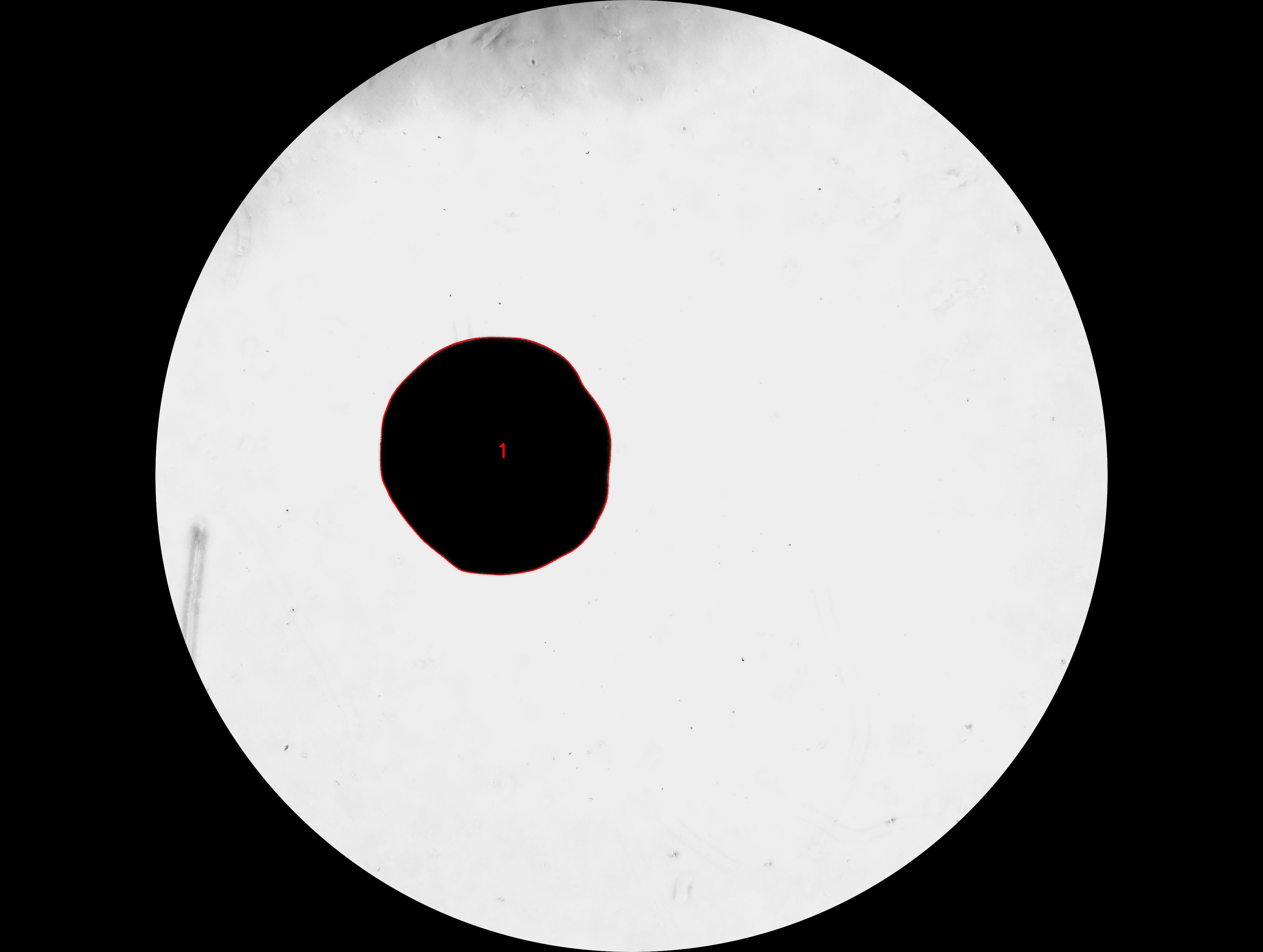

Supplement: Supplementary file 11 — Source data Fig. 3 [file 44319_2025_619_MOESM11_ESM.zip › Figure 3/C,D,F,G/Raw images_mask/OS_day90/MN 12C1 B C12 D90 2x/R_day 90_0007.jpg]

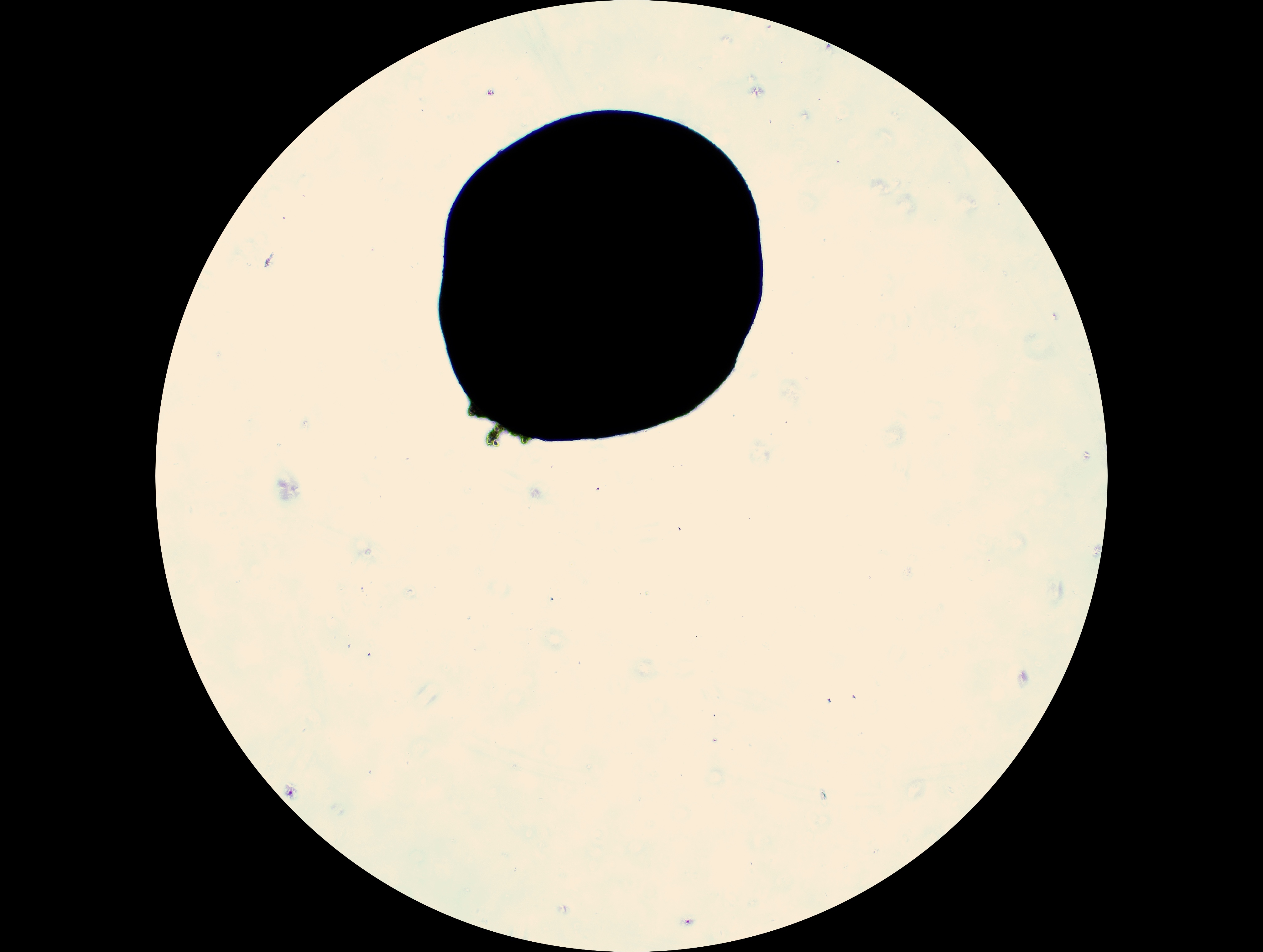

Supplement: Supplementary file 11 — Source data Fig. 3 [file 44319_2025_619_MOESM11_ESM.zip › Figure 3/C,D,F,G/Raw images_mask/OS_day90/MN 12C1 B C12 D90 2x/day 90_0008.jpg]

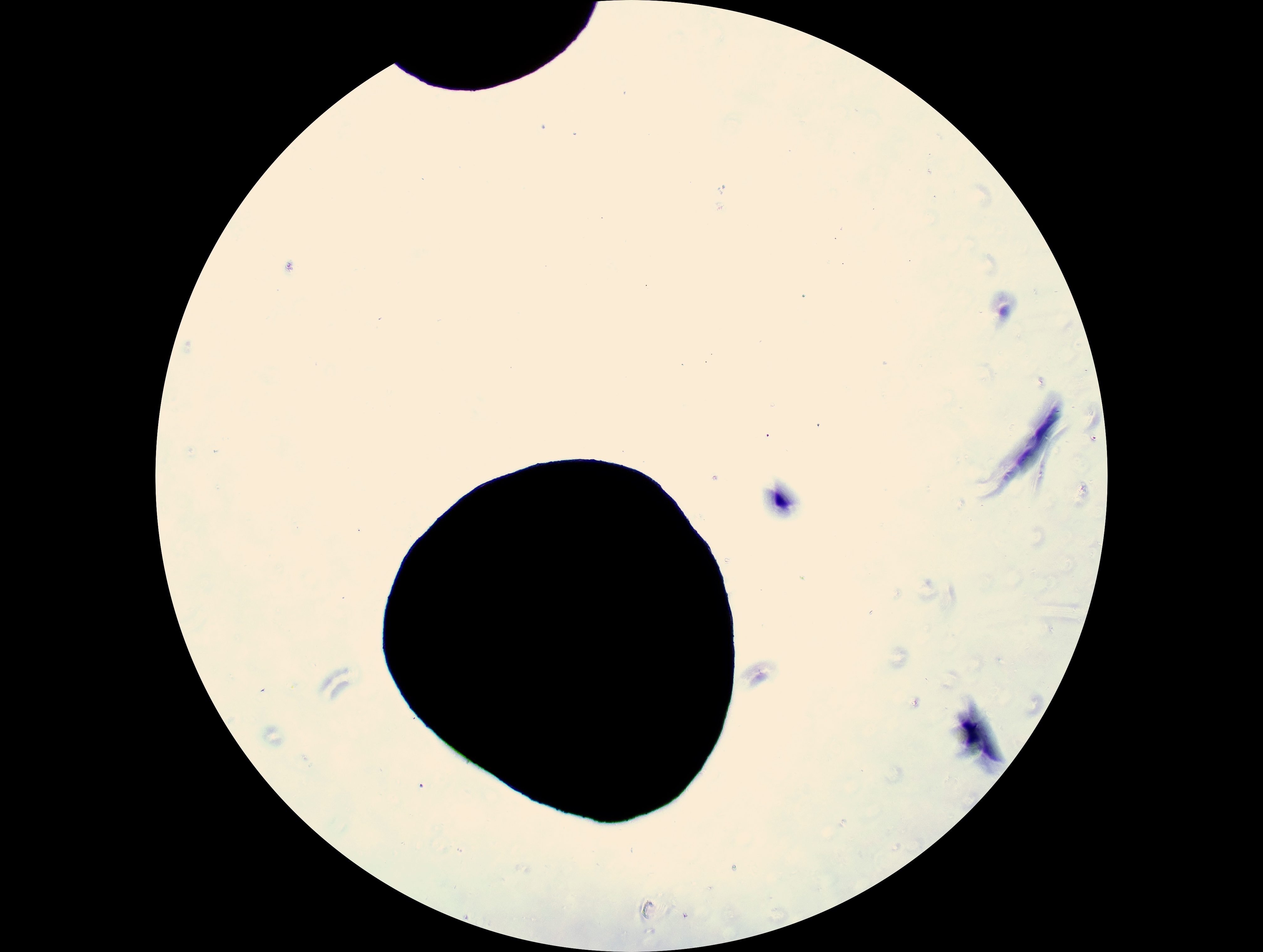

Supplement: Supplementary file 11 — Source data Fig. 3 [file 44319_2025_619_MOESM11_ESM.zip › Figure 3/C,D,F,G/Raw images_mask/OS_day90/MN 12C1 B C12 D90 2x/day 90_0009.jpg]

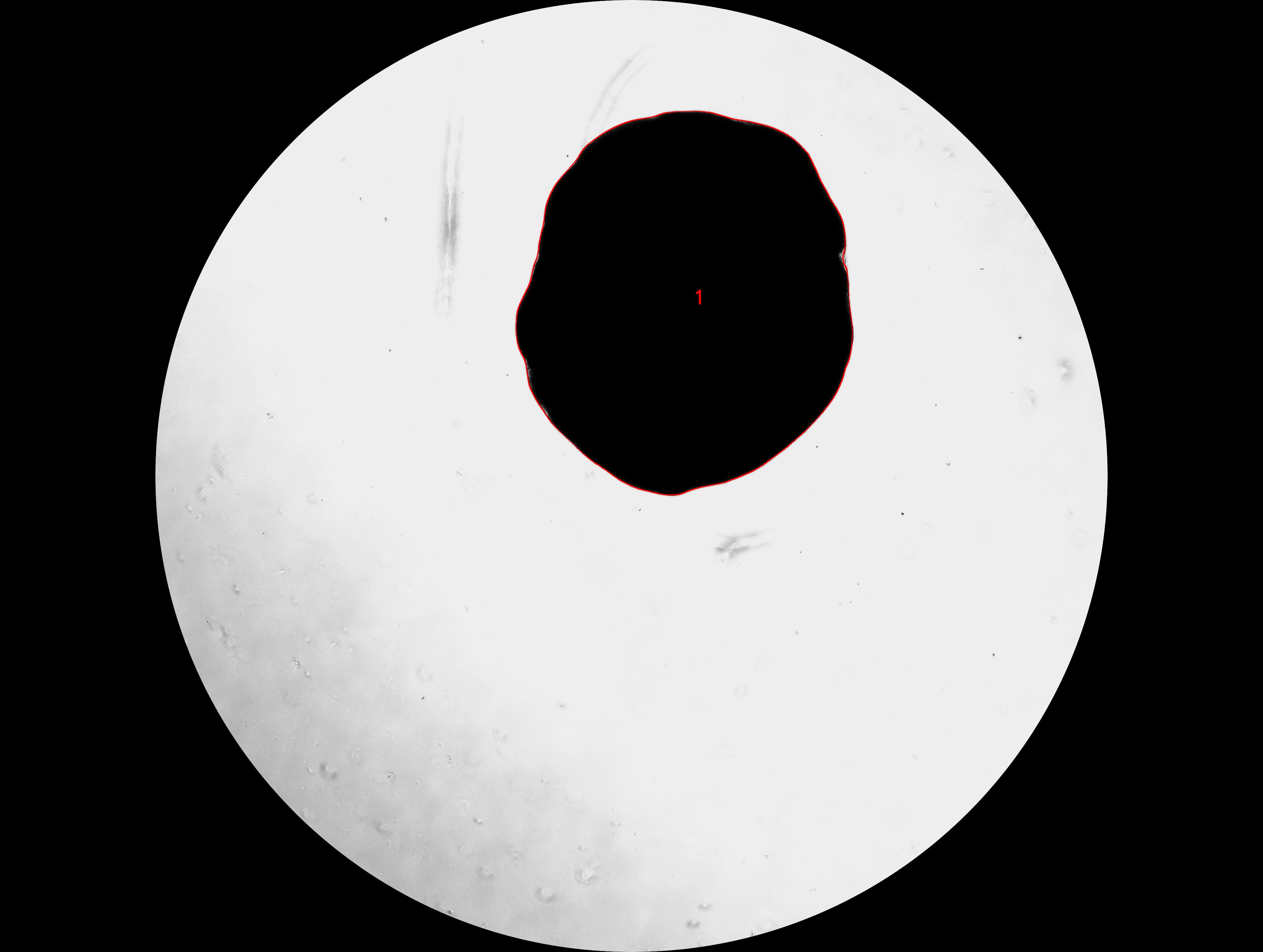

Supplement: Supplementary file 11 — Source data Fig. 3 [file 44319_2025_619_MOESM11_ESM.zip › Figure 3/C,D,F,G/Raw images_mask/OS_day90/MN 12C1 B C12 D90 2x/R_day 90_0006.jpg]

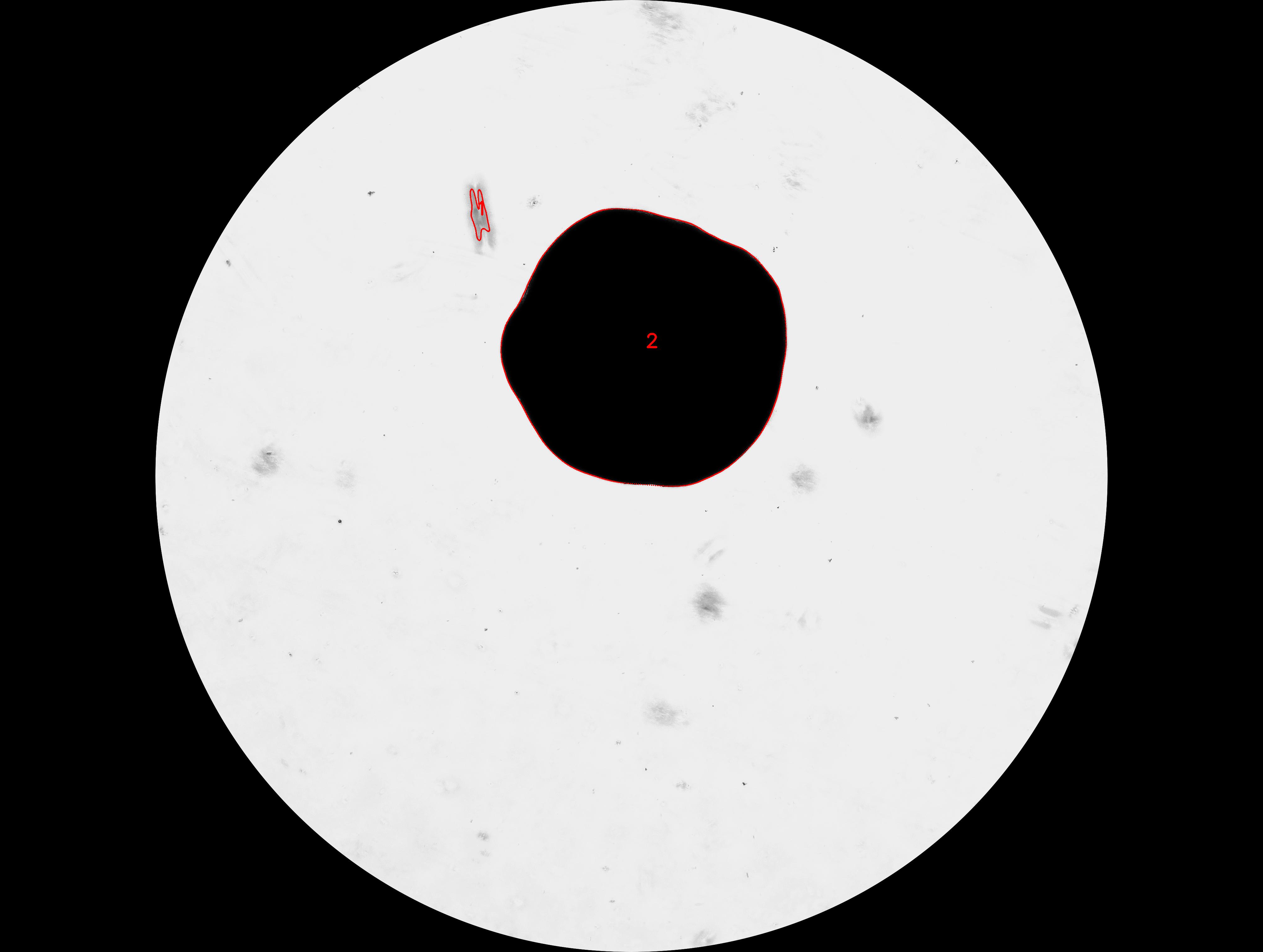

Supplement: Supplementary file 11 — Source data Fig. 3 [file 44319_2025_619_MOESM11_ESM.zip › Figure 3/C,D,F,G/Raw images_mask/OS_day90/MN 12C1 B C12 D90 2x/R_day 90_0012.jpg]

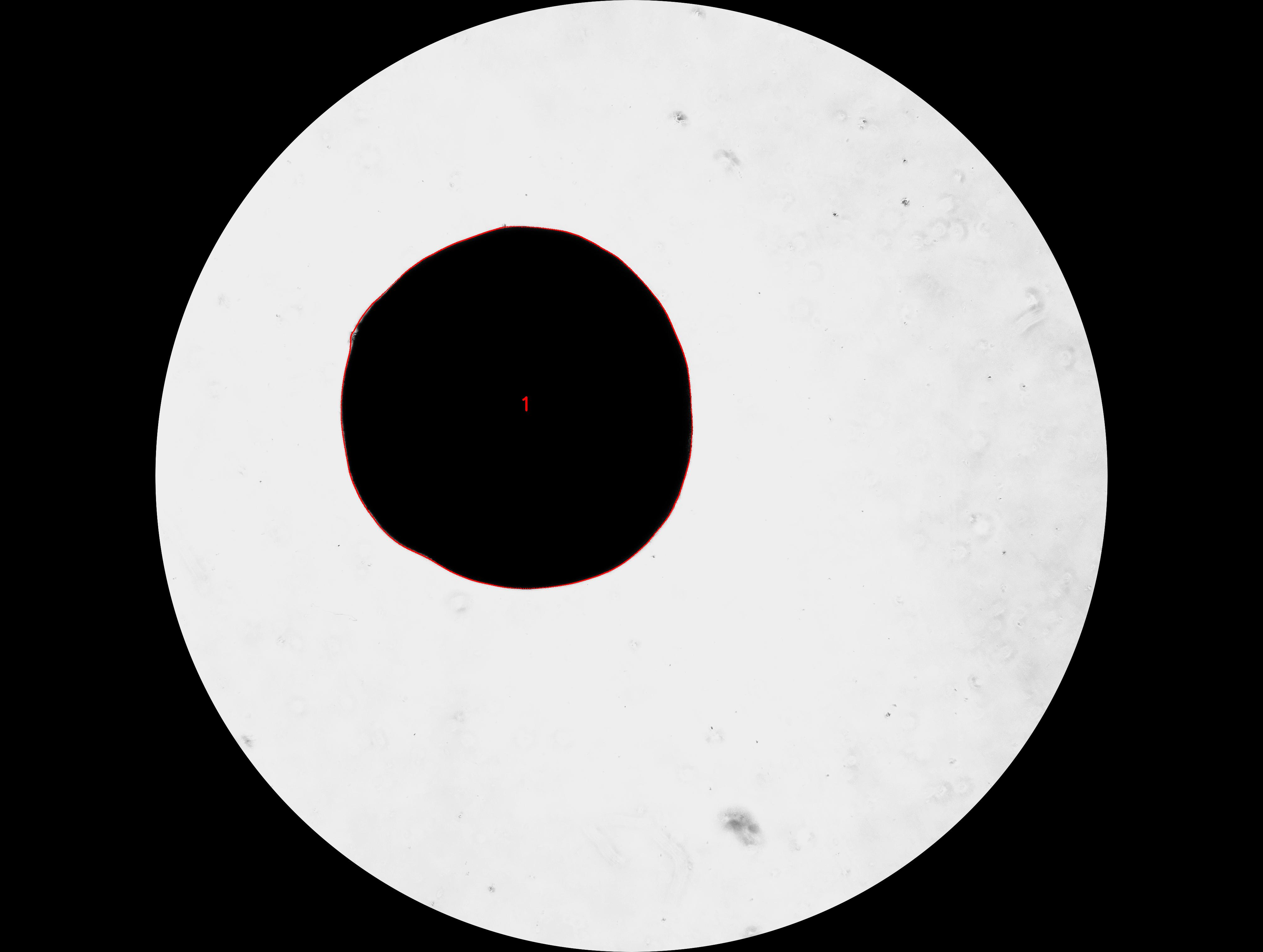

Supplement: Supplementary file 11 — Source data Fig. 3 [file 44319_2025_619_MOESM11_ESM.zip › Figure 3/C,D,F,G/Raw images_mask/OS_day90/MN 12C1 B C12 D90 2x/R_day 90_0004.jpg]

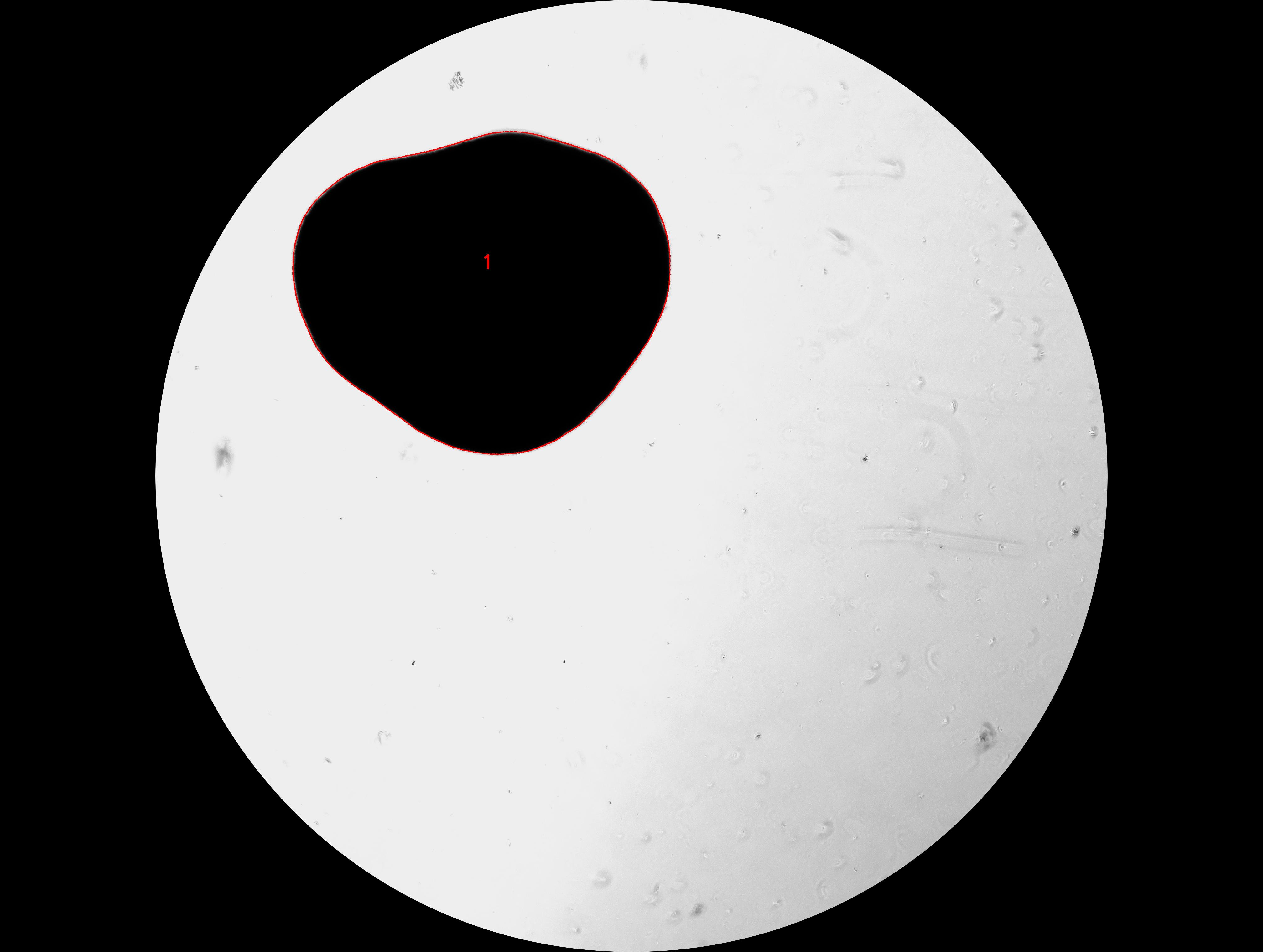

Supplement: Supplementary file 11 — Source data Fig. 3 [file 44319_2025_619_MOESM11_ESM.zip › Figure 3/C,D,F,G/Raw images_mask/OS_day90/MN 12C1 B C12 D90 2x/R_day 90_0010.jpg]

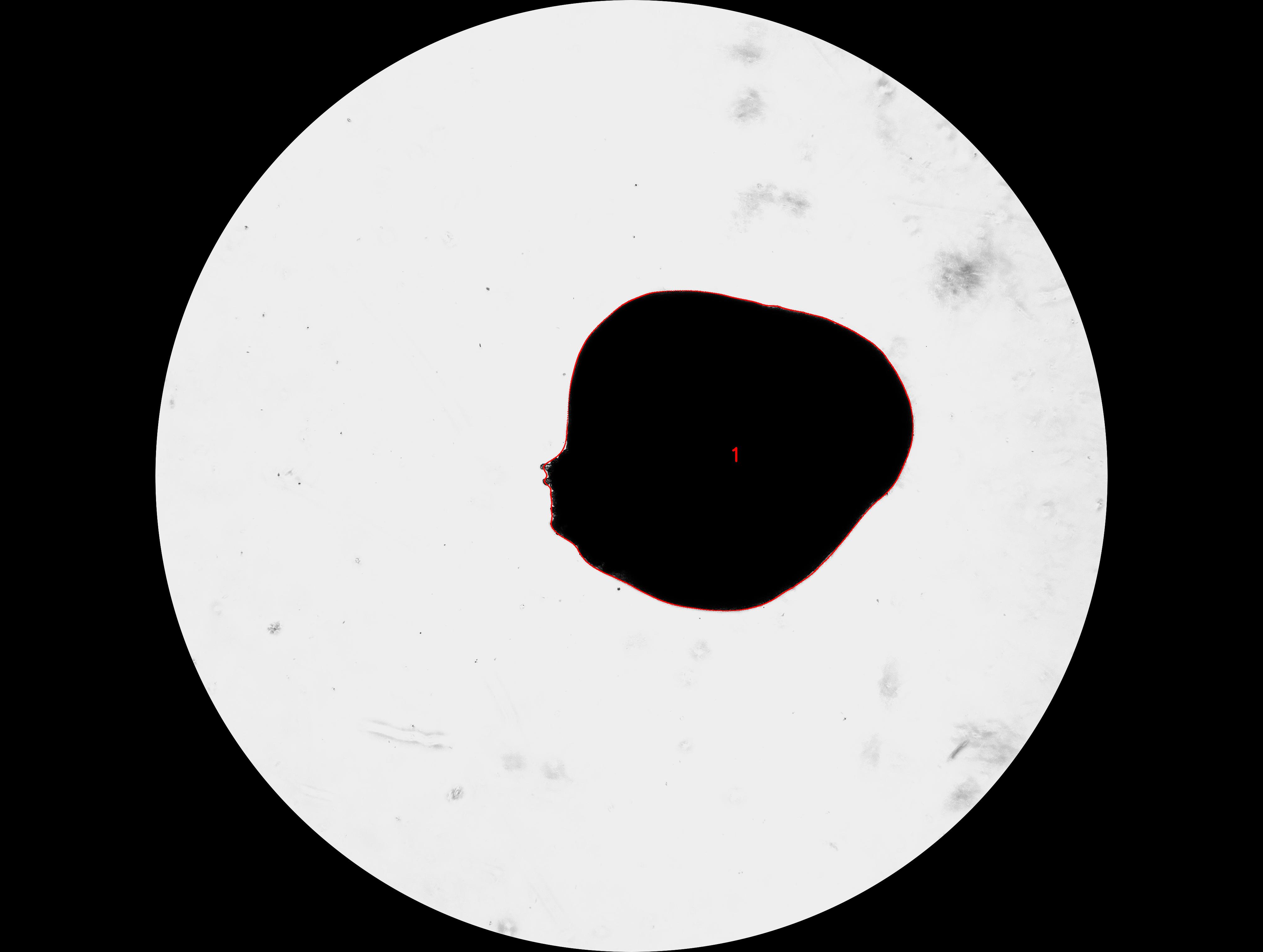

Supplement: Supplementary file 11 — Source data Fig. 3 [file 44319_2025_619_MOESM11_ESM.zip › Figure 3/C,D,F,G/Raw images_mask/OS_day90/MN 12C1 B C12 D90 2x/R_day 90_0011.jpg]

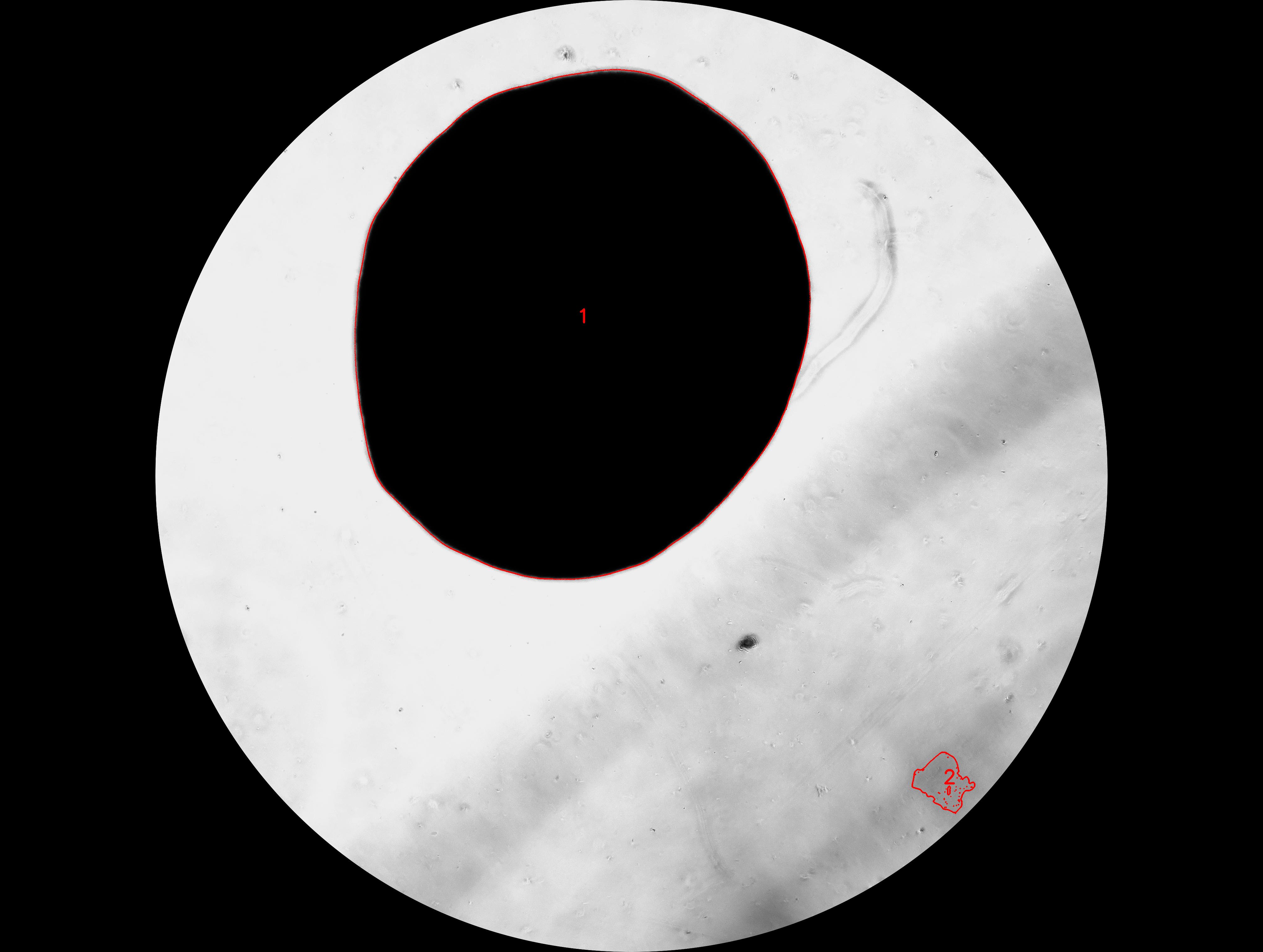

Supplement: Supplementary file 11 — Source data Fig. 3 [file 44319_2025_619_MOESM11_ESM.zip › Figure 3/C,D,F,G/Raw images_mask/OS_day90/MN 12C1 B C12 D90 2x/R_day 90_0005.jpg]

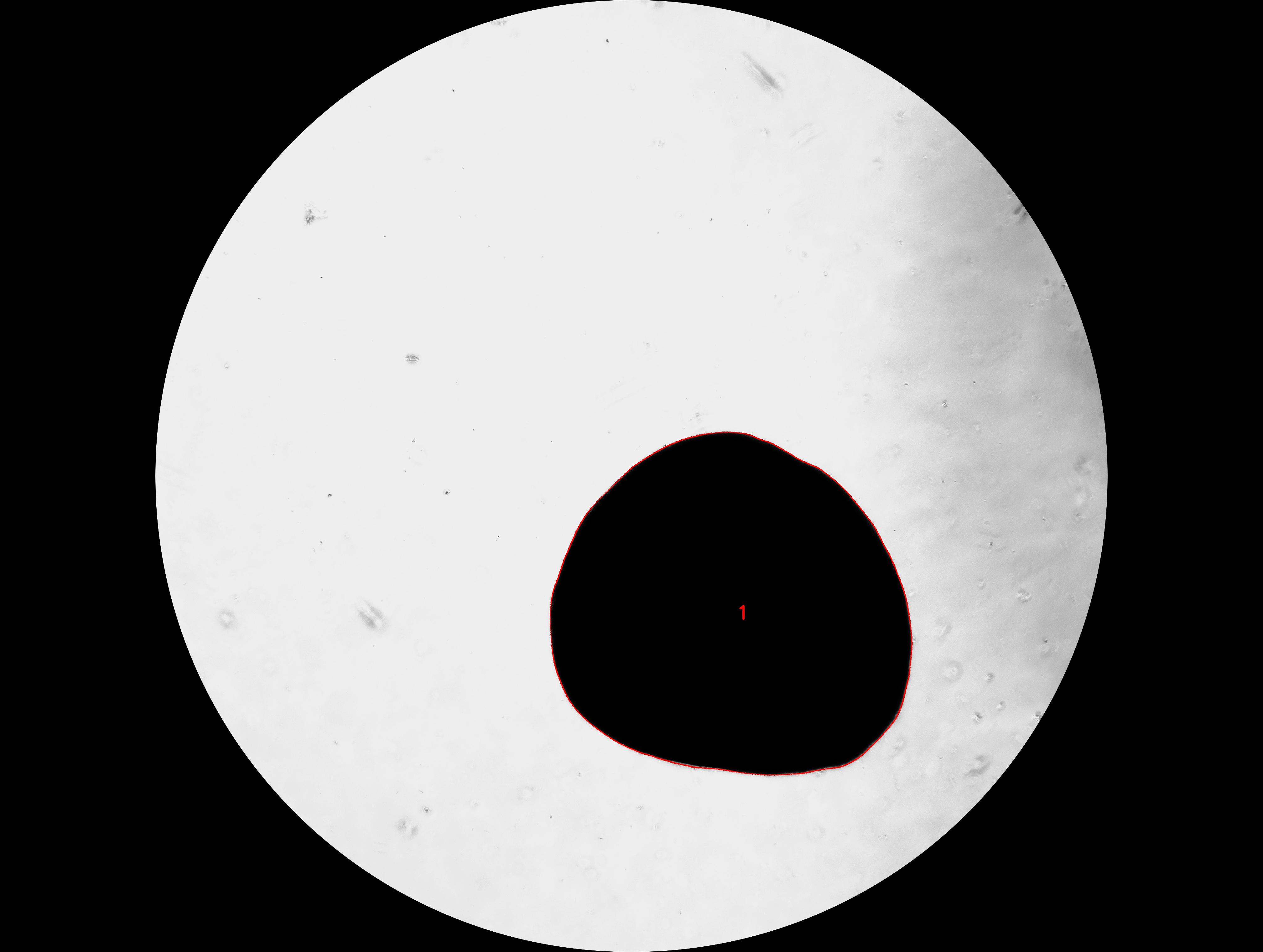

Supplement: Supplementary file 11 — Source data Fig. 3 [file 44319_2025_619_MOESM11_ESM.zip › Figure 3/C,D,F,G/Raw images_mask/OS_day90/MN 12C1 B C12 D90 2x/R_day 90_0015.jpg]

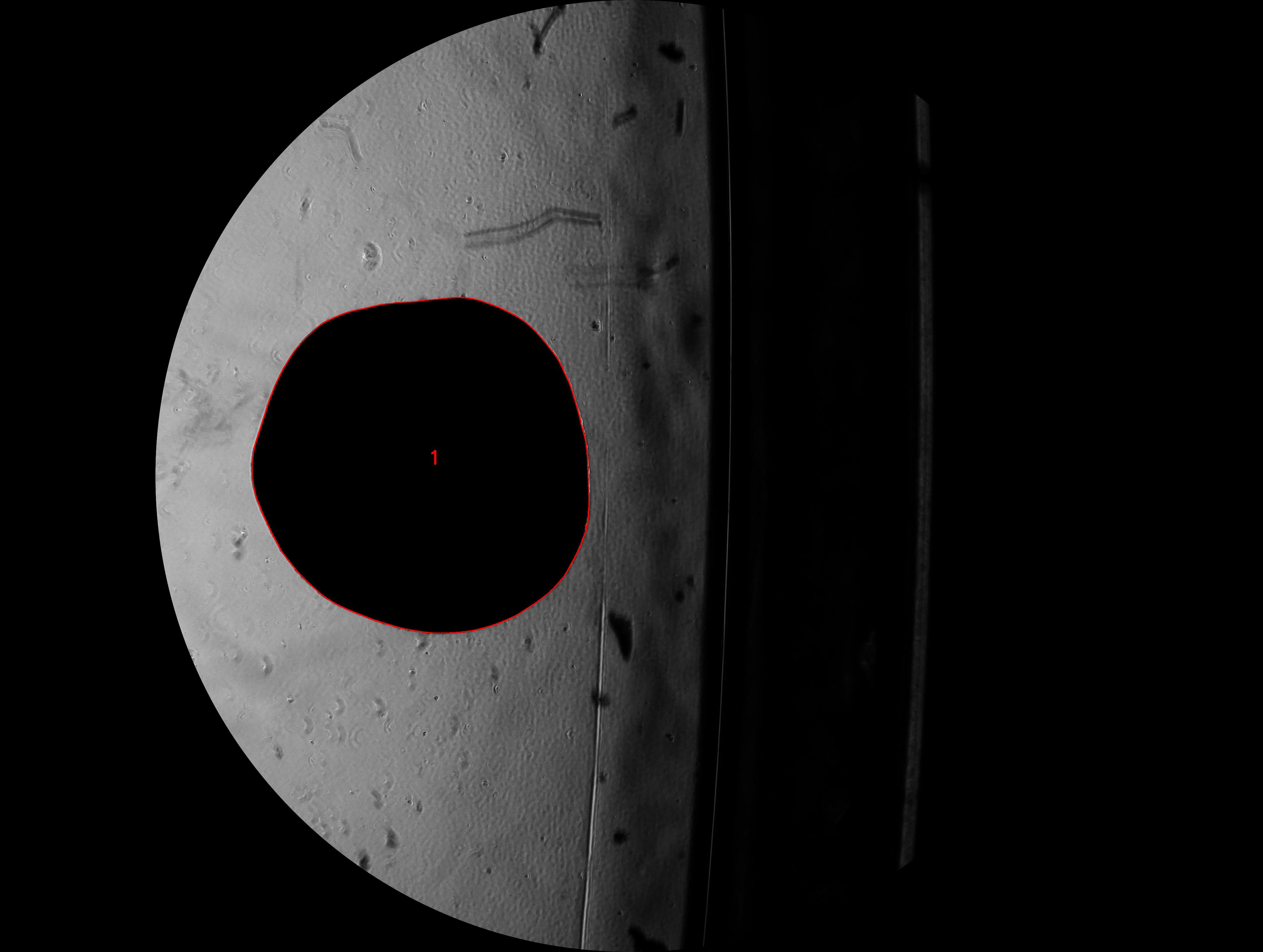

Supplement: Supplementary file 11 — Source data Fig. 3 [file 44319_2025_619_MOESM11_ESM.zip › Figure 3/C,D,F,G/Raw images_mask/OS_day90/MN 12C1 B C12 D90 2x/R_day 90_0014.jpg]

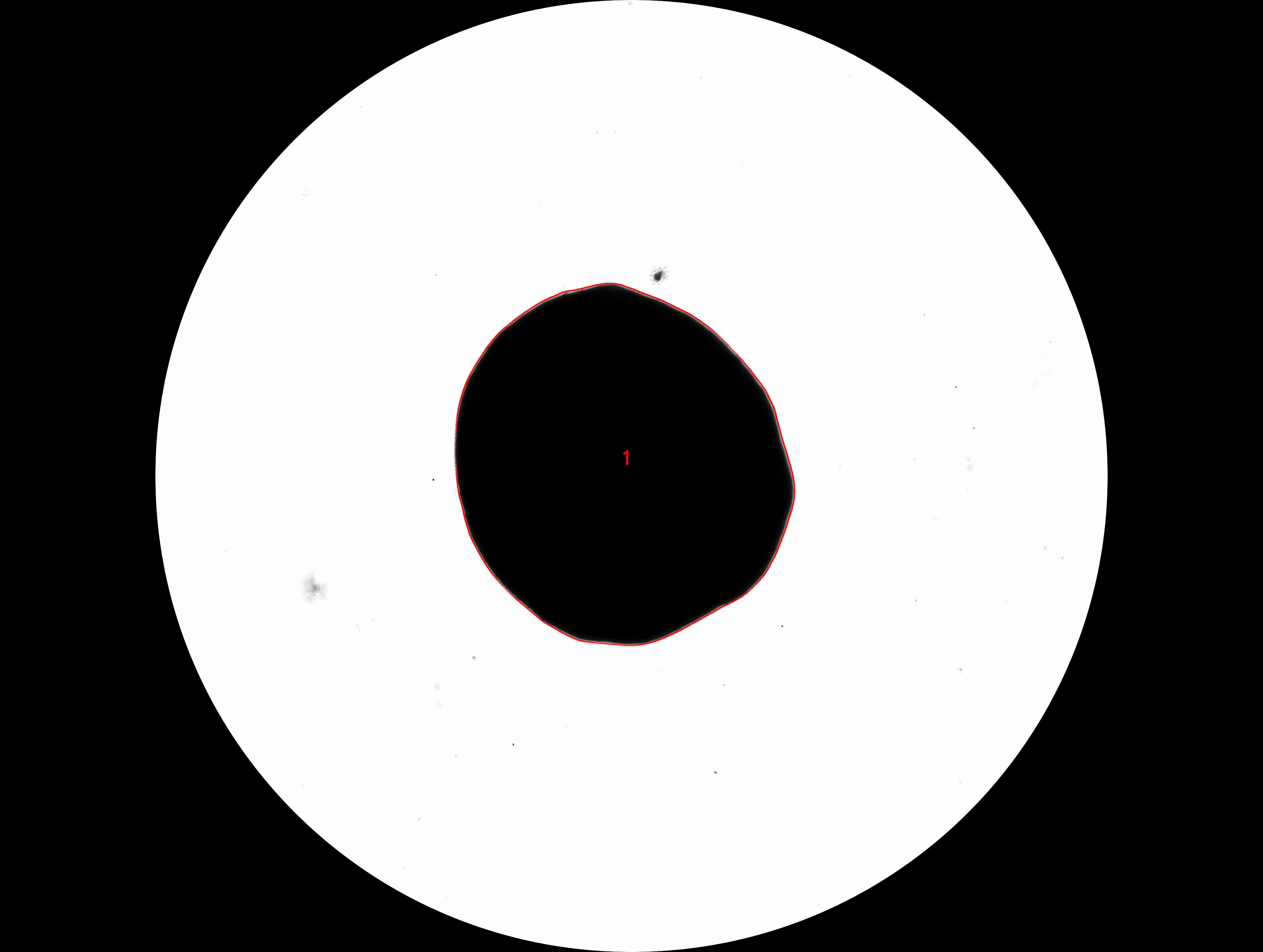

Supplement: Supplementary file 11 — Source data Fig. 3 [file 44319_2025_619_MOESM11_ESM.zip › Figure 3/C,D,F,G/Raw images_mask/OS_day90/MN 12C1 B C12 D90 2x/R_day 90_0000.jpg]

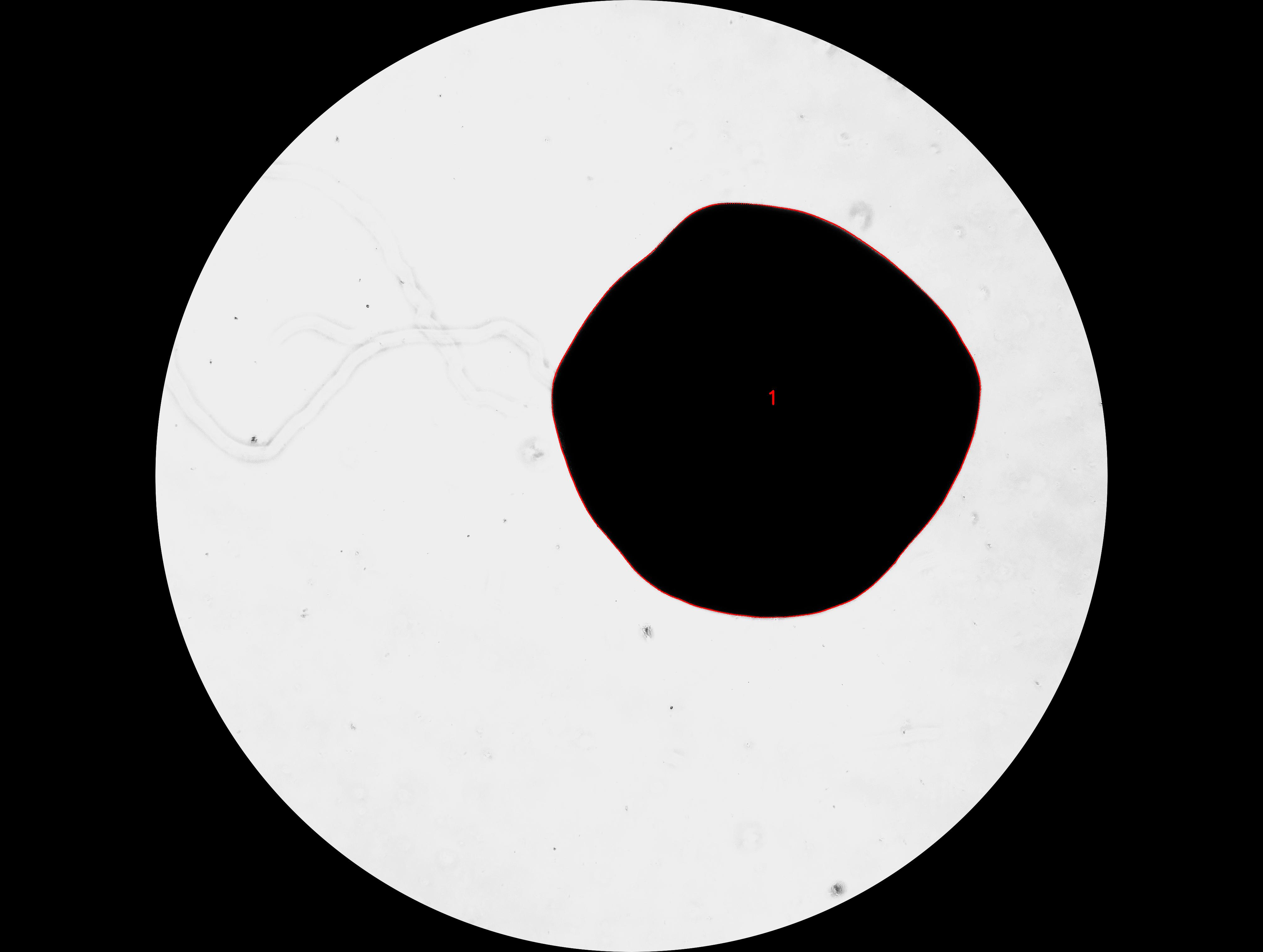

Supplement: Supplementary file 11 — Source data Fig. 3 [file 44319_2025_619_MOESM11_ESM.zip › Figure 3/C,D,F,G/Raw images_mask/OS_day90/MN 12C1 B C12 D90 2x/R_day 90_0002.jpg]

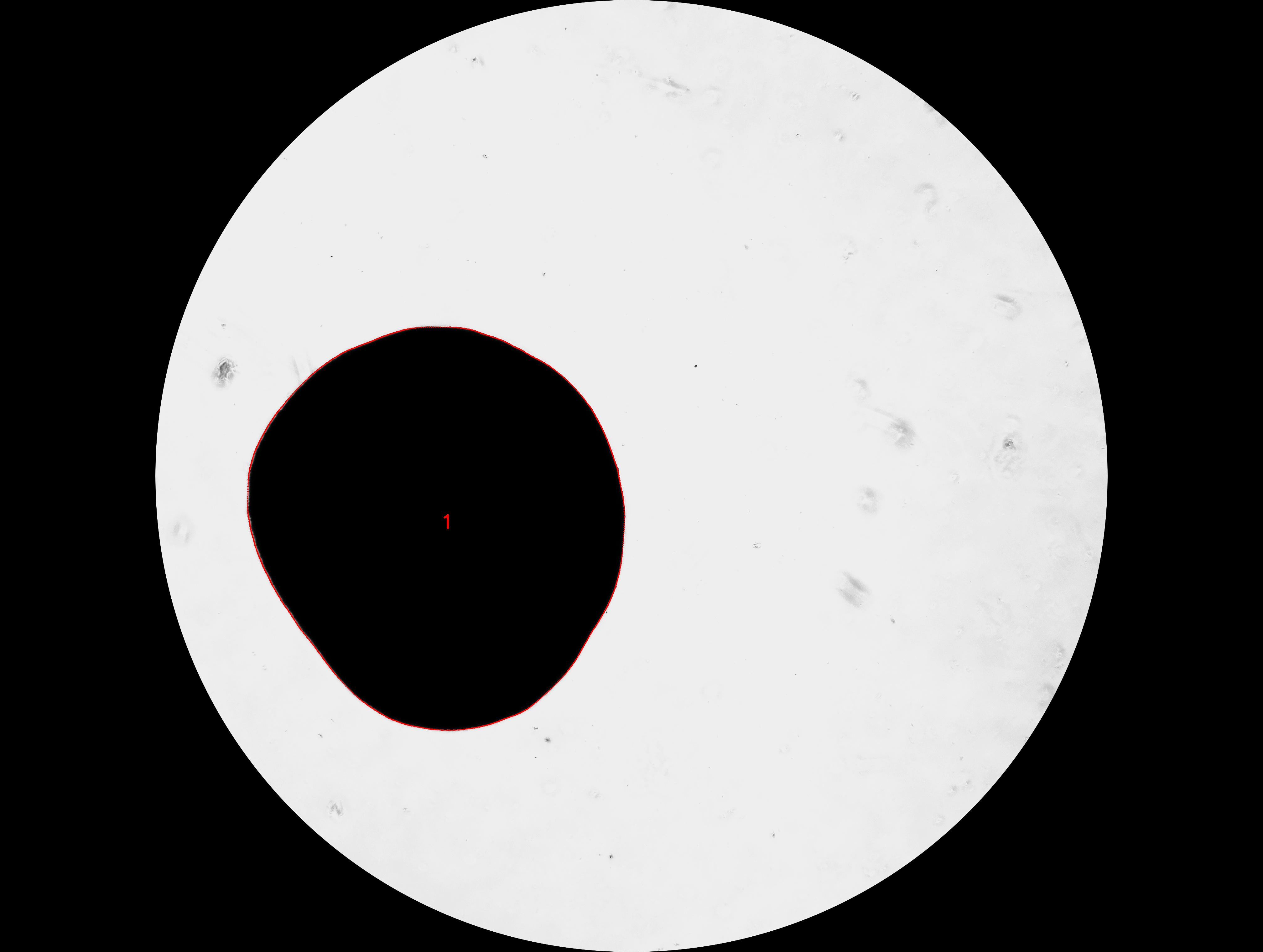

Supplement: Supplementary file 11 — Source data Fig. 3 [file 44319_2025_619_MOESM11_ESM.zip › Figure 3/C,D,F,G/Raw images_mask/OS_day90/MN 12C1 B C12 D90 2x/R_day 90_0003.jpg]

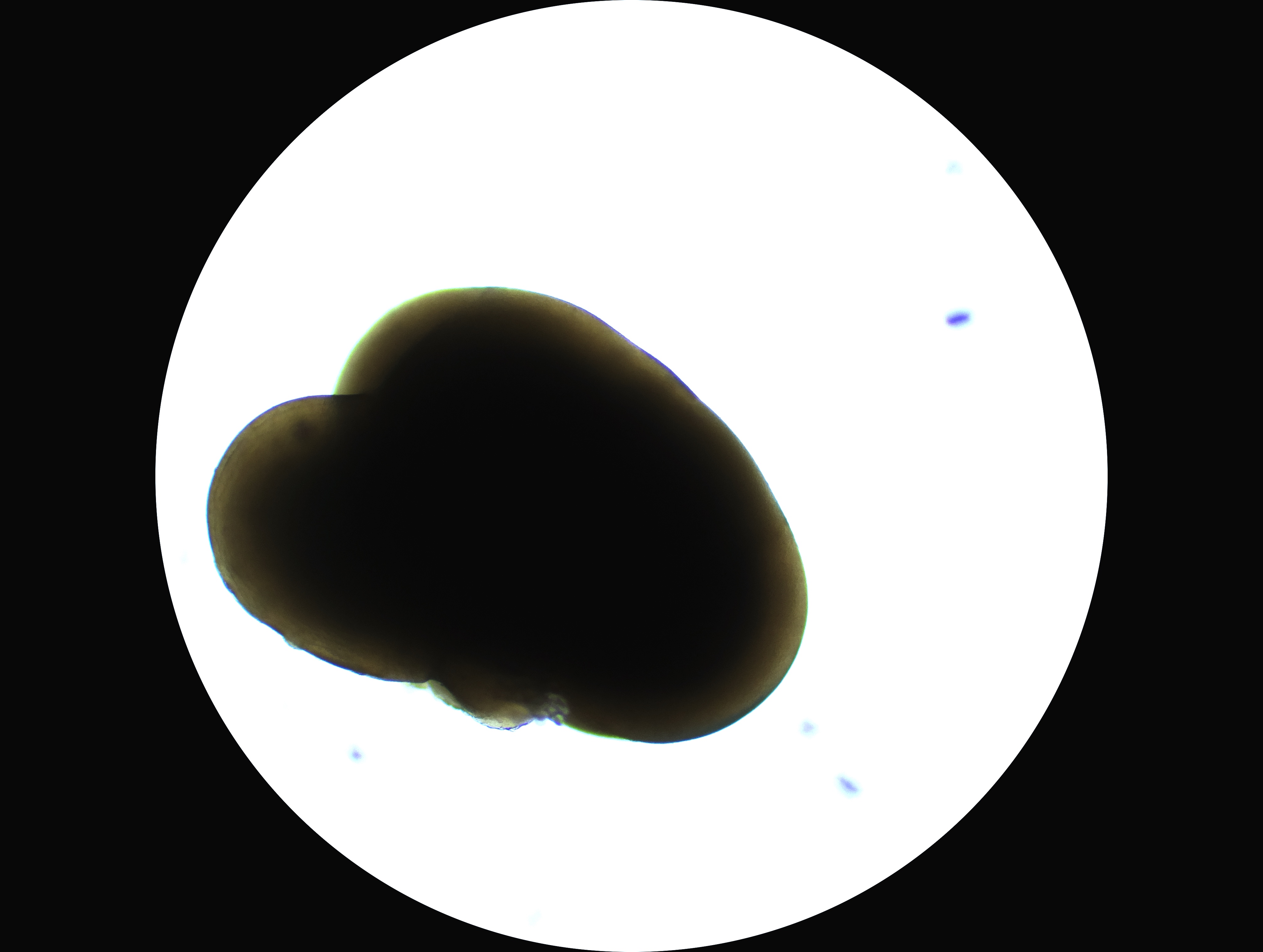

Supplement: Supplementary file 11 — Source data Fig. 3 [file 44319_2025_619_MOESM11_ESM.zip › Figure 3/C,D,F,G/Raw images_mask/OS_day90/MN 11C1 B C3 D90 2x/Day90_0008.jpg]

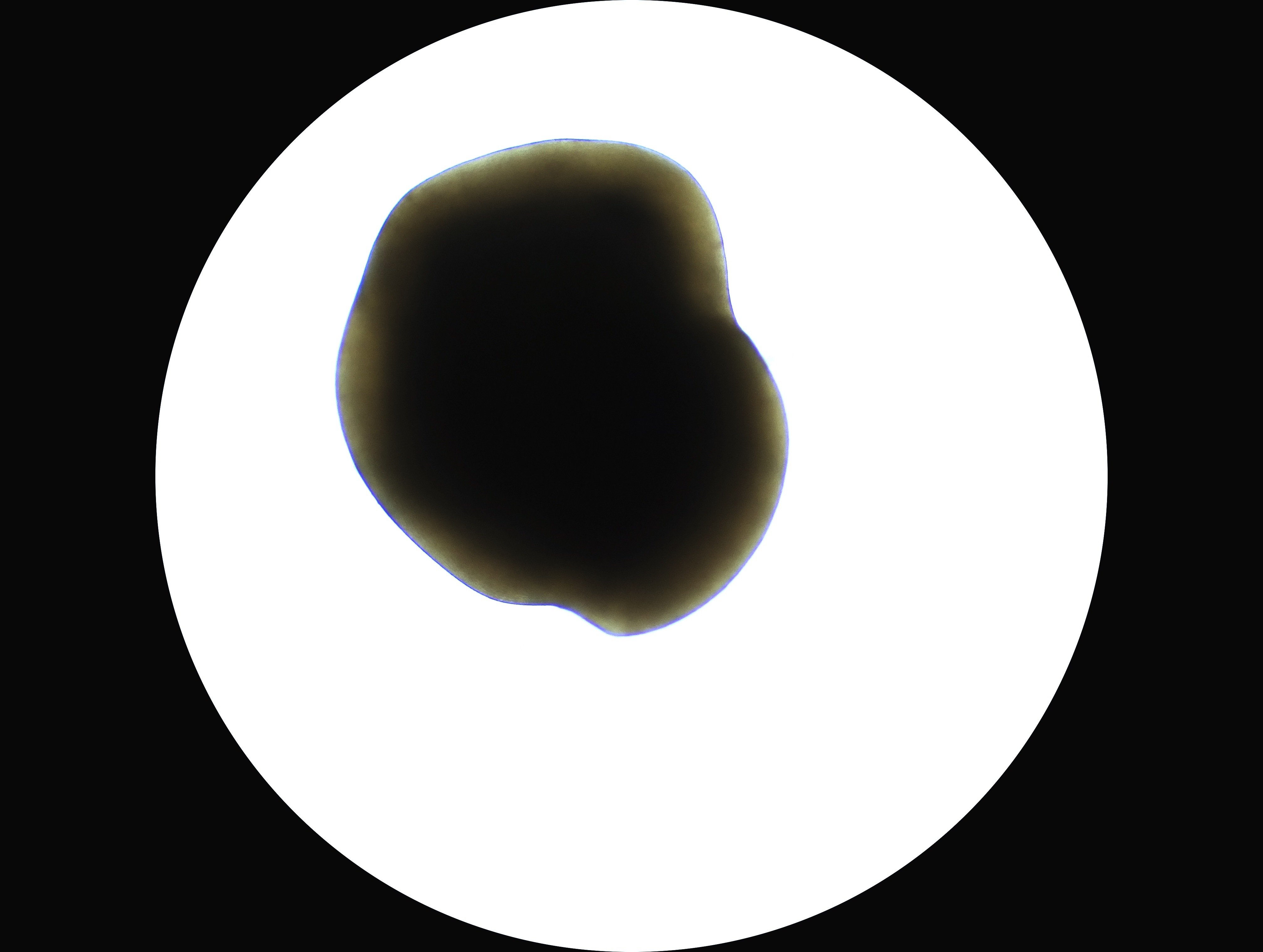

Supplement: Supplementary file 11 — Source data Fig. 3 [file 44319_2025_619_MOESM11_ESM.zip › Figure 3/C,D,F,G/Raw images_mask/OS_day90/MN 11C1 B C3 D90 2x/Day90_0009.jpg]

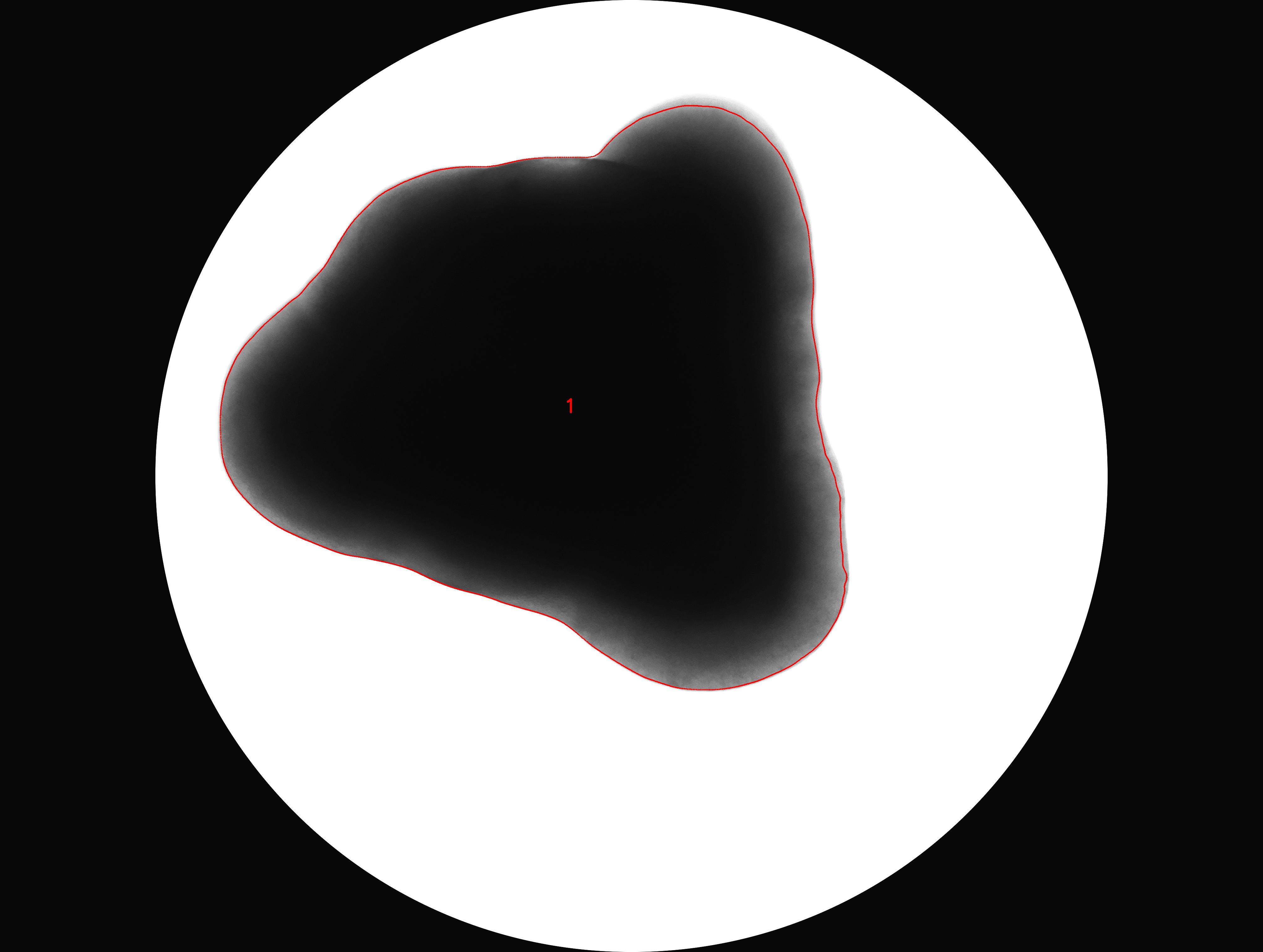

Supplement: Supplementary file 11 — Source data Fig. 3 [file 44319_2025_619_MOESM11_ESM.zip › Figure 3/C,D,F,G/Raw images_mask/OS_day90/MN 11C1 B C3 D90 2x/R_Day90_0004.jpg]

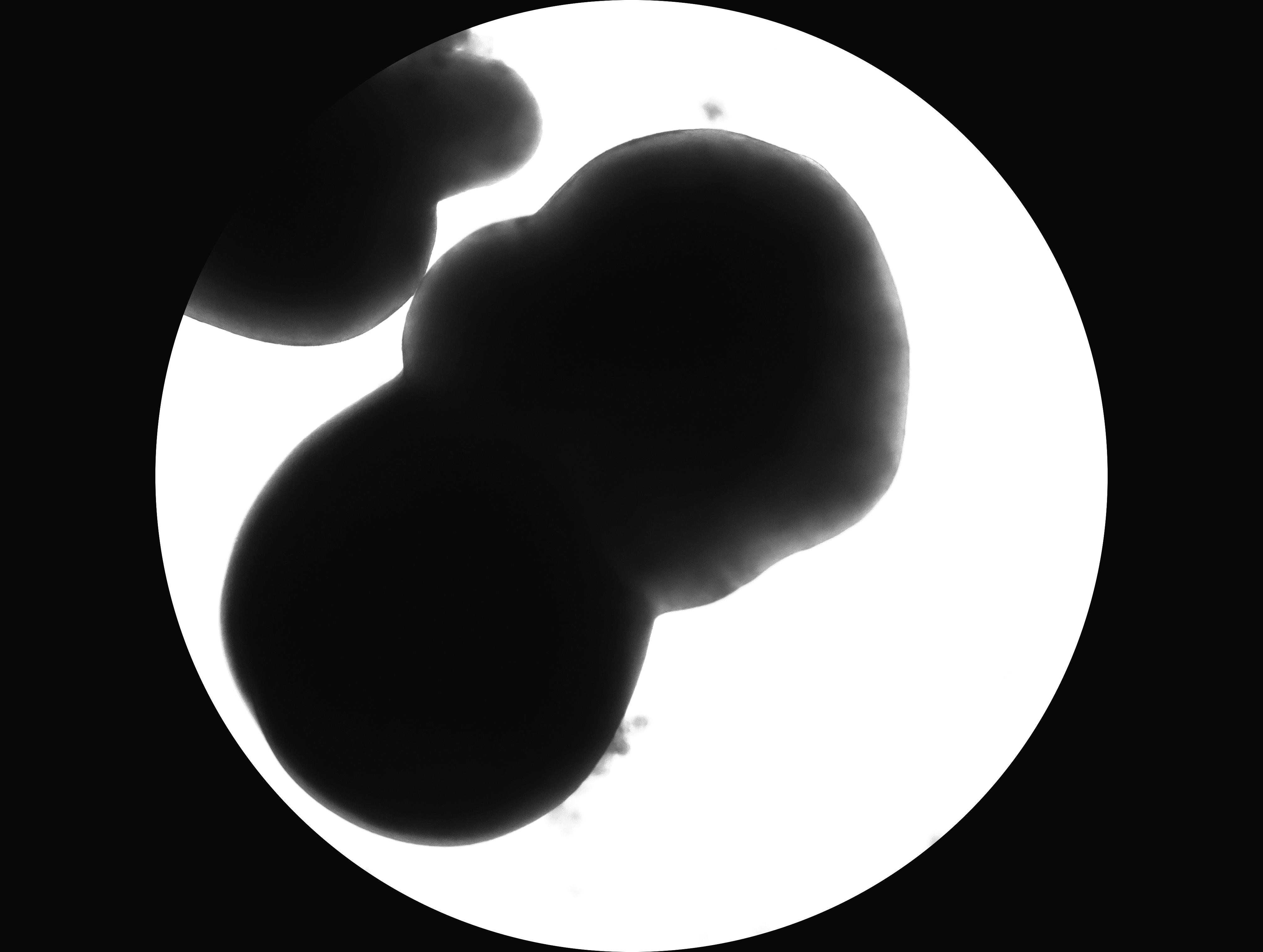

Supplement: Supplementary file 11 — Source data Fig. 3 [file 44319_2025_619_MOESM11_ESM.zip › Figure 3/C,D,F,G/Raw images_mask/OS_day90/MN 11C1 B C3 D90 2x/R_Day90_0005.jpg]

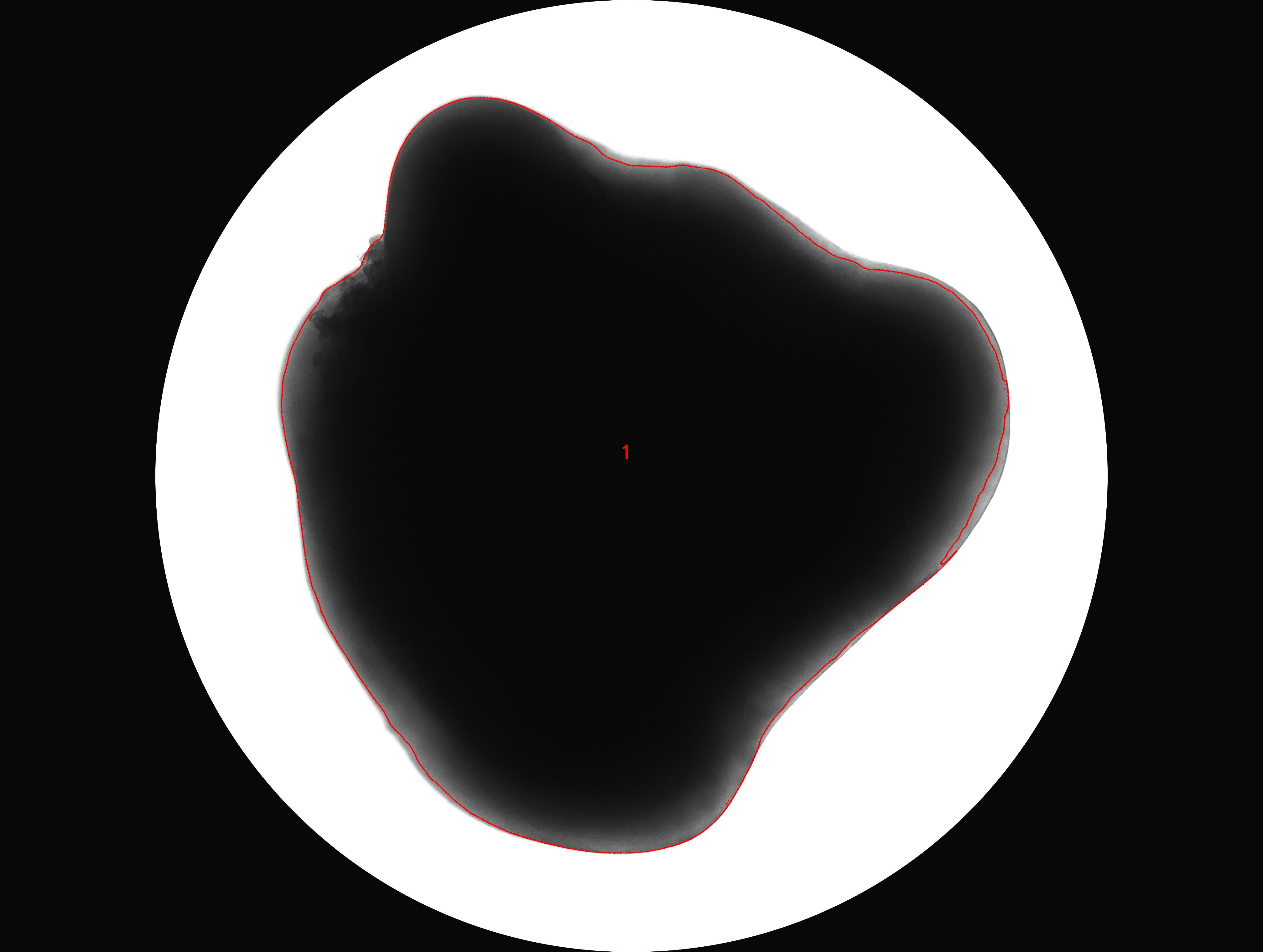

Supplement: Supplementary file 11 — Source data Fig. 3 [file 44319_2025_619_MOESM11_ESM.zip › Figure 3/C,D,F,G/Raw images_mask/OS_day90/MN 11C1 B C3 D90 2x/R_Day90_0007.jpg]

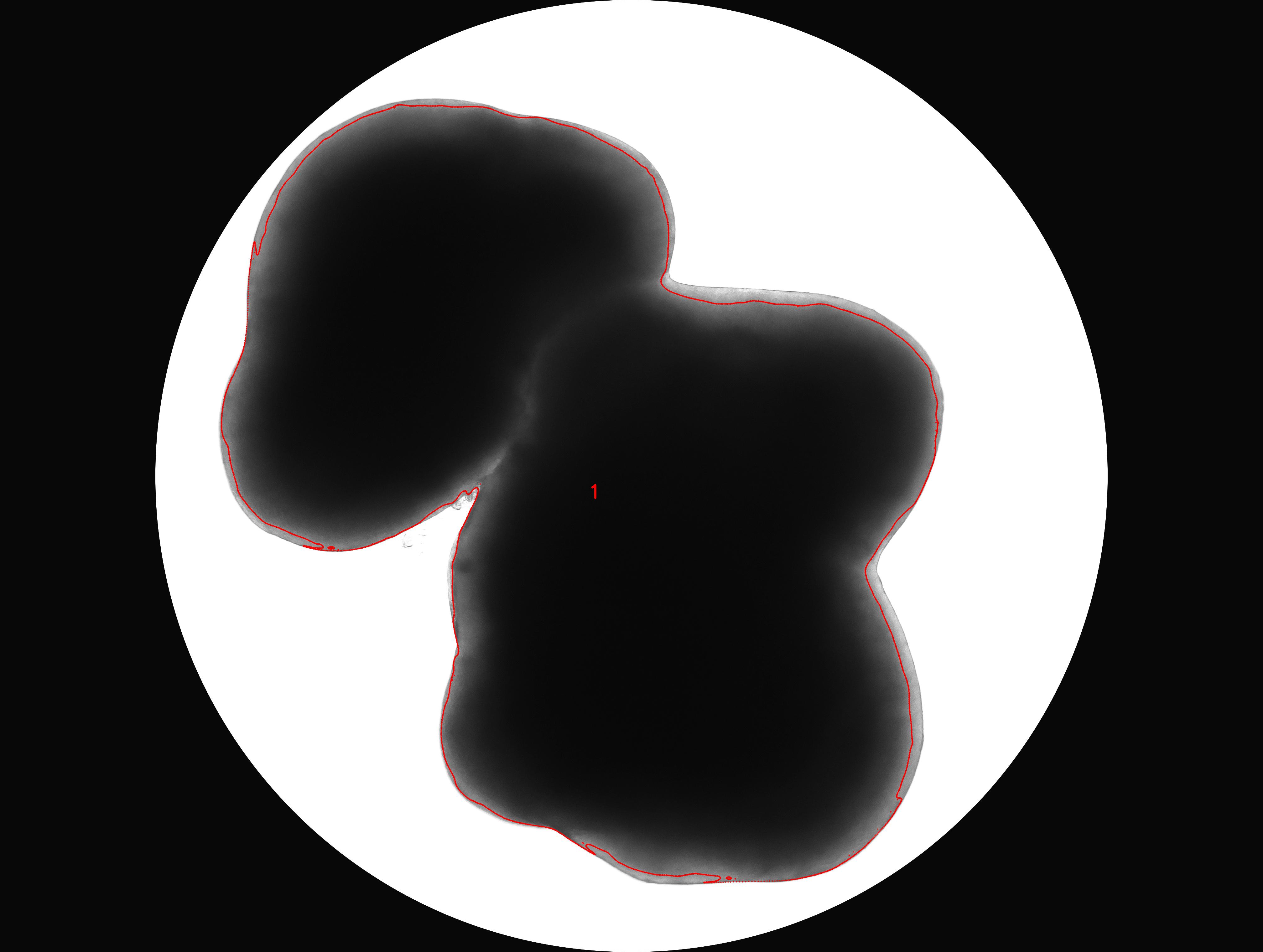

Supplement: Supplementary file 11 — Source data Fig. 3 [file 44319_2025_619_MOESM11_ESM.zip › Figure 3/C,D,F,G/Raw images_mask/OS_day90/MN 11C1 B C3 D90 2x/R_Day90_0006.jpg]

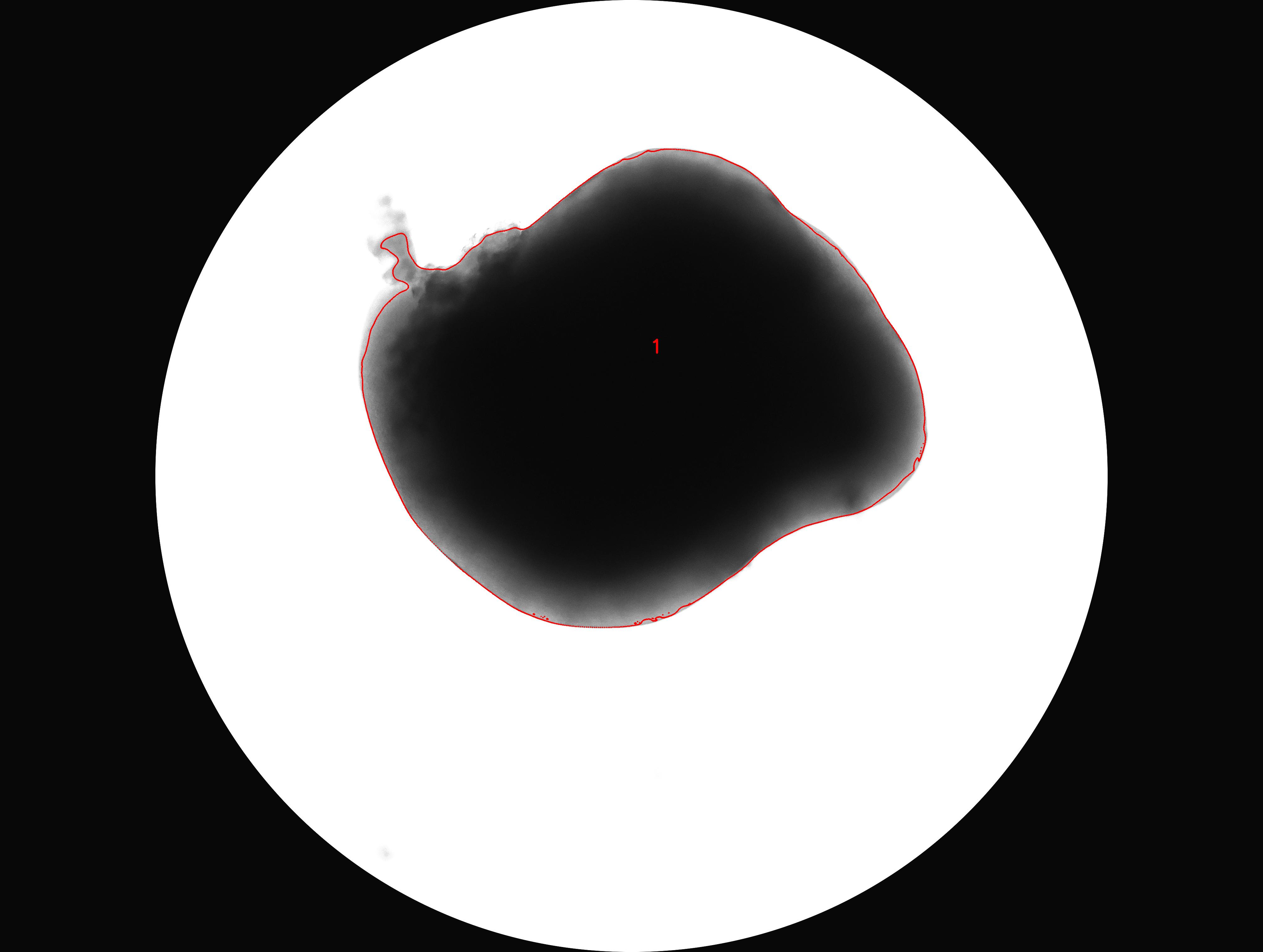

Supplement: Supplementary file 11 — Source data Fig. 3 [file 44319_2025_619_MOESM11_ESM.zip › Figure 3/C,D,F,G/Raw images_mask/OS_day90/MN 11C1 B C3 D90 2x/R_Day90_0002.jpg]

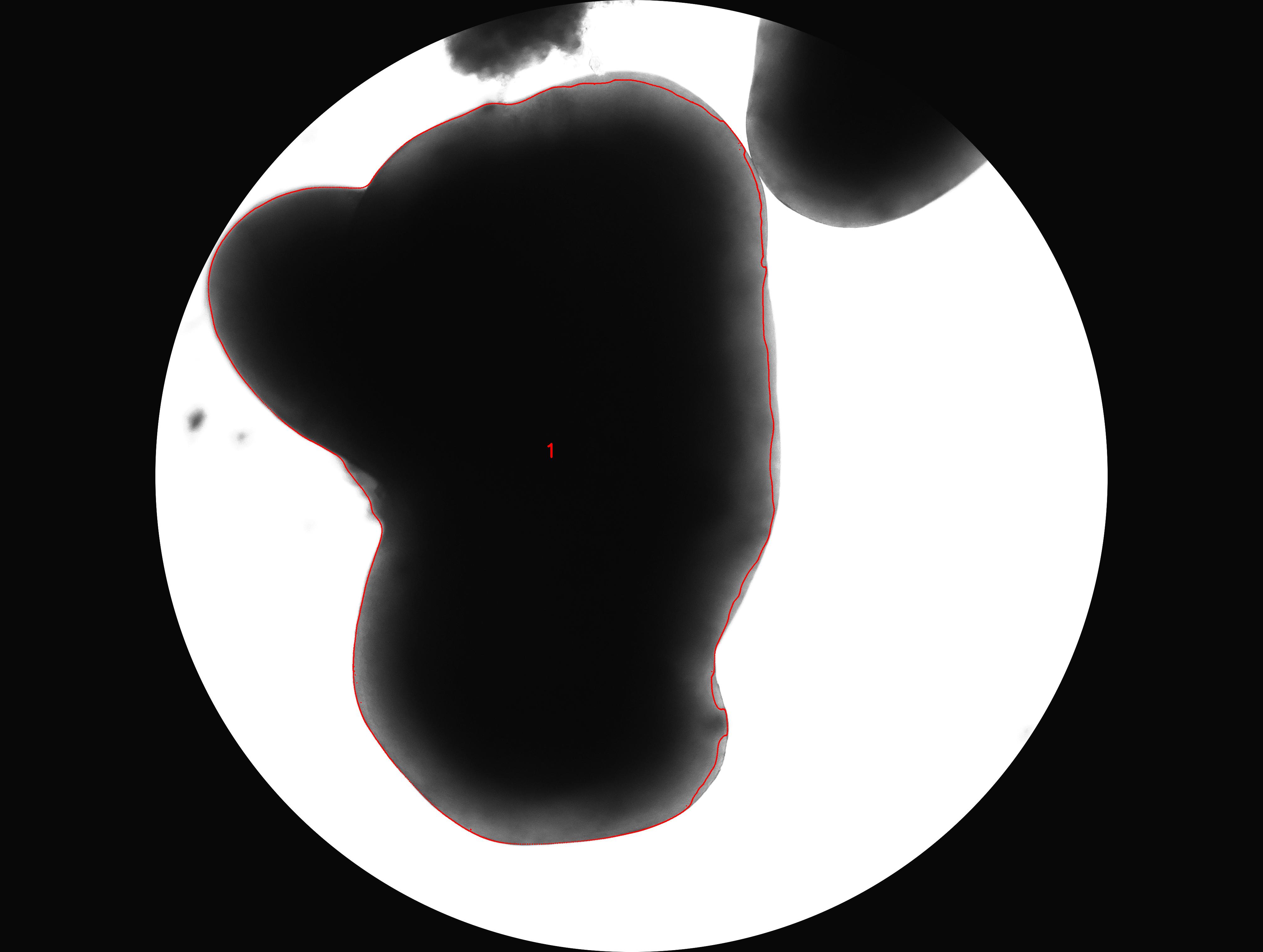

Supplement: Supplementary file 11 — Source data Fig. 3 [file 44319_2025_619_MOESM11_ESM.zip › Figure 3/C,D,F,G/Raw images_mask/OS_day90/MN 11C1 B C3 D90 2x/R_Day90_0003.jpg]

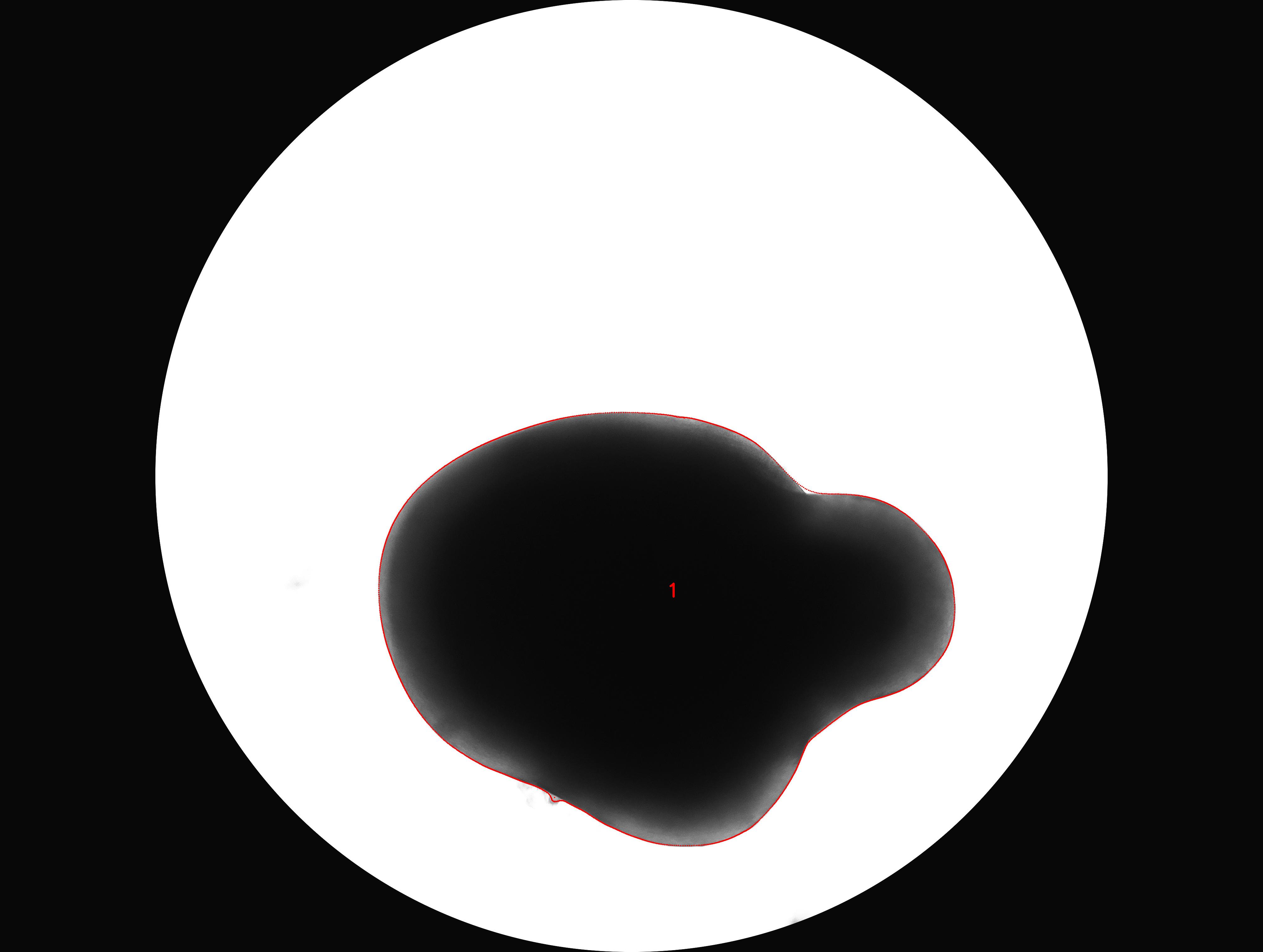

Supplement: Supplementary file 11 — Source data Fig. 3 [file 44319_2025_619_MOESM11_ESM.zip › Figure 3/C,D,F,G/Raw images_mask/OS_day90/MN 11C1 B C3 D90 2x/R_Day90_0001.jpg]

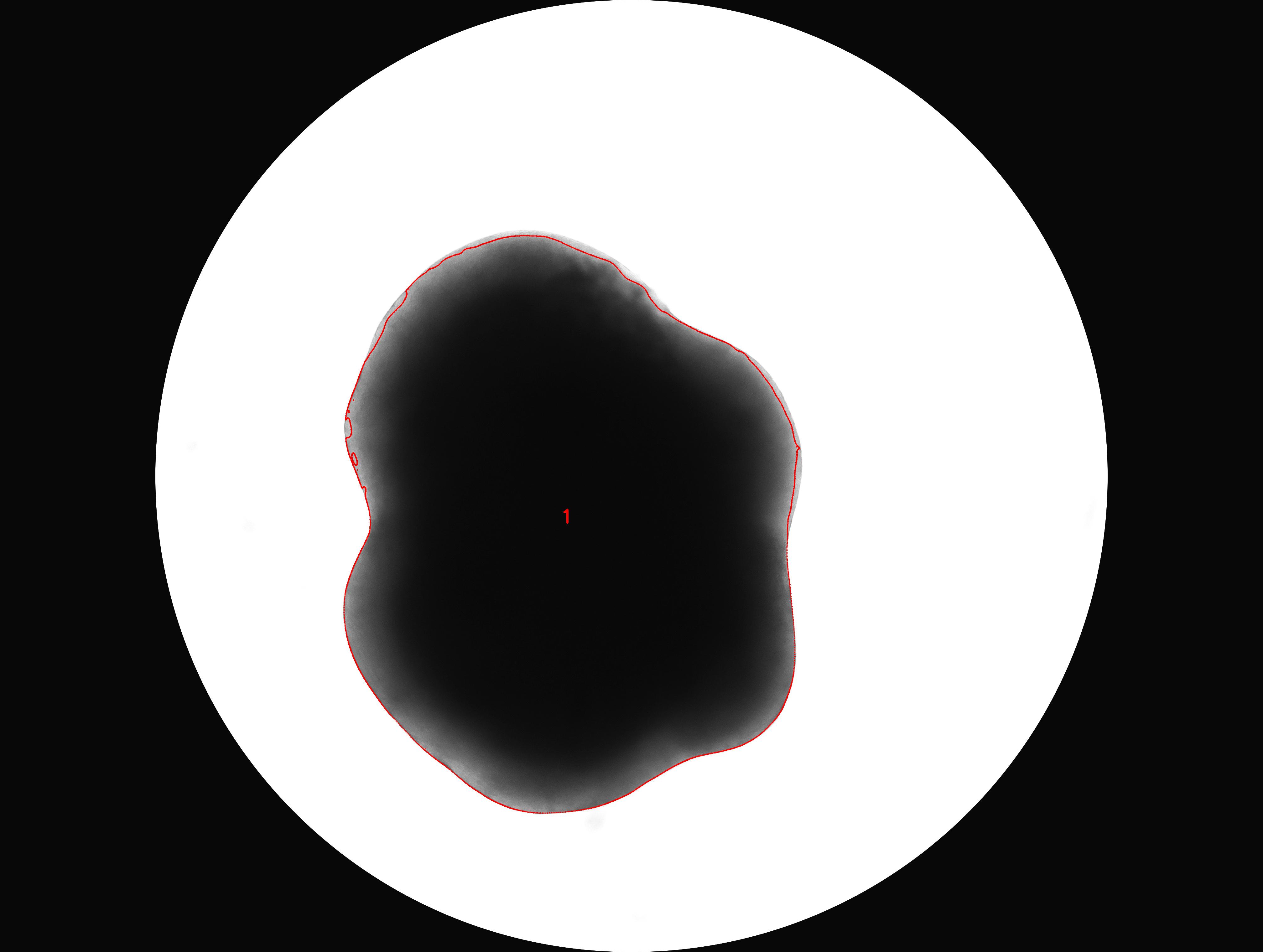

Supplement: Supplementary file 11 — Source data Fig. 3 [file 44319_2025_619_MOESM11_ESM.zip › Figure 3/C,D,F,G/Raw images_mask/OS_day90/MN 11C1 B C3 D90 2x/R_Day90_0000.jpg]

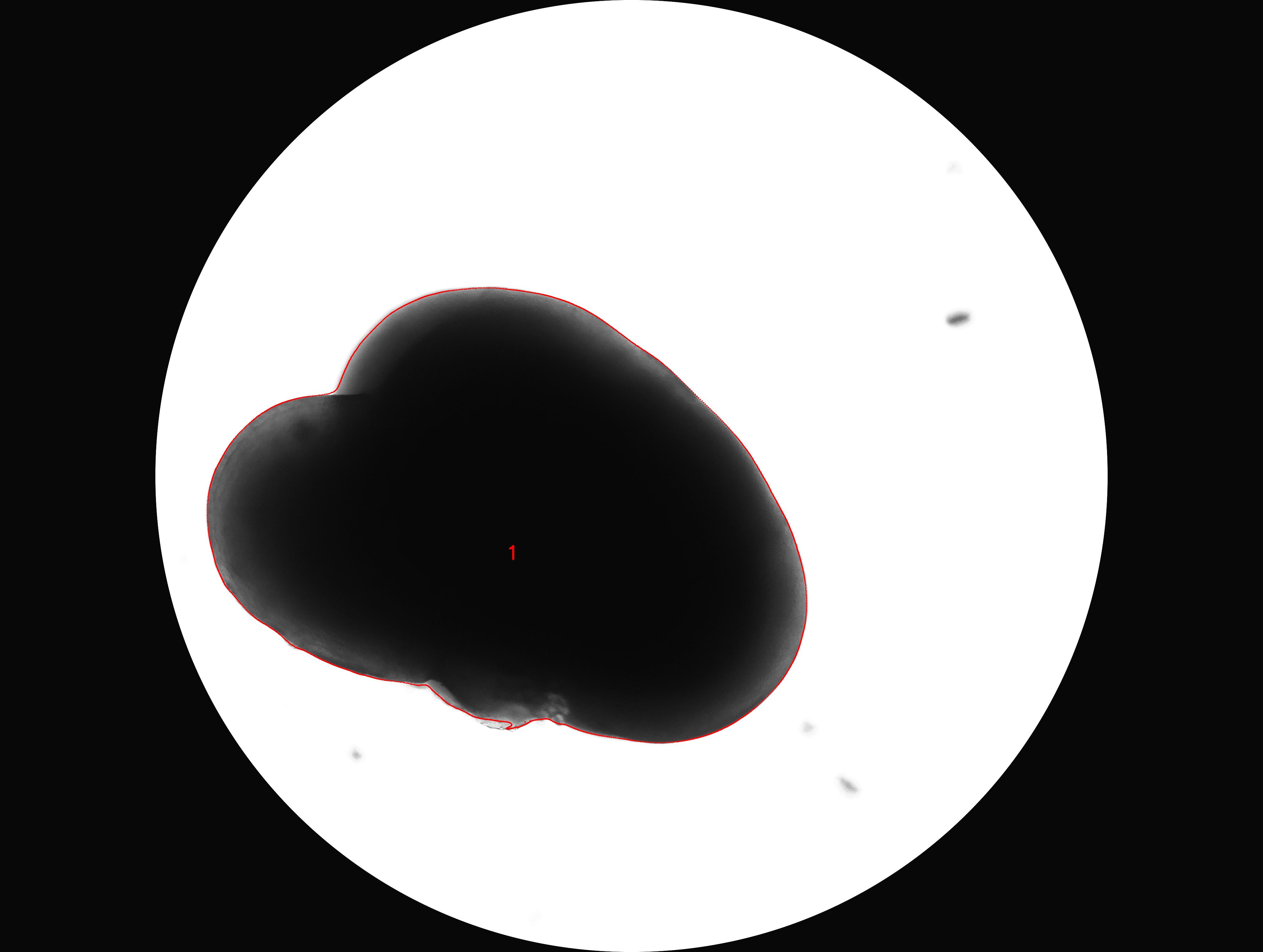

Supplement: Supplementary file 11 — Source data Fig. 3 [file 44319_2025_619_MOESM11_ESM.zip › Figure 3/C,D,F,G/Raw images_mask/OS_day90/MN 11C1 B C3 D90 2x/R_Day90_0008.jpg]

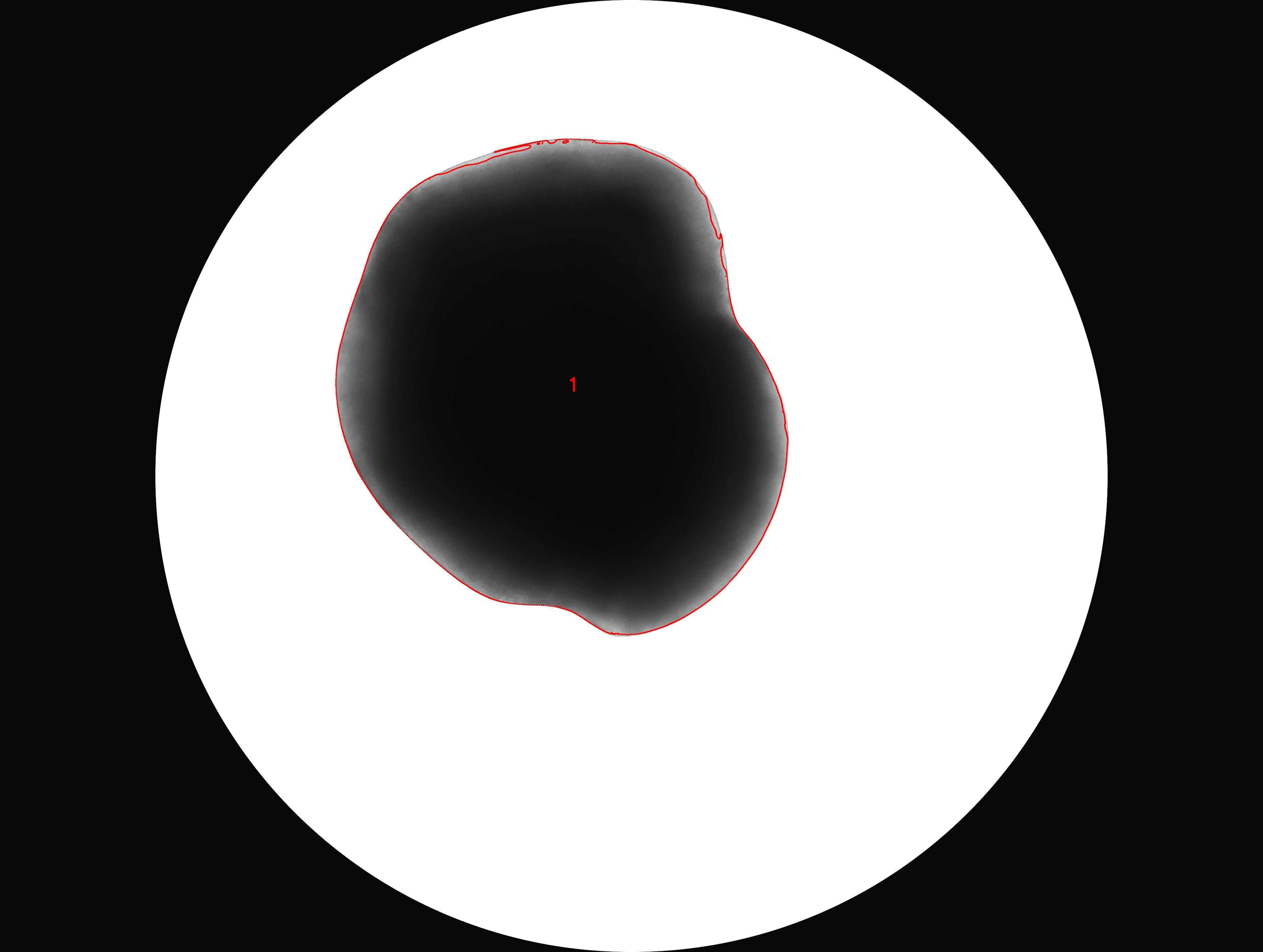

Supplement: Supplementary file 11 — Source data Fig. 3 [file 44319_2025_619_MOESM11_ESM.zip › Figure 3/C,D,F,G/Raw images_mask/OS_day90/MN 11C1 B C3 D90 2x/R_Day90_0009.jpg]

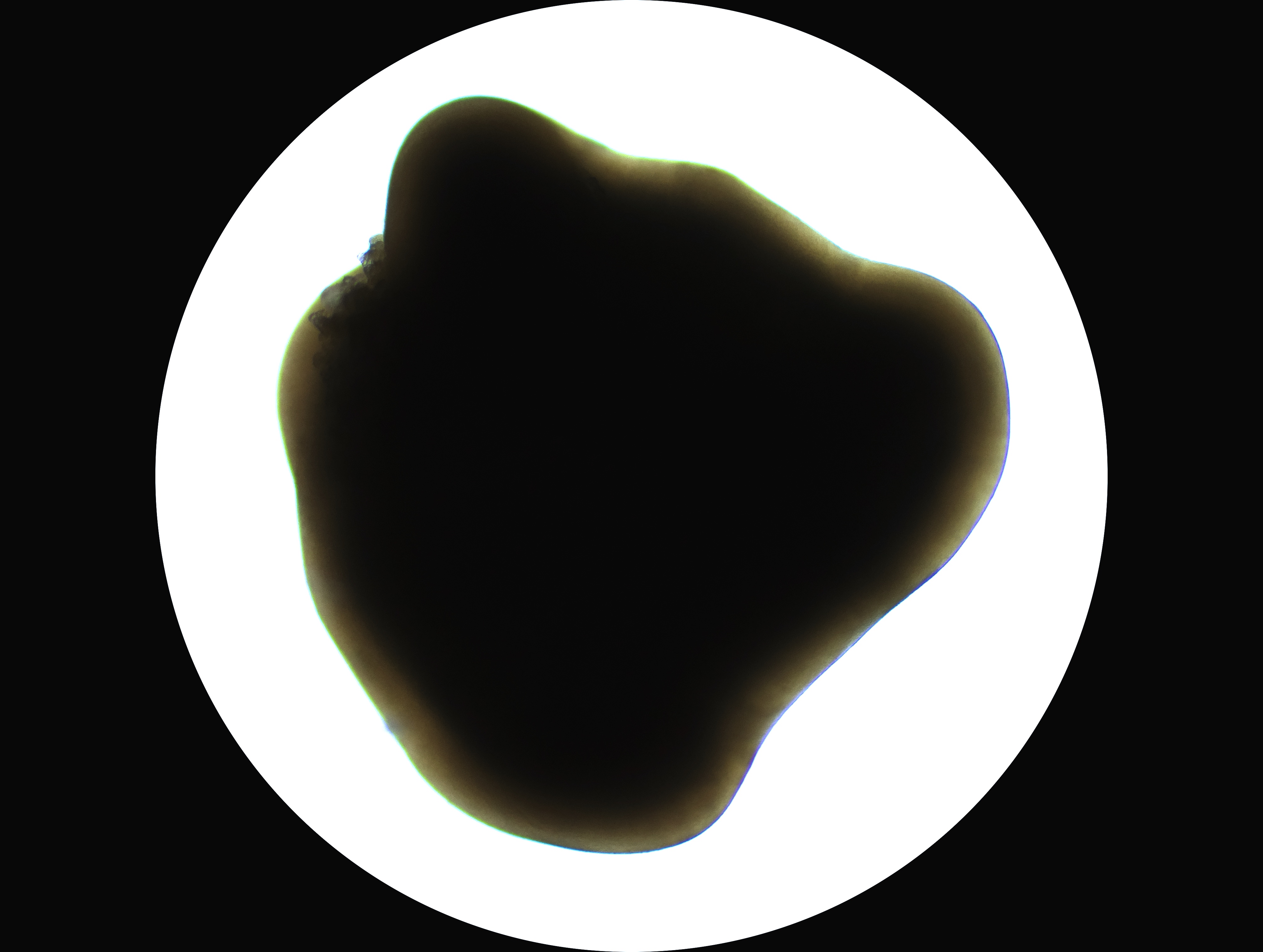

Supplement: Supplementary file 11 — Source data Fig. 3 [file 44319_2025_619_MOESM11_ESM.zip › Figure 3/C,D,F,G/Raw images_mask/OS_day90/MN 11C1 B C3 D90 2x/Day90_0007.jpg]

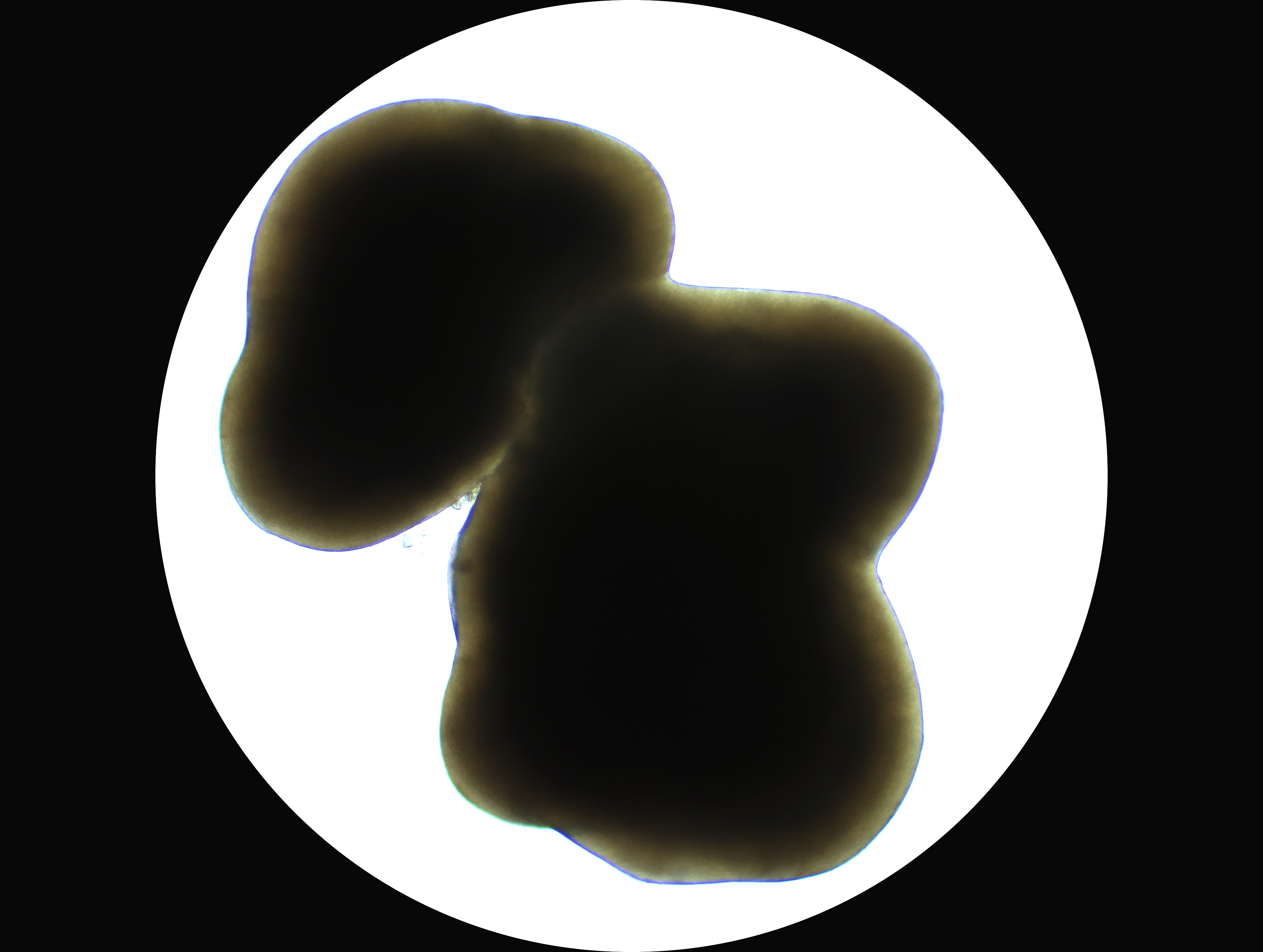

Supplement: Supplementary file 11 — Source data Fig. 3 [file 44319_2025_619_MOESM11_ESM.zip › Figure 3/C,D,F,G/Raw images_mask/OS_day90/MN 11C1 B C3 D90 2x/Day90_0006.jpg]

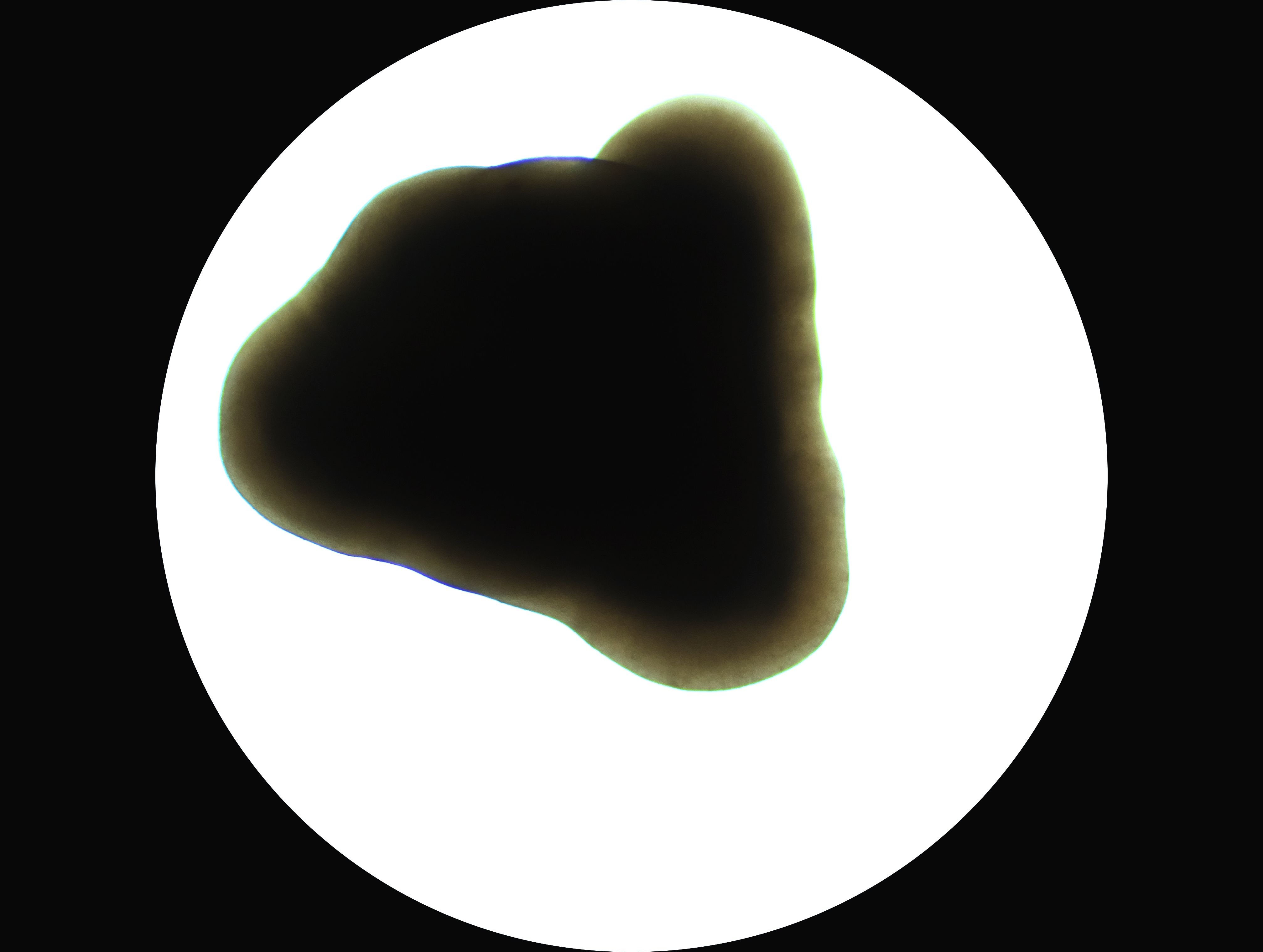

Supplement: Supplementary file 11 — Source data Fig. 3 [file 44319_2025_619_MOESM11_ESM.zip › Figure 3/C,D,F,G/Raw images_mask/OS_day90/MN 11C1 B C3 D90 2x/Day90_0004.jpg]

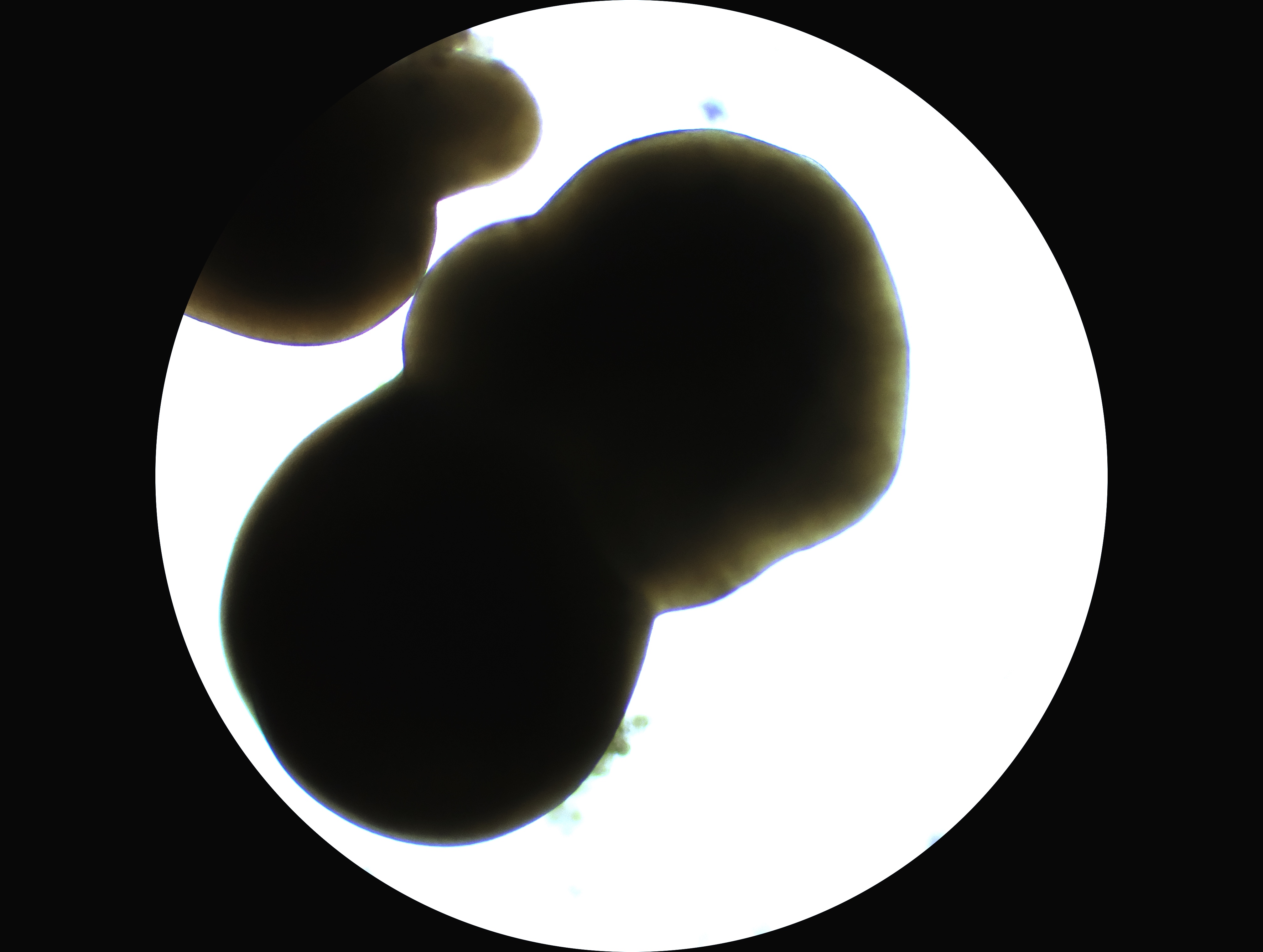

Supplement: Supplementary file 11 — Source data Fig. 3 [file 44319_2025_619_MOESM11_ESM.zip › Figure 3/C,D,F,G/Raw images_mask/OS_day90/MN 11C1 B C3 D90 2x/Day90_0005.jpg]

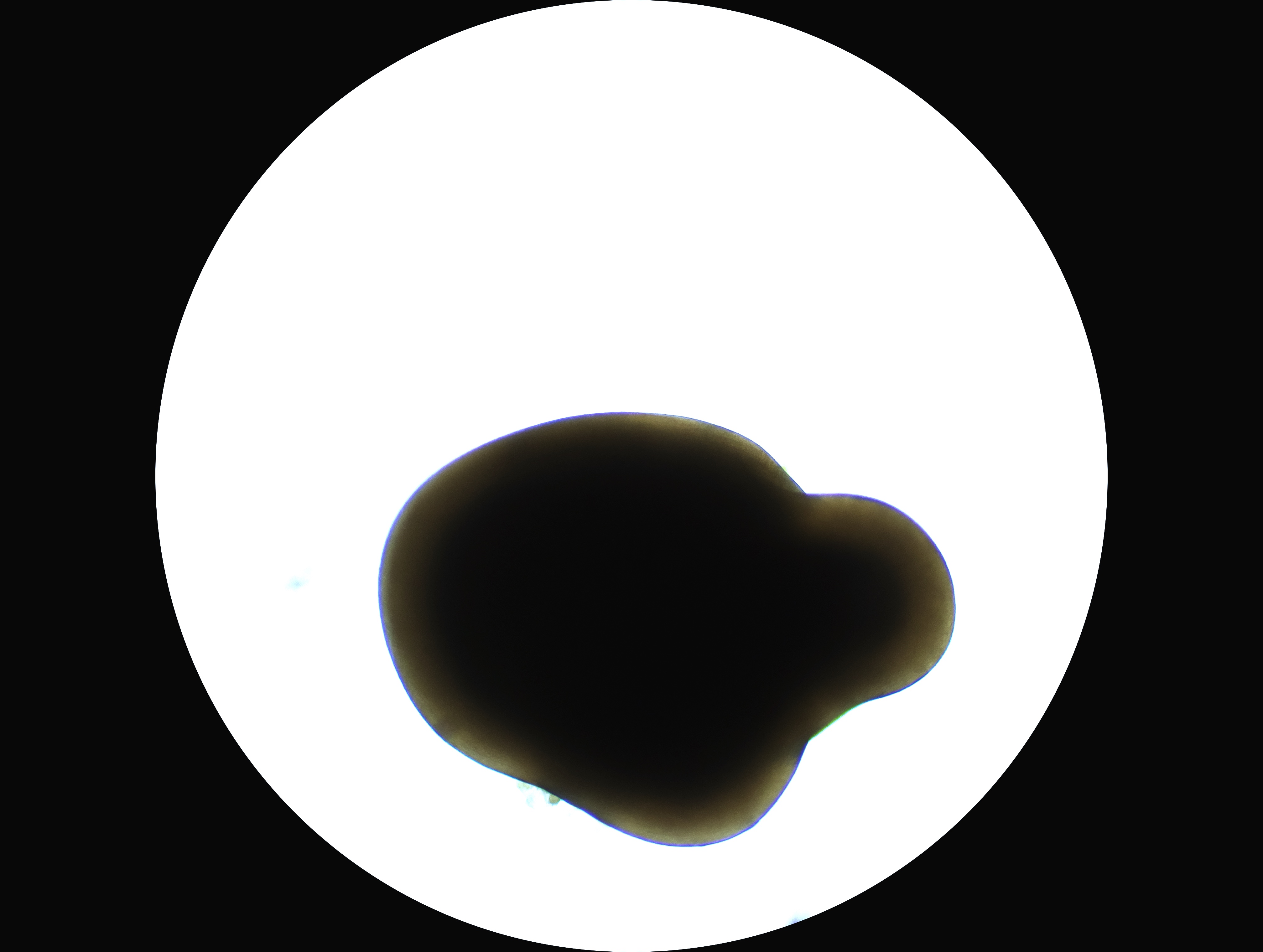

Supplement: Supplementary file 11 — Source data Fig. 3 [file 44319_2025_619_MOESM11_ESM.zip › Figure 3/C,D,F,G/Raw images_mask/OS_day90/MN 11C1 B C3 D90 2x/Day90_0001.jpg]

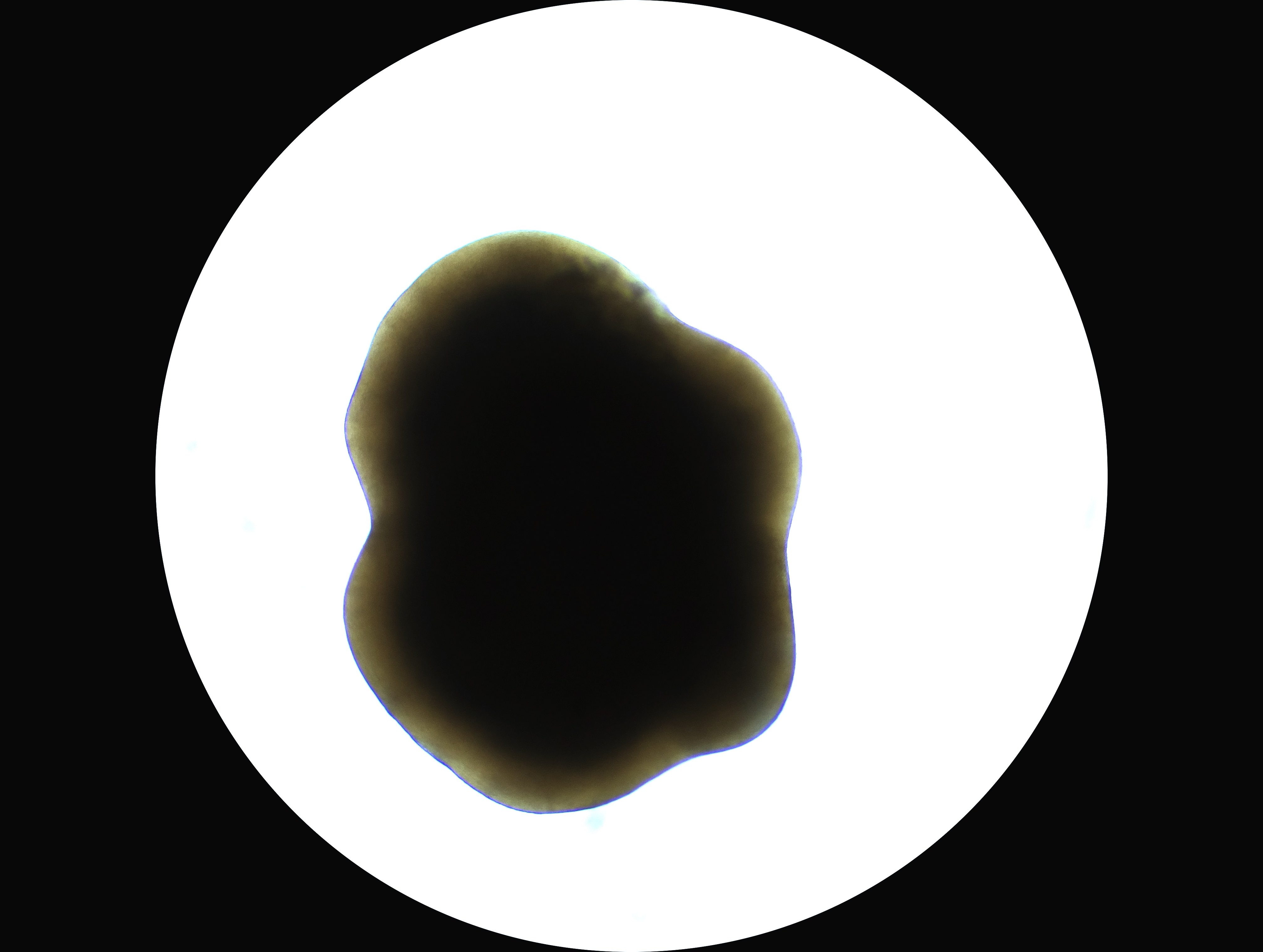

Supplement: Supplementary file 11 — Source data Fig. 3 [file 44319_2025_619_MOESM11_ESM.zip › Figure 3/C,D,F,G/Raw images_mask/OS_day90/MN 11C1 B C3 D90 2x/Day90_0000.jpg]

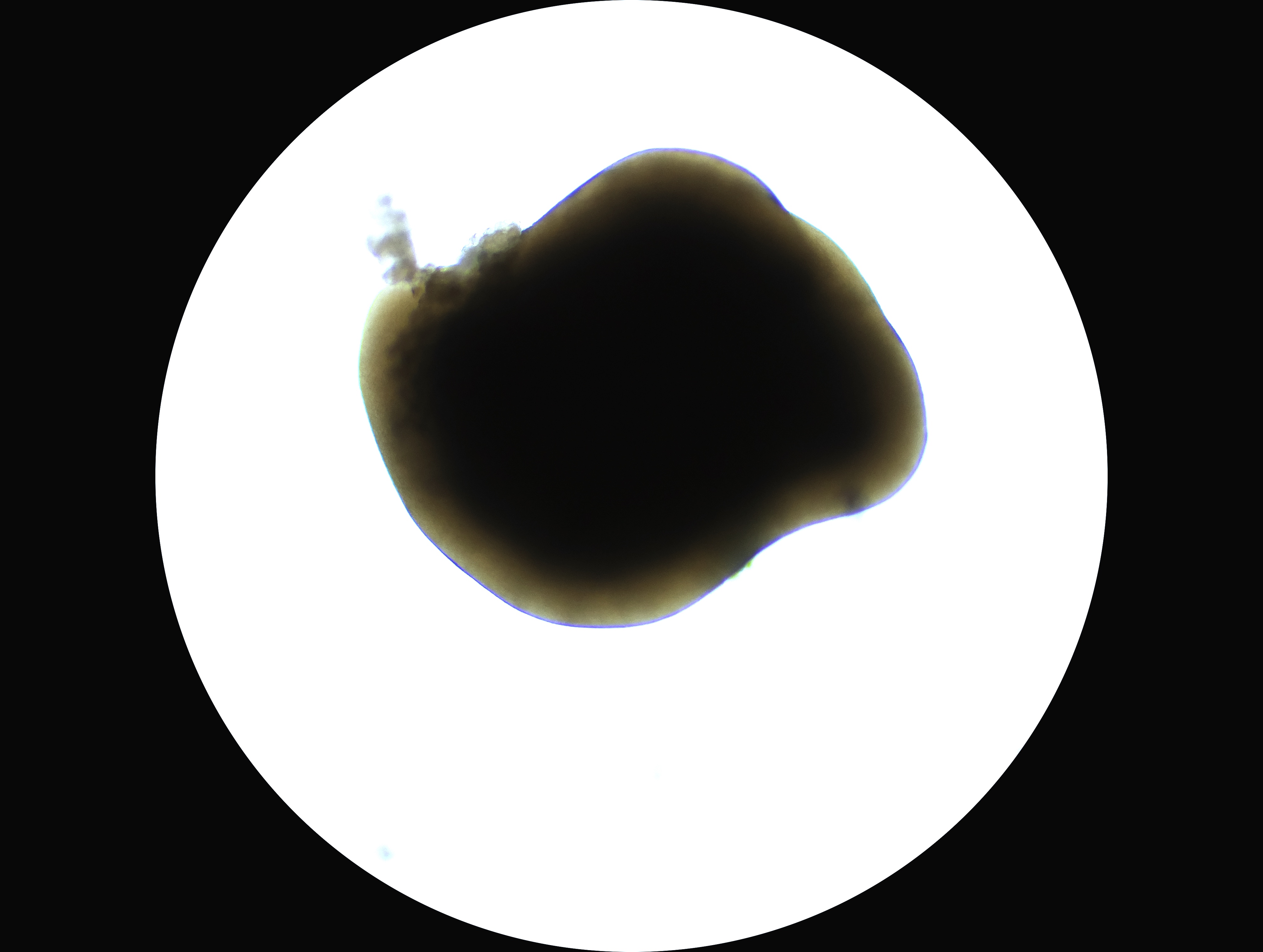

Supplement: Supplementary file 11 — Source data Fig. 3 [file 44319_2025_619_MOESM11_ESM.zip › Figure 3/C,D,F,G/Raw images_mask/OS_day90/MN 11C1 B C3 D90 2x/Day90_0002.jpg]

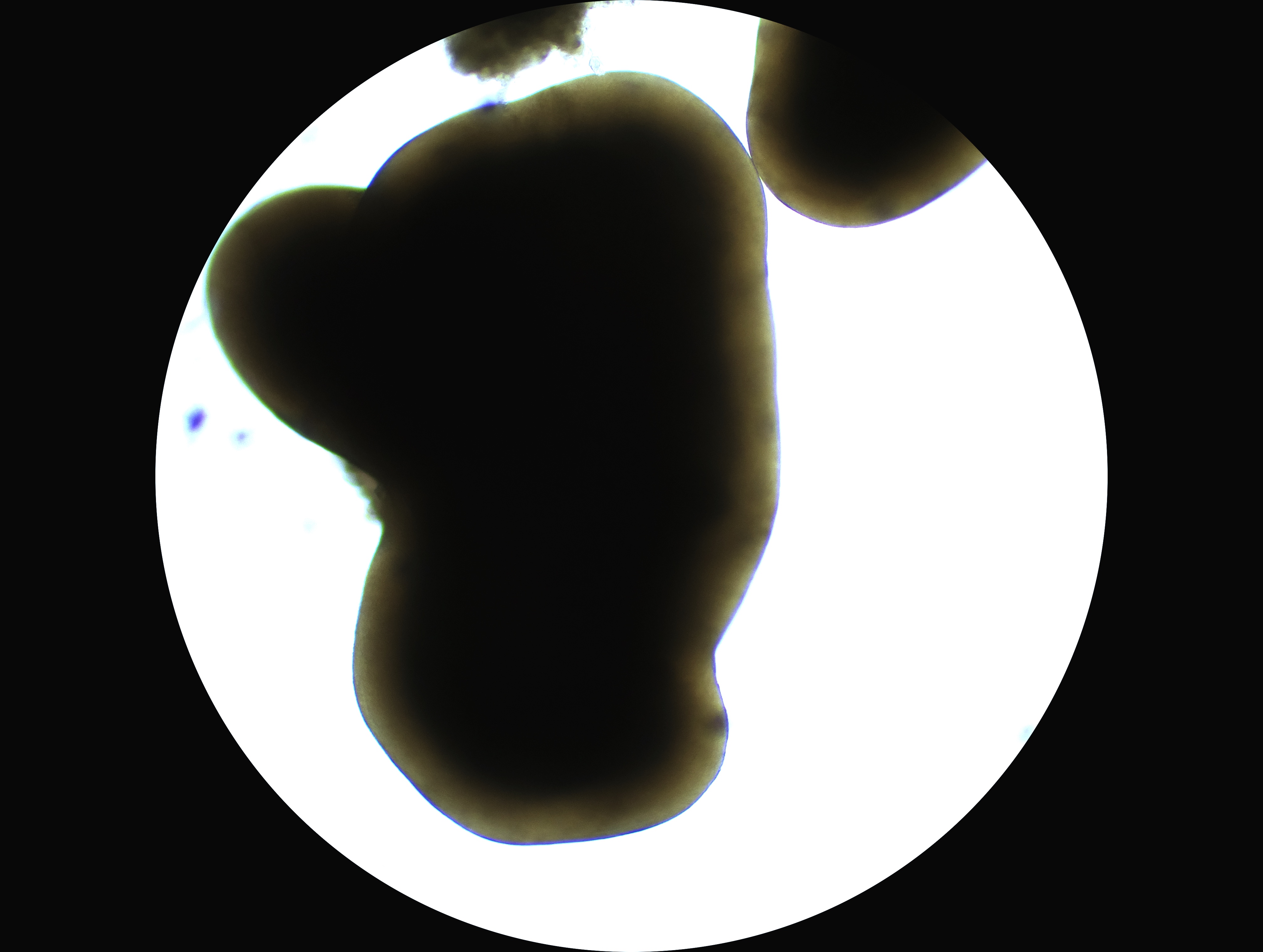

Supplement: Supplementary file 11 — Source data Fig. 3 [file 44319_2025_619_MOESM11_ESM.zip › Figure 3/C,D,F,G/Raw images_mask/OS_day90/MN 11C1 B C3 D90 2x/Day90_0003.jpg]

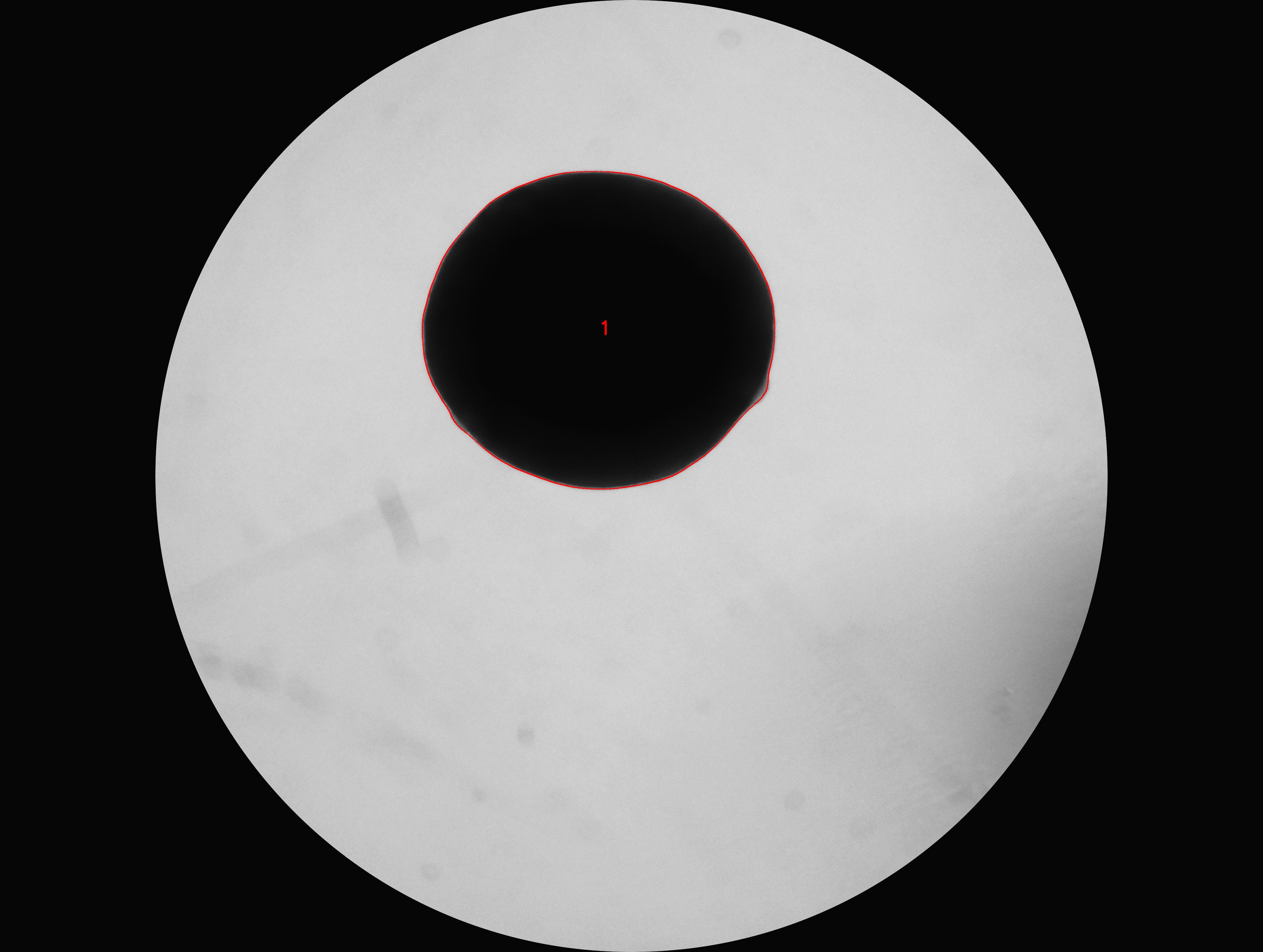

Supplement: Supplementary file 11 — Source data Fig. 3 [file 44319_2025_619_MOESM11_ESM.zip › Figure 3/C,D,F,G/Raw images_mask/OS_day90/MN 11C1 B C7 D90 2x/R_Day 90_0026.jpg]

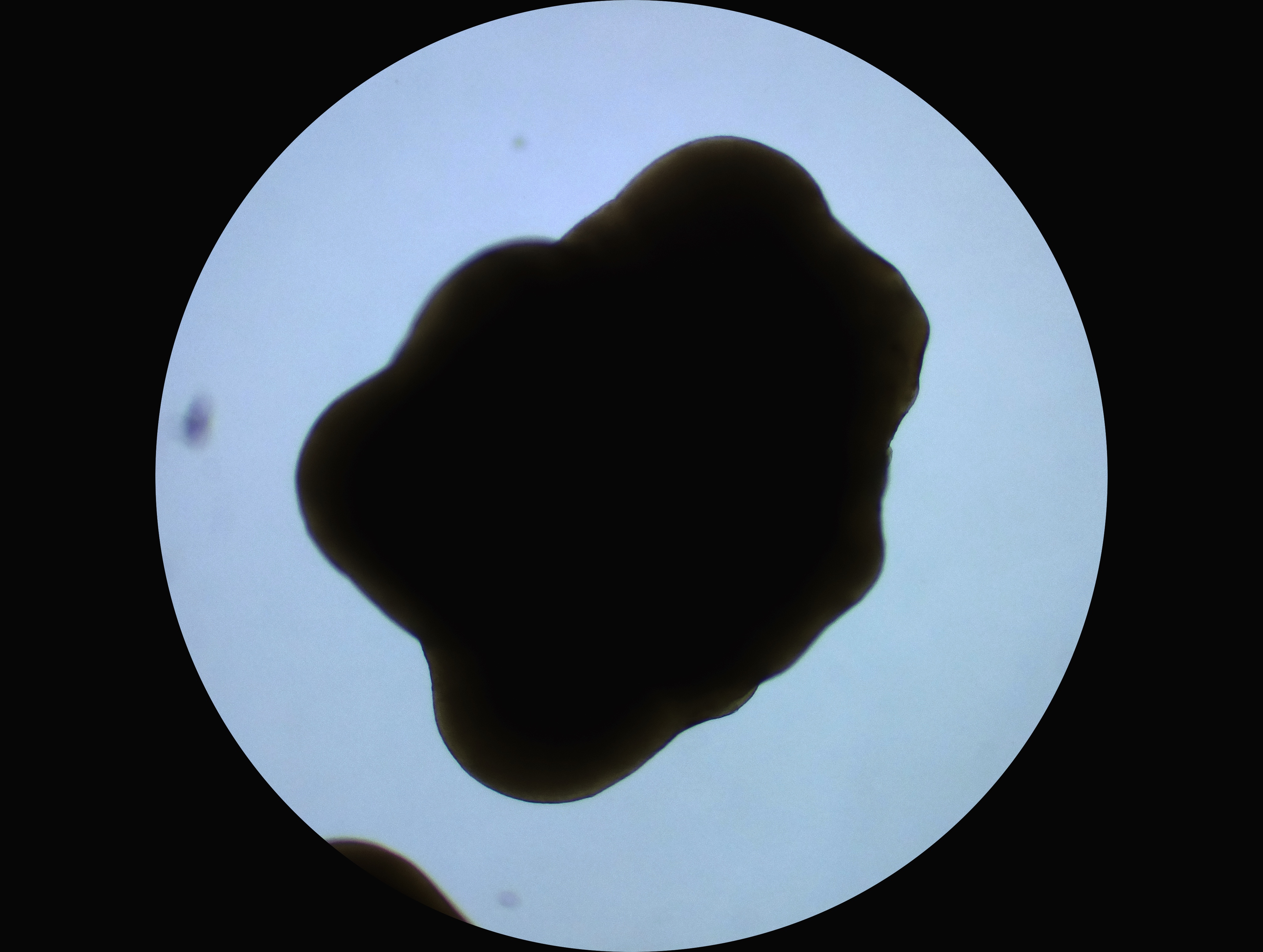

Supplement: Supplementary file 11 — Source data Fig. 3 [file 44319_2025_619_MOESM11_ESM.zip › Figure 3/C,D,F,G/Raw images_mask/OS_day90/MN 11C1 B C7 D90 2x/Day 90_0029.jpg]

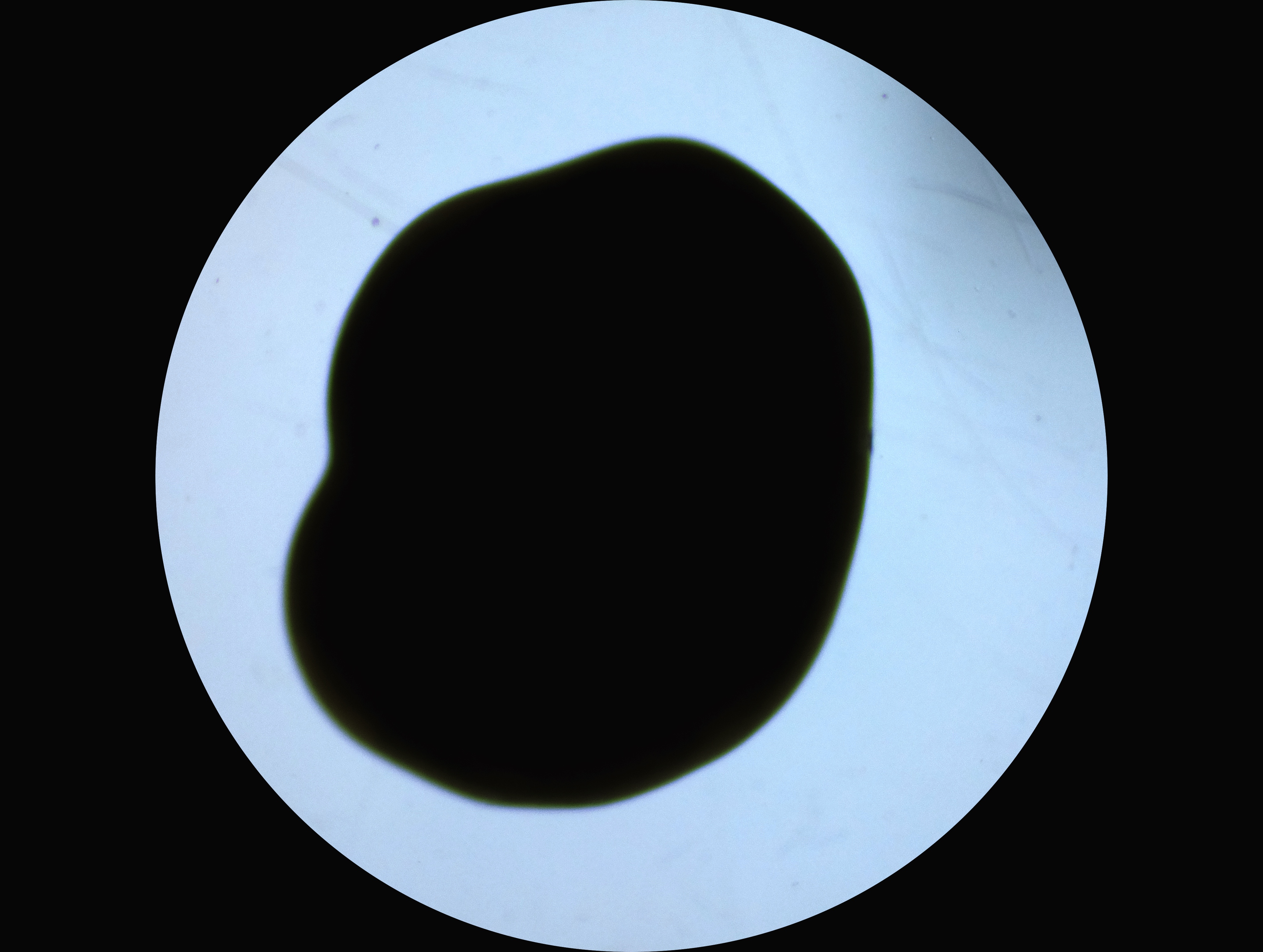

Supplement: Supplementary file 11 — Source data Fig. 3 [file 44319_2025_619_MOESM11_ESM.zip › Figure 3/C,D,F,G/Raw images_mask/OS_day90/MN 11C1 B C7 D90 2x/Day 90_0014.jpg]

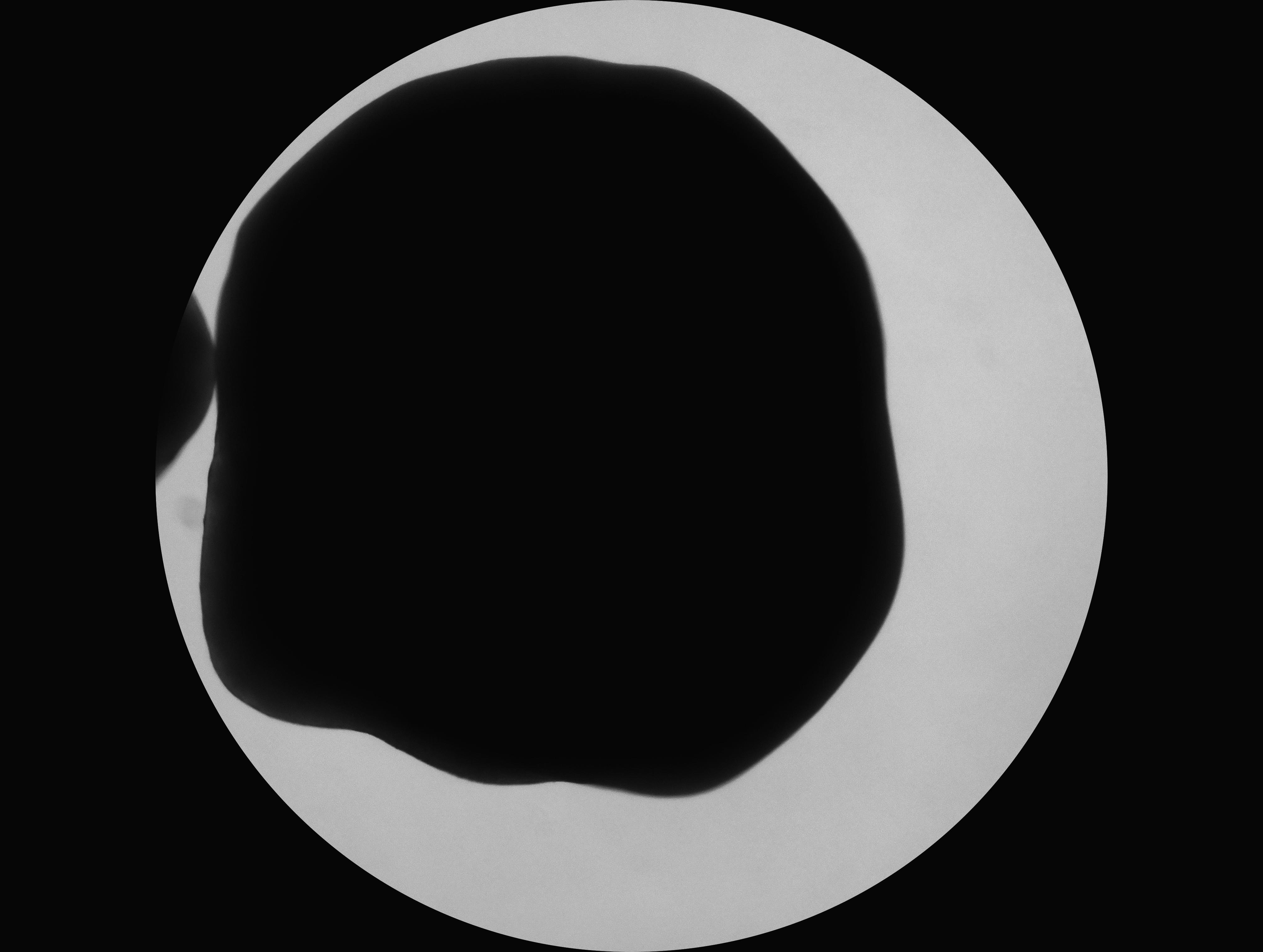

Supplement: Supplementary file 11 — Source data Fig. 3 [file 44319_2025_619_MOESM11_ESM.zip › Figure 3/C,D,F,G/Raw images_mask/OS_day90/MN 11C1 B C7 D90 2x/R_Day 90_0033.jpg]

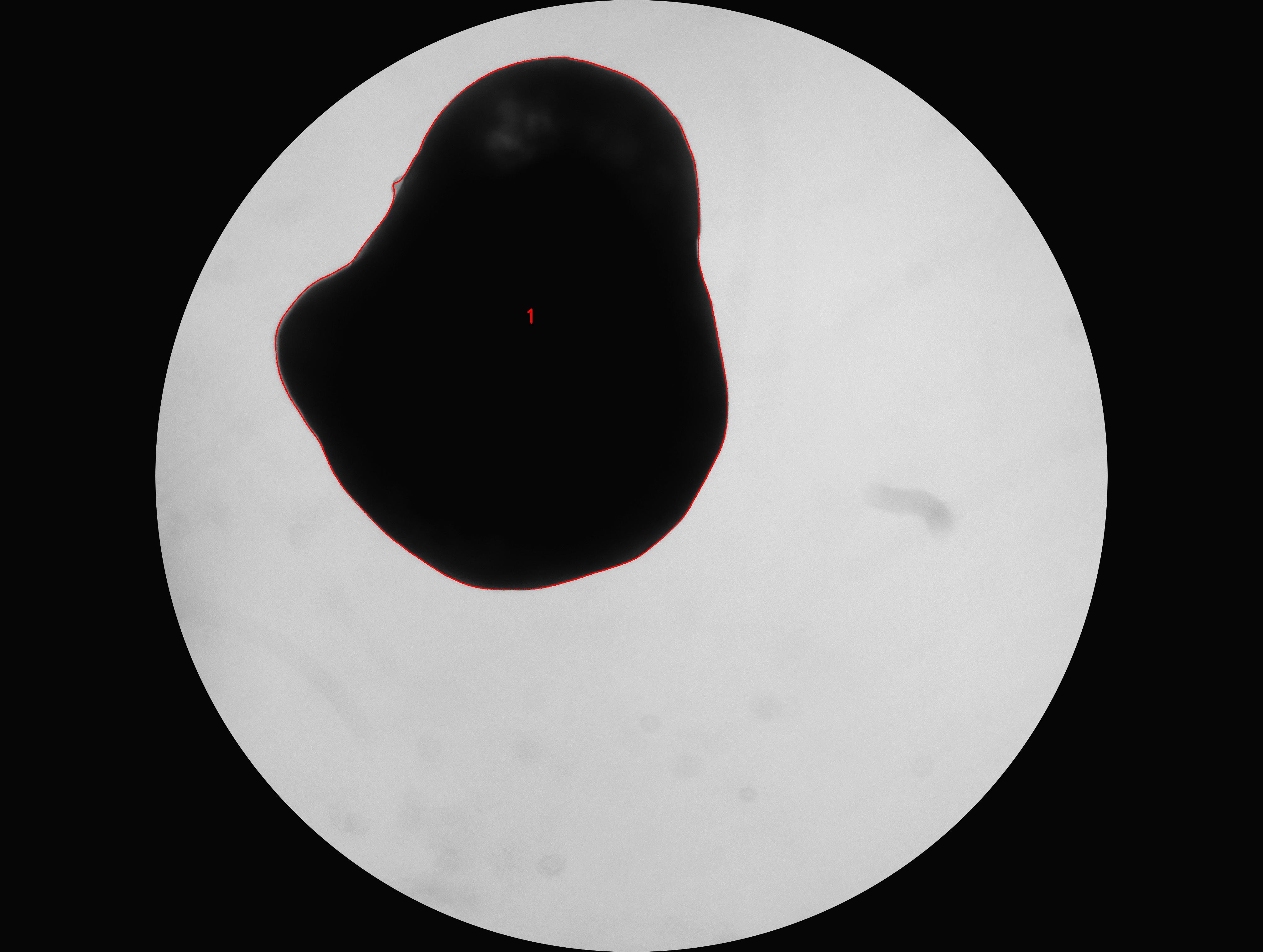

Supplement: Supplementary file 11 — Source data Fig. 3 [file 44319_2025_619_MOESM11_ESM.zip › Figure 3/C,D,F,G/Raw images_mask/OS_day90/MN 11C1 B C7 D90 2x/R_Day 90_0025.jpg]

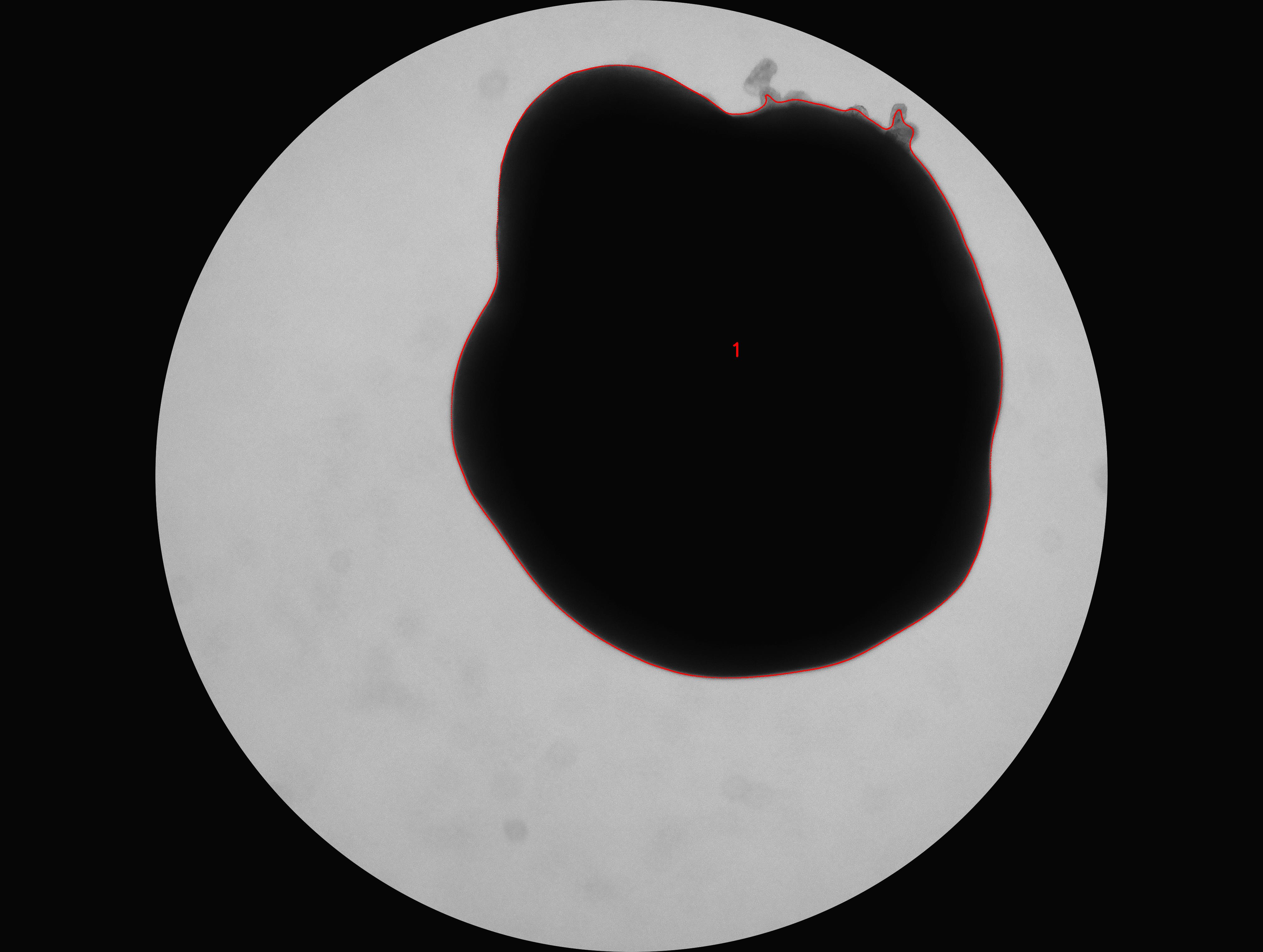

Supplement: Supplementary file 11 — Source data Fig. 3 [file 44319_2025_619_MOESM11_ESM.zip › Figure 3/C,D,F,G/Raw images_mask/OS_day90/MN 11C1 B C7 D90 2x/R_Day 90_0031.jpg]

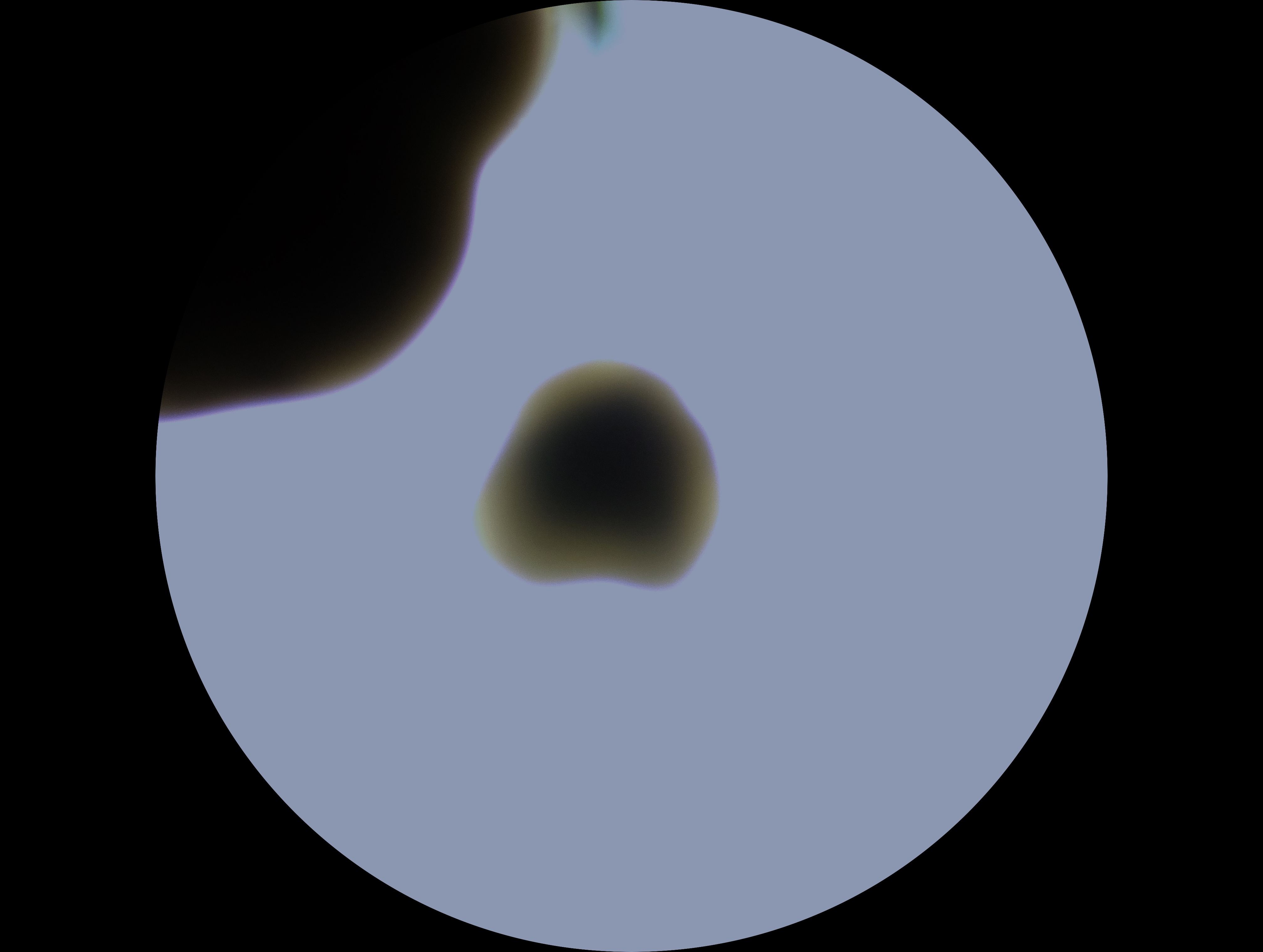

Supplement: Supplementary file 11 — Source data Fig. 3 [file 44319_2025_619_MOESM11_ESM.zip › Figure 3/C,D,F,G/Raw images_mask/OS_day90/MN 11C1 B C7 D90 2x/Day 90_0002.jpg]

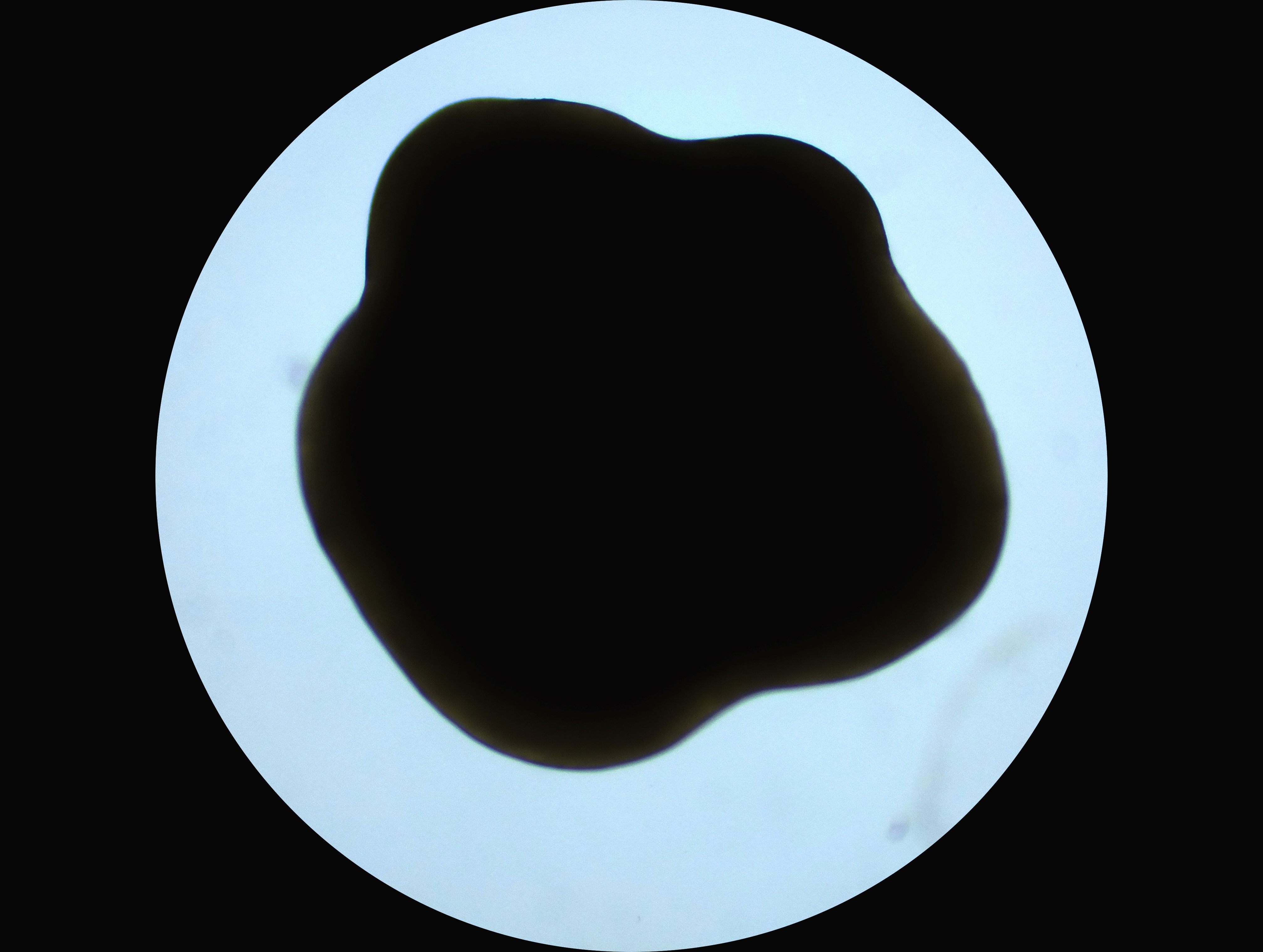

Supplement: Supplementary file 11 — Source data Fig. 3 [file 44319_2025_619_MOESM11_ESM.zip › Figure 3/C,D,F,G/Raw images_mask/OS_day90/MN 11C1 B C7 D90 2x/Day 90_0016.jpg]

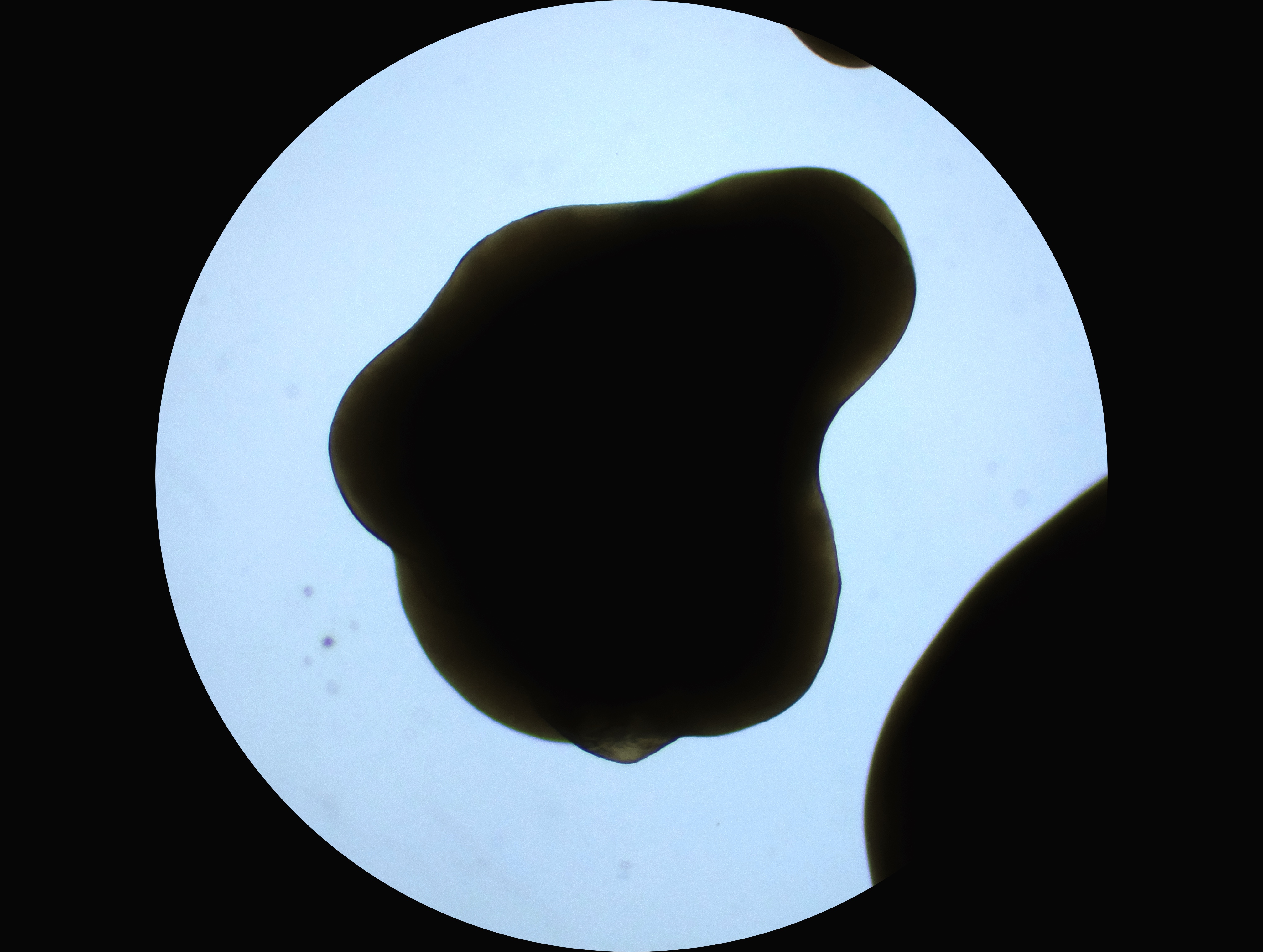

Supplement: Supplementary file 11 — Source data Fig. 3 [file 44319_2025_619_MOESM11_ESM.zip › Figure 3/C,D,F,G/Raw images_mask/OS_day90/MN 11C1 B C7 D90 2x/Day 90_0017.jpg]

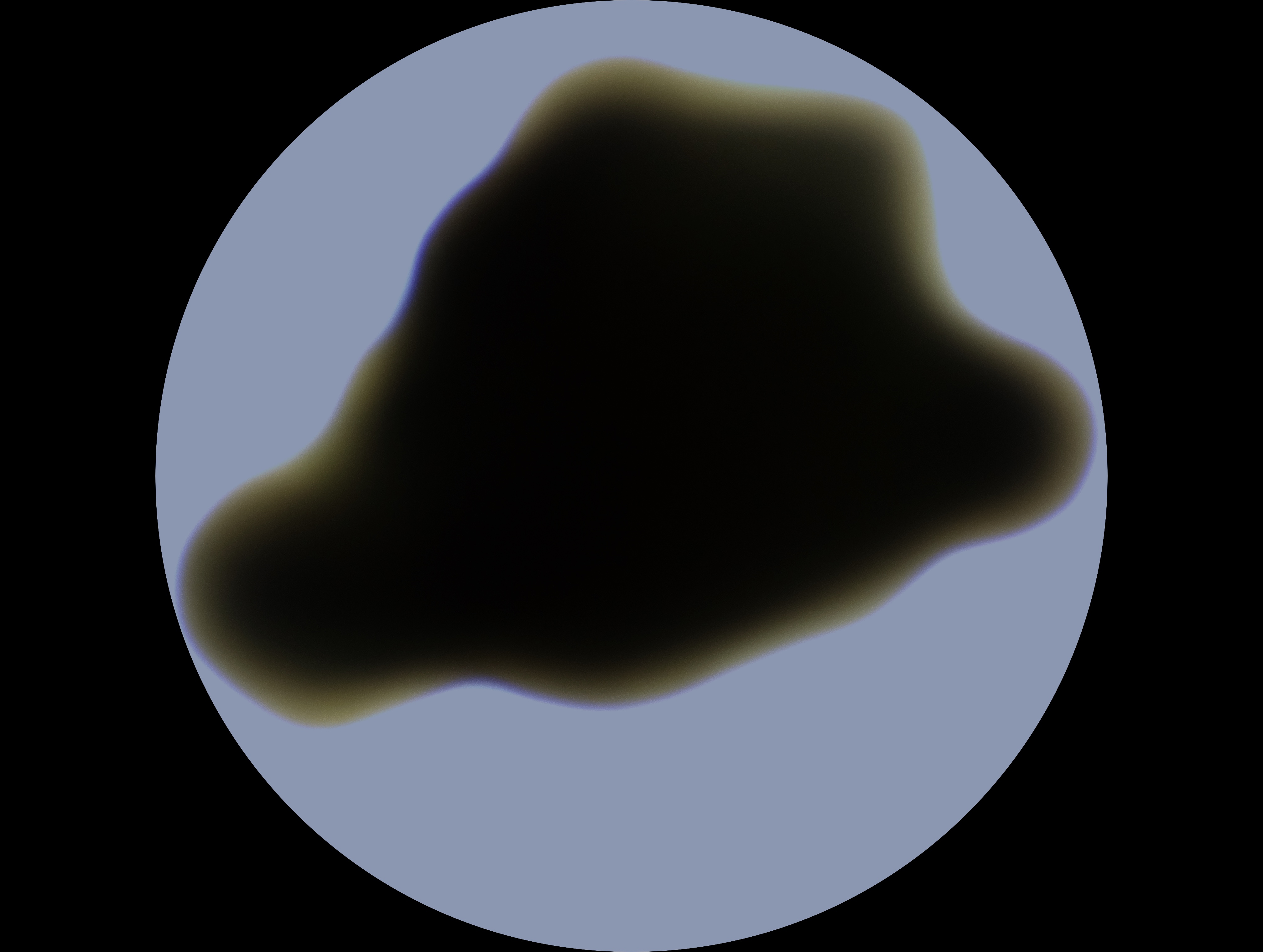

Supplement: Supplementary file 11 — Source data Fig. 3 [file 44319_2025_619_MOESM11_ESM.zip › Figure 3/C,D,F,G/Raw images_mask/OS_day90/MN 11C1 B C7 D90 2x/Day 90_0003.jpg]

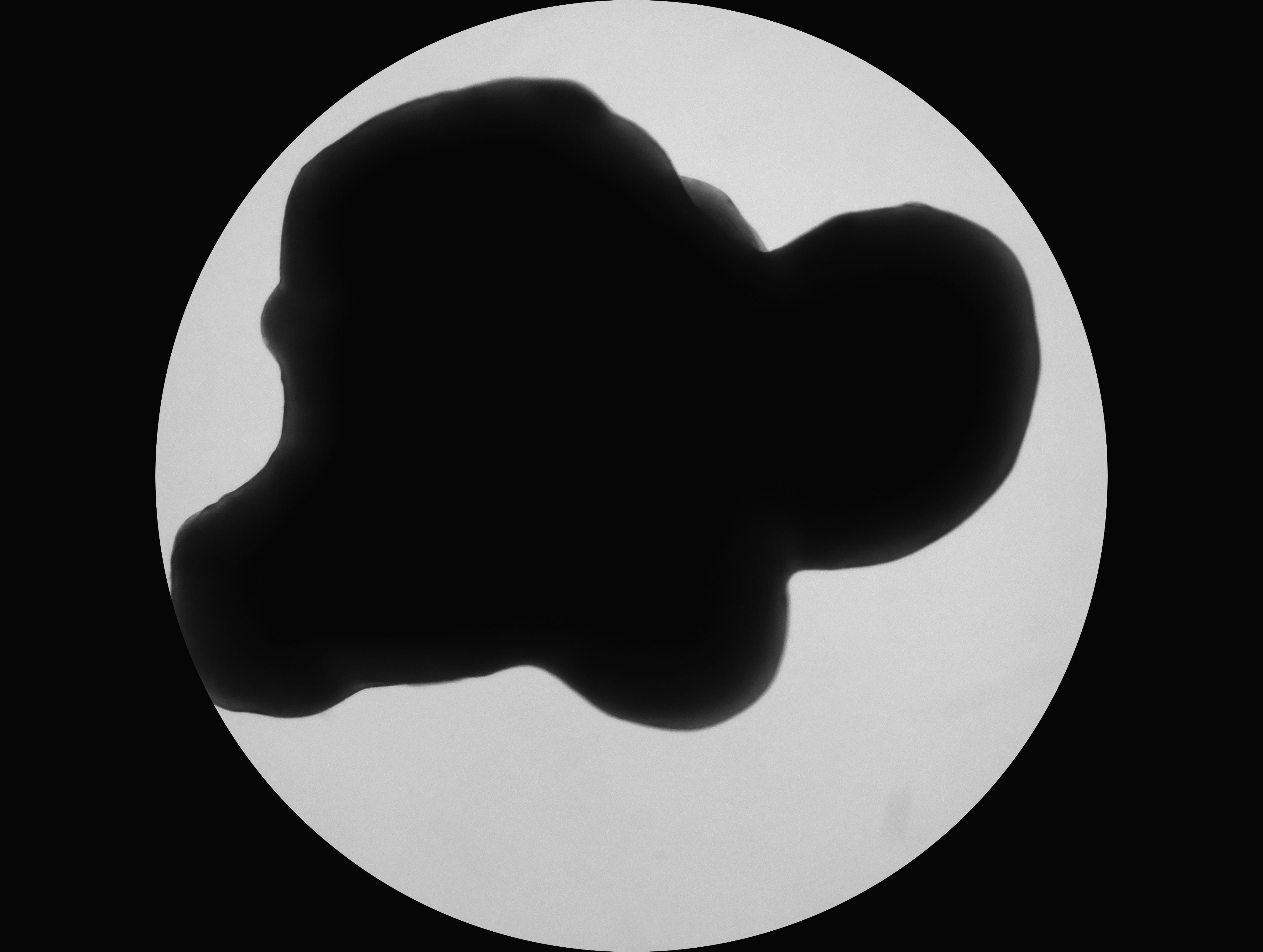

Supplement: Supplementary file 11 — Source data Fig. 3 [file 44319_2025_619_MOESM11_ESM.zip › Figure 3/C,D,F,G/Raw images_mask/OS_day90/MN 11C1 B C7 D90 2x/R_Day 90_0024.jpg]

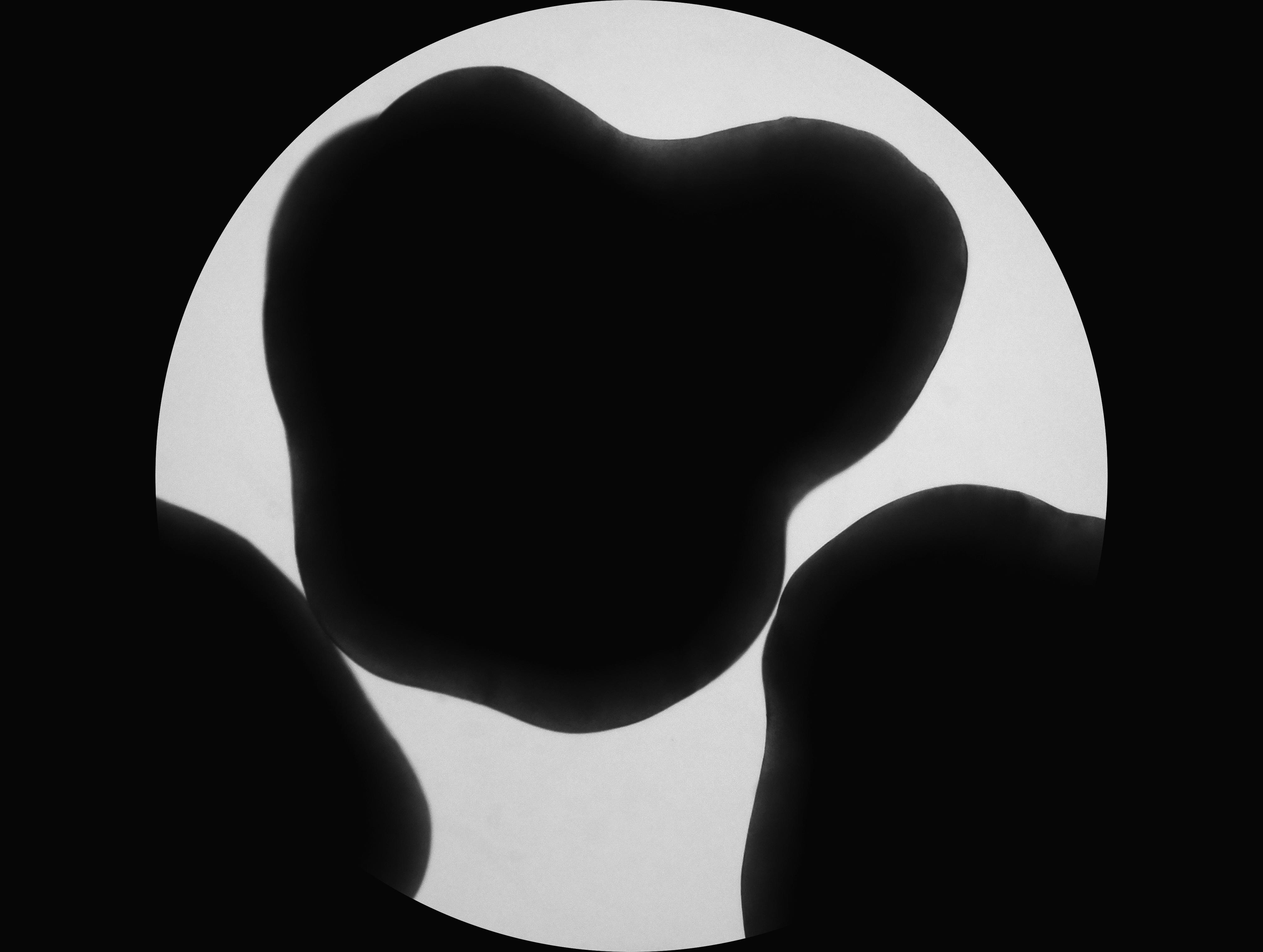

Supplement: Supplementary file 11 — Source data Fig. 3 [file 44319_2025_619_MOESM11_ESM.zip › Figure 3/C,D,F,G/Raw images_mask/OS_day90/MN 11C1 B C7 D90 2x/R_Day 90_0020.jpg]

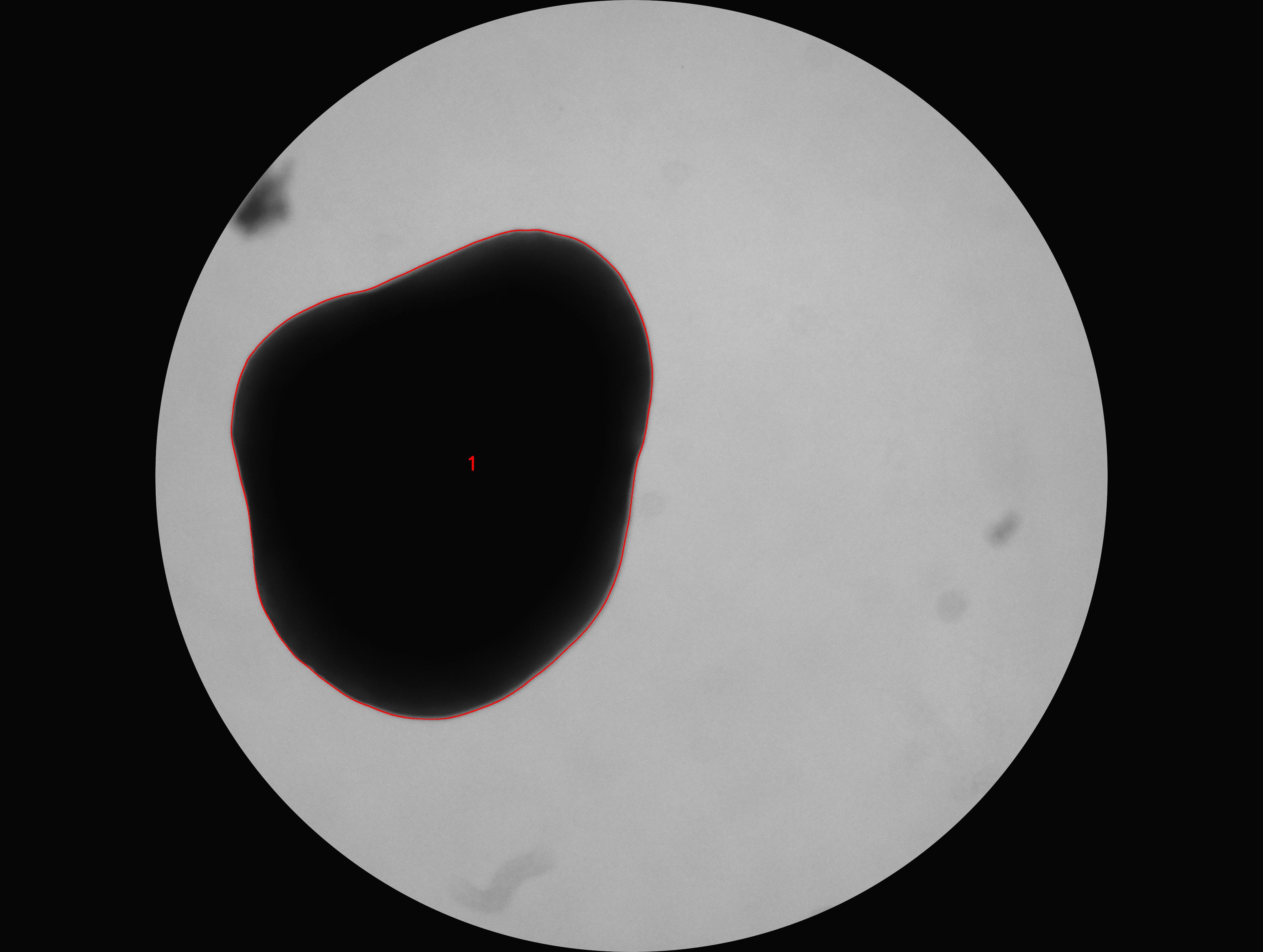

Supplement: Supplementary file 11 — Source data Fig. 3 [file 44319_2025_619_MOESM11_ESM.zip › Figure 3/C,D,F,G/Raw images_mask/OS_day90/MN 11C1 B C7 D90 2x/R_Day 90_0034.jpg]

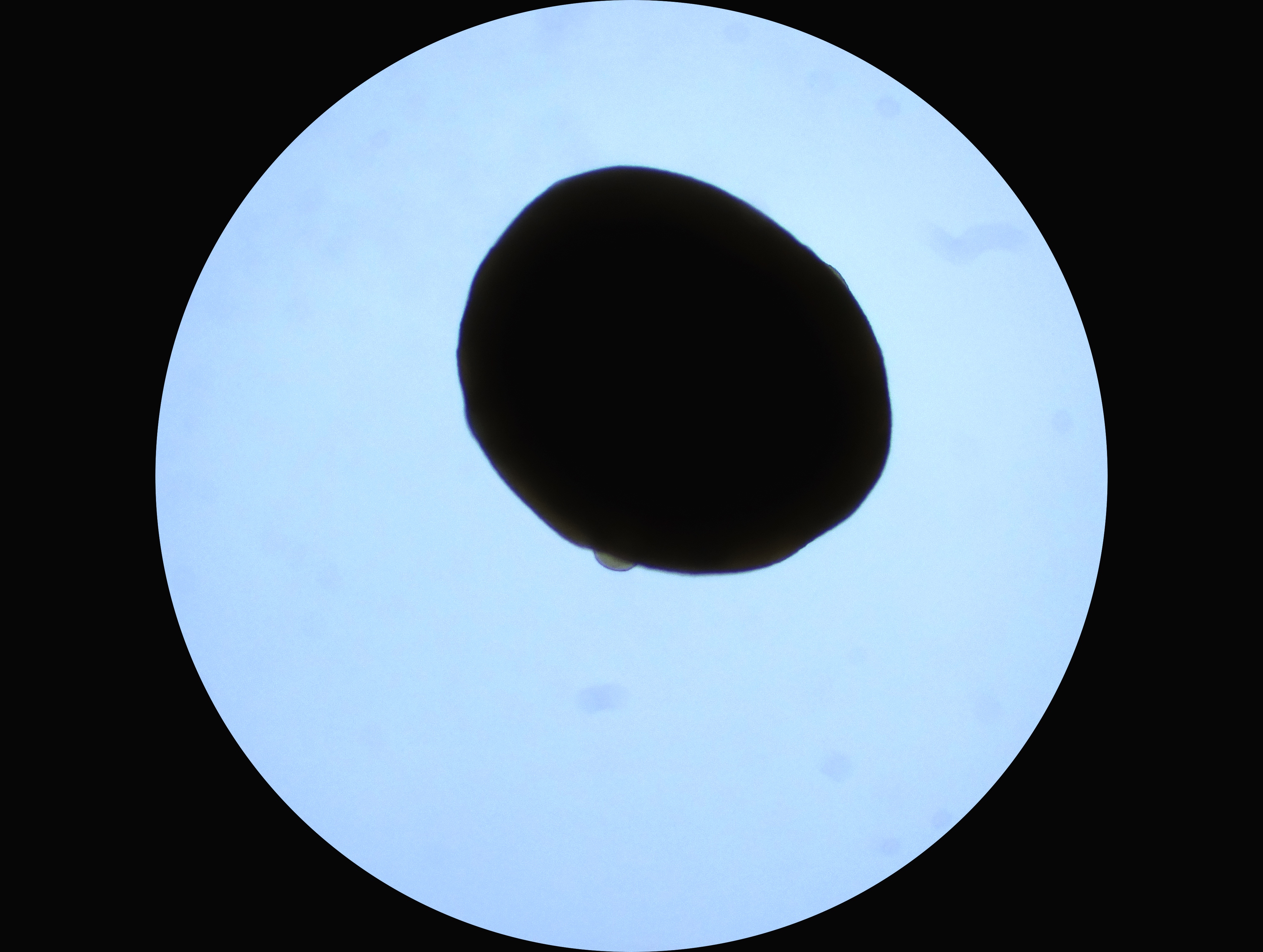

Supplement: Supplementary file 11 — Source data Fig. 3 [file 44319_2025_619_MOESM11_ESM.zip › Figure 3/C,D,F,G/Raw images_mask/OS_day90/MN 11C1 B C7 D90 2x/Day 90_0007.jpg]

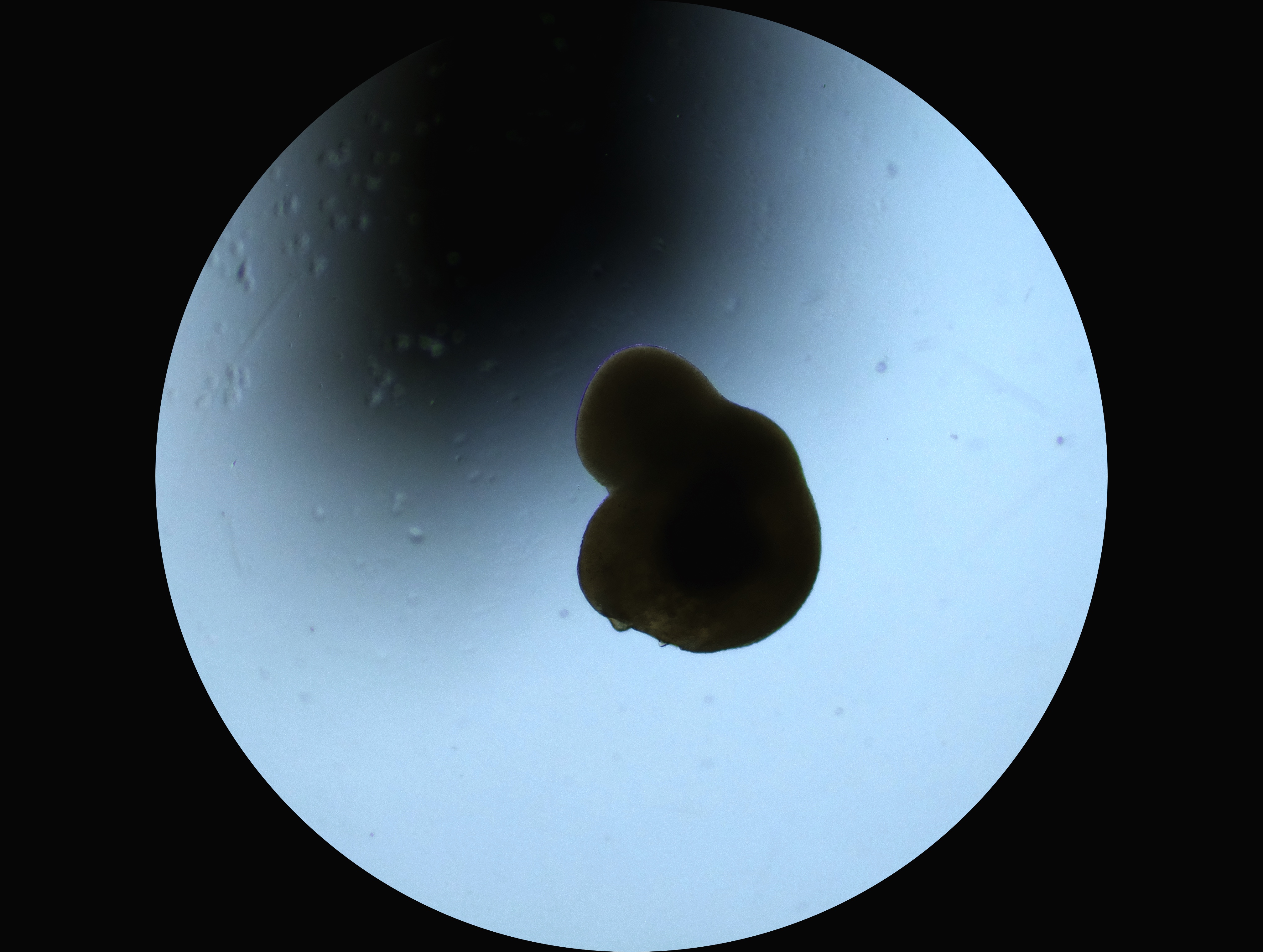

Supplement: Supplementary file 11 — Source data Fig. 3 [file 44319_2025_619_MOESM11_ESM.zip › Figure 3/C,D,F,G/Raw images_mask/OS_day90/MN 11C1 B C7 D90 2x/Day 90_0013.jpg]

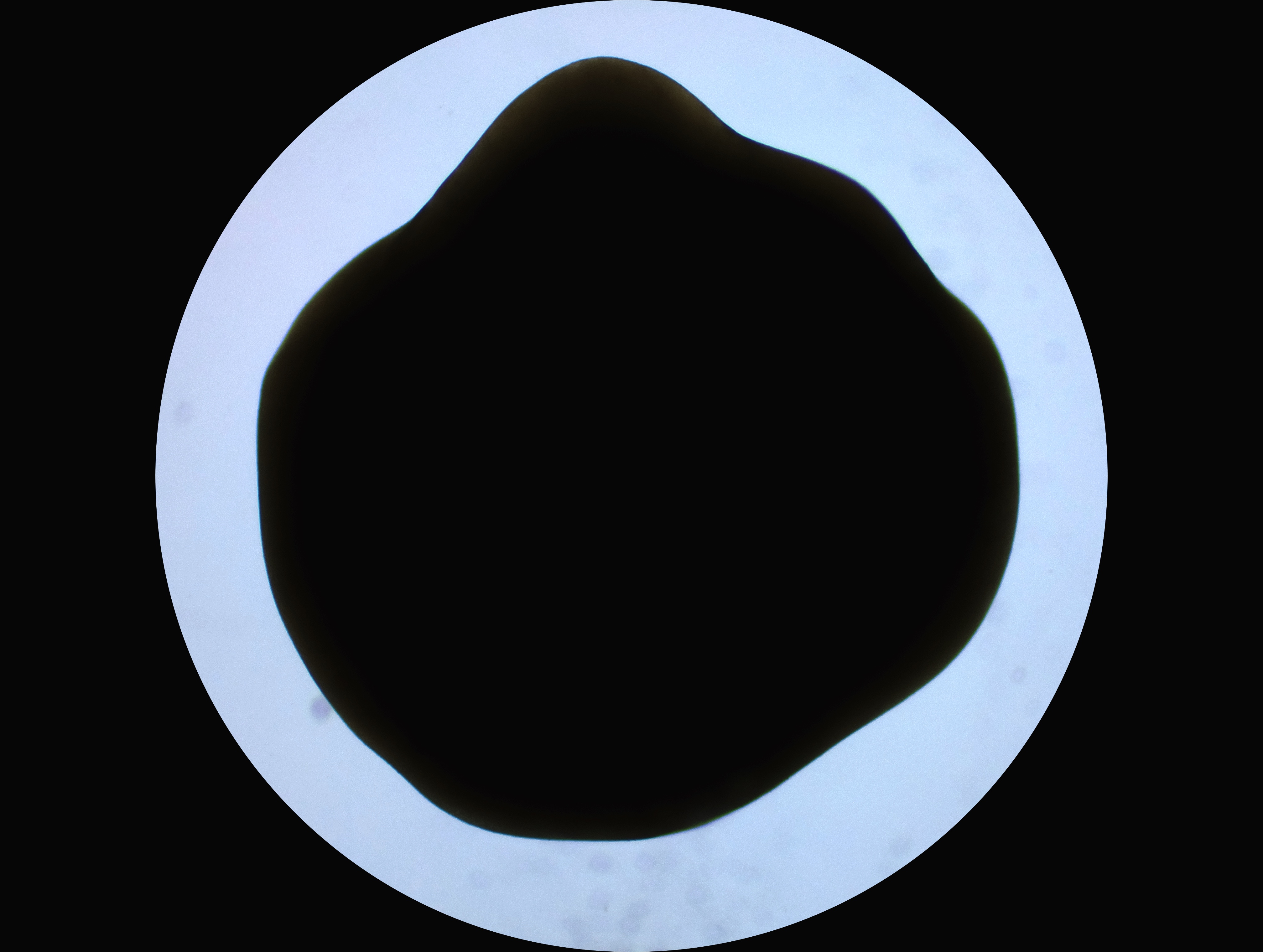

Supplement: Supplementary file 11 — Source data Fig. 3 [file 44319_2025_619_MOESM11_ESM.zip › Figure 3/C,D,F,G/Raw images_mask/OS_day90/MN 11C1 B C7 D90 2x/Day 90_0012.jpg]

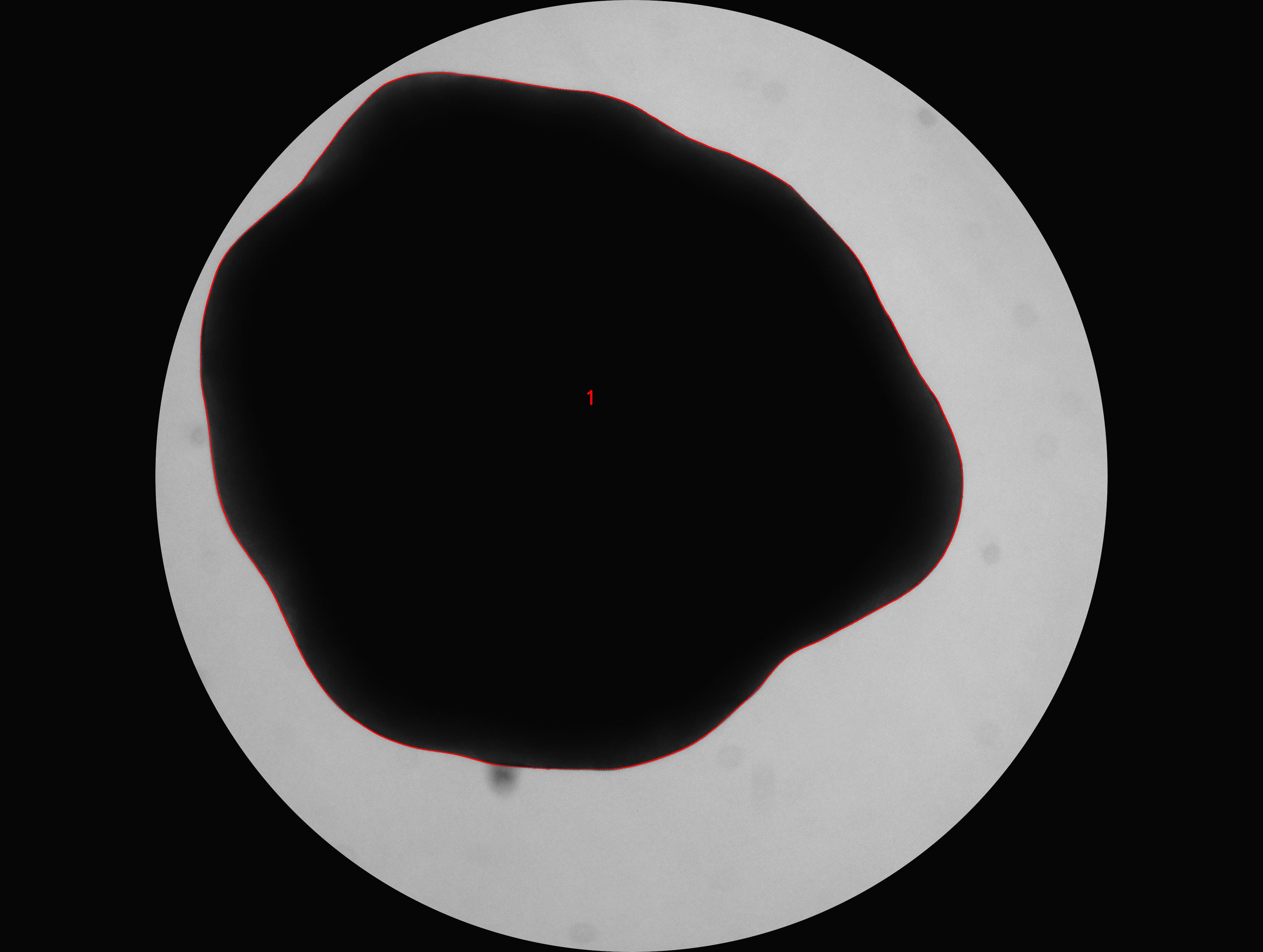

Supplement: Supplementary file 11 — Source data Fig. 3 [file 44319_2025_619_MOESM11_ESM.zip › Figure 3/C,D,F,G/Raw images_mask/OS_day90/MN 11C1 B C7 D90 2x/R_Day 90_0035.jpg]

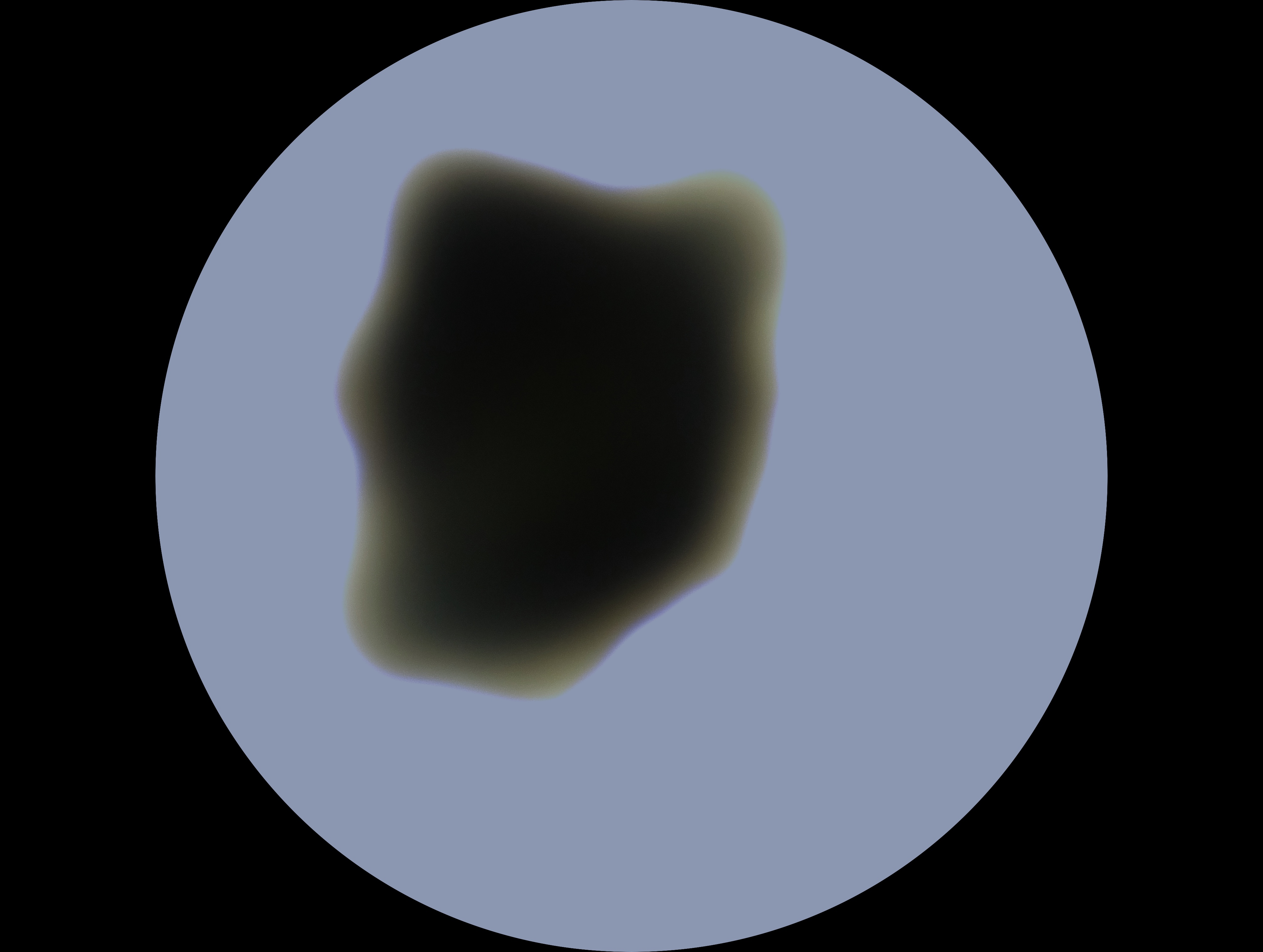

Supplement: Supplementary file 11 — Source data Fig. 3 [file 44319_2025_619_MOESM11_ESM.zip › Figure 3/C,D,F,G/Raw images_mask/OS_day90/MN 11C1 B C7 D90 2x/Day 90_0004.jpg]

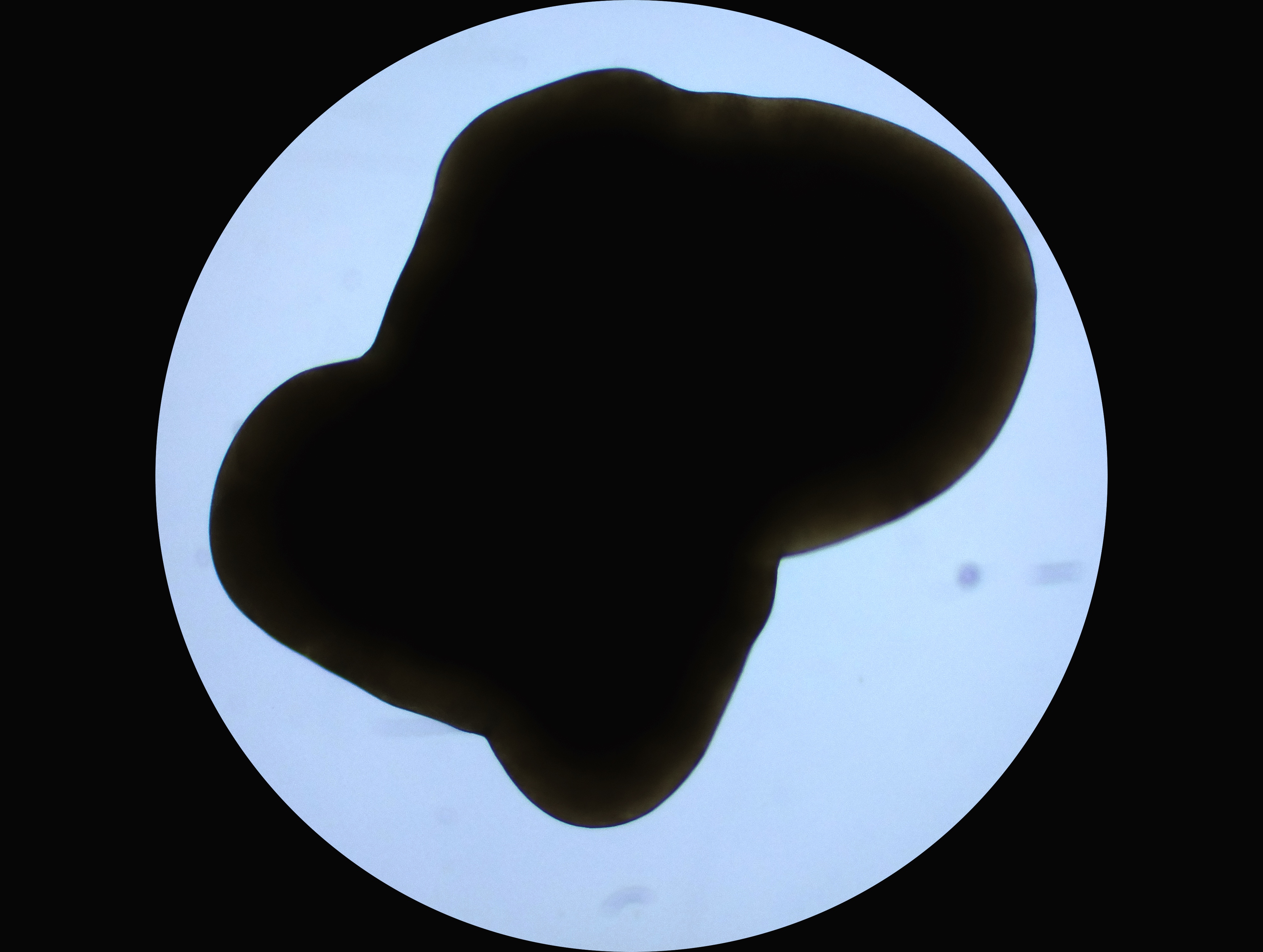

Supplement: Supplementary file 11 — Source data Fig. 3 [file 44319_2025_619_MOESM11_ESM.zip › Figure 3/C,D,F,G/Raw images_mask/OS_day90/MN 11C1 B C7 D90 2x/Day 90_0011.jpg]

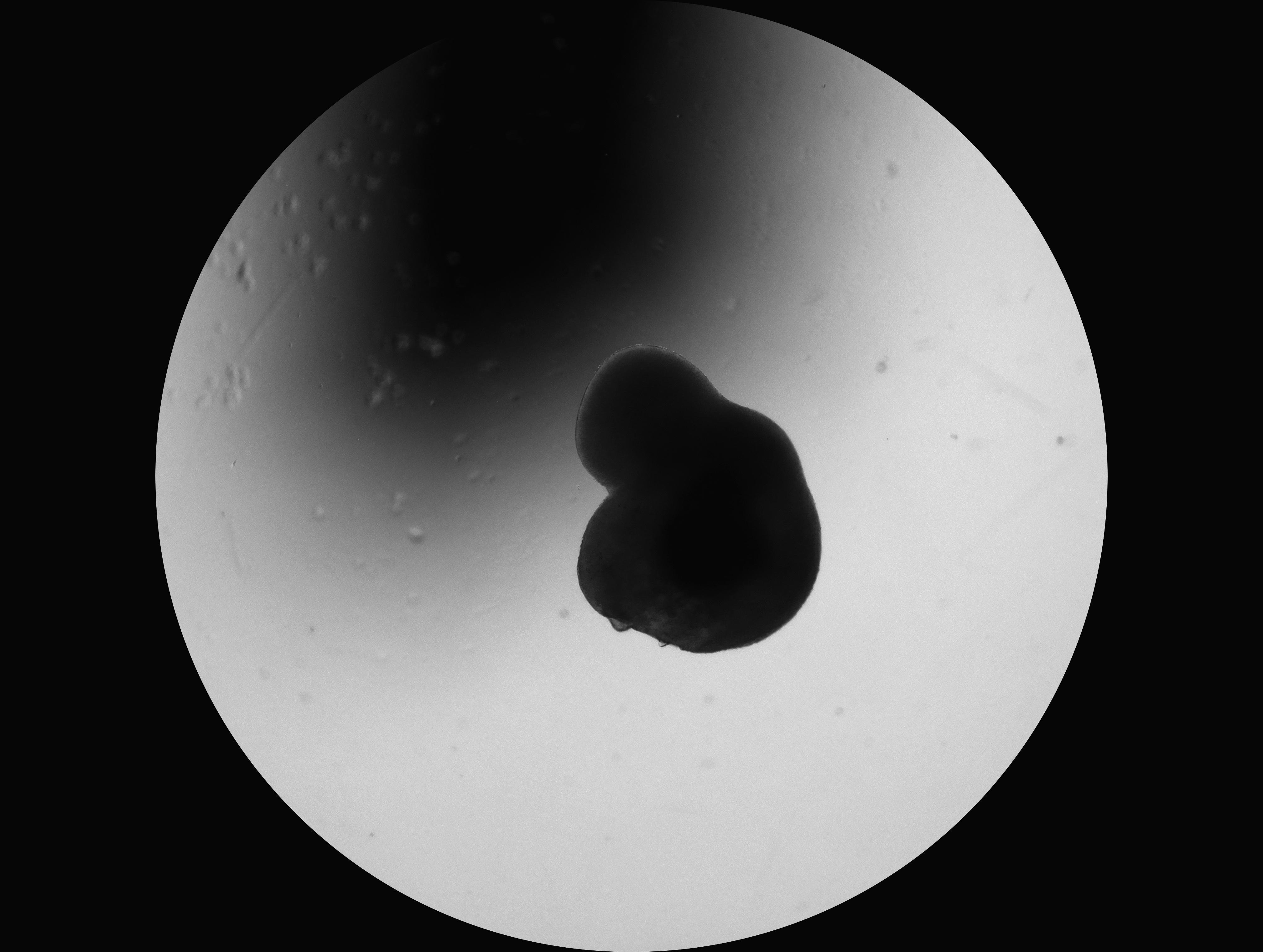

Supplement: Supplementary file 11 — Source data Fig. 3 [file 44319_2025_619_MOESM11_ESM.zip › Figure 3/C,D,F,G/Raw images_mask/OS_day90/MN 11C1 B C7 D90 2x/R_Day 90_0013.jpg]

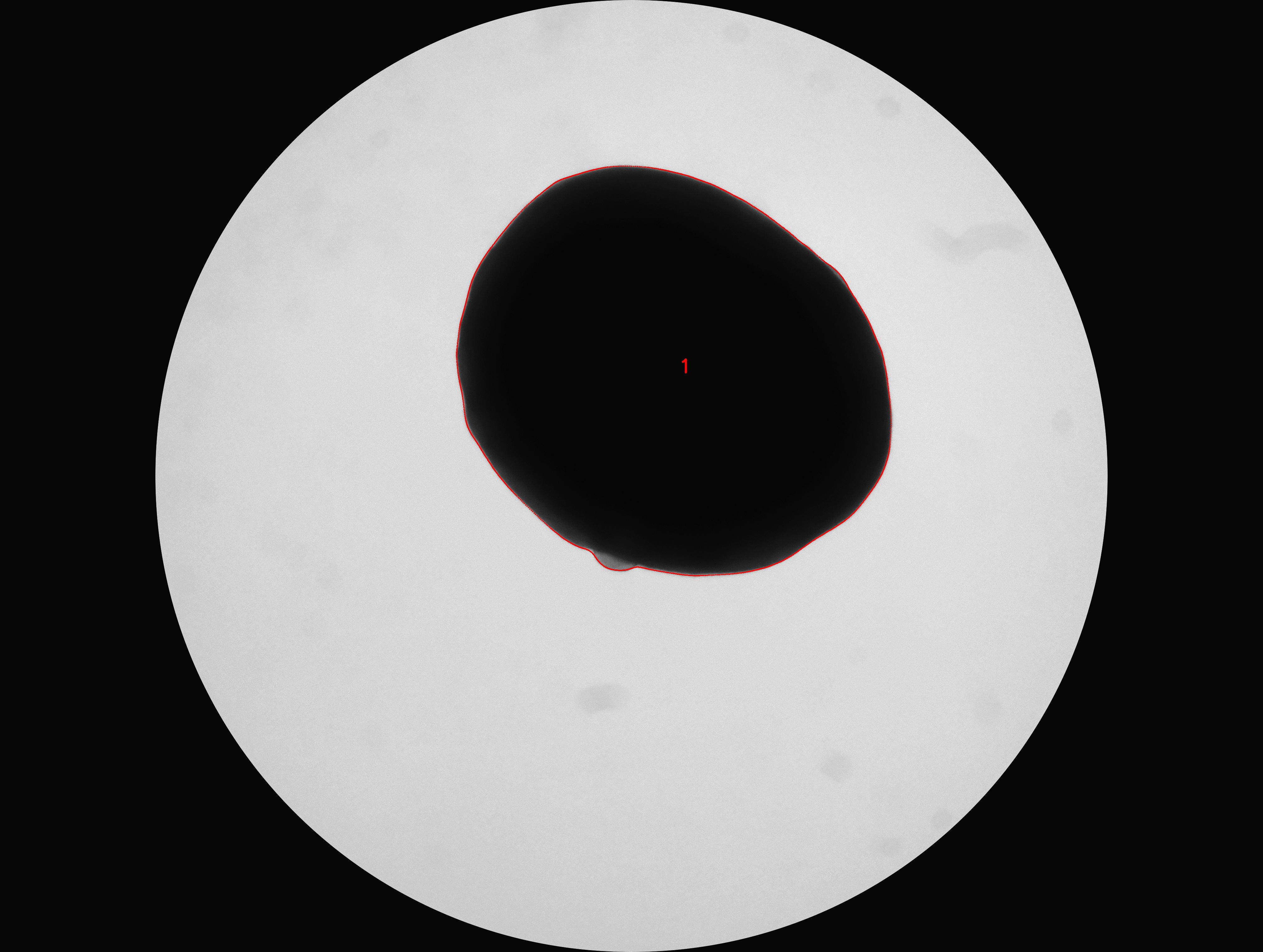

Supplement: Supplementary file 11 — Source data Fig. 3 [file 44319_2025_619_MOESM11_ESM.zip › Figure 3/C,D,F,G/Raw images_mask/OS_day90/MN 11C1 B C7 D90 2x/R_Day 90_0007.jpg]

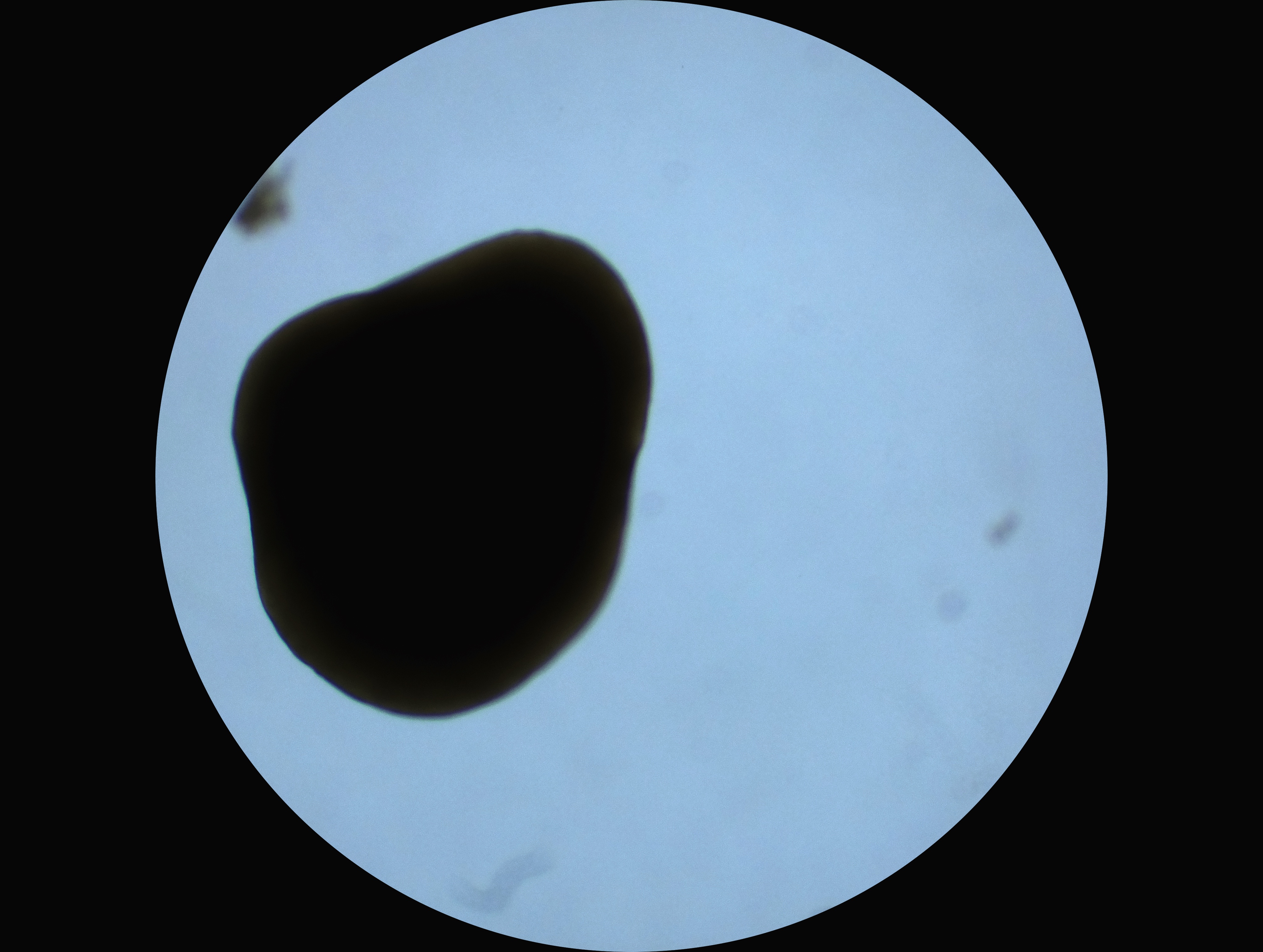

Supplement: Supplementary file 11 — Source data Fig. 3 [file 44319_2025_619_MOESM11_ESM.zip › Figure 3/C,D,F,G/Raw images_mask/OS_day90/MN 11C1 B C7 D90 2x/Day 90_0034.jpg]

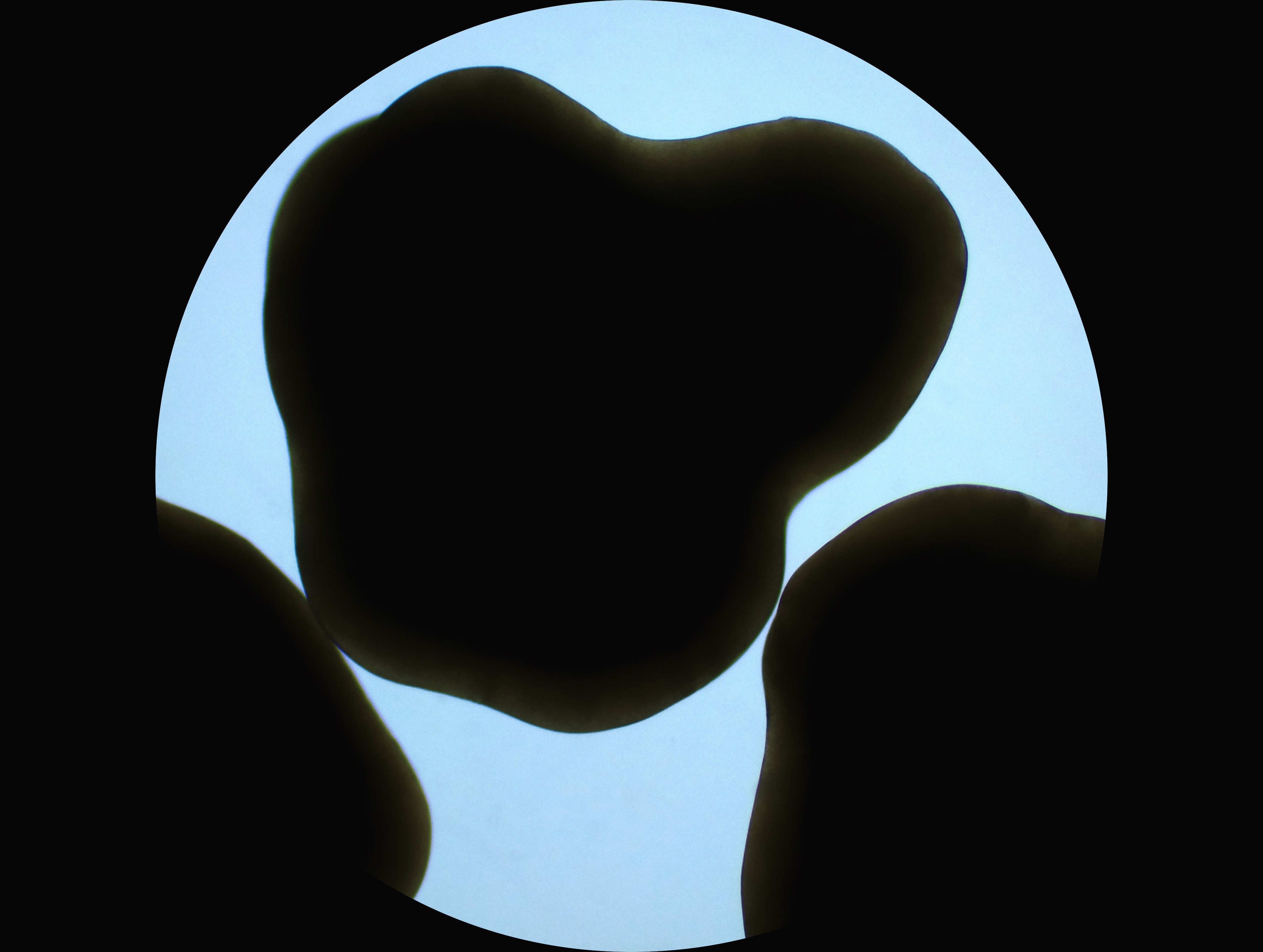

Supplement: Supplementary file 11 — Source data Fig. 3 [file 44319_2025_619_MOESM11_ESM.zip › Figure 3/C,D,F,G/Raw images_mask/OS_day90/MN 11C1 B C7 D90 2x/Day 90_0020.jpg]

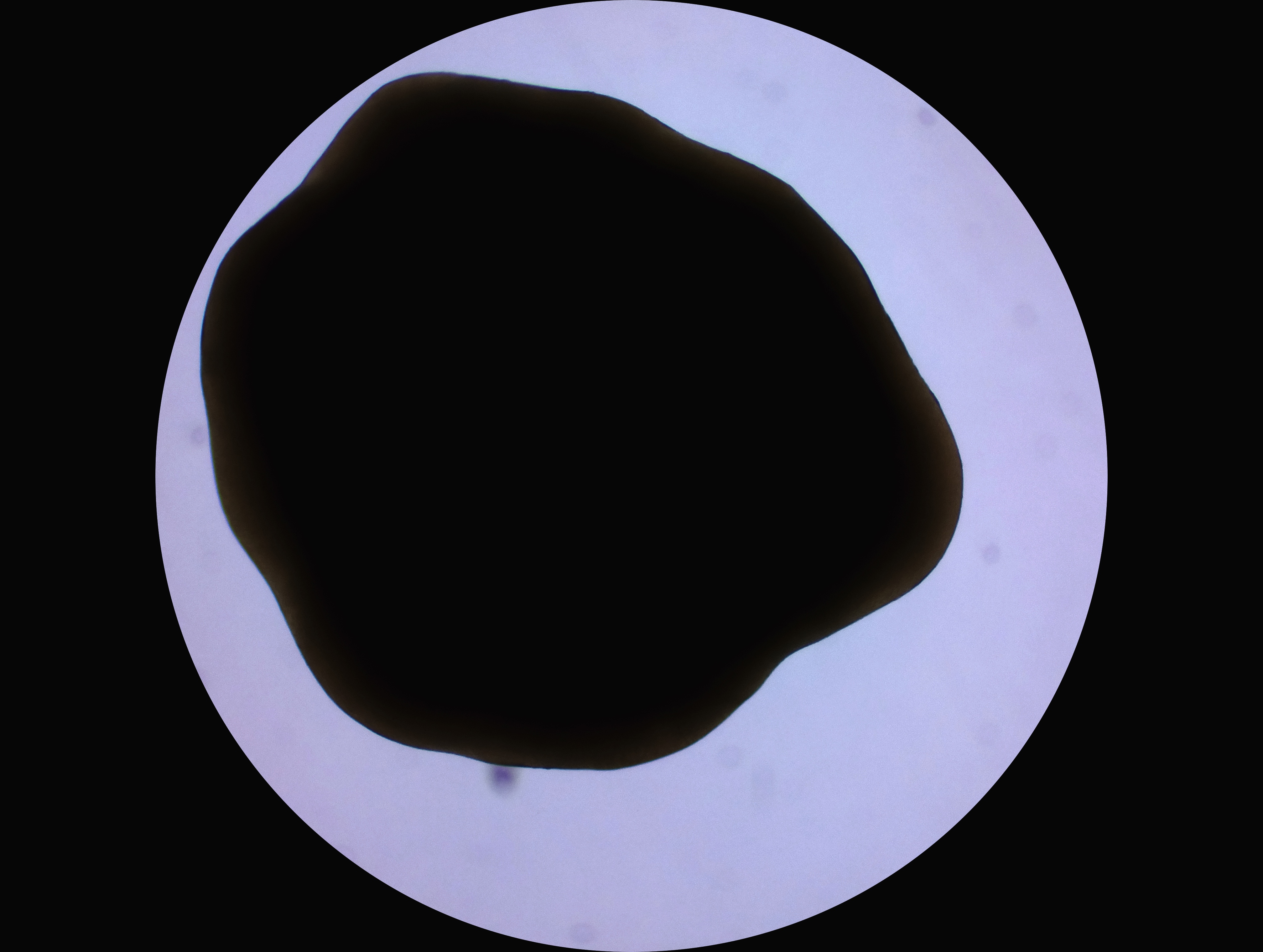

Supplement: Supplementary file 11 — Source data Fig. 3 [file 44319_2025_619_MOESM11_ESM.zip › Figure 3/C,D,F,G/Raw images_mask/OS_day90/MN 11C1 B C7 D90 2x/Day 90_0035.jpg]

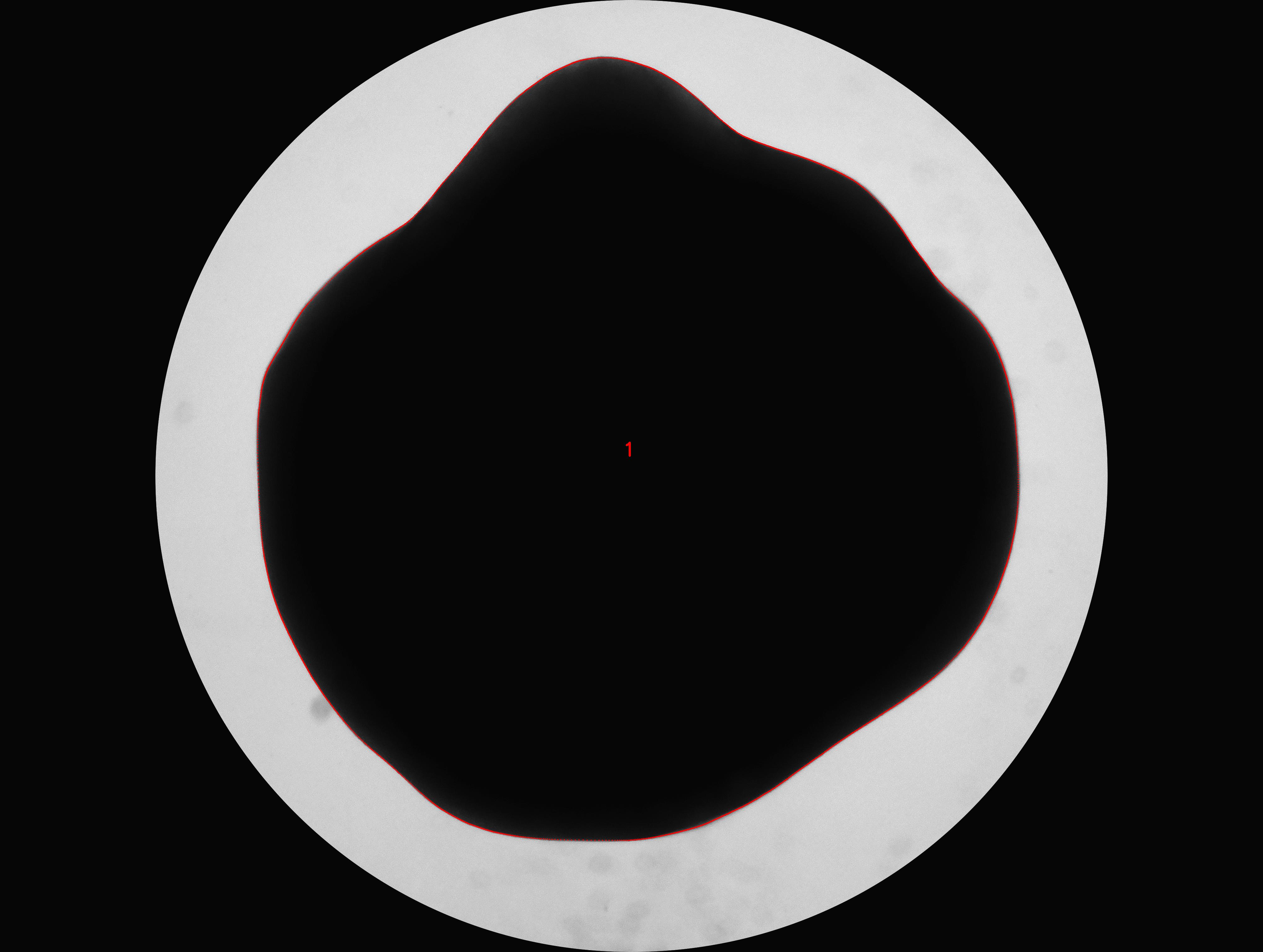

Supplement: Supplementary file 11 — Source data Fig. 3 [file 44319_2025_619_MOESM11_ESM.zip › Figure 3/C,D,F,G/Raw images_mask/OS_day90/MN 11C1 B C7 D90 2x/R_Day 90_0012.jpg]

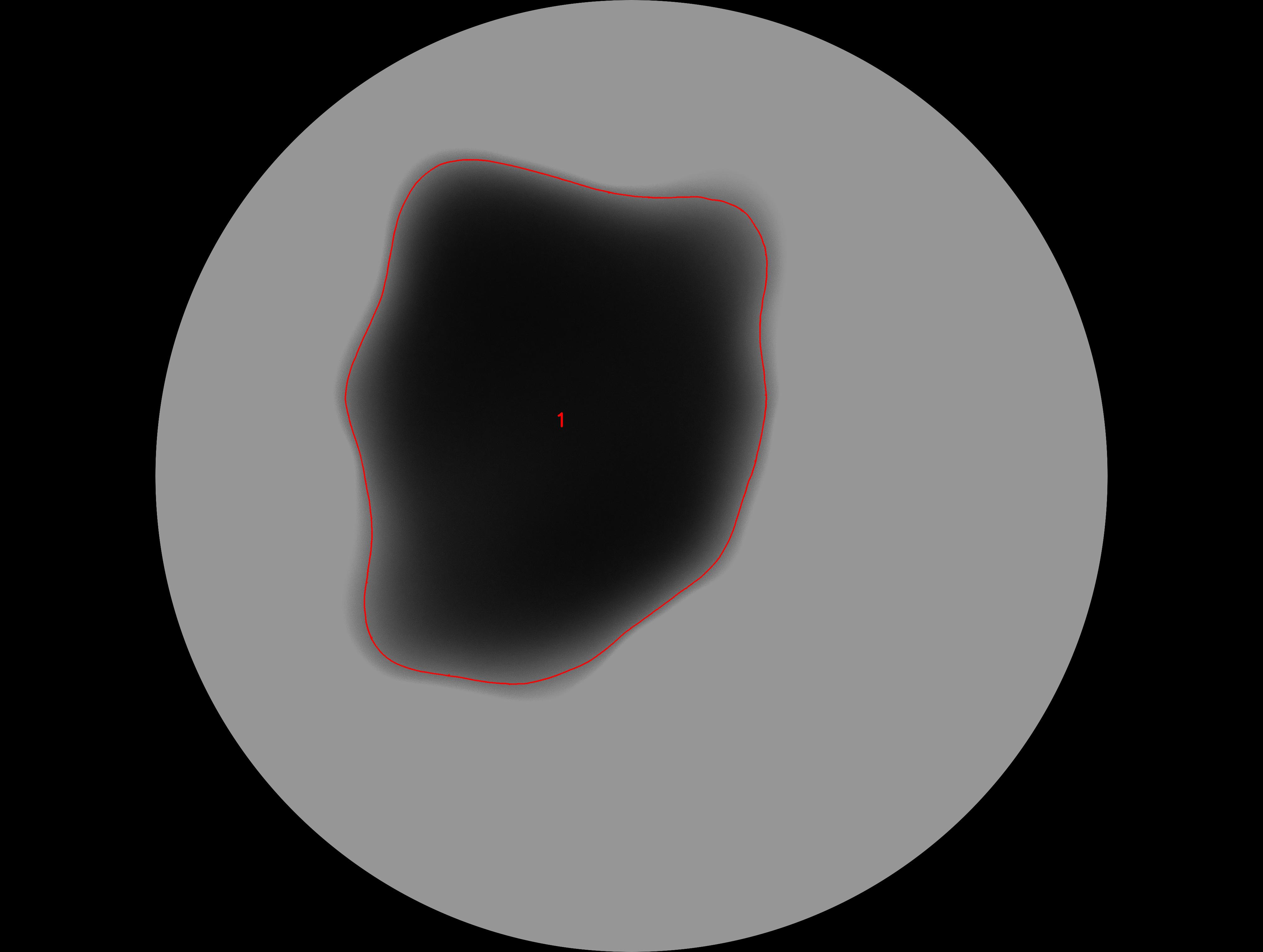

Supplement: Supplementary file 11 — Source data Fig. 3 [file 44319_2025_619_MOESM11_ESM.zip › Figure 3/C,D,F,G/Raw images_mask/OS_day90/MN 11C1 B C7 D90 2x/R_Day 90_0004.jpg]

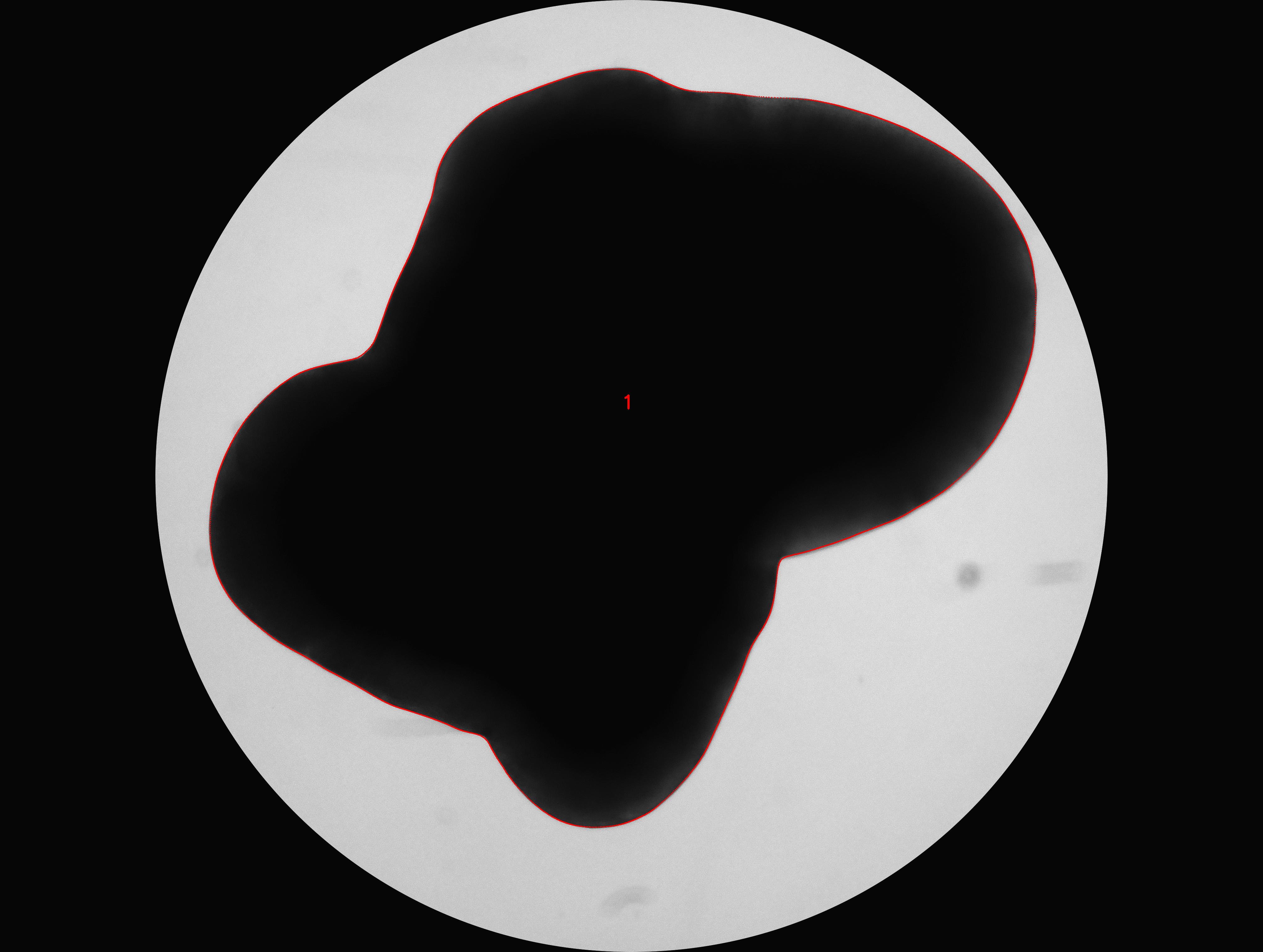

Supplement: Supplementary file 11 — Source data Fig. 3 [file 44319_2025_619_MOESM11_ESM.zip › Figure 3/C,D,F,G/Raw images_mask/OS_day90/MN 11C1 B C7 D90 2x/R_Day 90_0011.jpg]

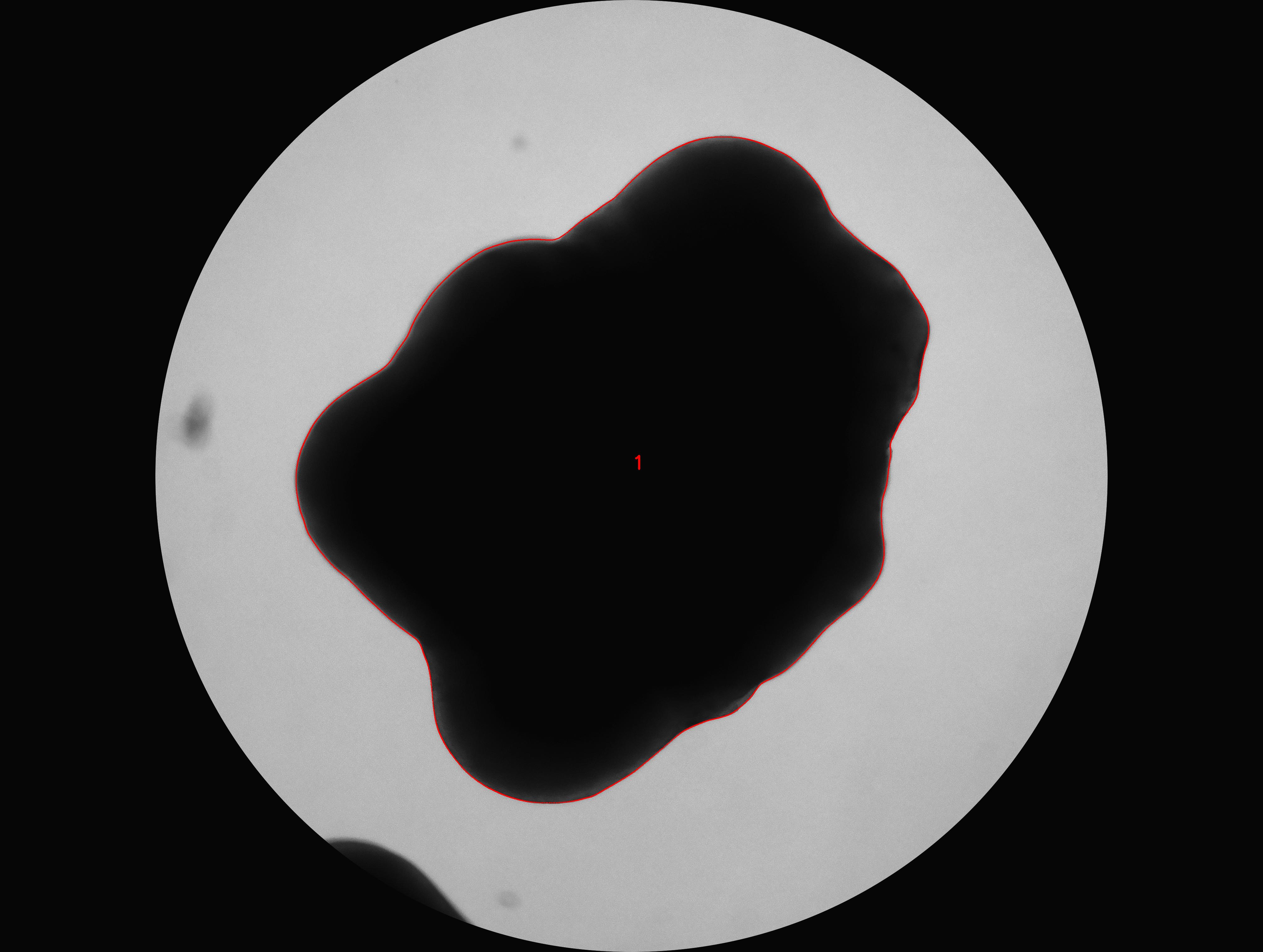

Supplement: Supplementary file 11 — Source data Fig. 3 [file 44319_2025_619_MOESM11_ESM.zip › Figure 3/C,D,F,G/Raw images_mask/OS_day90/MN 11C1 B C7 D90 2x/R_Day 90_0029.jpg]

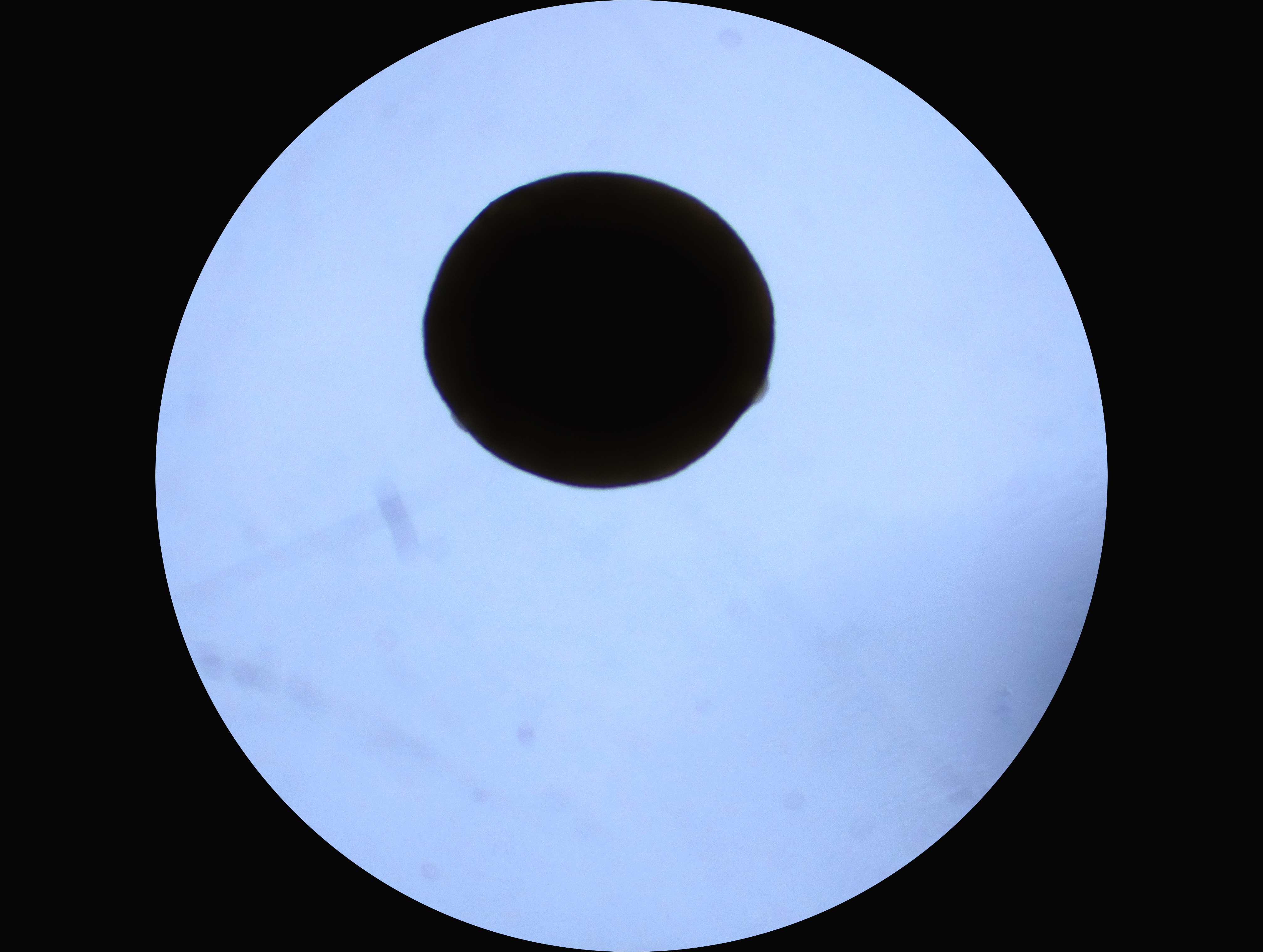

Supplement: Supplementary file 11 — Source data Fig. 3 [file 44319_2025_619_MOESM11_ESM.zip › Figure 3/C,D,F,G/Raw images_mask/OS_day90/MN 11C1 B C7 D90 2x/Day 90_0026.jpg]

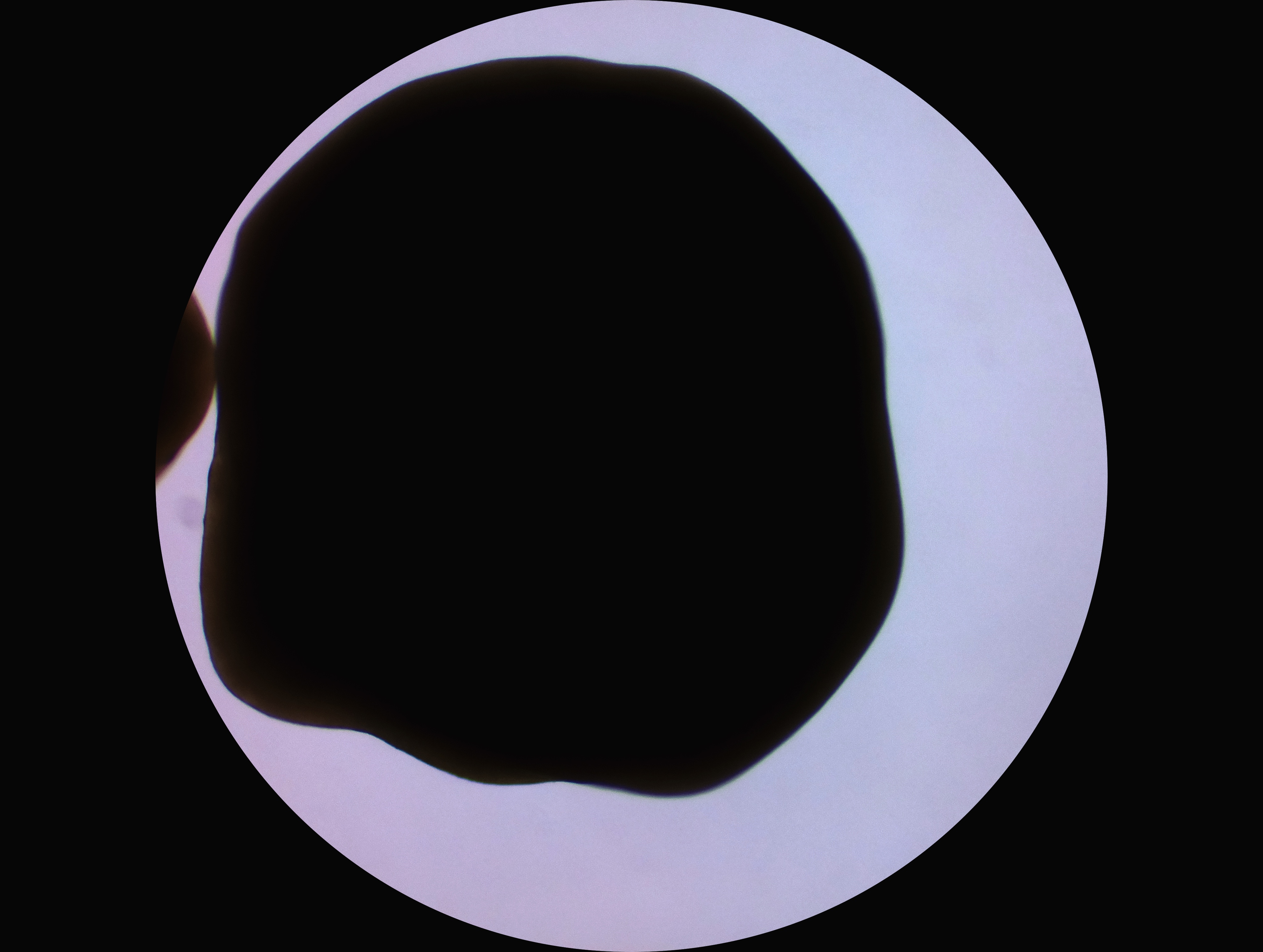

Supplement: Supplementary file 11 — Source data Fig. 3 [file 44319_2025_619_MOESM11_ESM.zip › Figure 3/C,D,F,G/Raw images_mask/OS_day90/MN 11C1 B C7 D90 2x/Day 90_0033.jpg]

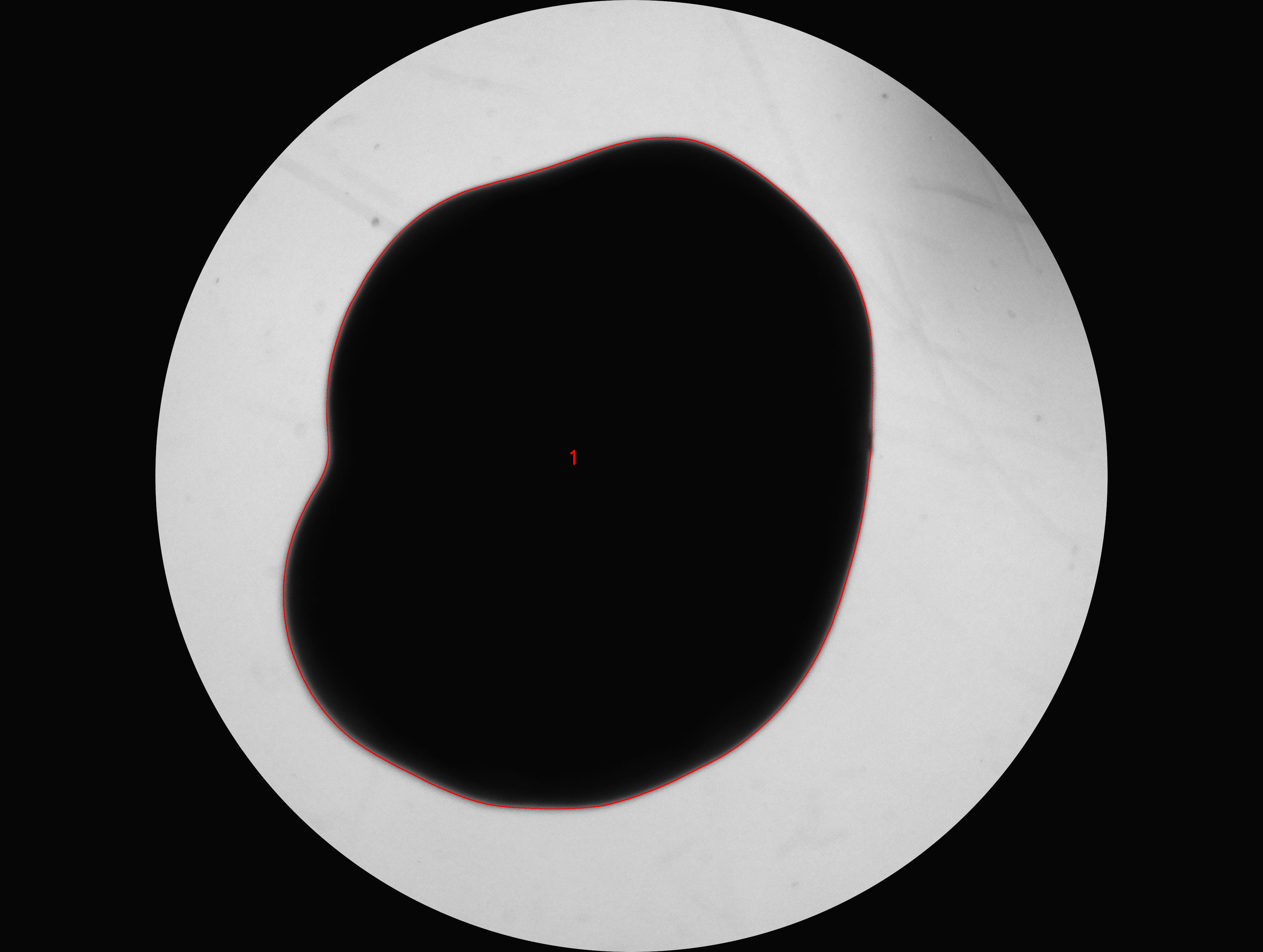

Supplement: Supplementary file 11 — Source data Fig. 3 [file 44319_2025_619_MOESM11_ESM.zip › Figure 3/C,D,F,G/Raw images_mask/OS_day90/MN 11C1 B C7 D90 2x/R_Day 90_0014.jpg]

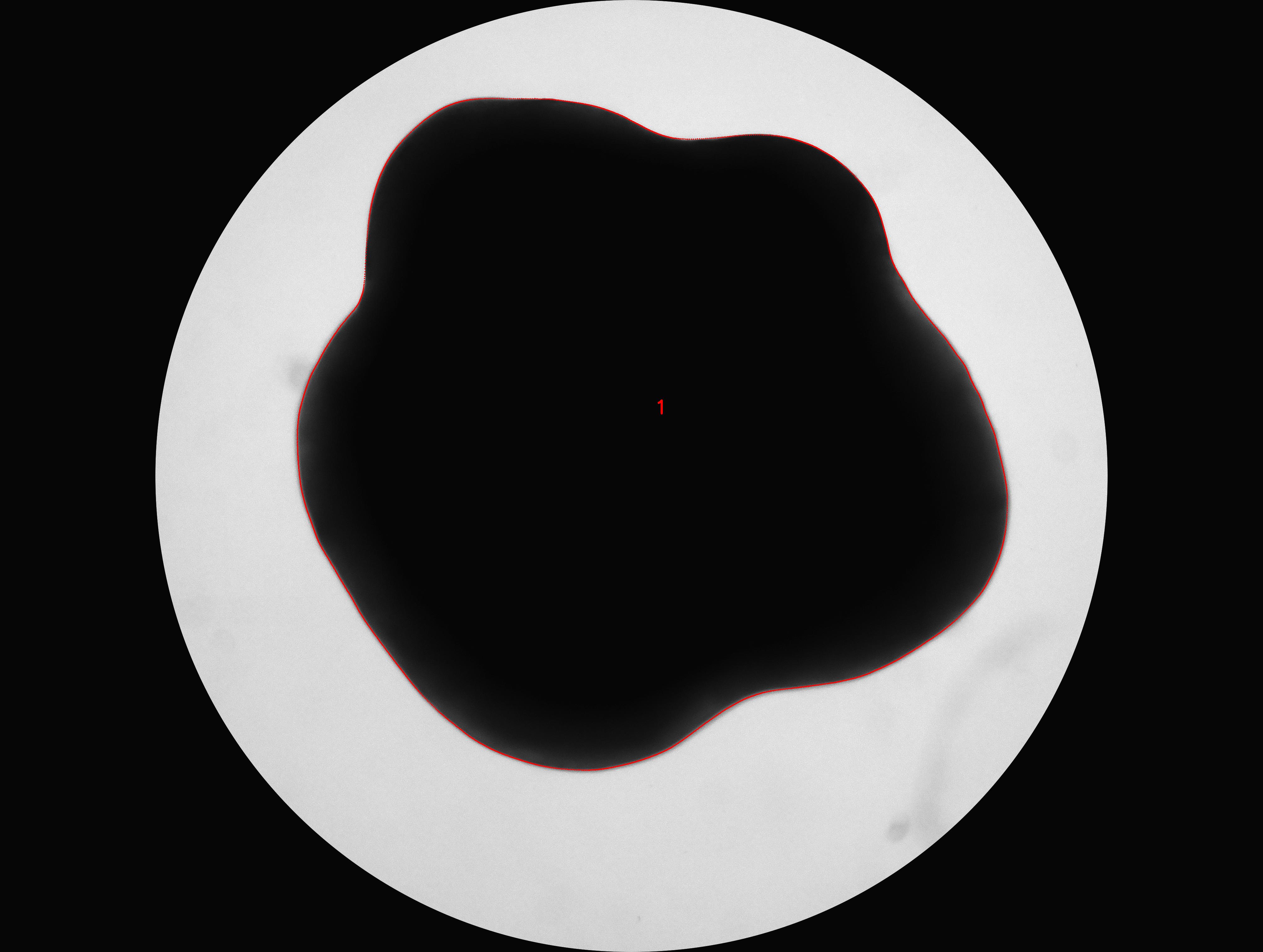

Supplement: Supplementary file 11 — Source data Fig. 3 [file 44319_2025_619_MOESM11_ESM.zip › Figure 3/C,D,F,G/Raw images_mask/OS_day90/MN 11C1 B C7 D90 2x/R_Day 90_0016.jpg]

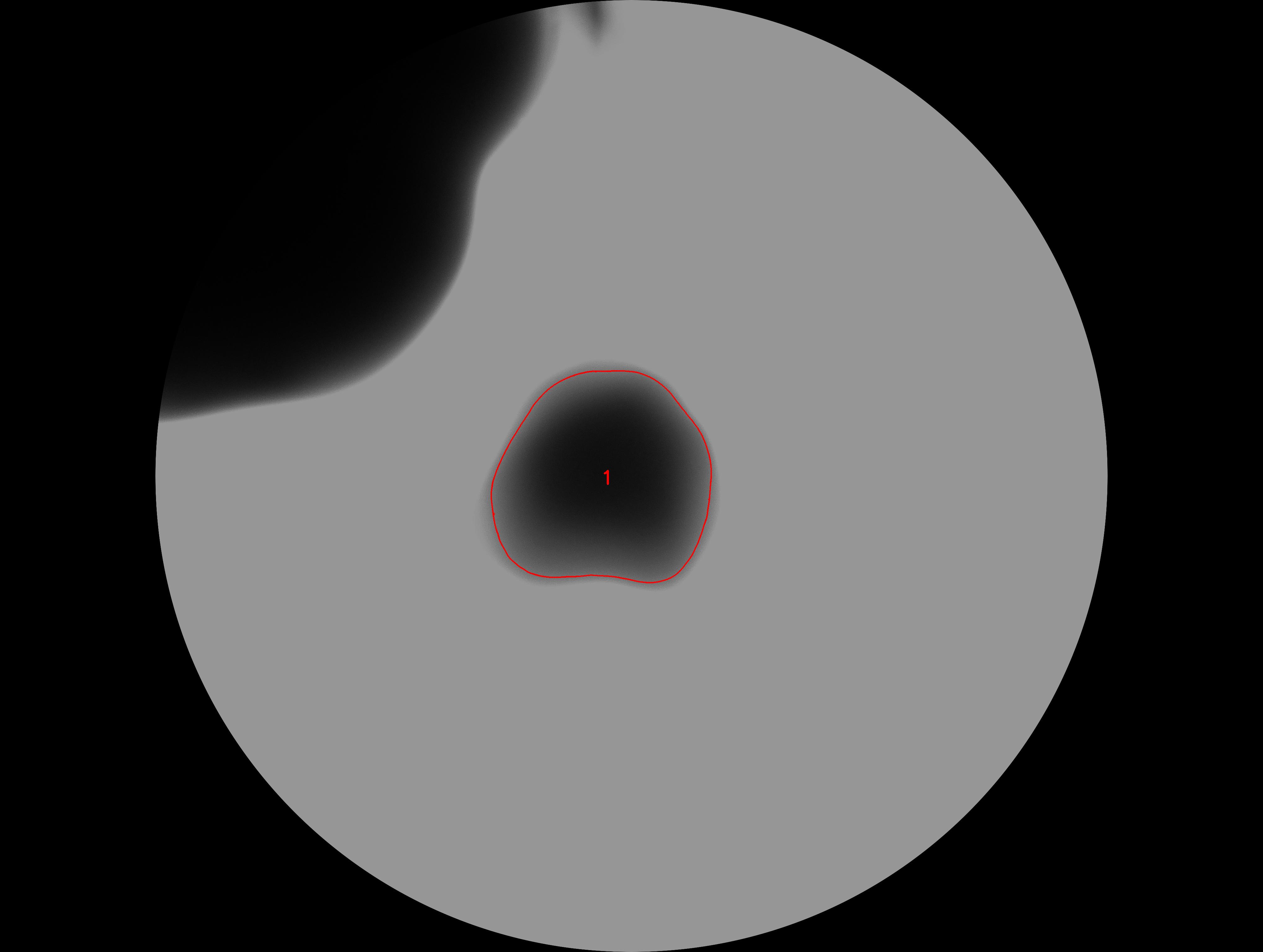

Supplement: Supplementary file 11 — Source data Fig. 3 [file 44319_2025_619_MOESM11_ESM.zip › Figure 3/C,D,F,G/Raw images_mask/OS_day90/MN 11C1 B C7 D90 2x/R_Day 90_0002.jpg]

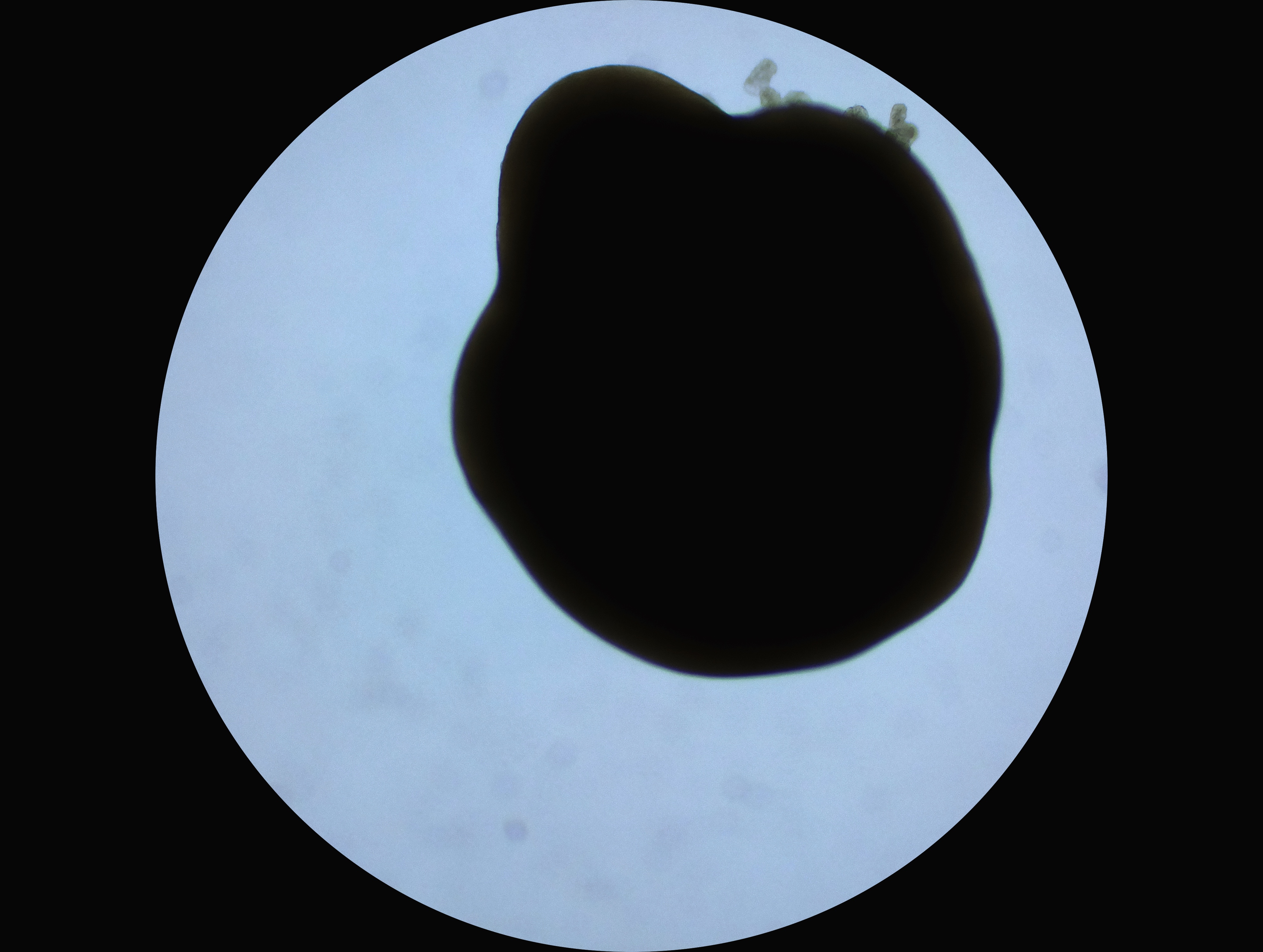

Supplement: Supplementary file 11 — Source data Fig. 3 [file 44319_2025_619_MOESM11_ESM.zip › Figure 3/C,D,F,G/Raw images_mask/OS_day90/MN 11C1 B C7 D90 2x/Day 90_0031.jpg]

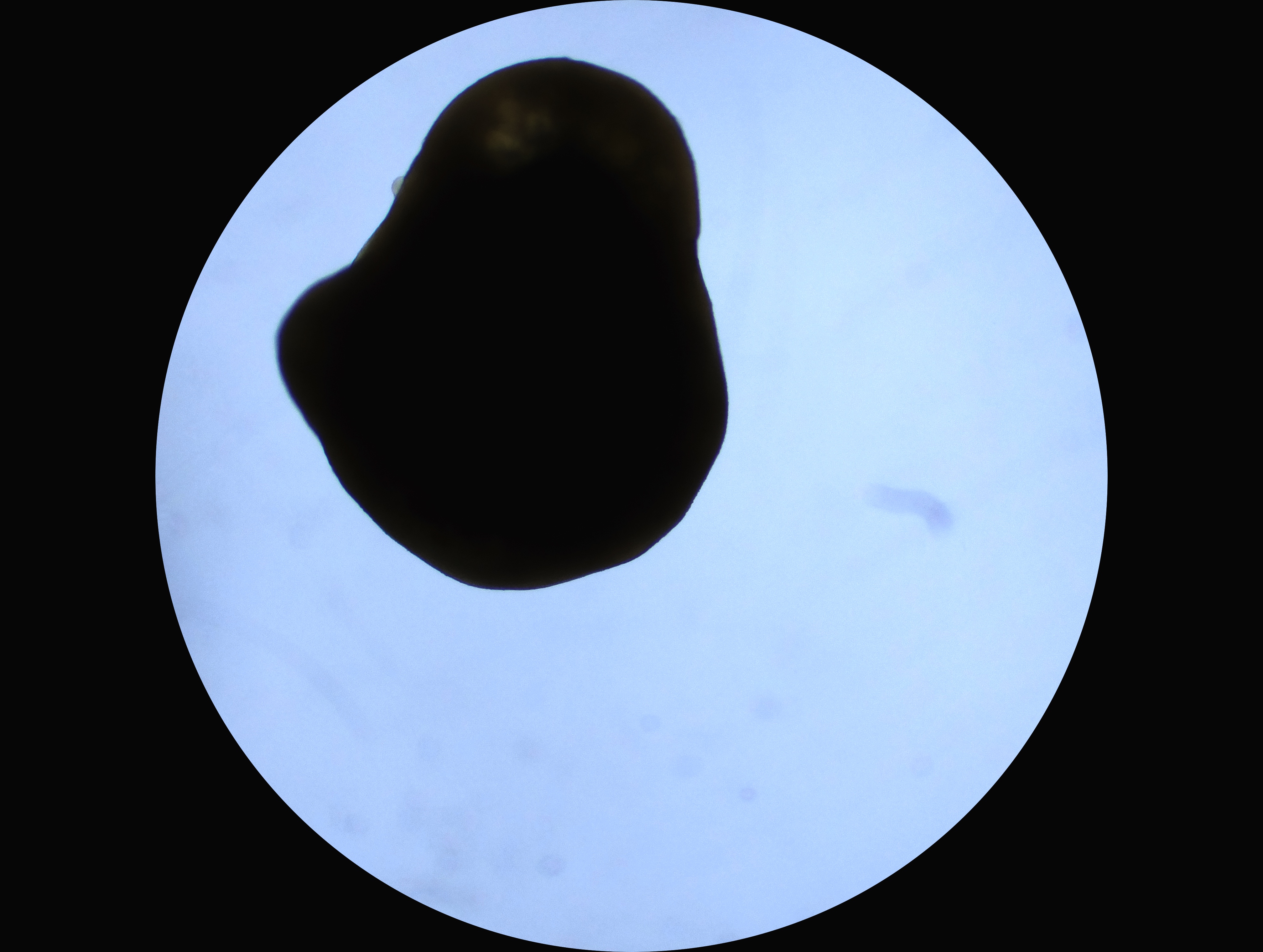

Supplement: Supplementary file 11 — Source data Fig. 3 [file 44319_2025_619_MOESM11_ESM.zip › Figure 3/C,D,F,G/Raw images_mask/OS_day90/MN 11C1 B C7 D90 2x/Day 90_0025.jpg]

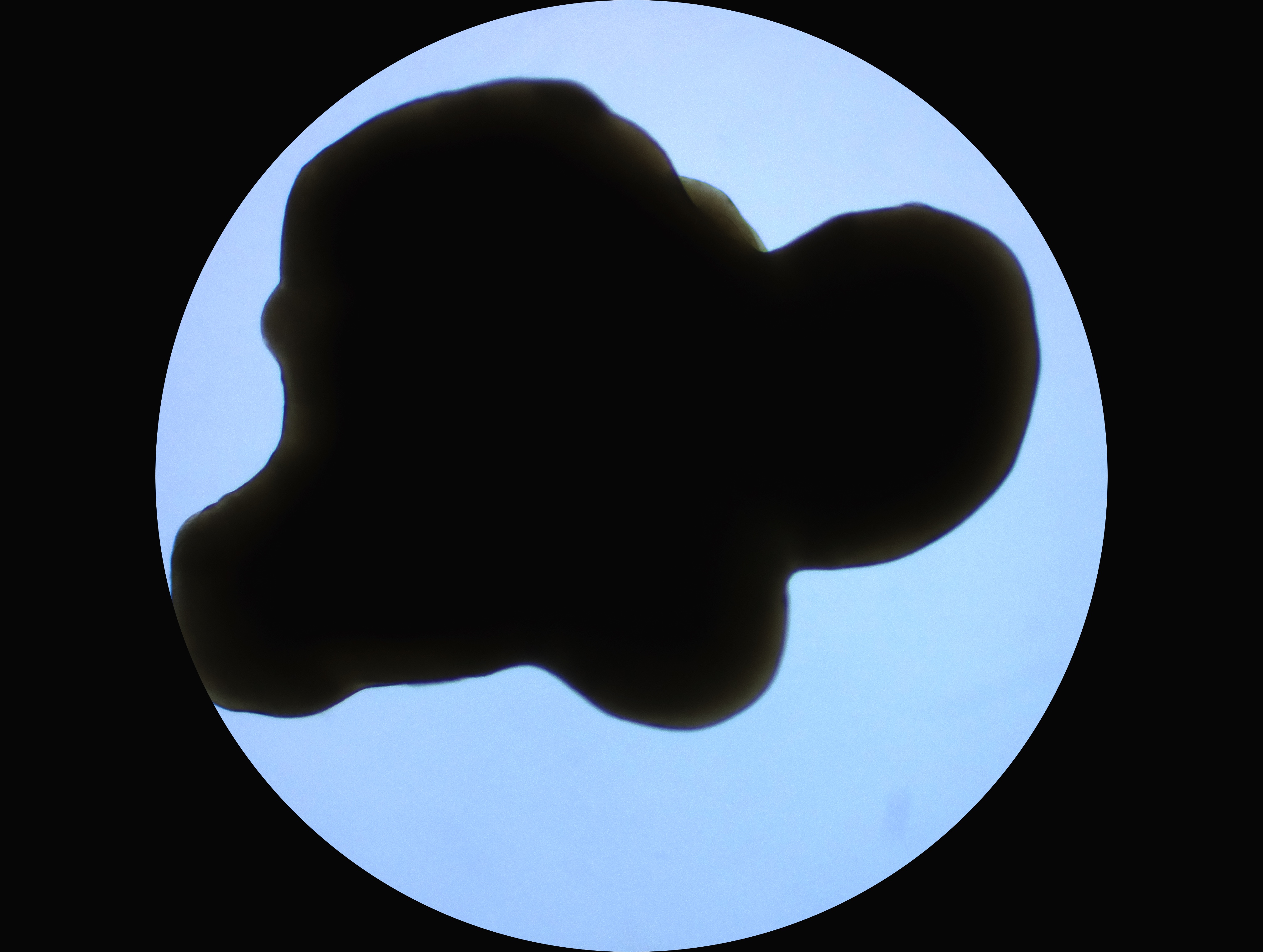

Supplement: Supplementary file 11 — Source data Fig. 3 [file 44319_2025_619_MOESM11_ESM.zip › Figure 3/C,D,F,G/Raw images_mask/OS_day90/MN 11C1 B C7 D90 2x/Day 90_0024.jpg]

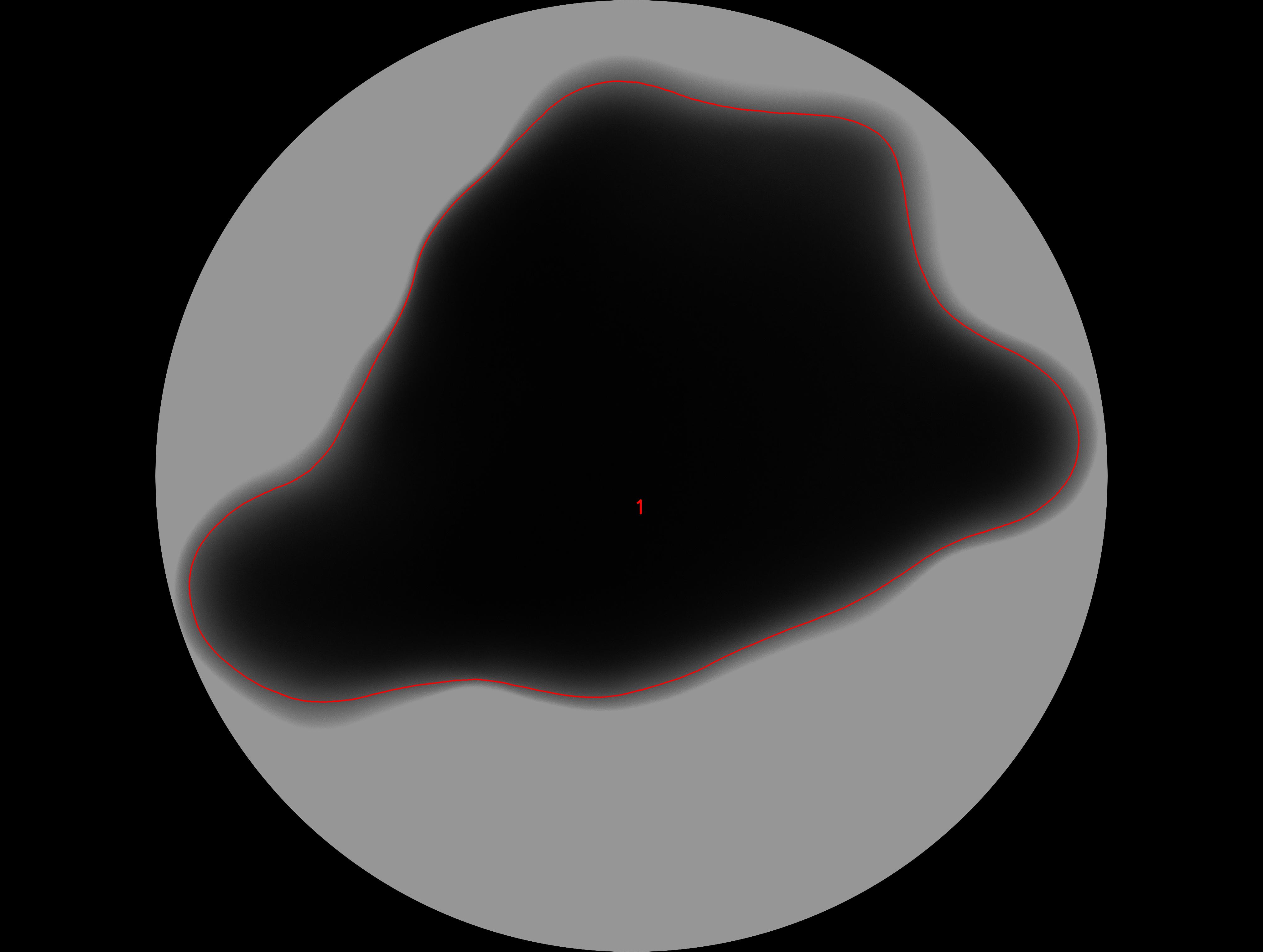

Supplement: Supplementary file 11 — Source data Fig. 3 [file 44319_2025_619_MOESM11_ESM.zip › Figure 3/C,D,F,G/Raw images_mask/OS_day90/MN 11C1 B C7 D90 2x/R_Day 90_0003.jpg]

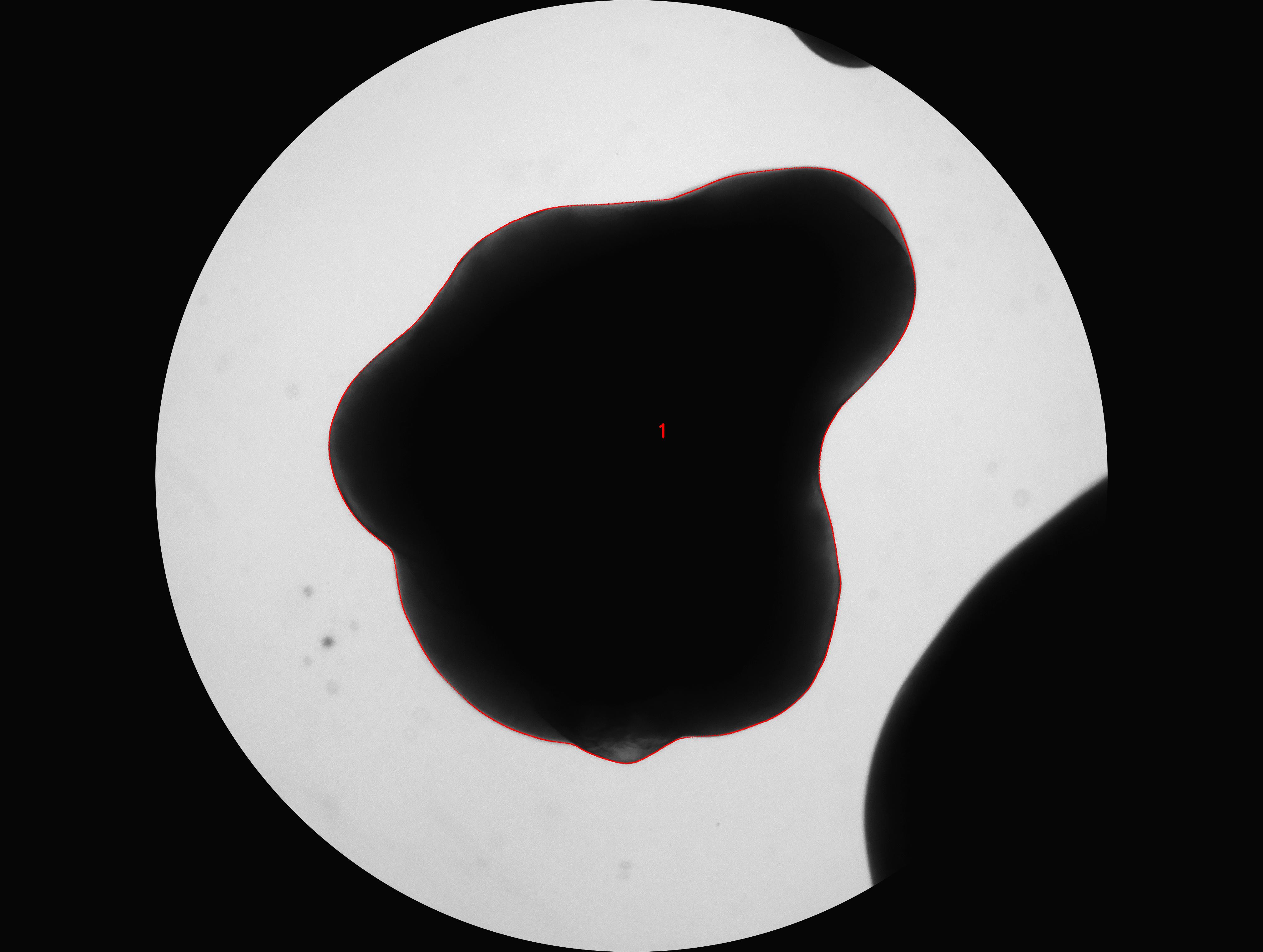

Supplement: Supplementary file 11 — Source data Fig. 3 [file 44319_2025_619_MOESM11_ESM.zip › Figure 3/C,D,F,G/Raw images_mask/OS_day90/MN 11C1 B C7 D90 2x/R_Day 90_0017.jpg]

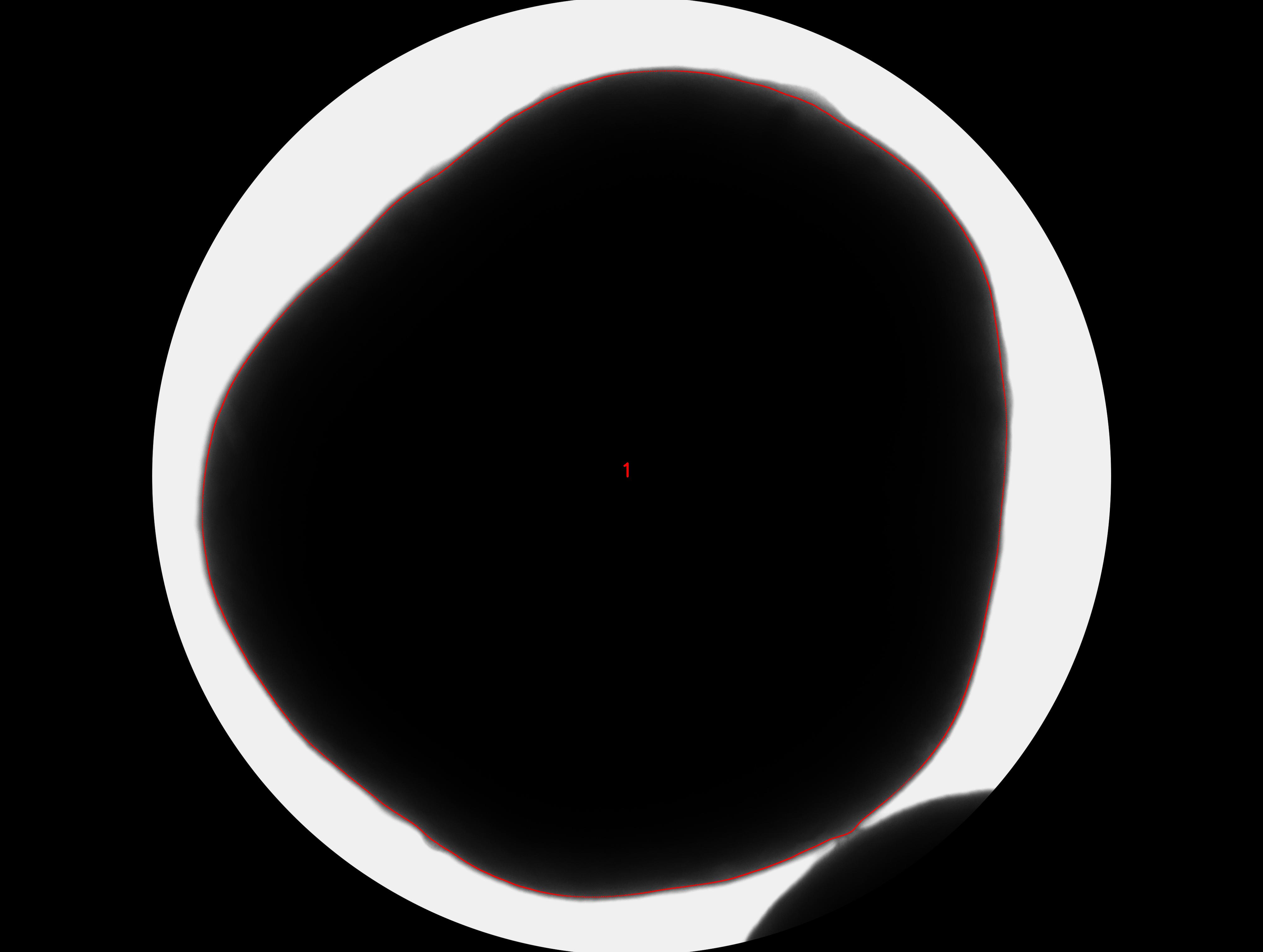

Supplement: Supplementary file 11 — Source data Fig. 3 [file 44319_2025_619_MOESM11_ESM.zip › Figure 3/C,D,F,G/Raw images_mask/RC_day90/GA 11C1 C63 D90 2x/R_GA_C63 Day 90_0004.jpg]

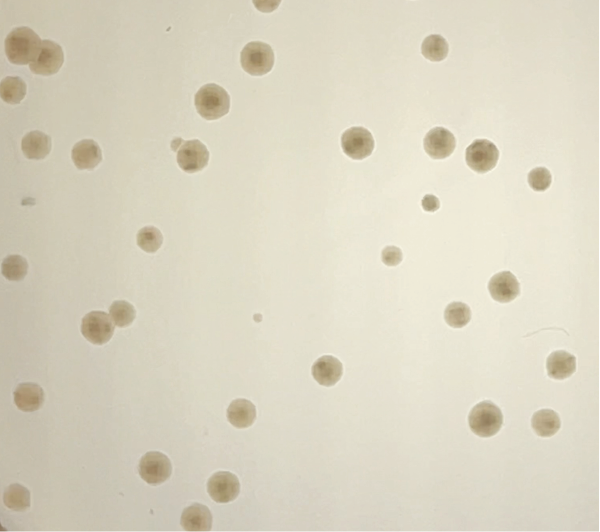

Supplement: Supplementary file 12 — Source data Fig. 4 [file 44319_2025_619_MOESM12_ESM.zip › Figure 4/A/Frame_movie2.png]

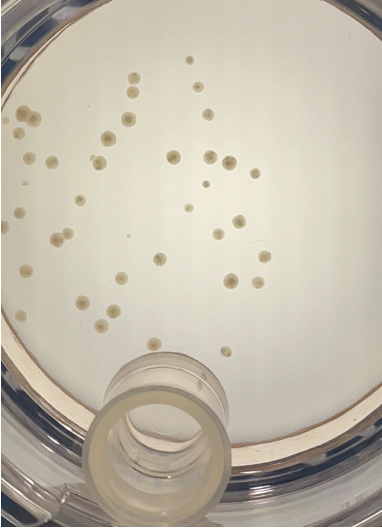

Supplement: Supplementary file 12 — Source data Fig. 4 [file 44319_2025_619_MOESM12_ESM.zip › Figure 4/A/Frame_movie1.png]
