# Supplementary material for: Increased reproducibility of brain organoids through controlled fluid dynamics
Source: EMBO Rep. 2025 Nov 19;26(24):6209–39. doi: 10.1038/s44319-025-00619-x (PMC12715241; doi:10.1038/s44319-025-00619-x)
Supplement: Supplementary file 14 — Expanded View Figures [file 44319_2025_619_MOESM14_ESM.pdf]

Expanded View Figures

Figure EV1. Prolonged aggregation time enhances EBs morphological homogeneity.

(**A**) Bar plots showing quantitative comparisons of ten morphological parameters between PA (blue) ($n = 102$, five batches) and RC (yellow) ($n = 88$, three batches) EBs at day 6. Each dot represents an individual EB. The height of the columns represents the median and the error bars represent the interquartile range (i.e., the range between the 25th percentile and the 75th percentile). Statistically significant differences between conditions are indicated. Mann–Whitney test, ns not significant, *$p < 0.05$, ****$p < 0.0001$. Exact p values: area ($p < 0.0001$), perimeter ($p = 0.7908$), average radius ($p < 0.0001$), roundness ($p < 0.0001$), max feret ($p < 0.0001$), min feret ($p < 0.0001$), mean curvature ($p = 0.0353$), std curvature ($p < 0.0001$), std curvature × R0 ($p < 0.0001$), DNE ($p < 0.0001$). (**B**) Bar plots representing the absolute relative median absolute deviation (MAD) for each morphological parameter, comparing inter-batch variability between PA (blue) ($n = 5$ batches) and RC (yellow) ($n = 3$ batches) conditions at day 6. The height of the columns represents the median and each dot represents one batch, with error bars indicating interquartile ranges. Significant differences are marked. Mann–Whitney test, ns not significant, *$p < 0.05$. Exact p values: area ($p = 0.0357$), perimeter ($p = 0.0357$), average radius ($p = 0.0357$), roundness ($p = 0.0357$), max feret ($p = 0.0357$), min feret ($p = 0.0357$), mean curvature ($p = 0.0357$), std curvature ($p = 0.3929$), std curvature × R0 ($p = 0.0357$), DNE ($p = 0.2500$). (**C**) Bar plots depicting ten morphological parameters of EBs at day 6, analyzed across two independent iPSC lines (Line 1 and Line 2) cultured under PA (blue) (Line 1:$n = 39$, Line 2:$n = 63$) and RC (yellow) (Line 1:$n = 16$, Line 2:$n = 72$) protocols. Each dot represents an individual EB. The height of the bars represents the mean, and error bars indicate the standard error of the mean (SEM). Statistical significance was assessed using a two-way ANOVA with Šidák's multiple comparisons test, with p values adjusted for multiple comparisons. ns not significant, *$p < 0.05$, **$p < 0.01$, ****$p < 0.0001$. Exact p values: area (PA $p = 0.3743$, RC $p = 0.6160$), perimeter (PA $p = 0.2463$, RC $p = 0.8357$), average radius (PA $p = 0.0340$, RC $p = 0.6476$), roundness (PA $p = 0.0065$, RC $p = 0.8687$), max feret (PA $p = 0.0339$, RC $p = 0.7193$), min feret (PA $p = 0.0941$, RC $p = 0.2902$), mean curvature (PA $p = 0.0071$, RC $p = 0.5519$), std curvature (PA $p = 0.5908$, RC $p < 0.0001$), std curvature × R0 (PA $p = 0.0571$, RC $p = 0.0058$), DNE (PA $p = 0.0042$, RC $p < 0.0001$).

 © The Author(s)

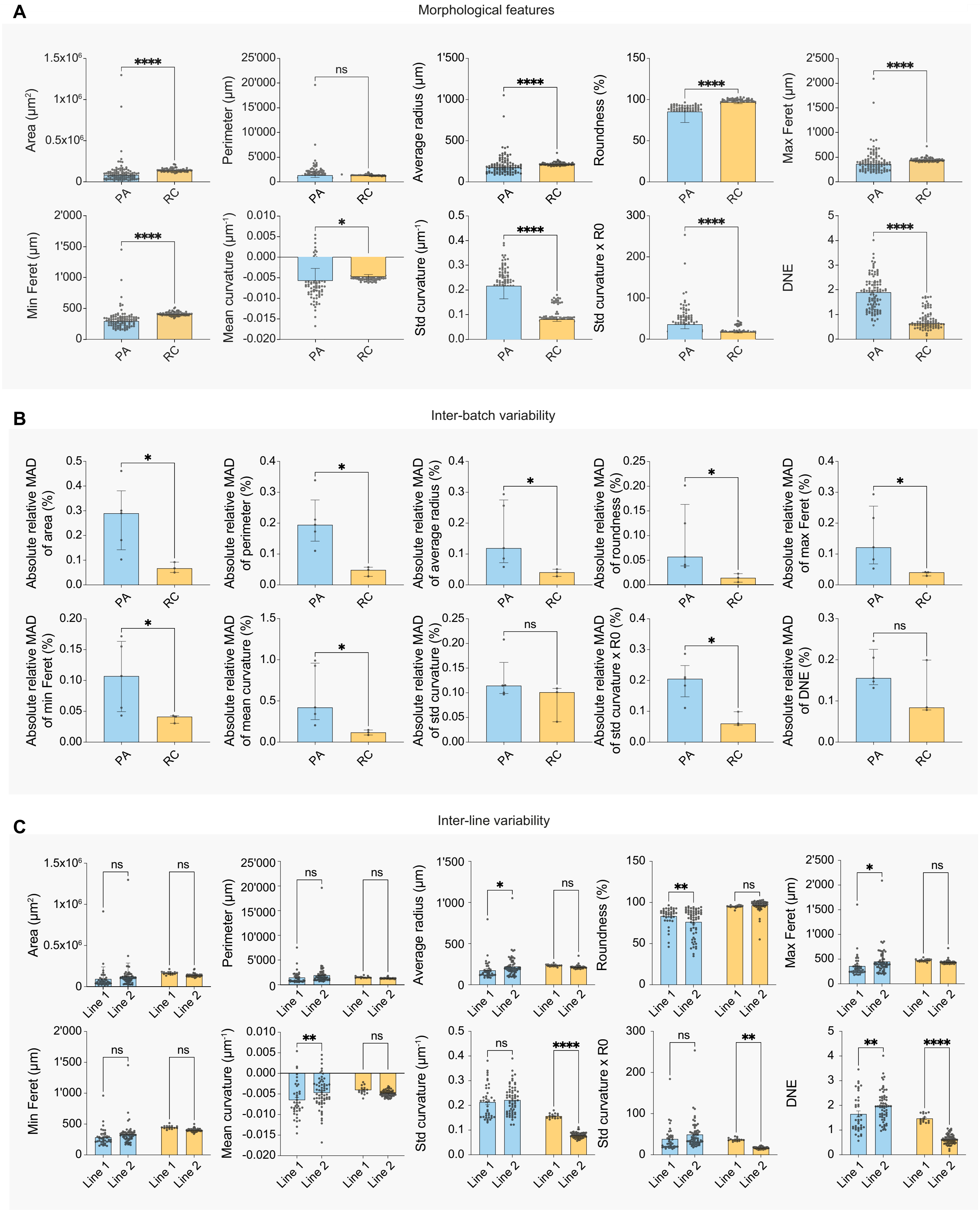
A
Morphological features
Area (µm²)
Perimeter (µm)
Average radius (µm)
Roundness (%)
Max Feret (µm)
Min Feret (µm)
Mean curvature (µm⁻¹)
Std curvature (µm⁻¹)
Std curvature x R0
DNE
PA
RC
ns
B
Inter-batch variability
Absolute relative MAD of area (%)
Absolute relative MAD of perimeter (%)
Absolute relative MAD of average radius (%)
Absolute relative MAD of roundness (%)
Absolute relative MAD of max Feret (%)
Absolute relative MAD of min Feret (%)
Absolute relative MAD of mean curvature (%)
Absolute relative MAD of std curvature (%)
Absolute relative MAD of std curvature x R0 (%)
Absolute relative MAD of DNE (%)
C
Inter-line variability
Line 1
Line 2

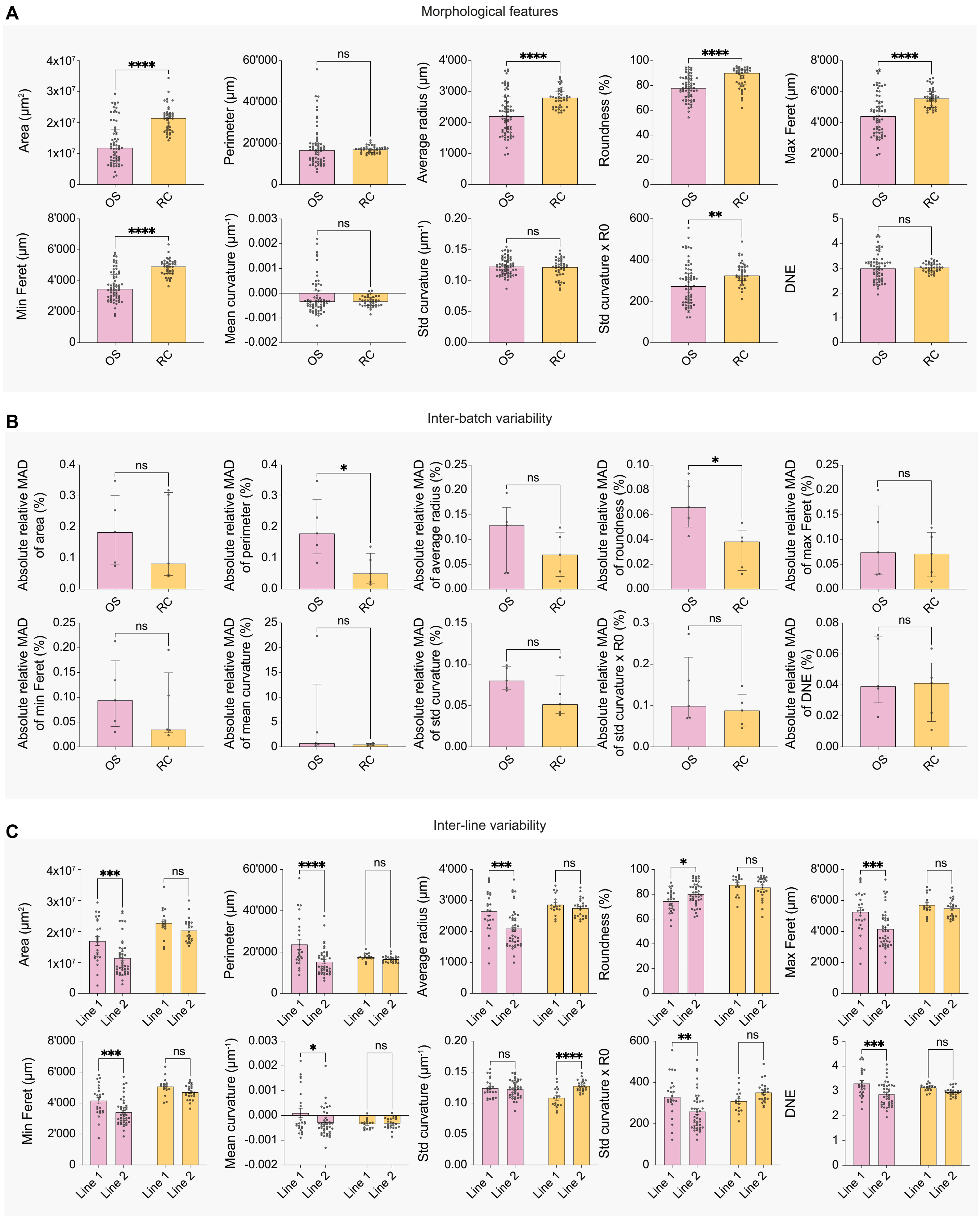
A
Morphological features
Area (μm²)
Perimeter (μm)
Average radius (μm)
Roundness (%)
Max Feret (μm)
Min Feret (μm)
Mean curvature (μm⁻¹)
Std curvature (μm⁻¹)
Std curvature x R0
DNE
OS
RC
ns
****
**
B
Inter-batch variability
Absolute relative MAD of area (%)
Absolute relative MAD of perimeter (%)
Absolute relative MAD of average radius (%)
Absolute relative MAD of roundness (%)
Absolute relative MAD of max Feret (%)
Absolute relative MAD of min Feret (%)
Absolute relative MAD of mean curvature (%)
Absolute relative MAD of std curvature (%)
Absolute relative MAD of std curvature x R0 (%)
Absolute relative MAD of DNE (%)
*
C
Inter-line variability
Line 1
Line 2
***

 © The Author(s)

Figure EV2. RC apparatus improves morphological homogeneity across batches and cell lines.

(**A**) Bar plots quantifying ten morphological parameters in OS (pink, $n = 67$, five batches) and RC (yellow, $n = 41$, five batches) organoids at day 90. Each dot represents an individual organoid. The height of the bars represents the median, and the error bars correspond to the interquartile range (i.e., the range between the 25th and 75th percentiles). Statistically significant differences between conditions are indicated. Mann–Whitney test, ns not significant, $^{**}p < 0.01$, $^{****}p < 0.0001$. Exact p values: area ($p < 0.0001$), perimeter ($p = 0.5366$), average radius ($p < 0.0001$), roundness ($p < 0.0001$), max feret ($p < 0.0001$), min feret ($p < 0.0001$), mean curvature ($p = 0.8205$), std curvature ($p = 0.5038$), std curvature × R0 ($p = 0.0011$), DNE ($p = 0.5366$). (**B**) Bar plots showing the absolute relative median absolute deviation (MAD) for each morphological parameter, comparing inter-batch variability between OS (pink,$n = 5$ batches) and RC (yellow, $n = 5$ batches) conditions at day 90. The height of the columns represents the median and each dot represents one batch, and error bars indicate interquartile ranges. Significant differences are marked. Mann–Whitney test, ns not significant, $^{*}p < 0.05$. Exact p values: area ($p = 0.5476$), perimeter ($p = 0.0317$), average radius ($p = 0.4206$), roundness ($p = 0.0159$), max feret ($p = 0.6905$), min feret ($p = 0.5476$), mean curvature ($p = 0.3095$), std curvature ($p = 0.1508$), std curvature × R0 ($p = 0.4206$), DNE ($p = 0.6905$). (**C**) Bar plots depicting ten morphological parameters of organoids at day 90, analyzed across two independent iPSC lines (Line 1 and Line 2) cultured under OS (pink) (Line 1:$n = 24$, Line 2:$n = 43$) and RC (yellow) (Line 1:$n = 17$, Line 2:$n = 24$) protocols. Each dot represents an individual organoid. The height of the bars represents the mean, and error bars indicate the standard error of the mean (SEM). Statistical significance was assessed using a two-way ANOVA with Šidák's multiple comparisons test, with p values adjusted for multiple comparisons. ns not significant, $^{*}p < 0.05$, $^{**}p < 0.01$, $^{***}p < 0.001$, **** $p < 0.0001$. Exact p values: area (OS $p = 0.0005$, RC $p = 0.3297$), perimeter (OS $p < 0.0001$, RC $p = 0.8638$), average radius (OS $p = 0.0003$, RC $p = 0.7651$), roundness (OS $p = 0.0278$, RC $p = 0.7080$), max feret (OS $p = 0.0002$, RC $p = 0.7798$), min feret (OS $p = 0.0006$, RC $p = 0.2890$), mean curvature (OS $p = 0.0143$, RC $p = 0.9863$), std curvature (OS $p = 0.9250$, RC $p < 0.0001$), std curvature × R0 (OS $p = 0.0018$, RC $p = 0.2055$), DNE (OS $p = 0.0001$, RC $p = 0.3061$).

© The Author(s)

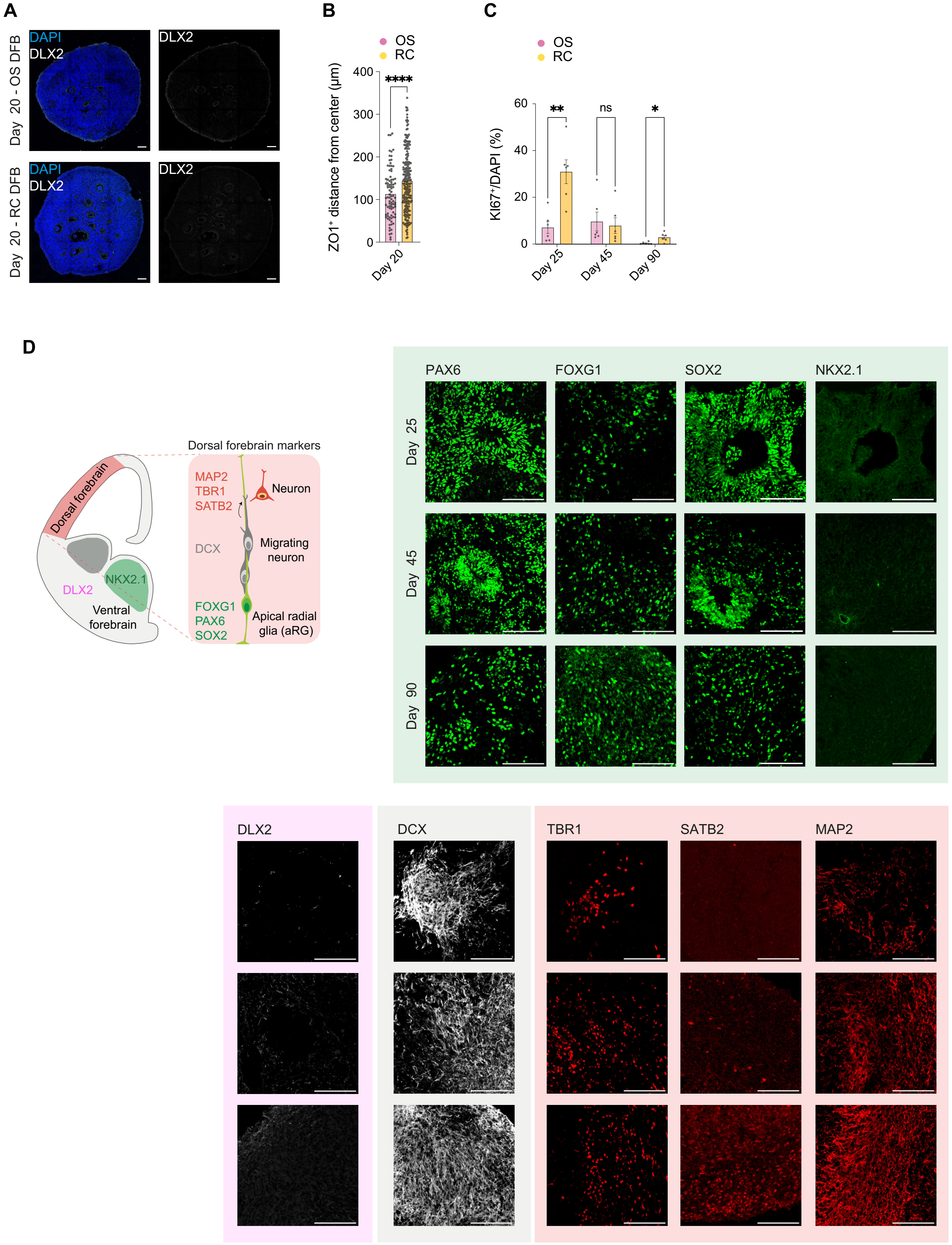
A
Day 20 - OS DFB
Day 20 - RC DFB
DAPI
DLX2
DLX2
B
OS
RC
ZO1+ distance from center (µm)
****
Day 20
C
OS
RC
KI67+/DAPI (%)
**
ns
*
Day 25
Day 45
Day 90
D
Dorsal forebrain markers
Dorsal forebrain
DLX2
NKX2.1
Ventral forebrain
MAP2
TBR1
SATB2
Neuron
DCX
Migrating neuron
FOXG1
PAX6
SOX2
Apical radial glia (aRG)
PAX6
FOXG1
SOX2
NKX2.1
Day 25
Day 45
Day 90
DLX2
DCX
TBR1
SATB2
MAP2

© The Author(s)

Figure EV3. RC organoids do not show expression of ventral forebrain markers.

(**A**) Immunofluorescence images for DLX2 (white) and DAPI (blue) in OS and RC organoids at day 20. Scale bars: 100 µm. (**B**) Quantification of of ZO1⁺ rosette distance from the organoid center. Each dot represents a single ZO1⁺ rosette ($n = 3$ batches per line). Data were presented as mean ± SEM. Statistical analysis was performed using unpaired two-tailed Welch's *t*-test with correction for multiple comparisons using the two-stage step-up method of Benjamini, Krieger, and Yekutieli. ****$p < 0.0001$. Exact *p* value: $p = 0.000104$. (**C**) Quantification of the proportion of KI67⁺/DAPI⁺ cells across time points ($n = 3$ batches per line). Data were presented as mean ± SEM. Statistical analysis was performed using an unpaired two-tailed Welch's *t*-test with correction for multiple comparisons using the two-stage step-up method of Benjamini, Krieger, and Yekutieli. ns not significant, *$p < 0.05$, **$p < 0.01$. Exact *p* values: day 25 ($p = 0.003616$), day 45 ($p = 0.746061$), day 90 ($p = 0.020226$). (**D**) Schematic representation of dorsal and ventral forebrain markers, illustrating marker expression patterns in organoids. Immunofluorescence staining at days 25, 45, and 90, showing expression of PAX6, FOXG1, SOX2, and NKX2.1 (green), DCX (white), TBR1, SATB2, and MAP2 (red) and DLX2 (violet) across different time points. Scale bars: 100 µm.

© The Author(s)

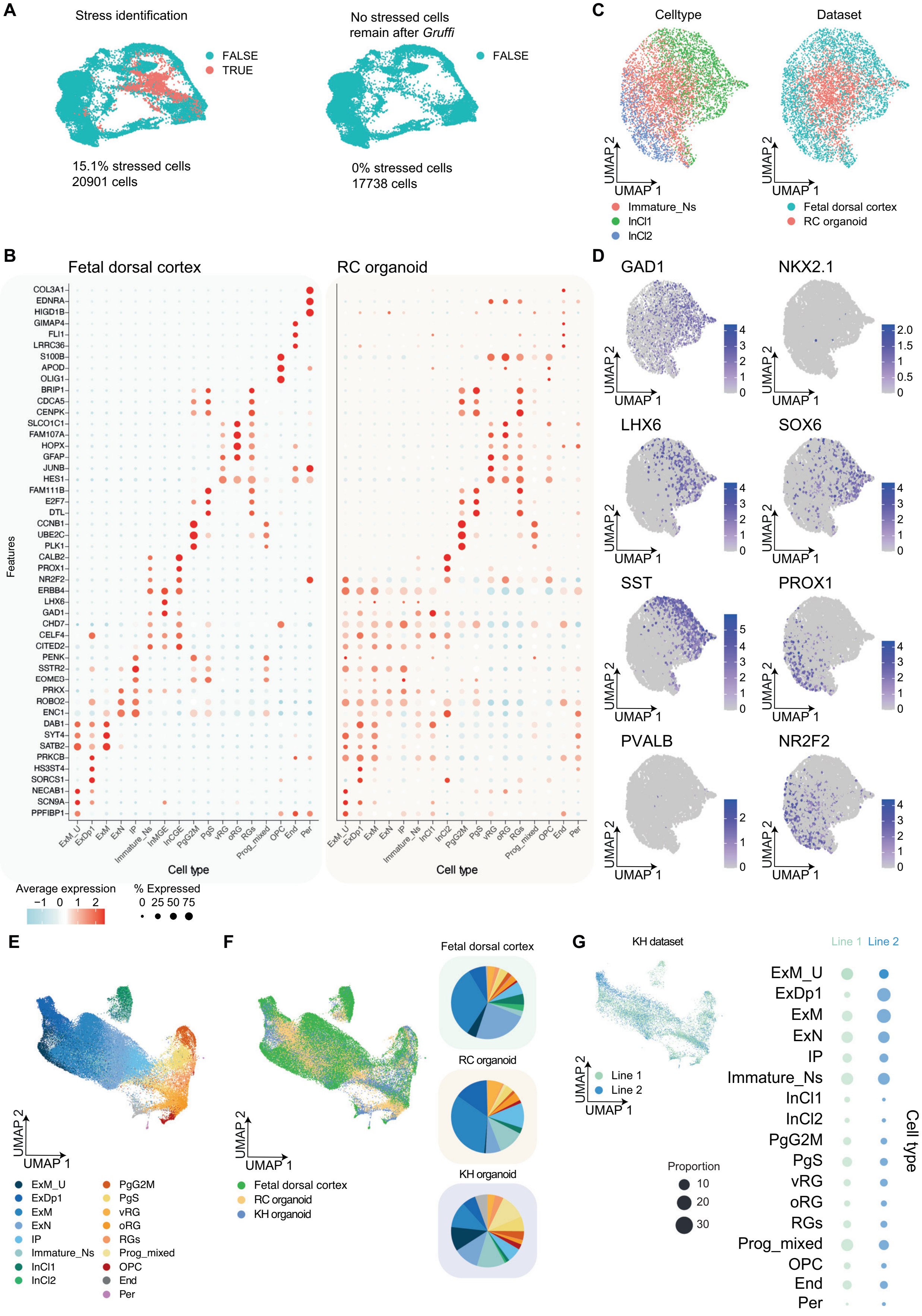
A
Stress identification
FALSE
TRUE
15.1% stressed cells
20901 cells
No stressed cells
remain after Gruffi
FALSE
0% stressed cells
17738 cells
C
Celltype
Dataset
UMAP 2
UMAP 1
Immature_Ns
InCl1
InCl2
Fetal dorsal cortex
RC organoid
B
Fetal dorsal cortex
RC organoid
Features
COL3A1
EDNRA
HIGD1B
GIMAP4
FLI1
LRRC36
S100B
APOD
OLIG1
BRIP1
CDCA5
CENPK
SLCO1C1
FAM107A
HOPX
GFAP
JUNB
HES1
FAM111B
E2F7
DTL
CCNB1
UBE2C
PLK1
CALB2
PROX1
NR2F2
ERBB4
LHX6
GAD1
CHD7
CELF4
CITED2
PENK
SSTR2
EOMES
PRKX
ROBO2
ENC1
DAB1
SYT4
SATB2
PRKCB
HS3ST4
SORCS1
NECAB1
SCN9A
PPFIBP1
ExM_U
ExDp1
ExM
ExN
IP
Immature_Ns
InMGE
InCGE
PgG2M
PgS
vRG
oRG
RGs
Prog_mixed
OPC
End
Per
InCl1
InCl2
Cell type
Average expression
−1 0 1 2
% Expressed
0 25 50 75
D
GAD1
NKX2.1
LHX6
SOX6
SST
PROX1
PVALB
NR2F2
E
F
Fetal dorsal cortex
RC organoid
KH organoid
ExM_U
ExDp1
ExM
ExN
IP
Immature_Ns
InCl1
InCl2
PgG2M
PgS
vRG
oRG
RGs
Prog_mixed
OPC
End
Per
G
KH dataset
Line 1
Line 2
Proportion
10
20
30
Cell type

 © The Author(s)

Figure EV4. Quality control and integrative transcriptomic analysis of RC organoids.

(**A**) Identification and removal of stressed cells using the GRUFFI algorithm (Vertesy et al, 2022). The left UMAP plot displays the proportion of stressed cells (red) in the dataset before filtering, with 15.1% of cells (20,901 total) identified as stressed. The right UMAP plot shows the dataset after GRUFFI filtering, where all stressed cells have been removed, retaining 17,738 high-quality cells for downstream analysis. (**B**) Dotplot showing the expression of canonical marker genes across annotated cell types in the fetal cortex (left) and RC organoids (right), confirming similar transcriptional patterns between corresponding populations. Dot size represents the percentage of expressing cells; color intensity indicates average expression. (**C**) UMAP representations highlighting the three interneuron-related clusters: Immature_Ns, InMGE, and InCGE. Cells are colored by cell type (left) or dataset of origin (right), showing separation between fetal and organoid-derived interneurons. (**D**) Feature plots of selected interneuron markers (GAD1, NKX2.1, LHX6, SOX6, SST, PROX1, PVALB, and NR2F2). (**E**) UMAP of the integrated dataset including fetal cortex (Polioudakis et al, 2019), RC organoids, and KH organoids (Khan et al, 2020). (**F**) Right: UMAP of the integrated dataset color-coded by dataset of origin. Left: Pie charts indicating the proportional distribution of different cell types in each dataset. (**G**) Proportional representation of cell types across the two KH organoid lines, visualized through dot plots. Cell types include: ExM_U (maturing excitatory upper enriched), ExDp1 (excitatory deep layer 1), ExM (maturing excitatory), ExN (migrating excitatory), IP (intermediate progenitors), Immature_Ns (immature neurons), InCl1 (interneuron cluster 1), InCl2 (interneuron cluster 2), PgG2M (cycling progenitors in G2/M phase), PgS (cycling progenitors in S phase), vRG (ventricular radial glia), oRG (outer radial glia), RGs (radial glial populations), Prog_mixed (mixed progenitors), OPC (oligodendrocyte progenitor cells), End (Endothelial cells) and Per (pericytes).

© The Author(s)

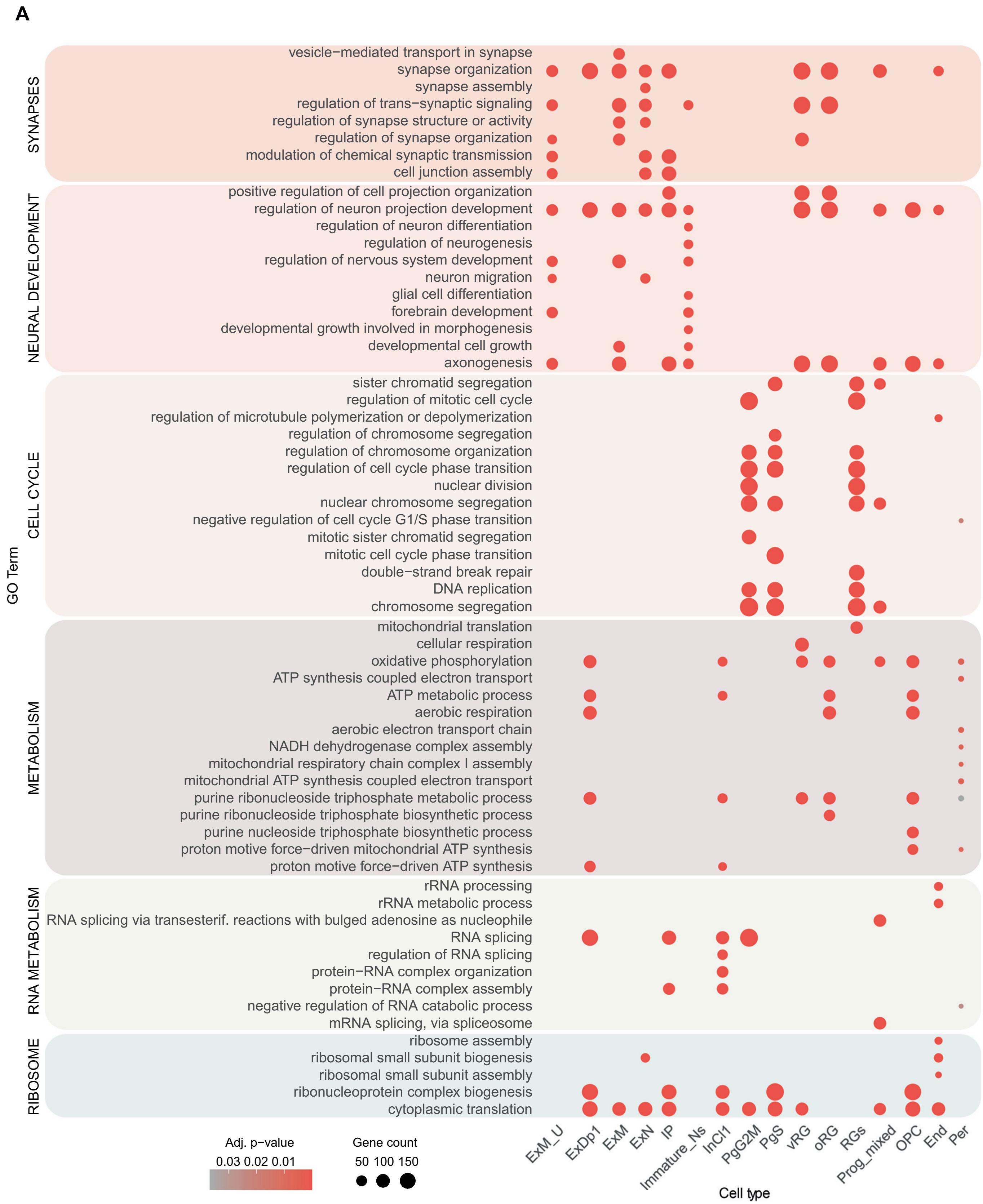
A
SYNAPSES
NEURAL DEVELOPMENT
CELL CYCLE
METABOLISM
RNA METABOLISM
RIBOSOME
GO Term
vesicle−mediated transport in synapse
synapse organization
synapse assembly
regulation of trans−synaptic signaling
regulation of synapse structure or activity
regulation of synapse organization
modulation of chemical synaptic transmission
cell junction assembly
positive regulation of cell projection organization
regulation of neuron projection development
regulation of neuron differentiation
regulation of neurogenesis
regulation of nervous system development
neuron migration
glial cell differentiation
forebrain development
developmental growth involved in morphogenesis
developmental cell growth
axonogenesis
sister chromatid segregation
regulation of mitotic cell cycle
regulation of microtubule polymerization or depolymerization
regulation of chromosome segregation
regulation of chromosome organization
regulation of cell cycle phase transition
nuclear division
nuclear chromosome segregation
negative regulation of cell cycle G1/S phase transition
mitotic sister chromatid segregation
mitotic cell cycle phase transition
double−strand break repair
DNA replication
chromosome segregation
mitochondrial translation
cellular respiration
oxidative phosphorylation
ATP synthesis coupled electron transport
ATP metabolic process
aerobic respiration
aerobic electron transport chain
NADH dehydrogenase complex assembly
mitochondrial respiratory chain complex I assembly
mitochondrial ATP synthesis coupled electron transport
purine ribonucleoside triphosphate metabolic process
purine ribonucleoside triphosphate biosynthetic process
purine nucleoside triphosphate biosynthetic process
proton motive force−driven mitochondrial ATP synthesis
proton motive force−driven ATP synthesis
rRNA processing
rRNA metabolic process
RNA splicing via transesterif. reactions with bulged adenosine as nucleophile
RNA splicing
regulation of RNA splicing
protein−RNA complex organization
protein−RNA complex assembly
negative regulation of RNA catabolic process
mRNA splicing, via spliceosome
ribosome assembly
ribosomal small subunit biogenesis
ribosomal small subunit assembly
ribonucleoprotein complex biogenesis
cytoplasmic translation
Adj. p−value
0.03 0.02 0.01
Gene count
50 100 150
ExM_U
ExDp1
ExM
ExN
IP
Immature_Ns
InCl1
PgG2M
PgS
vRG
oRG
RGs
Prog_mixed
OPC
End
Per
Cell type

 © The Author(s)

Figure EV5. Functional annotation.

(**A**) Gene Ontology (GO) enrichment analysis of biological processes across different cell types in the dataset. The dot size corresponds to the number of genes enriched within each GO term, while the color intensity reflects the adjusted *p* value, indicating the significance of enrichment. Cell types include: ExM_U (maturing excitatory upper enriched), ExDp1 (excitatory deep layer 1), ExM (maturing excitatory), ExN (migrating excitatory), IP (intermediate progenitors), Immature_Ns (immature neurons), InCl1 (interneuron cluster 1), InCl2 (interneuron cluster 2), PgG2M (cycling progenitors in G2/M phase), PgS (cycling progenitors in S phase), vRG (ventricular radial glia), oRG (outer radial glia), RGs (radial glial populations), Prog_mixed (mixed progenitors), OPC (oligodendrocyte progenitor cells), End (Endothelial cells) and Per (pericytes). Differentially expressed genes for each cluster were identified using the Wilcoxon rank-sum test (Seurat FindAllMarkers function). GO enrichment analysis was performed with the clusterProfiler package (enrichGO function) using the Benjamini–Hochberg method for multiple testing correction (*q* value cutoff = 0.05).

© The Author(s)
